# Photoredox-Catalyzed Radical Coupling of C7-Chloromethyl-Substituted Thiazolino Ring-Fused 2-Pyridones with Quinoxalinones

**DOI:** 10.1021/acs.joc.4c01224

**Published:** 2024-07-25

**Authors:** Victor Hellgren, Pardeep Singh, Abhilash Kulkarni, Niusha Bagheri, Jerker Widengren, Gopinathan Manavalan, Fredrik Almqvist

**Affiliations:** † Department of Chemistry, Umeå University, SE-90187 Umeå, Sweden; ‡ Department of Applied Physics, Royal Institute of Technology (KTH), SE-10691 Stockholm, Sweden; § Umeå Centre for Microbial Research, UCMR, Umeå University, SE-90187 Umeå, Sweden

## Abstract

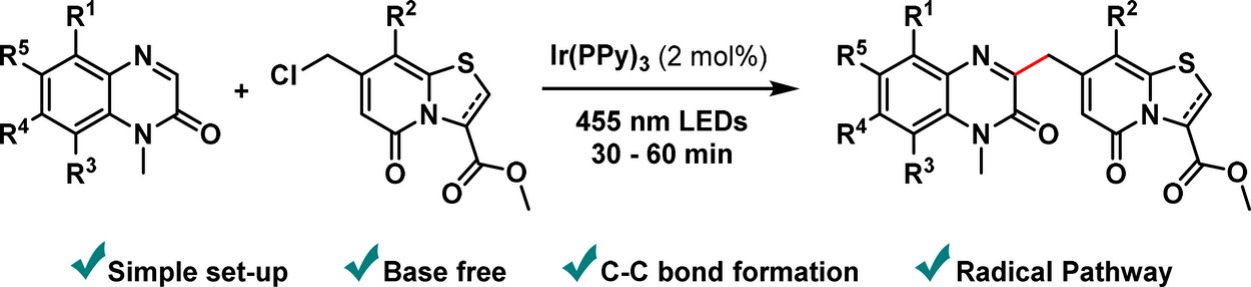

We have developed an Ir(PPy)_3_ photoredox-catalyzed cross-coupling reaction that allows installation of quinoxalinones at the C7 position of thiazolino ring-fused 2-pyridones (TRPs) under mild conditions. The methodology tolerates various substituted quinoxalinones and biologically relevant substituents on the C8 position of the TRP. The TRP scaffold has large potential in the development of lead compounds, and while the coupled products are interesting from a drug-development perspective, the methodology will be useful for developing more potent and drug-like TRP-based candidates.

The thiazolino ring-fused 2-pyridone (TRP) scaffold has been a foundational element in the development of diverse biologically active compounds. The substituents on the pyridone ring influence the potency and determine which target an analogue is active toward. For example, compound **A** (Figure 1A) has broad spectrum bactericidal activity toward, among others, methicillin-resistant *S. aureus* (MRSA), vancomycin-resistant enterococci (VRE), and streptococcal species.^1^ Compound **B** (Figure 1A) is capable of inhibiting the aggregation of Aβ(1–40) amyloid fibers,^2^ and compound **C** (Figure 1A) belongs to a class that prevents biofilm formation of *Mycobacterium tuberculosis* (*Mtb*) and restores the effect of isoniazid (INH) on INH-resistant *Mtb* strains.^3,4^ Compound **D** (Figure 1A) has antivirulent properties toward uropathogenic *E. coli*(5) and was developed for fluorescent labeling. In the development of new analogues, the substituent on the C7 position has been especially important for activity, and finding new methods to functionalize this position is therefore important to access more potent and drug-like derivatives. While current methods already exist (Figure 1B), none have made use of photocatalyzed processes which offers unique reactivity and allow introduction of substituents that are unattainable with other methods. Exploring photocatalyzed methods with TRPs is therefore interesting as late-stage modification is already challenging with this complex scaffold containing multiple functionalities which complicates selective modifications. In this work, we have developed a photoredox-catalyzed cross-coupling reaction (Figure 1C) that allows installation of substituted quinoxalinones at the C7 position. The reaction proceeds under mild conditions, making use of single-electron transfer (SET) from Ir(PPy)_3_. Historically, bulkier groups such as naphthalene at the C7 position have generated the most successful analogues. The similarity in size to naphthalene and the heterocyclic nature of quinoxalinones therefore make their fusion with TRPs an interesting pairing. Additionally, functionalized quinoxalinones are privileged structures in medicinal chemistry and are found in a large variety of lead compounds with a wide range of activities.^6−9^

**Figure 1:**
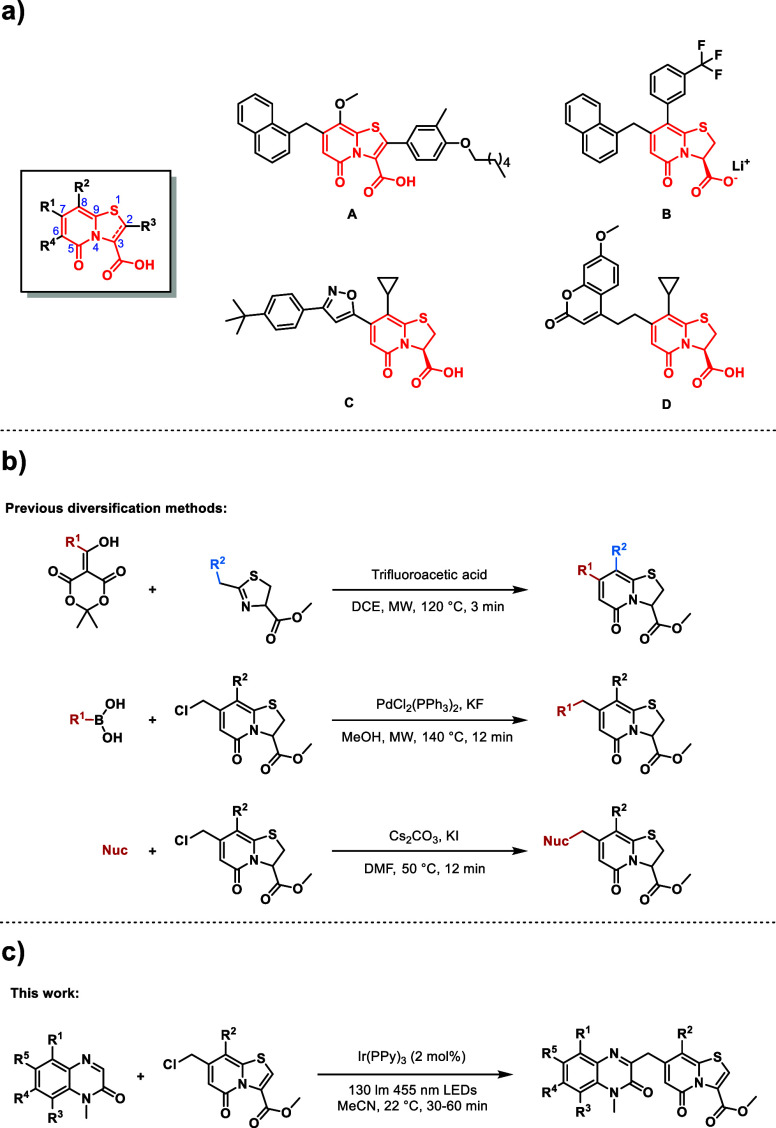
(a) Examples of active analogues based on the TRP scaffold. (b) Previous methods to diversify the C7 position. (c) Photoredox-catalyzed cross-coupling between a C7-methyl chloride-substituted TRP and substituted quinoxalinones.

While the generated compounds may possess interesting biological activity, the developed methodology is a gateway toward other, previously unexplored, C7-substituents on the TRP scaffold.

## Condition Screening

The reaction was initially carried out with a cyclopropyl-substituted chloro-TRP (**2a**, 1.0 equiv) and an unsubstituted quinoxalinone (**1a**, 1.2 equiv) dissolved in MeCN (9.4 mL/mmol **2a**) (Table 1, entry 1). The mixture was irradiated with 455 nm LEDs with a luminous flux of 130 lm for 300 min, which generated the desired product **3aa** (36%, entry 1). Intrigued by the results, we investigated if the reaction could be made more efficient by introducing a photoredox catalyst. Screening of different photoredox catalysts (entries 2–6) revealed that Ir(PPy)_3_ was the most suitable, facilitating the reaction in 30 min with 56% yield. Interestingly, the yields when using organic photoredox catalysts (entries 3–5) were similar to when no catalyst was added, suggesting that their presence had no effect on the reaction. Solvents were then screened (entries 7–10), which showed that the reaction proceeded best in MeCN but also worked well in DCM and DCE. Based on the hypothesized mechanism, we expected that the addition of a base would be favorable; however, the addition of K_2_CO_3_ (1.0 equiv, entry 11) gave similar results as without a base, and the addition of TEA (1.0 equiv., entry 12) resulted in a lower yield. Using 2.0 equiv of **1a** (entry 13) instead of 1.2 equiv resulted in a small increase in yield, from 56% to 64%, but this increase did not justify its continued use.

**Table 1:**
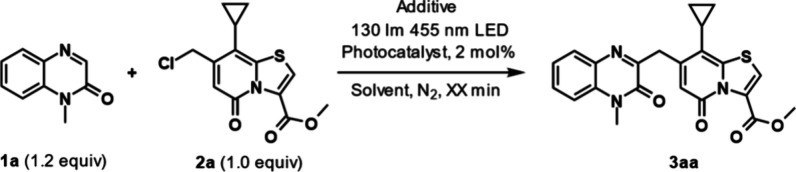
Optimization of the Conditions for Photoredox-Catalyzed Cross-Coupling between **1a** and **2a**

entry	photocatalyst (additive)	solvent	time (min)	yield (%)^a^
1	none	MeCN	300	36
2	**Ir(PPy)**_**3**_	**MeCN**	**30**	**56**
3	4CzIPN	MeCN	390	40
4^b^	Eosin Y	MeCN	420	40
5^b^	Rose Bengal	MeCN	300	41
6	[Ir(dtbbpy)(ppy)_2_]PF_6_	MeCN	60	25
7	Ir(PPy)_3_	DCM	30	53
8	Ir(PPy)_3_	THF	30	21
9	Ir(PPy)_3_	DMF	30	35
10	Ir(PPy)_3_	DCE	30	50
11	Ir(PPy)_3_ (1 equiv of K_2_CO_3_)	MeCN	30	59
12	Ir(PPy)_3_ (1 equiv of TEA)	MeCN	30	44
13^c^	Ir(PPy)_3_	MeCN	30	64
14^d^	Ir(PPy)_3_	MeCN	30	32
15^e^	Ir(PPy)_3_	MeCN	780	48

a Isolated yields.

b Initially irradiated for 16 h at their λ_max_, 527 nm.

c 2 equiv of **1a**.

d No degassing.

e Done on 501 mg scale of **2a**, and the product was recrystallized from MeOH.

The necessity of degassing the reaction mixture prior to irradiation was also investigated, which showed that the reaction is able to proceed with oxygen present but results in a lower yield (entry 14). The reaction also proceeded well with an increase in reaction time when scaled to 501 mg of **2a**, giving product **3aa** in 48% yield after recrystallization from MeOH (entry 15). A series of control experiments was also performed, confirming that the reaction does not proceed efficiently when irradiated at 395 nm, does not proceed at all without light or with only heating, and requires dry conditions (Supporting Information, Table S1).

## Quinoxalinone Scope

**Scheme 1:**
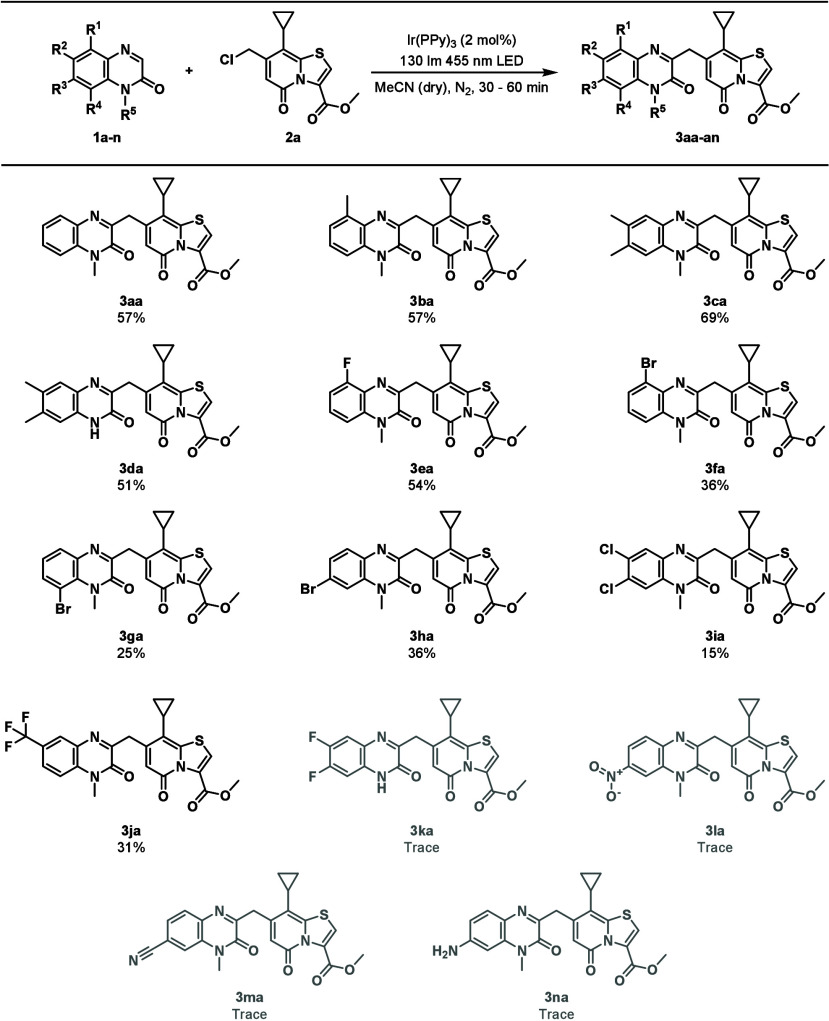
Substrate Scope of **2a** with Quinoxalinones

With the proper conditions in hand, the scope of the reaction was tested, first with differently substituted *N*-methyl quinoxalinones (Scheme 1). Introduction of one or two methyl groups on the quinoxalinone was well tolerated, giving the expected products **3ba** and **3ca** in 57% and 69% yields, respectively. The reaction also proceeded well with a 6,7-dimethylated quinoxalinone without a methylated amide nitrogen, giving **3da** in 51% yield. Changing the substitution from 6,7-dimethyl to 6,7-difluoro while keeping the amide nitrogen free resulted in a complex mixture with traces of **3ka**. Substitution with strongly electron-withdrawing groups such as NO_2_ and CN required longer reaction times before the TRP starting material was consumed (up to 1 h) and gave products **3la** and **3ma**, respectively, with inseparable impurities. Replacing the electron-withdrawing group with an amine gave product **3na** with inseparable impurities. Halogen substituents (F, Cl, and Br) were tolerated, giving the corresponding products **3ea**–**3fa** in 15–36% yield. Trifluoromethyl-substituted quinoxalinone was also tolerated, giving product **3ja** in 31% yield. From these experiments it is observed that substitution on the C5 position of the quinoxalinone does not influence the yield significantly. The yields of reactions with C6- and C7-substituted quinoxalinones are more affected by the electronic properties of the substituents compared to the C5-substituted ones as σ-donating substituents increase the yield while σ-withdrawing substituents decrease yields compared to nonsubstituted quinoxalinone.

## TRP Scope

**Scheme 2:**
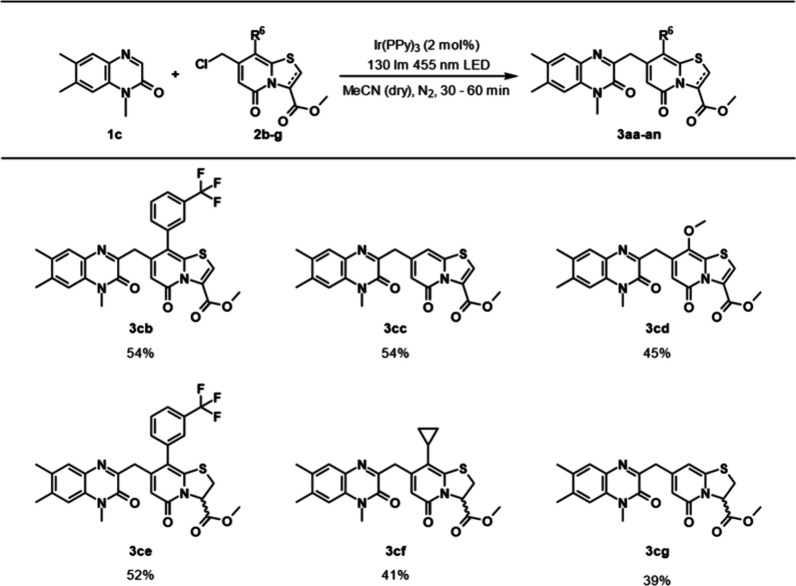
Substrate Scope of TRPs with **1c**

The effects of different substituents on the C8 position of the TRP were investigated using **1c** as the quinoxalinone coupling partner (Scheme 2). The size of the C8 substituent on the TRP did not appear to influence the yield strongly as both *m*-trifluoromethylphenyl-substituted and nonsubstituted TRPs gave their respective products, **3cb** and **3cc,** in 54% yield. Experiments with a methoxy-substituted TRP gave the expected product **3cd** in 45% yield. Overall, the cyclopropyl-substituted TRP gave the highest yield. Attempts with C2–C3-reduced pyridones **2e**–**2g** were also made. Here, the *m*-trifluoromethylphenyl-substituted TRP gave the highest yield, 52%, of the corresponding product **3ce**, while cyclopropyl-substituted (**2f**) and nonsubstituted (**2g**) TRPs gave lower yields of their respective products **3cf** (41%) and **3cg** (39%).

## Mechanistic Investigation

**Figure 2:**
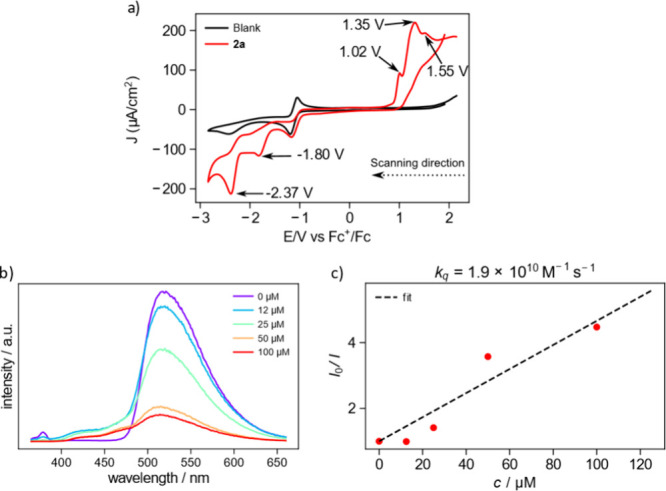
(a) Cyclic voltammogram of the blank (black) and **2a** (red). Conditions: **2a** (5 mM) with NBu_4_PF_6_ (0.1 M) in MeCN (10 mL). Working electrode = cylindrical-shaped glassy carbon electrode (surface area 3.14 mm^2^, 2 mm diameter), counter electrode = Pt sheet, pseudoreference electrode = Ag wire. Scan rate, 50 mV/s. Measurements started at + 2.16 V. Potentials relative to Fc^+^/Fc. (b) Overlapped emission spectra of Ir(PPy)_3_ in air-saturated MeCN with different concentrations of **2a** after excitation at 340 nm. (c) Stern–Volmer plot with the quenching rate constant (*k*_q_) calculated from the linear fit using τ_0(Ir(PPy)3)_ = 1.9 μs^10^ under oxygen-free conditions.

To gain insight into the mechanism of this transformation, cyclic voltammetry and Stern–Volmer experiments were conducted on **2a** (Figure 2).

Cyclic voltammetry showed three irreversible oxidation peaks at 1.55, 1.35 and 1.02 V and two irreversible reduction peaks at −1.80 and −2.37 V. Knowing the redox potential of Ir(PPy)_3,_^10^ the theoretical driving force for the irreversible reduction of **2a** by Ir(PPy)_3_* was calculated using the Rehm–Weller equation.^11^ This gave a negative Gibbs free energy difference (Δ*G*) of −0.12 eV, suggesting a positive driving force for the reaction. The Stern–Volmer experiment showed a linear relationship between the reduction in fluorescence intensity of Ir(PPy)_3_ and the increasing concentration of **2a**, suggesting that **2a** is able to quench Ir(PPy)_3_*.

**Scheme 3:**
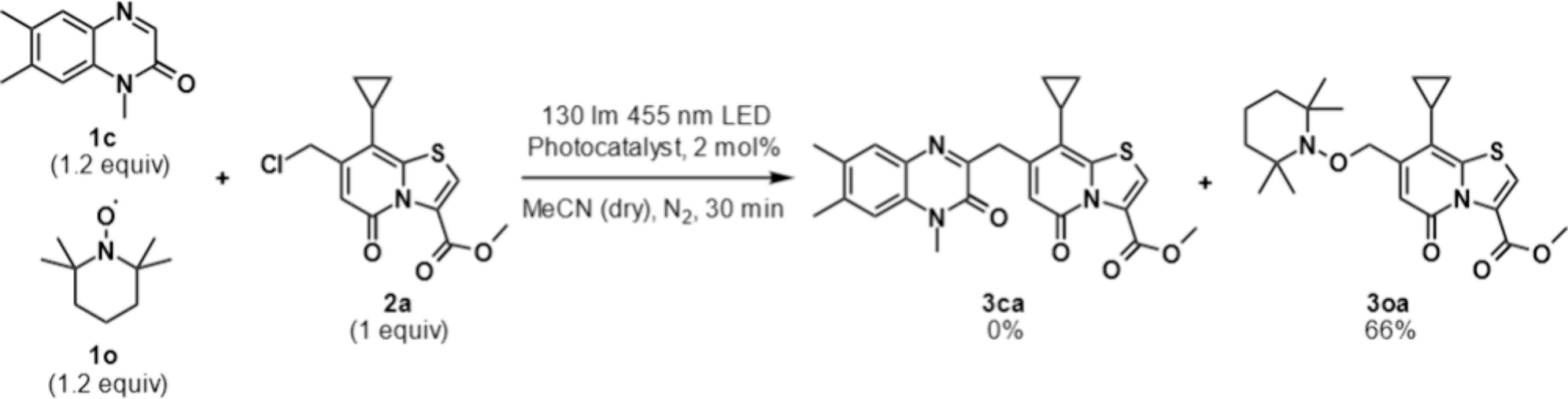
Radical Trapping Experiment with TEMPO

To investigate the presence of a radical intermediate, a radical trapping experiment was carried out under the optimized conditions between **2a** and **1c** with the addition of (2,2,6,6-tetramethylpiperidin-1-yl)oxyl (TEMPO) (1.2 equiv) to the reaction mixture (Scheme 3).

This gave exclusively the TEMPO-coupled product (**3oa**), which supports the hypothesis that the reaction proceeds through a radical intermediate.

**Scheme 4:**
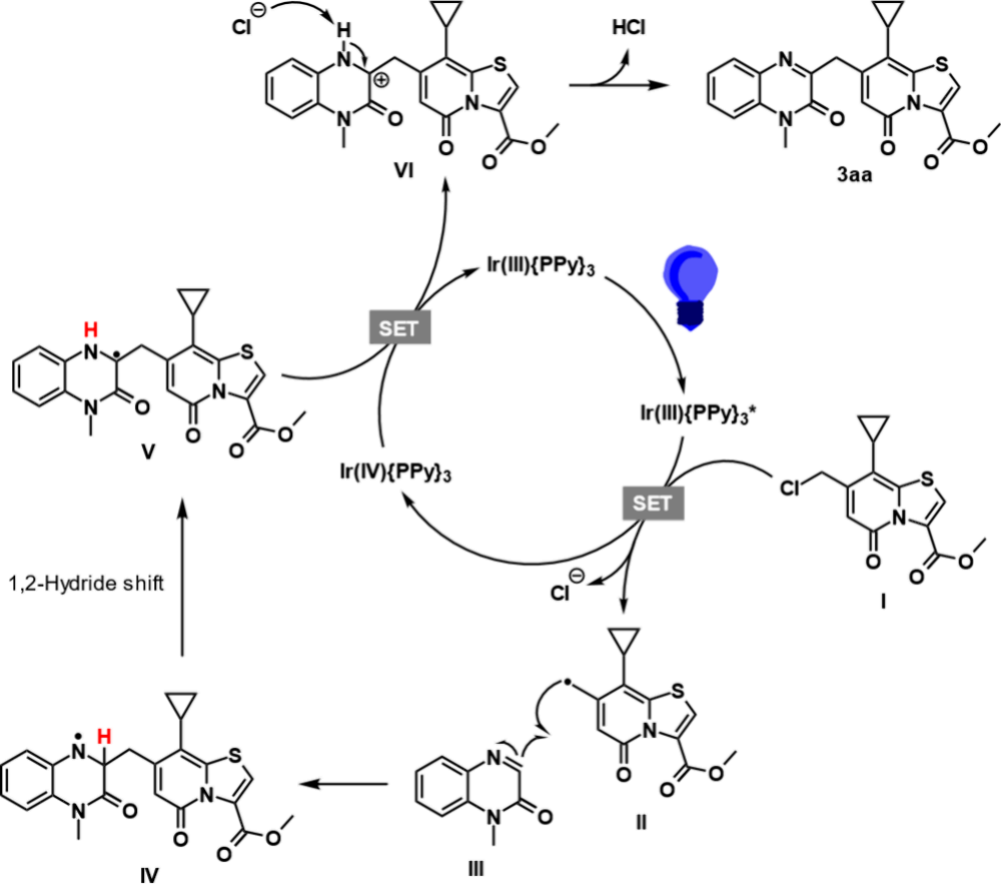
Proposed Mechanism

Based on the combined results from the mechanistic investigation, the radical trapping experiment and reported mechanisms of radical couplings with quinoxalinones in the literature,^12−17^ we propose the following mechanism for the transformation (Scheme 4).

The photocatalyst is excited by the 455 nm light and undergoes oxidative quenching with the C7-methyl chloride TRP (**I**), generating a benzyl-type radical (**II**) and a chloride ion. The TRP radical then undergoes radical addition to the imine carbon of the quinoxalinone (**III**), resulting in a N-centered radical (**IV**). This radical species can then undergo a 1,2-hydride shift to a more stable tertiary C-centered radical (**V**).^15,16^ The photocatalyst is then regenerated through SET from **V** to the catalyst, generating a positively charged TRP–quinoxalinone-coupled species (**VI**) that gives the final product **3aa** after deprotonation.

## Conclusion

We have developed a method to introduce quinoxalinones on the C7 position of substituted TRPs using photoredox catalysis. This method provides a new and fast approach to C7 functionalization of TRPs and has provided insight into the photochemical properties of C7-chloromethyl-substituted TRPs. With this, a new tool for C7 functionalization has been discovered which can be used in the future for incorporation of other unexplored radical acceptors in the pursuit of more potent and drug-like TRP-based drug candidates.

## Experimental Section

### General Information and Data Collection

All reagents and solvents were used as received from commercial suppliers without further purification. All of the necessary reactions were carried out in dry solvents under a nitrogen atmosphere. Reaction progress was monitored on aluminum-based silica gel TLC plates (median pore size 60 Å, fluorescent indicator 254 nm) and detected with UV light at 254 and 366 nm. Automated flash column chromatography was performed using a Biotage Isolera One system and purchased prepacked silica gel cartridges (BiotageSfar, duo 60 μm). ^1^H, ^13^C, and ^19^F NMR spectra were recorded on a Bruker AVANCE III 400 MHz spectrometer (101 MHz ^13^C, 376 MHz ^19^F) with a BBO-F/H Smart probe at 298 K unless otherwise stated. All spectrometers were operated by Topspin 3.5.7. Structural assignments were made with additional information from gHSQC and gHMBC experiments. LC-MS was conducted on a Micromass ZQ mass spectrometer using ES+ ionization. HRMS was performed on an Agilent mass spectrometer with ESI-TOF (ES+). FTIR spectra were acquired by pressing the solid sample onto the diamond window of an attenuated total reflectance (ATR) cell (Golden Gate, a single bound diamond window) and then measuring the spectrum with a resolution of 4 cm^–1^ over the 600–4500 cm^–1^ range at a forward/reverse scanning rate of 10 kHz on a Bruker Vertex 70/V instrument. Absorption spectra were acquired in solution using 10 mm path length quartz high-precision cell cuvettes and a UV–vis spectrophotometer (UV5, Mettler Toledo) with background subtraction for the solvent (MeCN). Excitation and emission spectra were recorded in solution using 3 mm path length quartz high precision cell cuvettes with a HORIBA Jobin Yvon spectrofluorometer (FluoroMax-3). Cyclic voltammetry was performed in a 30 mL glass cell using a using a modulab potentiostat (Solartron Analytical, AMETEK). The working electrode was a glassy carbon electrode, polished using a cotton polishing cloth with slurries of progressively finer alumina particles (1, 0.3, and 0.05 μm) purchased from BUEHLER, IL. The quasi-reference electrode was a silver wire, and the counter electrode was a Pt sheet.

### Synthesis of Quinoxalinones (**1a–1m**).^17,18^

The di-, mono-, or nonsubstituted phenylene-1,2-diamine (1 equiv) was added to an appropriately sized round-bottom flask followed by absolute ethanol (2.3 mL/mmol). Ethyl glyoxalate (1.2 equiv) was then added as a 50% (w/w) solution in toluene, and the resulting mixture was stirred at 90 °C in an oil bath for 1 h and then at room temperature overnight. The mixture was then cooled in ice water, and the resulting solid was filtered off using vacuum filtration. The solid was washed with ice cold absolute ethanol on the filter until the liquid passing through was colorless. After extensive drying on the filter, the solid was transferred to a round-bottom flask and further dried at 50 °C under reduced pressure for 2 h. The crude material was used, without further purification, in either methylation or directly in the coupling reaction.

### Methylation of Quinoxalinones

The dried, crude, quinoxalinone (1 equiv) was mixed with K_2_CO_3_ (1.2 equiv) and suspended in DMF (4.6 mL/mmol) before MeI (1.6 equiv) was added. The flask was then capped with a septum, and the mixture was stirred at room temperature and monitored by TLC (80% EtOAc in heptane). Upon completion, the mixture was diluted with deionized water (4.6 mL/mmol) and transferred to a separatory funnel using EtOAc (23 mL/mmol). The aqueous phase was extracted with 2 × EtOAc (11 mL/mmol), and the combined organic phases were washed once with sat. NH_4_Cl_(aq)_ (7 mL/mmol) and then once with brine (10 mL/mmol). The washed organic phase was then dried over Na_2_SO_4_ and filtered into a round-bottom flask before the solvent was removed using rotary evaporation.

#### 1-Methylquinoxalin-2(1*H*)-one (**1a**)

Compound **1a** was dissolved in DCM, purified with automated flash column chromatography (gradient, 0% → 100% EtOAc in heptane), and obtained as an off-white solid (2.13 g, 13.28 mmol, 69%). ^1^H NMR (400 MHz, CDCl_3_): δ 8.31 (s, 1H), 7.88 (dd, *J* = 7.9, 1.5 Hz, 1H), 7.60 (ddd, *J* = 8.6, 7.4, 1.6 Hz, 1H), 7.41–7.31 (m, 2H), 3.70 (s, 3H). ^13^C{^1^H} NMR (101 MHz, CDCl_3_): δ 155.2, 150.4, 133.5, 133.4, 131.2, 130.7, 123.9, 113.9, 28.9.

#### 1,5-Dimethylquinoxalin-2(1*H*)-one (**1b**)

Compound **1b** was dissolved in DCM, purified with automated flash column chromatography (gradient, 0% → 70% EtOAc in heptane), and obtained as a beige solid (82 mg, 0.473 mmol, 5%). ^1^H NMR (400 MHz, CDCl_3_): δ 8.30 (s, 1H), 7.47 (t, *J* = 7.9 Hz, 1H), 7.23–7.19 (m, 2H), 7.17 (d, *J* = 8.5 Hz, 1H), 3.68 (s, 3H), 2.67 (s, 3H). ^13^C{^1^H} NMR (101 MHz, CDCl_3_): δ 155.1, 148.5, 139.4, 133.5, 132.2, 130.9, 125.2, 111.8, 29.0, 17.7.

#### 1,6,7-Trimethylquinoxalin-2(1*H*)-one (**1c**)

Compound **1c** was dissolved in DCM, purified with automated flash column chromatography (gradient, 60% → 70% EtOAc in heptane), and obtained as a beige/pink solid (711 mg, 3.779 mmol, 81%). ^1^H NMR (400 MHz, CDCl_3_): δ 8.22 (s, 1H), 7.61 (s, 1H), 7.09 (s, 1H), 3.66 (s, 3H), 2.42 (s, 3H), 2.35 (s, 3H). ^13^C{^1^H} NMR (101 MHz, CDCl_3_): δ 155.3, 149.1, 141.1, 132.8, 132.0, 131.4, 130.6, 114.5, 28.8, 20.8, 19.3.

#### 6,7-Dimethylquinoxalin-2(1*H*)-one (**1d**)

Compound **1d** did not require additional purification and was obtained as a beige solid (120 mg, 0.689 mmol, 76%). ^1^H NMR (400 MHz, (CD_3_)_2_SO): δ 12.29 (s, 1H), 8.06 (s, 1H), 7.55 (s, 1H), 7.06 (s, 1H), 2.31 (s, 3H), 2.28 (s, 3H). ^13^C{^1^H} NMR (101 MHz, (CD_3_)_2_SO): δ 155.0, 150.3, 140.4, 131.9, 130.6, 129.8, 128.6, 115.7, 19.8, 18.8.

#### 5-Fluoro-1-methyl-2(1*H*)-quinoxalinone (**1e**)

Compound **1e** was dissolved in DCM, purified with automated flash column chromatography (gradient, 0% → 70% EtOAc in heptane), and obtained as a beige solid (173 mg, 0.973 mmol, 24%). ^1^H NMR (400 MHz, CDCl_3_): δ 8.32 (s, 1H), 7.55 (td, *J* = 8.4, 5.7 Hz, 1H), 7.16–7.06 (m, 2H). ^13^C{^1^H} NMR (101 MHz, CDCl_3_): δ 160.3, 157.7, 155.0, 150.2 (d, *J* = 1.8 Hz), 135.1 (d, *J* = 2.5 Hz), 131.8 (d, *J* = 9.9 Hz), 123.4 (d, *J* = 13.9 Hz), 110.3 (d, *J* = 19.5 Hz), 109.6 (d, *J* = 4.2 Hz), 29.3. ^19^F{^1^H} NMR (376 MHz, CDCl_3_): δ −122.0.

#### 5-Bromo-*N*-methylquinoxalin-2(1*H*)-one (**1f**)

Compound **1f** was dissolved in DCM, purified with automated flash column chromatography (gradient, 40% → 60% EtOAc in heptane), and obtained as a beige/brown solid (280 mg, 1.173 mmol, 36%). ^1^H NMR (400 MHz, CDCl_3_): δ 8.37 (s, 1H), 7.64 (dd, *J* = 7.9, 1.2 Hz, 1H), 7.46–7.40 (m, 1H), 7.30 (dd, *J* = 8.5, 1.2 Hz, 1H), 3.68 (s, 3H). ^13^C{^1^H} NMR (101 MHz, CDCl_3_): δ 154.7, 150.8, 134.9, 131.6, 131.1, 128.1, 126.3, 113.7, 29.3.

#### 8-Bromo-1-methylquinoxalin-2(1*H*)-one (**1g**)

Compound **1g** was dissolved in DCM, purified with automated flash column chromatography (gradient, 40% → 60% EtOAc in heptane), and obtained as a beige/brown solid (29 mg, 0.122 mmol, 3%). ^1^H NMR (400 MHz, CDCl_3_): δ 8.29 (s, 1H), 7.83 (q, *J* = 1.6 Hz, 1H), 7.81 (q, *J* = 1.6 Hz, 1H), 7.19 (t, *J* = 7.9 Hz, 1H), 4.04 (s, 3H). ^13^C{^1^H} NMR (101 MHz, CDCl_3_): δ 156.4, 150.4, 138.2, 136.0, 133.3, 130.8, 124.8, 107.2, 36.0.

#### 7-Bromo-1-methylquinoxalin-2(1*H*)-one (**1h**)

Compound **1h** was dissolved in DCM, purified with automated flash column chromatography (gradient, 0% → 70% EtOAc in heptane), and obtained as a beige/brown solid (108 mg, 0.452 mmol, 6%). ^1^H NMR (400 MHz, CDCl_3_): δ 8.29 (s, 1H), 7.73 (d, *J* = 8.4 Hz, 1H), 7.50 (d, *J* = 2.0, 1H), 7.47 (dd, *J* = 8.4, 2.0 Hz, 1H), 3.66 (s, 3H). ^13^C{^1^H} NMR (101 MHz, CDCl_3_): δ 154.8, 150.5, 134.4, 132.4, 131.9, 127.2, 125.5, 117.1, 29.0.

#### 6,7-Dichloro-1-methylquinoxalin-2(1*H*)-one (**1i**)

Compound **1i** was dissolved in DCM, purified with automated flash column chromatography (gradient, 0% → 100% EtOAc in heptane), and obtained as a beige solid (376 mg, 1.645 mmol, 27%). ^1^H NMR (400 MHz, CDCl_3_): δ 8.28 (s, 1H), 7.97 (s, 1H), 7.44 (s, 1H), 3.65 (s, 3H). ^13^C{^1^H} NMR (101 MHz, CDCl_3_): δ 154.5, 151.6, 135.5, 132.8, 132.5, 131.4, 127.8, 115.5, 29.2.

#### 1-Methyl-6-(trifluoromethyl)-2(1*H*)-quinoxalinone (**1j**)

Compound **1j** was dissolved in DCM, purified with automated flash column chromatography (gradient, 0% → 70% EtOAc in heptane), and obtained as a yellow solid (106 mg, 0.465 mmol, 6%). ^1^H NMR (400 MHz, CDCl_3_): δ 8.37 (s, 1H), 8.18–8.15 (m, 1H), 7.82 (dd, *J* = 8.8, 2.1 Hz, 1H), 7.45 (d, *J* = 8.8 Hz, 1H), 3.72 (s, 3H). ^13^C{^1^H} NMR (101 MHz, CDCl_3_): δ 154.9, 151.8, 135.8, 132.9, 128.1 (q, *J* = 3.9 Hz), 127.7–127.4 (m), 126.8–125.7 (m), 125.4–121.9 (m), 114.7, 29.1. ^19^F{^1^H} NMR (376 MHz, CDCl_3_): δ −62.1.

#### 6,7-Difluoroquinoxalin-2(1*H*)-one (**1k**)

Compound **1k** was obtained as a purple solid and required no further purification (143 mg, 0.785 mmol, 91%). ^1^H NMR (400 MHz, (CD_3_)_2_SO): δ 12.53 (s, 1H), 8.18 (s, 1H), 8.02–7.85 (m, 1H), 7.32–7.17 (m, 1H). ^13^C{^1^H} NMR (101 MHz, (CD_3_)_2_SO): δ 154.5, 152.3, 150.7 (dd, *J* = 250.6, 14.8 Hz), 145.6 (dd, *J* = 242.6, 14.0 Hz), 129.4 (d, *J* = 10.2 Hz), 128.3 (d, *J* = 9.6 Hz), 116.92–115.56 (m), 103.5 (d, *J* = 21.8 Hz). ^19^F{^1^H} NMR (376 MHz, (CD_3_)_2_SO): δ −132.44 (d, *J* = 23.4 Hz), −143.46 (d, *J* = 23.2 Hz).

#### 1-Methyl-7-nitroquinoxalin-2(1*H*)-one (**1l**)

Compound **1l** was dissolved in DCM, purified with automated flash column chromatography (40% EtOAc in heptane), and obtained as a red solid (144 mg, 0.706 mmol, 10%). ^1^H NMR (400 MHz, CDCl_3_): δ 8.43 (s, 1H), 8.24 (d, *J* = 2.3 Hz, 1H), 8.19 (dd, *J* = 8.7, 2.3 Hz, 1H), 8.04 (d, *J* = 8.7 Hz, 1H), 3.76 (s, 3H).

#### 4-Methyl-3-oxo-3,4-dihydroquinoxaline-6-carbonitrile (**1m**)

Compound **1m** was dissolved in DCM, purified with automated flash column chromatography (gradient, 0 → 100% EtOAc in heptane), and obtained as a beige solid (66 mg, 0.359 mmol, 4%). ^1^H NMR (400 MHz, CDCl_3_): δ 8.39 (s, 1H), 7.98 (d, *J* = 8.1 Hz, 1H), 7.63 (d, *J* = 1.6 Hz, 1H), 7.61 (dd, *J* = 8.1, 1.6 Hz, 1H), 3.70 (s, 3H). ^13^C{^1^H} NMR (101 MHz, CDCl_3_): δ 154.5, 153.3, 135.5, 133.9, 131.7, 126.8, 118.1, 118.0, 114.4, 29.1.

#### Synthesis of 7-Amino-1-methylquinoxalin-2(1*H*)-one (**1n**)^19^

**1l** (105 mg, 0.512 mmol, 1.0 equiv) was added to a 100 mL round-bottom flask followed by B_2_pin_2_ (403 mg, 1.589 mmol, 3.1 equiv) and KOtBu (69 mg, 0.616 mmol, 1.2 equiv). iPrOH (4 mL/mmol **1l**) was then added, and the mixture was refluxed for 30 min. The solution was then added to a separatory funnel followed by 20 mL of EtOAc and 20 mL of brine. The aqueous phase was extracted with EtOAc (2 × 20 mL). The pH was adjusted to 12 using 1 M NaOH_(aq)_ and then extracted once again using 20 mL of EtOAc. The combined organic phases were dried over Na_2_SO_4_ and filtered into a 250 mL round-bottom flask. The solvent was removed using rotary evaporation. The resulting solid was dissolved in acetone, evaporated onto Celite before purification using flash column chromatography (gradient, 80 → 100% EtOAc in heptane), and obtained as a yellow solid (37 mg, 0.211, 41%). ^1^H NMR (400 MHz, CD_3_OD): δ 7.80 (s, 1H), 7.51 (d, *J* = 8.7 Hz, 1H), 6.73 (dd, *J* = 8.7, 2.2 Hz, 1H), 6.63 (d, *J* = 2.2 Hz, 1H), 3.62 (s, 3H). −NH_2_ protons were not observed. Other peaks were consistent with the literature.^20^

#### Methyl 7-(Chloromethyl)-8-cyclopropyl-5-oxo-5*H*-thiazolo[3,2-*a*]pyridine-3-carboxylate (**2a**)

**2a** was prepared according to the published protocol.^21^^1^H and ^13^C NMR spectra were consistent with the literature.

#### Synthesis of Methyl 7-(Chloromethyl)-5-oxo-8-(3-(trifluoromethyl)phenyl)-5*H*-thiazolo[3,2-*a*]pyridine-3-carboxylate (**2b**)

(±)-Methyl 7-(chloromethyl)-5-oxo-8-(3-(trifluoromethyl)phenyl)-2,3-dihydro-5*H*-thiazolo[3,2-*a*]pyridine-3-carboxylate (**2e**, 776 mg, 1.92 mmol, 1.0 equiv) was added to a 50 mL round-bottom flask followed by DCM (7 mL). The solution was cooled to 0 °C, and 70% *m*-CPBA (905 mg, 3.67 mmol, 1.9 equiv) was added in portions while stirring. The solution was then stirred at room temperature and monitored by TLC (75% EtOAc in heptane). After 3 h, all of the starting material had been consumed. The solution was diluted with DCM and purified with automated flash column chromatography (gradient, 60% → 90% EtOAc in heptane) to remove residual benzoic acid. The collected fractions were evaporated into a 100 mL round-bottom flask and dissolved in toluene (5 mL). TFAA (0.8 mL, 5.75 mmol, 5.0 equiv) was then added, and the mixture was heated to 60 °C and monitored by TLC (75% EtOAc in heptane). All starting material was consumed after 4 h. The solvent was then removed by coevaporation with DCM on a rotary evaporator, giving a yellow solid. The solid was dissolved in DCM (5 mL) and added dropwise to conc. H_2_SO_4_ (1 mL) at 0 °C. The flask was washed twice with DCM (2.5 mL, then 1 mL), and the washings were added dropwise to the conc. H_2_SO_4_. The biphasic mixture was stirred at 0 °C for 1 h. The reaction was then quenched with sat. NaHCO_3(aq)_ (100 mL), and the aqueous solution was extracted with DCM (3 × 15 mL). The combined organic phases were dried over Na_2_SO_4_, filtered, and evaporated using rotary evaporation, giving a light-yellow amorphous solid (410 mg, 1.021 mmol, 53%) requiring no additional purification. ^1^H NMR (400 MHz, CDCl_3_): δ 7.79–7.72 (m, 1H), 7.70–7.65 (m, 2H), 7.62–7.58 (m, 1H), 7.11 (s, 1H), 6.59 (s, 1H), 4.22 (s, 2H), 4.00 (s, 3H). ^13^C{^1^H} NMR (101 MHz, CDCl_3_): δ 160.5, 158.9, 148.5, 146.8, 135.4, 133.7, 132.5–132.0 (m), 132.1, 130.4, 127.1 (q, *J* = 3.7 Hz), 126.4–126.1 (m), 125.4–122.2 (m), 114.7, 112.5, 111.9, 53.7, 42.5. ^19^F{^1^H} NMR (376 MHz, CDCl3): δ −62.7. HRMS (ESI-TOF) *m*/*z* [M + Na]^+^ calcd for C_17_H_11_ClF_3_NNaO_3_S, 423.9992; found, 423.9981. ATR-FTIR (neat) cm^–1^: 1734 (m), 1658 (m), 1561 (w), 1473 (m), 1341 (m), 1273 (m), 1258 (m), 1231 (w), 1165 (m), 1123 (s), 1077 (m), 1030 (m), 847 (w), 806 (w), 784 (w), 704 (m).

#### Synthesis of Methyl 7-(Chloromethyl)-5-oxo-5*H*-thiazolo[3,2-*a*]pyridine-3-carboxylate (**2c**)

(±)-Methyl 7-(chloromethyl)-5-oxo-2,3-dihydro-5*H*-thiazolo[3,2-*a*]pyridine-3-carboxylate (**2g**, 309 mg, 1.19 mmol, 1.0 equiv) was added to a 50 mL round-bottom flask followed by DCM (8 mL). The solution was cooled to 0 °C, and 70% *m*-CPBA (514 mg, 2.08 mmol, 1.75 equiv) was added in portions while stirring. The solution was stirred for 5 min, brought to room temperature, and monitored by TLC (80% EtOAc in heptane). After 3 h, all of the starting material had been consumed. The solution was diluted with DCM and purified with automated flash column chromatography (gradient, 0% → 100% EtOAc in heptane) to remove residual benzoic acid. The collected fractions were evaporated into a 100 mL round-bottom flask and dissolved in toluene (5 mL). TFAA (0.33 mL, 2.38 mmol, 5.0 equiv) was then added, and the solution was stirred at 60 °C and monitored by TLC (80% EtOAc in heptane). All of the starting material was consumed after 4 h. The solvent was then removed by coevaporation with DCM on a rotary evaporator, giving a yellow semisolid. The mixture was transferred to a separatory funnel using DCM (10 mL) and washed with sat. NaHCO_3(aq)_ (10 mL). The aqueous phase was extracted with DCM (3 × 10 mL), and the combined organic phases were dried over Na_2_SO_4_, filtered, and evaporated using rotary evaporation. The crude was then purified with automated flash column chromatography (gradient, 0 → 100% EtOAc in heptane), giving **2c** as a yellow amorphous solid (113 mg, 0.441 mmol, 37%). ^1^H NMR (400 MHz, CDCl_3_): δ 7.14 (s, 1H), 6.76 (d, *J* = 1.5 Hz, 1H), 6.39 (dd, *J* = 1.6 Hz, 0.8, 1H), 4.45 (d, *J* = 0.7 Hz, 2H), 3.98 (s, 3H). ^13^C{^1^H} NMR (101 MHz, CDCl_3_): δ 160.7, 159.3, 148.8, 147.8, 131.3, 114.2, 109.8, 99.7, 53.7, 44.4. HRMS (ESI-TOF) *m*/*z* [M + Na]^+^ calcd for C_10_H_8_ClNNaO_3_S, 279,9806; found, 279.9783. ATR-FTIR (neat) cm^–1^: 3049 (w), 2923 (w), 1731 (s), 1664 (s), 1561 (m), 1495 (s), 1426 (m), 1325 (m), 1240 (s), 1202 (s), 1158 (m), 1126 (m), 1032 (s), 1014 (m), 931 (m), 831 (m), 782 (s), 761 (s), 739 (s), 675 (m), 610 (m).

#### Synthesis of Methyl 7-(Chloromethyl)-8- methoxy-5-oxo-5*H*-thiazolo[3,2-*a*]pyridine-3-carboxylate (**2d**)

(±)-Methyl 7-(chloromethyl)-8-methoxy-5-oxo-2,3-dihydro-5*H*-thiazolo[3,2-*a*]pyridine-3-carboxylate (634 mg, 2.19 mmol, 1.0 equiv) was added to a 15 mL round-bottom flask followed by DCM (7 mL). The solution was cooled to 0 °C, and 70% *m*-CPBA (1.1 g, 4.46 mmol, 2.0 equiv) was added in portions while stirring. The solution was then stirred at room temperature and monitored by TLC (90% EtOAc in heptane). All of the starting material had been consumed after 1.5 h. The reaction was then quenched with sat. NaHCO_3(aq)_ (25 mL) and transferred to a separatory funnel using DCM (15 mL). The aqueous phase was then extracted with 3 × 15 mL of DCM. The organic phases were then dried over Na_2_SO_4_ and filtered into a 250 mL round-bottom flask. The crude was diluted with DCM and purified with automated flash column chromatography (gradient, 0 → 100% EtOAc in heptane) to remove residual benzoic acid. The collected fractions were evaporated into a 50 mL round-bottom flask and dissolved in toluene (5 mL). TFAA (0.82 mL, 5.90 mmol, 5.0 equiv) was then added, and the mixture was heated to 110 °C and monitored by TLC (90% EtOAc in heptane). All of the starting material was consumed after 20 min. The solvent was then removed by coevaporation with DCM using rotary evaporation, giving a brown liquid. The liquid was diluted with DCM (5 mL) and added dropwise to conc. H_2_SO_4_ (100 μL) at 0 °C. The flask was washed once with DCM (2 mL), and the washings were added dropwise to the conc. H_2_SO_4_. The biphasic mixture was stirred at 0 °C for 30 min and then at rt for 2 h. The reaction was then quenched with sat. NaHCO_3(aq)_ (25 mL). The mixture was transferred to a separatory funnel using DCM (15 mL) and brine (25 mL). The aqueous phase was then extracted with DCM (3 × 15 mL). The combined organic phases were dried over Na_2_SO_4_, filtered, and evaporated using rotary evaporation, giving a brown solid. The solid was dissolved in DCM and purified with automated flash column chromatography (gradient, 20% → 50% EtOAc in heptane), and **2d** was obtained as a yellow/orange paste (39 mg, 0.136 mmol, 6%). ^1^H NMR (400 MHz, CDCl_3_): δ 7.15 (s, 1H), 6.42 (s, 1H), 4.52 (s, 2H), 3.98 (s, 3H), 3.90 (s, 3H). ^13^C{^1^H} NMR (101 MHz, CDCl_3_): δ 160.6, 158.0, 143.8, 139.9, 135.3, 132.2, 114.6, 111.2, 61.2, 53.7, 39.4. HRMS (ESI-TOF) *m*/*z* [M + Na]^+^ calcd for C_11_H_10_ClNNaO_4_S, 309.9911; found, 309.9894. ATR-FTIR (neat) cm^–1^: 2952 (w), 1736 (m), 1657 (s), 1559 (m), 1480 (s), 1454 (m), 1339 (w), 1248 (s), 1158 (m), 1123 (m), 1041 (m), 994 (s), 946 (w), 839 (m), 747 (s), 673 (m).

#### Methyl 7-(Chloromethyl)-5-oxo-8-(3-(trifluoromethyl)phenyl)-2,3-dihydro-5*H*-thiazolo[3,2-*a*]pyridine-3-carboxylate (**2e**)

**2e** was prepared according to the published protocol.^22^^1^H and ^13^C NMR spectra were consistent with the literature.

#### Methyl 7-(Chloromethyl)-8-cyclopropyl-5-oxo-2,3-dihydro-5*H*-thiazolo[3,2-*a*]pyridine-3-carboxylate (**2f**)

**2f** was prepared according to the published protocol.^23^^1^H and ^13^C NMR spectra were consistent with the literature.

#### Methyl 7-(Chloromethyl)-5-oxo-2,3-dihydro-5*H*-thiazolo[3,2-*a*]pyridine-3-carboxylate (**2g**)

**2g** was prepared according to the published protocol.^23^^1^H and ^13^C NMR spectra were consistent with the literature.

### Synthesis of Quinoxalinone-Coupled Pyridones (**3aa–3oa**)

Quinoxalinone **1a**–**1n** (1.2 equiv), C7-methyl chloride-pyridone **2a**–**2g** (1.0 equiv), and Ir(PPy)_3_ (2 mol %) were added to an oven-dried photoreaction vial which was then sealed with a Biotage septum cap for microwave reaction vials. The vial was then evacuated and backfilled with N_2(g)_ 3 times on a Schlenk line. Dry MeCN (9.4 mL/mmol pyridone) was then added, and the mixture was degassed by purging with N_2(g)_ for 5 min. The mixture was then stirred at 22 °C with water cooling while irradiating with 455 nm LEDs with an input power of 10 W and output luminous flux of 130 lm for 30 min to 1 h (for setup see Supporting Information Figures S.1–S.5). Reactions were monitored with TLC (80% EtOAc in heptane). When all of the TRP was consumed, the solution was transferred to a separatory funnel using EtOAc (10 mL) followed by deionized water (10 mL) and brine (2 mL). The aqueous phase was extracted with EtOAc (3 × 10 mL), and the combined organic phases were dried over Na_2_SO_4_ and filtered into a 100 mL round-bottom flask. The solvent was then removed using rotary evaporation.

#### Methyl 8-Cyclopropyl-7-((4-methyl-3-oxo-3,4-dihydroquinoxalin-2-yl)methyl)-5-oxo-5*H*-thiazolo[3,2-*a*]pyridine-3-carboxylate (**3aa**)

Compound **3aa** was dissolved in DCM, purified with automated flash column chromatography (gradient, 0% → 100% EtOAc in heptane), and obtained as a yellow/orange amorphous solid (39 mg, 0.093 mmol, 57%). ^1^H NMR (400 MHz, CDCl_3_): δ 7.80 (dd, *J* = 8.0, 1.5 Hz, 1H), 7.55 (ddd, *J* = 8.6, 7.3, 1.5 Hz, 1H), 7.36–7.32 (m, 1H), 7.31 (dd, *J* = 7.7, 1.1 Hz, 1H), 7.08 (s, 1H), 6.15 (s, 1H), 4.41 (br s, 2H), 3.91 (s, 3H), 3.67 (s, 3H), 1.99–1.89 (m, 1H), 1.10–1.00 (m, 2H), 0.83–0.74 (m, 2H). ^13^C{^1^H} NMR (101 MHz, CDCl_3_): δ 161.22, 159.0, 158.0, 154.6, 151.1, 147.7, 133.4, 132.7, 131.6, 130.4, 130.1, 123.9, 114.2, 113.8, 113.2, 110.9, 53.4, 37.5, 29.3, 11.1, 7.9. HRMS (ESI-TOF) *m*/*z* [M + H]^+^ calcd for C_22_H_20_N_3_O_4_S, 422.1169; found, 422.1167. ATR-FTIR (neat) cm^–1^: 1737 (m), 1645 (s), 1601 (m), 1563 (w), 1467 (s), 1434 (w), 1332 (w), 1244 (m), 1227 (m), 1179 (w), 1125 (w), 1096 (w), 1039 (w), 954 (w), 909 (w), 746 (s), 646 (w).

#### Methyl 8-Cyclopropyl-7-((4,8-dimethyl-3-oxo-3,4-dihydroquinoxalin-2-yl)methyl)-5-oxo-5*H*-thiazolo[3,2-*a*]pyridine-3-carboxylate (**3ba**)

Compound **3ba** was dissolved in DCM, purified with automated flash column chromatography (gradient, 0% → 100% EtOAc in heptane), and obtained as a light-yellow amorphous solid (41 mg, 0.095 mmol, 57%). ^1^H NMR (400 MHz, CDCl_3_): δ 7.41 (dd, *J* = 8.4, 7.4 Hz, 1H), 7.18 (d, *J* = 7.3 Hz, 1H), 7.13 (d, *J* = 8.4 Hz, 1H), 7.08 (s, 1H), 6.19 (s, 1H), 4.42 (s, 2H), 3.91 (s, 3H), 3.66 (s, 3H), 2.58 (s, 3H), 2.00–1.83 (m, 1H), 1.12–0.99 (m, 2H), 0.82–0.74 (m, 2H). ^13^C{^1^H} NMR (101 MHz, CDCl_3_): δ 161.2, 159.0, 156.0, 154.5, 151.4, 147.5, 138.9, 133.5, 131.6, 131.3, 130.1, 125.2, 114.2, 113.3, 111.7, 111.0, 53.4, 37.5, 29.4, 17.6, 11.2, 7.9. HRMS (ESI-TOF) *m*/*z* [M + H]^+^ calcd for C_23_H_22_N_3_O_4_S, 436.1326; found, 436.1332. ATR-FTIR (neat) cm^–1^: 1738 (m), 1643 (s), 1597 (m), 1467 (s), 1434 (m), 1364 (w), 1333 (w), 1245 (m), 1176 (w), 1125 (w), 1037 (m), 954 (w), 909 (w), 844 (w), 746 (s), 665 (m).

#### Methyl 8-Cyclopropyl-5-oxo-7-((4,6,7-trimethyl-3-oxo-3,4-dihydroquinoxalin-2-yl)methyl)-5*H*-thiazolo[3,2-*a*]pyridine-3-carboxylate (**3ca**)

Compound **3ca** was dissolved in DCM, purified with automated flash column chromatography (gradient, 0% → 100% EtOAc in heptane), and obtained as a yellow/orange amorphous solid (32 mg, 0.073 mmol, 69%). ^1^H NMR (400 MHz, CDCl_3_): δ 7.56 (s, 1H), 7.07–7.06 (m, 2H), 6.15 (s, 1H), 4.39 (s, 2H), 3.91 (s, 3H), 3.65 (s, 3H), 2.41 (s, 3H), 2.33 (s, 3H), 1.98–1.86 (m, 1H), 1.09–0.98 (m, 2H), 0.83–0.72 (m, 2H). ^13^C{^1^H} NMR (101 MHz, CDCl_3_): δ 161.3, 159.0, 156.6, 154.7, 151.4, 147.5, 140.3, 132.9, 131.7, 131.4, 131.2, 130.2, 114.3, 114.1, 113.2, 111.0, 53.4, 37.5, 29.2, 20.7, 19.3, 11.2, 7.9. HRMS (ESI-TOF) *m*/*z* [M + H]^+^ calcd for C_24_H_24_N_3_O_4_S, 450.1482; found, 450.1480. ATR-FTIR (neat) cm^–1^: 1736 (m), 1648 (s), 1618 (m), 1559 (w), 1467 (s), 1363 (w), 1334 (w), 1243 (m), 1175 (w), 1223 (w), 1039 (w), 844 (w), 820 (w), 747 (m).

#### Methyl 8-Cyclopropyl-7-((6,7-dimethyl-3-oxo-3,4-dihydroquinoxalin-2-yl)methyl)-5-oxo-5*H*-thiazolo[3,2-*a*]pyridine-3-carboxylate (**3da**)

Compound **3da** was dissolved in DCM, purified with automated flash column chromatography (gradient, 0% → 5% MeOH in DCM), and obtained as a yellow/brown amorphous solid (22 mg, 0.052 mmol, 51%). ^1^H NMR (400 MHz, CDCl_3_): δ 12.00 (s, 1H), 7.53 (s, 1H), 7.08 (s, 1H), 7.03 (s, 1H), 6.28 (s, 1H), 4.43 (s, 2H), 3.92 (s, 3H), 2.36 (s, 3H), 2.32 (s, 3H), 1.94–1.85 (m, 1H), 1.11–1.03 (m, 2H), 0.86–0.78 (m, 2H). ^13^C{^1^H} NMR (101 MHz, CDCl_3_): δ 161.3, 159.1, 157.0, 156.2, 151.4, 147.5, 140.5, 133.5, 131.7, 131.4, 129.3, 129.1, 115.9, 114.1, 113.3, 111.7, 53.4, 36.6, 20.3, 19.6, 11.3, 7.9. HRMS (ESI-TOF) *m*/*z* [M + H]^+^ calcd for C_23_H_22_N_3_O_4_S, 436.1326; found, 436.1322. ATR-FTIR (neat) cm^–1^: 1740 (m), 1657 (s), 1558 (w), 1470 (s), 1334 (w), 1246 (m), 1126 (w), 1039 (w), 825 (w), 746 (m).

#### Methyl 8-Cyclopropyl-7-((8-fluoro-4-methyl-3-oxo-3,4-dihydroquinoxalin-2-yl)methyl)-5-oxo-5*H*-thiazolo[3,2-*a*]pyridine-3-carboxylate (**3ea**)

Compound **3ea** was dissolved in DCM, purified with automated flash column chromatography (80% EtOAc in heptane), and obtained as a yellow amorphous solid (40 mg, 0.091 mmol, 54%). ^1^H NMR (400 MHz, CDCl_3_): δ 7.50 (td, *J* = 8.4, 5.6 Hz, 1H), 7.12–7.04 (m, 3H), 6.09 (s, 1H), 4.45 (s, 2H), 3.90 (s, 3H), 3.65 (s, 3H), 2.04–1.94 (m, 1H), 1.17–1.03 (m, 2H), 0.90–0.74 (m, 2H). ^13^C{^1^H} NMR (101 MHz, CDCl_3_): δ 161.2, 158.9, 158.5 (d, *J* = 257.9 Hz), 158.2 (d, *J* = 1.6 Hz), 154.4, 150.7, 147.8, 135.1 (d, *J* = 2.6 Hz), 131.6, 130.9 (d, *J* = 9.8 Hz), 122.5 (d, *J* = 13.6 Hz), 114.3, 113.1, 110.5, 110.3 (d, *J* = 19.6 Hz), 109.5 (d, *J* = 4.1 Hz), 53.4, 37.6, 29.7, 11.1, 7.9. ^19^F{^1^H} NMR (376 MHz, CDCl_3_): δ −122.0. HRMS (ESI-TOF) *m*/*z* [M + H]^+^ calcd for C_22_H_19_FN_3_O_4_S, 440.1075; found, 440.1089. ATR-FTIR (neat) cm^–1^: 1739 (m), 1654 (s), 1614 (m), 1568 (w), 1469 (s), 1434 (w), 1249 (m), 1223 (m), 1038 (m), 795 (m), 739 (w).

#### Methyl 7-((8-Bromo-4-methyl-3-oxo-3,4-dihydroquinoxalin-2-yl)methyl)-8-cyclopropyl-5-oxo-5*H*-thiazolo[3,2-*a*]pyridine-3-carboxylate (**3fa**)

Compound **3fa** was dissolved in DCM, purified with automated flash column chromatography (gradient, 0% → 100% EtOAc in heptane), and obtained as an orange amorphous solid (30 mg, 0.061 mmol, 36%). ^1^H NMR (400 MHz, CDCl_3_): δ 7.63 (dd, *J* = 7.9, 1.2 Hz, 1H), 7.39 (t, *J* = 8.1 Hz, 1H), 7.08 (s, 1H), 6.22 (s, 1H), 4.48 (s, 2H), 3.92 (s, 3H), 3.68 (s, 3H), 2.09–1.95 (m, 1H), 1.16–1.04 (m, 2H), 0.85–0.77 (m, 2H). ^13^C{^1^H} NMR (101 MHz, CDCl_3_): δ 161.2, 159.0, 158.7, 154.3, 150.8, 147.8, 134.8, 131.7, 130.7, 130.4, 128.1, 126.0, 114.3, 113.5, 113.4, 111.1, 53.4, 37.5, 29.7, 11.3, 8.0. HRMS (ESI-TOF) *m*/*z* [M + H]^+^ calcd for C_22_H_19_BrN_3_O_4_S, 500.0274; found, 500.0278. ATR-FTIR (neat) cm^–1^: 1737 (m), 1649 (s), 1589 (m), 1560 (w), 1466 (s), 1245 (m), 1104 (w), 1039 (w), 786 (w), 745 (s).

#### Methyl 7-((5-Bromo-4-methyl-3-oxo-3,4-dihydroquinoxalin-2-yl)methyl)-8-cyclopropyl-5-oxo-5*H*-thiazolo[3,2-*a*]pyridine-3-carboxylate (**3ga**)

Compound **3ga** was dissolved in DCM, purified with automated flash column chromatography (60% EtOAc in heptane), and obtained as an orange amorphous solid (11 mg, 0.022 mmol, 25%). ^1^H NMR (400 MHz, CDCl_3_): δ 7.79 (dd, *J* = 7.9, 1.5 Hz, 1H), 7.74 (dd, *J* = 7.9, 1.6 Hz, 1H), 7.17 (t, *J* = 7.9 Hz, 1H), 7.09 (s, 1H), 6.16 (s, 1H), 4.41 (s, 2H), 4.02 (s, 3H), 3.93 (s, 3H), 1.97–1.86 (m, 1H), 1.10–0.99 (m, 2H), 0.83–0.74 (m, 2H). ^13^C{^1^H} NMR (101 MHz, CDCl_3_): δ 161.2, 159.0, 158.1, 155.8, 150.6, 147.8, 137.4, 135.3, 133.2, 131.7, 130.3, 124.8, 114.2, 113.2, 111.2, 107.0, 53.5, 37.3, 36.5, 11.2, 7.9. HRMS (ESI-TOF) *m*/*z* [M + H]^+^ calcd for C_22_H_19_BrN_3_O_4_S, 500.0274; found, 500.0307. ATR-FTIR (neat) cm^–1^: 2957 (w), 2922 (m), 2852 (w), 1737 (m), 1649 (s), 1601 (w), 1466 (s), 1362 (w), 1332 (w), 1242 (m), 1177 (w), 1124 (w), 1067 (w), 1037 (m), 790 (w), 744 (s).

#### Methyl 7-((6-Bromo-4-methyl-3-oxo-3,4-dihydroquinoxalin-2-yl)methyl)-8-cyclopropyl-5-oxo-5*H*-thiazolo[3,2-*a*]pyridine-3-carboxylate (**3ha**)

Compound **3ha** was dissolved in DCM, purified with automated flash column chromatography (70% EtOAc in heptane), and obtained as a yellow/orange amorphous solid (30 mg, 0.061 mmol, 36%). ^1^H NMR (400 MHz, CDCl_3_): δ 7.65 (d, *J* = 8.4 Hz, 1H), 7.46 (d, *J* = 1.9 Hz, 1H), 7.44 (dd, *J* = 8.4, 2.0 Hz, 1H), 7.09 (s, 1H), 6.14 (s, 1H), 4.38 (s, 2H), 3.92 (s, 3H), 3.64 (s, 3H), 1.97–1.85 (m, 1H), 1.11–0.97 (m, 2H), 0.82–0.69 (m, 2H). ^13^C{^1^H} NMR (101 MHz, CDCl_3_): δ 161.2, 159.0, 158.4, 154.3, 150.7, 147.8, 134.4, 131.7, 131.5, 131.4, 127.2, 124.5, 116.9, 114.3, 113.1, 111.1, 53.4, 37.4, 29.4, 11.1, 7.9. HRMS (ESI-TOF) *m*/*z* [M + H]^+^ calcd for C_22_H_19_BrN_3_O_4_S, 500.0274; found, 500.0276. ATR-FTIR (neat) cm^–1^: 1729 (m), 1653 (s), 1595 (m), 1467 (m), 1249 (w), 1225 (w), 1182 (w), 1041 (w), 995 (w), 836 (m), 748 (s).

#### Methyl 8-Cyclopropyl-7-((6,7-dichloro-4-methyl-3-oxo-3,4-dihydroquinoxalin-2-yl)methyl)-5-oxo-5*H*-thiazolo[3,2-*a*]pyridine-3-carboxylate (**3ia**)

Compound **3ia** was dissolved in DCM, purified with automated flash column chromatography (gradient, 0% → 100% EtOAc in heptane), and obtained as a yellow amorphous solid (12 mg, 0.025 mmol, 15%). ^1^H NMR (400 MHz, CDCl_3_): δ 7.90 (s, 1H), 7.40 (s, 1H), 7.09 (s, 1H), 6.14 (s, 1H), 4.40 (s, 2H), 3.93 (s, 3H), 3.64 (s, 3H), 1.98–1.82 (m, 1H), 1.10–1.00 (m, 2H), 0.80–0.71 (m, 2H). ^13^C{^1^H} NMR (101 MHz, CDCl_3_): δ 161.2, 159.6, 159.0, 154.1, 150.4, 147.9, 134.6, 132.8, 131.7, 131.0, 127.8, 115.3, 114.3, 113.1, 111.2, 53.5, 37.5, 29.6, 11.2, 7.9. HRMS (ESI-TOF) *m*/*z* [M + H]^+^ calcd for C_22_H_18_Cl_2_N_3_O_4_S, 490.0390; found, 490.0399. ATR-FTIR (neat) cm^–1^: 2924 (w), 1737 (m), 1652 (s), 1597 (m), 1467 (s), 1333 (w), 1301 (w), 1245 (m), 1103 (w), 1039 (w), 885 (w), 843 (w), 746 (m).

#### Methyl 8-Cyclopropyl-7-((4-methyl-3-oxo-7-(trifluoromethyl)-3,4-dihydroquinoxalin-2-yl)methyl)-5-oxo-5*H*-thiazolo[3,2-*a*]pyridine-3-carboxylate (**3ja**)

Compound **3ja** was dissolved in DCM, purified with automated flash column chromatography (gradient, 0% → 100% EtOAc in heptane), and obtained as a yellow amorphous solid (26 mg, 0.053 mmol, 31%). ^1^H NMR (400 MHz, CDCl_3_): δ 8.12–8.07 (m, 1H), 7.78 (dd, *J* = 8.7, 2.1 Hz, 1H), 7.41 (d, *J* = 8.7 Hz, 1H), 7.10 (s, 1H), 6.16 (s, 1H), 4.44 (s, 2H), 3.93 (s, 3H), 3.71 (s, 3H), 1.97–1.86 (m, 1H), 1.11–1.02 (m, 2H), 0.83–0.74 (m, 2H). ^13^C{^1^H} NMR (101 MHz, CDCl_3_): δ 161.2, 159.9, 159.0, 154.5, 150.4, 147.9, 135.7, 132.1, 131.8, 127.85–127.5 (m), 126.87–126.7 (m), 126.6–126.0 (m), 125.4–122.1 (m), 114.5, 114.3, 113.1, 111.2, 53.5, 37.4, 29.6, 11.2, 7.9. ^19^F{^1^H} NMR (376 MHz, CDCl_3_): δ −62.0. HRMS (ESI-TOF) *m*/*z* [M + H]^+^ calcd for C_23_H_19_F_3_N_3_O_4_S, 490.1043; found, 490.1054. ATR-FTIR (neat) cm^–1^: 2360 (w), 1736 (m), 1653 (s), 1618 (m), 1559 (w), 1468 (s), 1324 (m), 1239 (m), 1174 (m), 1120 (s), 1039 (w), 820 (m), 747 (m), 662 (w).

#### Methyl 5-Oxo-8-(3-(trifluoromethyl)phenyl)-7-((4,6,7-trimethyl-3-oxo-3,4-dihydroquinoxalin-2-yl)methyl)-5*H*-thiazolo[3,2-*a*]pyridine-3-carboxylate (**3cb**)

Compound **3cb** was dissolved in DCM, purified with automated flash column chromatography (gradient, 0% → 100% EtOAc in heptane), and obtained as a light-orange amorphous solid (61 mg, 0.110 mmol, 54%). ^1^H NMR (400 MHz, CDCl_3_): δ 7.75–7.53 (m, 4H), 7.53 (s, 1H), 7.04–7.02 (m, 2H), 6.32 (s, 1H), 3.97 (s, 2H), 3.96 (s, 3H), 3.58 (s, 3H), 2.40 (s, 3H), 2.33 (s, 3H). ^13^C{^1^H} NMR (101 MHz, CDCl_3_): δ 160.8, 159.0, 156.0, 154.4, 148.9, 147.5, 140.4, 136.8, 133.9, 132.91, 131.89, 131.6, 131.3, 131.1, 130.14, 130.09, 127.5–127.3 (m), 125.8–125.5 (m), 125.3–122.4 (m), 114.33, 114.30, 113.9, 111.5, 53.5, 37.8, 29.1, 20.7, 19.3. ^19^F{^1^H} NMR (376 MHz, CDCl_3_): δ −62.72. HRMS (ESI-TOF) *m*/*z* [M + H]^+^ calcd for C_28_H_23_F_3_N_3_O_4_S, 554.1356; found, 554.1382. ATR-FTIR (neat) cm^–1^: 1739 (w), 1651 (s), 1619 (m), 1468 (s), 1328 (m), 1258 (m), 1165 (m), 1124 (s), 1075 (w), 1032 (w), 849 (w), 806 (w) 730 (m), 649 (w).

#### Methyl 5-Oxo-7-((4,6,7-trimethyl-3-oxo-3,4-dihydroquinoxalin-2-yl)methyl)-5*H*-thiazolo[3,2-*a*]pyridine-3-carboxylate (**3cc**)

Compound **3cc** was dissolved in DCM, purified with automated flash column chromatography (gradient, 60% → 100% EtOAc in heptane), and obtained as a light-yellow amorphous solid (44 mg, 0.107 mmol, 54%). ^1^H NMR (400 MHz, CDCl_3_): δ 7.58 (s, 1H), 7.05 (s, 1H), 7.03 (s, 1H), 6.77 (d, *J* = 1.4 Hz, 1H), 6.41–6.31 (m, 1H), 4.13 (s, 2H), 3.93 (s, 3H), 3.65 (s, 3H), 2.41 (s, 3H), 2.34 (s, 3H). ^13^C{^1^H} NMR (101 MHz, CDCl_3_): δ 161.0, 159.7, 155.9, 154.8, 150.0, 146.7, 140.5, 133.0, 131.5, 131.2, 131.1, 130.3, 114.4, 113.3, 110.8, 101.8, 53.5, 40.4, 29.3, 20.7, 19.3. HRMS (ESI-TOF) *m*/*z* [M + H]^+^ calcd for C_21_H_20_N_3_O_4_S, 410.1169; found, 410.1185. ATR-FTIR (neat) cm^–1^: 1739 (m), 1642 (s), 1619 (s), 1581 (m), 1493 (s), 1333 (w), 1238 (m), 1201 (m), 1033 (w), 846 (w), 784 (m), 732 (w).

#### Methyl 8-Methoxy-5-oxo-7-((4,6,7-trimethyl-3-oxo-3,4-dihydroquinoxalin-2-yl)methyl)-5*H*-thiazolo[3,2-*a*]pyridine-3-carboxylate (**3 cd**)

Compound **3 cd** was dissolved in DCM, purified with automated flash column chromatography (gradient, 0% → 100% EtOAc in heptane), and obtained as an orange semisolid (38 mg, 0.086 mmol, 45%). ^1^H NMR (400 MHz, CDCl_3_): δ 7.56 (s, 1H), 7.08 (s, 1H), 7.07 (br s, 1H), 6.17 (s, 1H), 4.22 (s, 2H), 3.94 (s, 3H), 3.90 (s, 3H), 3.66 (s, 3H), 2.41 (s, 3H), 2.33 (s, 3H). ^13^C{^1^H} NMR (101 MHz, CDCl_3_): δ 160.9, 158.0, 156.0, 154.7, 145.6, 140.4, 139.1, 136.6, 132.9, 132.0, 131.5, 131.2, 130.3, 114.4, 113.9, 110.9, 60.6, 53.5, 34.3, 29.2, 20.7, 19.3. HRMS (ESI-TOF) *m*/*z* [M + Na]^+^ calcd for C_22_H_21_N_3_NaO_5_S, 462.1094; found, 462.1073. ATR-FTIR (neat) cm^–1^: 2950 (w), 1738 (m), 1645 (m), 1618 (m), 1557 (m), 1476 (m), 1339 (w), 1246 (m), 1042 (m), 997 (m), 844 (m), 745 (s), 664 (m).

#### Methyl 5-Oxo-8-(3-(trifluoromethyl)phenyl)-7-((4,6,7-trimethyl-3-oxo-3,4-dihydroquinoxalin-2-yl)methyl)-2,3-dihydro-5*H*-thiazolo[3,2-*a*]pyridine-3-carboxylate (**3ce**)

Compound **3ce** was dissolved in DCM, purified with automated flash column chromatography (gradient, 50% → 100% EtOAc in heptane), and obtained as a yellow amorphous solid (57 mg, 0.103 mmol, 52%). ^1^H NMR (400 MHz, 343 K, (CD_3_)_2_SO): δ 7.67–7.44 (m, 5H), 7.27 (s, 1H), 6.12 (s, 1H), 5.60 (dd, *J* = 9.2, 2.8 Hz, 1H), 3.92–3.82 (m, 3H), 3.76 (s, 3H), 3.55–3.48 (m, 4H), 2.38 (s, 3H), 2.31 (s, 3H). ^13^C{^1^H} NMR (101 MHz, 343 K, (CD_3_)_2_SO): δ 168.2, 159.6, 155.3, 153.2, 150.8, 147.7, 139.4, 137.1, 133.9, 131.7, 130.7, 129.9, 129.4, 129.2–128.9 (m), 128.7, 126.5–126.3 (m), 124.6–124.2 (m), 125.1–121.9 (m), 114.6–114.4 (m), 113.2, 63.1, 52.4, 36.4, 30.8, 28.5, 19.5, 18.1. HRMS (ESI-TOF) *m*/*z* [M + H]^+^ calcd for C_28_H_25_F_3_N_3_O_4_S, 556.1512; found, 556.1507. ATR-FTIR (neat) cm^–1^: 1751 (w), 1652 (s), 1619 (m), 1584 (w), 1483 (m), 1437 (w), 1330 (m), 1211 (w), 1164 (m), 1122 (m), 1074 (w), 705 (w).

#### Methyl 8-Cyclopropyl-5-oxo-7-((4,6,7-trimethyl-3-oxo-3,4-dihydroquinoxalin-2-yl)methyl)-2,3-dihydro-5*H*-thiazolo[3,2-*a*]pyridine-3-carboxylate (**3cf**)

Compound **3cf** was dissolved in DCM, purified with automated flash column chromatography (gradient, 20% → 100% EtOAc in heptane), and obtained as a light-yellow amorphous solid (37 mg, 0.082 mmol, 41%). ^1^H NMR (400 MHz, CDCl_3_): δ 7.57 (s, 1H), 7.07 (s, 1H), 5.98 (s, 1H), 5.54 (dd, *J* = 8.5, 2.4 Hz, 1H), 4.41 (d, *J* = 15.9 Hz, 1H), 4.14 (d, *J* = 15.8 Hz, 1H), 3.77 (s, 3H), 3.66 (s, 3H), 3.65–3.60 (m, 1H), 3.48 (dd, *J* = 11.7, 2.3 Hz, 1H), 2.42 (s, 3H), 2.34 (s, 3H), 1.79–1.67 (m, 1H), 1.00–0.80 (m, 2H), 0.77–0.66 (m, 2H). ^13^C{^1^H} NMR (101 MHz, CDCl_3_): δ 168.9, 161.3, 156.6, 154.7, 154.4, 147.3, 140.3, 132.8, 131.5, 131.2, 130.2, 114.6, 114.4, 62.9, 53.3, 37.4, 31.9, 29.2, 20.7, 19.3, 11.5, 7.8, 7.4. HRMS (ESI-TOF) *m*/*z* [M + H]^+^ calcd for C_24_H_26_N_3_O_4_S, 452.1639; found, 452.1661. ATR-FTIR (neat) cm^–1^: 2922 (w), 2852 (w), 1752 (w), 1649 (s), 1619 (m), 1582 (w), 1488 (m), 1426 (w), 1210 (w), 1174 (w), 1025 (w), 846 (w).

#### Methyl 5-Oxo-7-((4,6,7-trimethyl-3-oxo-3,4-dihydroquinoxalin-2-yl)methyl)-2,3-dihydro-5*H*-thiazolo[3,2-*a*]pyridine-3-carboxylate (**3cg**)

Compound **3cg** was dissolved in DCM, purified with automated flash column chromatography (gradient, 70% → 100% EtOAc in heptane), and obtained as a yellow semisolid (31 mg, 0.077 mmol, 39%). ^1^H NMR (400 MHz, CDCl_3_): δ 7.57 (s, 1H), 7.05 (s, 1H), 6.28–6.21 (m, 2H), 5.50 (dd, *J* = 8.4, 2.3 Hz, 1H), 4.00 (s, 2H), 3.77 (s, 3H), 3.73–3.61 (m, 4H), 3.50 (dd, *J* = 11.7, 2.3 Hz, 1H), 2.40 (s, 3H), 2.33 (s, 3H). ^13^C{^1^H} NMR (101 MHz, CDCl_3_): δ 168.6, 161.9, 155.7, 154.8, 152.1, 146.6, 140.4, 132.9, 131.5, 131.2, 130.2, 114.8, 114.3, 102.9, 62.7, 53.4, 40.0, 32.0, 29.3, 20.7, 19.3. HRMS (ESI-TOF) *m*/*z* [M + H]^+^ calcd for C_21_H_22_N_3_O_4_S, 412.1326; found, 412.1341. ATR-FTIR (neat) cm^–1^: 1751 (w), 1644 (s), 1618 (s), 1578 (m), 1506 (s), 1468 (w), 1309 (w), 1212 (m), 747 (s).

#### Methyl 8-Cyclopropyl-5-oxo-7-(((2,2,6,6-tetramethylpiperidin-1-yl)oxy)methyl)-5*H*-thiazolo[3,2-*a*]pyridine-3-carboxylate (**3oa**)

Compound **3oa** was dissolved in DCM, purified with automated flash column chromatography (gradient, 50% → 80% EtOAc in heptane), and obtained as a bright-yellow amorphous solid (46 mg, 0.111 mmol, 66%). ^1^H NMR (400 MHz, CDCl_3_): δ 7.06 (s, 1H), 6.70 (s, 1H), 4.98 (d, *J* = 1.1 Hz, 2H), 3.97 (s, 3H), 1.76–1.65 (m, 1H), 1.52–1.45 (m, 4H), 1.39–1.30 (m, 1H), 1.20–1.15 (m, 12H), 1.04–0.98 (m, 2H), 0.67–0.60 (m, 2H). ^13^C{^1^H} NMR (101 MHz, CDCl_3_): δ 161.0, 159.2, 151.3, 146.4, 131.3, 113.6, 110.0, 108.2, 74.7, 60.0, 53.1, 39.5, 32.6, 20.2, 16.8, 9.7, 7.2. HRMS (ESI-TOF) *m*/*z* [M + H]^+^ calcd for C_22_H_31_N_2_O_4_S, 419.1999; found, 419.2028. ATR-FTIR (neat) cm^–1^: 2930 (w), 1740 (m), 1654 (s), 1567 (w), 1470 (s), 1334 (w), 1243 (m), 1130 (w), 1039 (m), 845 (w), 749 (m).

## Data Availability

The data underlying this study are available in the published article and its Supporting Information.

## Supporting Information Available

gHSQC, gHMBC, ^1^H, ^13^C, and ^19^F NMR spectra, HRMS spectra, and ATR-FTIR spectra for all new compounds; ^1^H and ^13^C NMR spectra for known compounds; description of setup for photochemical experiments, control experiments, scale-up experiment, Stern–Volmer experiments, and cyclic voltammetry (PDF)
