# Supplementary material for: Photoredox-Catalyzed Radical Coupling of C7-Chloromethyl-Substituted Thiazolino Ring-Fused 2-Pyridones with Quinoxalinones
Source: J Org Chem. 2024 Jul 25;89(16):11802–10. doi: 10.1021/acs.joc.4c01224 (PMC11334187; doi:10.1021/acs.joc.4c01224)
Supplement: Supplementary file 1 — jo4c01224_si_001.pdf [file jo4c01224_si_001.pdf]

Photoredox Catalyzed Radical Coupling of C7-chloromethyl Substituted Thiazolino Ring-Fused 2-Pyridones with Quinoxalinones

Victor Hellgren[a], Pardeep Singh[a]*, Abhilash Kulkarni[b], Niusha Bagheri[b], Jerker Widengren[b], Gopinathan Manavalan[a], Fredrik Almqvist[a,c]*.

[a]Umeå University, Department of Chemistry, SE-90187 Umeå, Sweden.

[b]Royal Institute of Technology (KTH), Department of Applied Physics, SE-10691 Stockholm, Sweden.

[c]Umeå Centre for Microbial Research, UCMR, Umeå University, SE-90187 Umeå, Sweden.

*Corresponding authors. E-mail: Fredrik.almqvist@umu.se and Pardeep.singh@umu.se

Contents

A General Information and Data Collection **S2**

B Description of Setup for Photochemical Experiments **S2**

C Control Experiments **S5**

D Scaled up Synthesis of 3aa **S6**

E Stern-Volmer Experiments **S6**

F Cyclic Voltammetry **S9**

G Copies of ^{1}H, ^{13}C, ^{19}F, gHSQC, and gHMBC spectra **S10**

H Copies of ATR-FTIR spectra **S139**

I Copies of Mass spectra **S149**

References **S156**

A General Information and Data Collection

All reagents and solvents were used as received from commercial suppliers without further purification. All the necessary reactions were carried out in dry solvents under a nitrogen atmosphere. Reaction progress was monitored on aluminum-based silica gel TLC plates (median pore size 60 Å, fluorescent indicator 254 nm) and detected with UV light at 254 and 366 nm. Automated flash column chromatography was performed using a Biotage Isolera One system and purchased prepacked silica gel cartridges (BiotageSfar, duo 60 μm). ^{1}H, ^{13}C, and ^{19}F NMR spectra were recorded on a Bruker AVANCE III 400 MHz spectrometer (101 MHz ^{13}C, 376 MHz ^{19}F) with a BBO-F/H Smart probe at 298 K unless otherwise stated. All spectrometers were operated by Topspin 3.5.7. Structural assignments were made with additional information from gHSQC and gHMBC experiments. LC-MS was conducted on a Micromass ZQ mass spectrometer using ES+ ionization. HRMS was performed on an Agilent mass spectrometer with ESI-TOF (ES+). FTIR spectra were acquired by pressing the solid sample onto the diamond window of an Attenuated Total Reflectance (ATR) cell (Golden Gate, a single bound diamond window) and then measuring the spectrum with a resolution of 4 cm^{-1} over the 600–4500 cm^{-1} range at a forward/reverse scanning rate of 10 kHz on a Bruker Vertex 70/V instrument. Absorption spectra were acquired in solution using 10 mm path length quartz high precision cell cuvettes and a UV/VIS spectrophotometer (UV5, Mettler Toledo), with background subtraction for the solvent (MeCN). Excitation and emission spectra were recorded in solution using 3 mm path length quartz high precision cell cuvettes with a HORIBA Jobin Yvon spectrofluorometer (FluoroMax-3). Cyclic voltammetry was performed in a 30 mL glass cell using a using a modulab potentiostat (Solartron Analytical, AMETEK). The working electrode was a glassy carbon electrode, polished using a cotton polishing cloth with slurries of progressively finer alumina particles (1, 0.3 and 0.05 μm) purchased from BUEHLER, IL. The quasi-reference electrode was a silver wire, and the counter electrode was a Pt sheet.

B Description of Setup for Photochemical Experiments

All photoreactions were conducted in flat-bottomed borosilicate glass vials capped with microwave lids. The distance between the light source and the reaction mixture was 1 mm as the vials were in direct contact with the LEDs as seen in Figure S.1. The LEDs were purchased from Mouser Electronics, Sweden (https://www.mouser.se/ProductDetail/ams-OSRAM/LZ4-40B208-0000?qs=aDBU8ng1tb%2Fi1iXF2H7pQA%3D%3D accessed on 2024-01-29). The reaction temperature was measured by irradiating a capped vial containing dry and degassed MeCN for 30 minutes and then directly inserting a thermocouple thermometer into the solution to record the temperature.

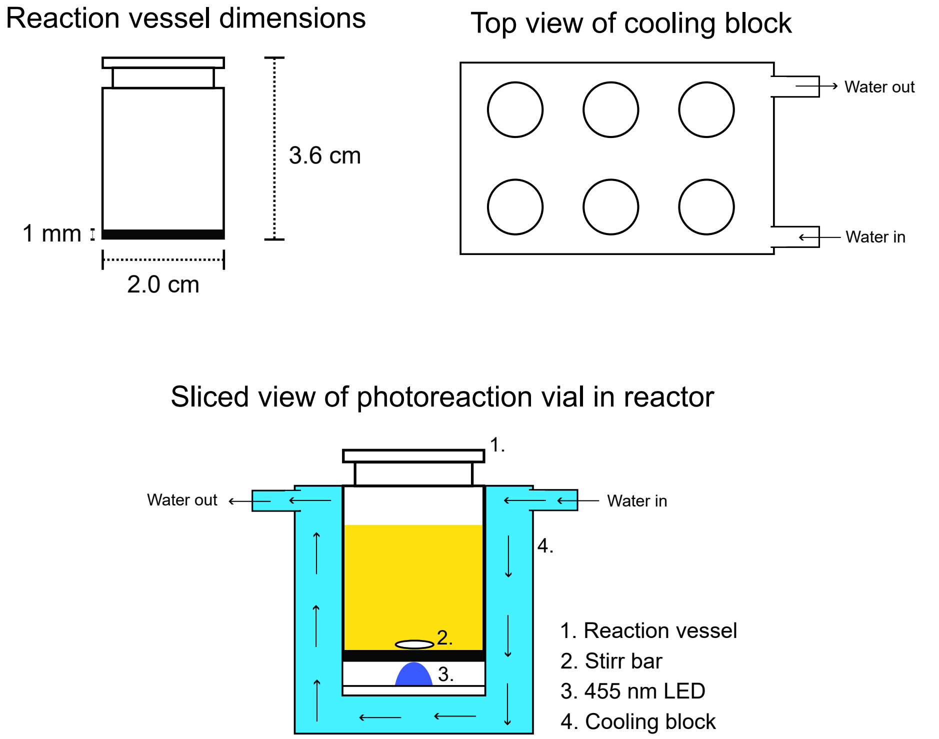

Figure S.1: Schematic drawings of reaction vessel (top left), cooling block (top right) and sliced view of photoreaction vial in the reactor (bottom).

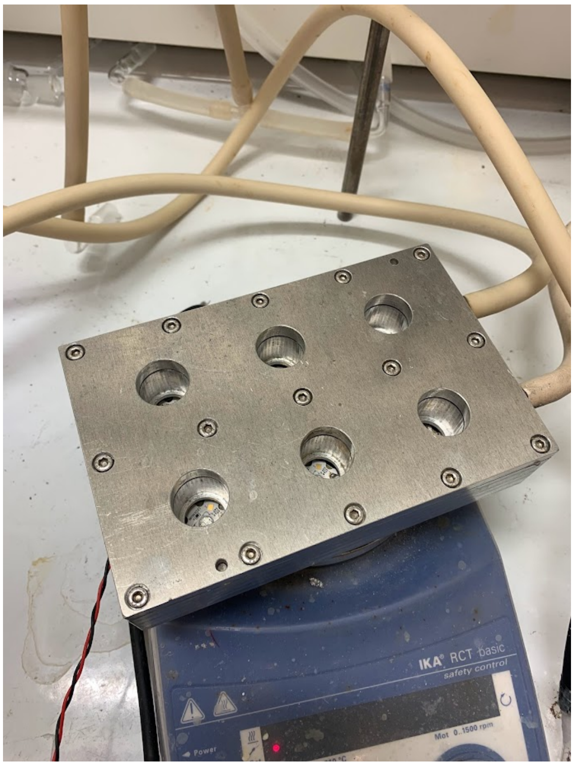

Figure S.2: 455 nm LED setup on a stir plate with water cooling block attached.

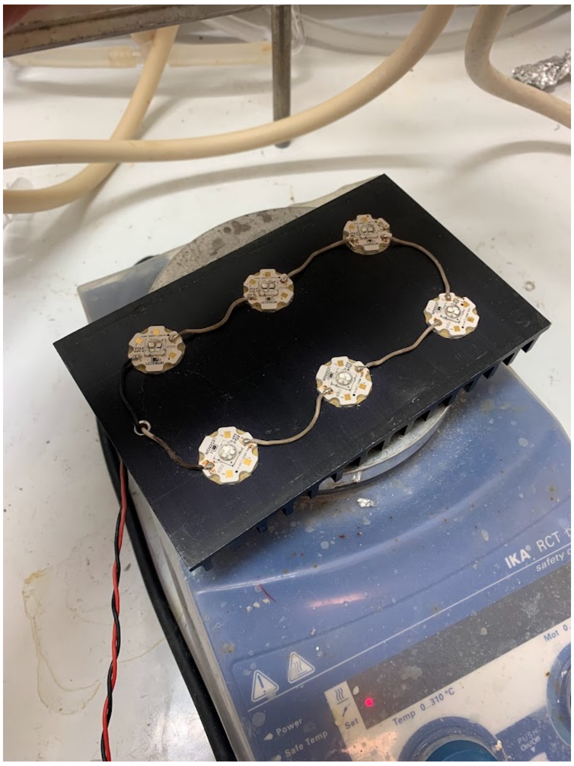

Figure S.3: 455 nm LED setup on a stir plate without water cooling block attached.

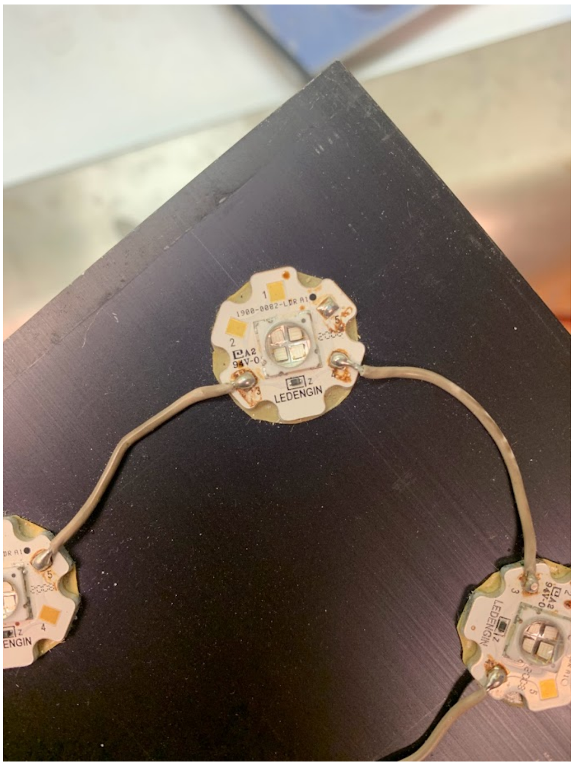

Figure S.4: 455 nm LEDs.

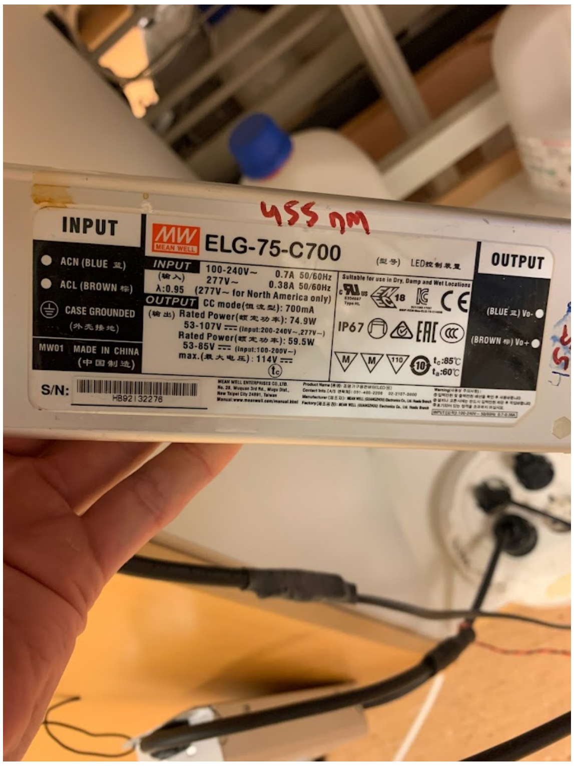

Figure S.5: 455 nm LED power supply.

C Control Experiments

Table S.1: Control experiments.

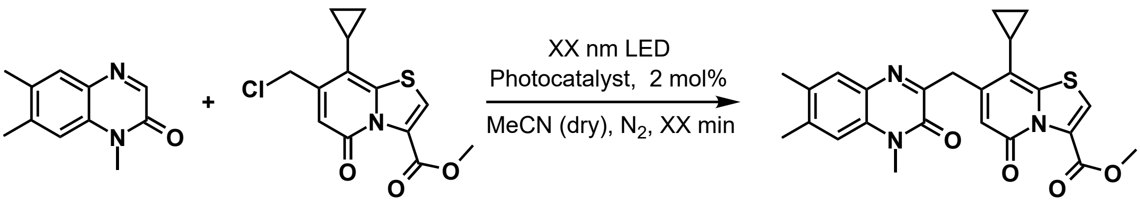

1c (1.2 equiv.) **2a** (1.0 equiv.) **3ca**

Entry	Photocatalyst (additive)	Wavelength (nm)	Time (min)	Yield (%)
1	None	395	240	0
2	$Ir(PPy)_3$	No light[a]	3 days	0
3	$Ir(PPy)_3$	No light[b]	3 days	0

[a]Reaction mixture was stirred at 80 °C in the dark. [b]Reaction mixture was stirred at room temperature in the dark.

D Scaled up Synthesis of 3aa

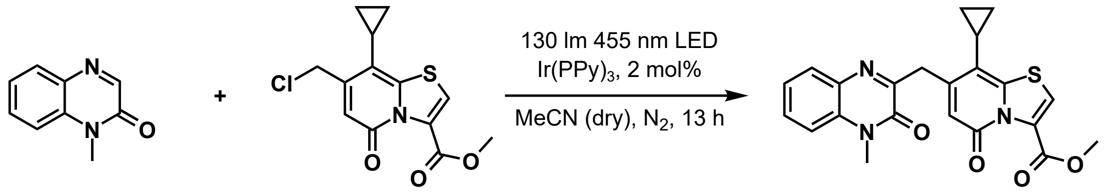

1a (2.02 mmol, 324 mg) **2a** (1.68 mmol, 501 mg) **3aa** (48%, 343 mg)

Figure S.6: Scaled up reaction between **2a** and **1a**.

2a (501 mg, 1.68 mmol, 1.0 equiv.), **1a** (324 mg, 2.02 mmol, 1.2 equiv.) and, Ir(PPy)$_3$ (22 mg, 0.034 mmol, 0.02 equiv.) were added to an oven-dried 20 mL microwave vial which was then sealed with a Biotage septum cap for microwave reaction vials. The vial was evacuated and backfilled with $N_{2(g)}$ and 16 mL of dry MeCN was then added. The yellow solution was degassed by purging with $N_{2(g)}$ for 5 minutes. The mixture was then stirred at rt with air cooling while irradiating with 455 nm LEDs with an input power of 10 W and output luminous flux of 130 lm for 13 h.

The dark solution was transferred to a separatory funnel with EtOAc (100 mL), deionized water (100 mL) and, brine (20 mL). The aqueous phase was extracted with EtOAc (3x100 mL). The combined organic phases were then dried over Na_2SO_4 and filtered. The solvent was removed under reduced pressure giving a black sticky solid. The crude was dissolved in DCM and purified with automated flash column chromatography (gradient, 0% → 100% EtOAc in heptane). The combined fractions were recrystallized from boiling MeOH (30 mL) giving **3aa** as an orange amorphous solid (343 mg, 0.815 mmol, 48%).

E Stern-Volmer Experiments

Stern-Volmer measurements were carried out to investigate the potential quenching of the photoredox catalyst additive Ir(PPy)$_3$ by **2a**. Comparison between the absorption spectrum of Ir(PPy)$_3$ (Figure S.7a) and the emission spectrum of **2a** (Figure S.7b), both measured in MeCN, revealed a large spectral overlap. To avoid unwanted excitation/absorption of **2a**, the fluorescence quenching experiments of Ir(PPy)$_3$ were performed with excitation at 340 nm where Ir(PPy)$_3$ still has considerable absorption, while the absorption from **2a** (Figure S.7c) is minimal.

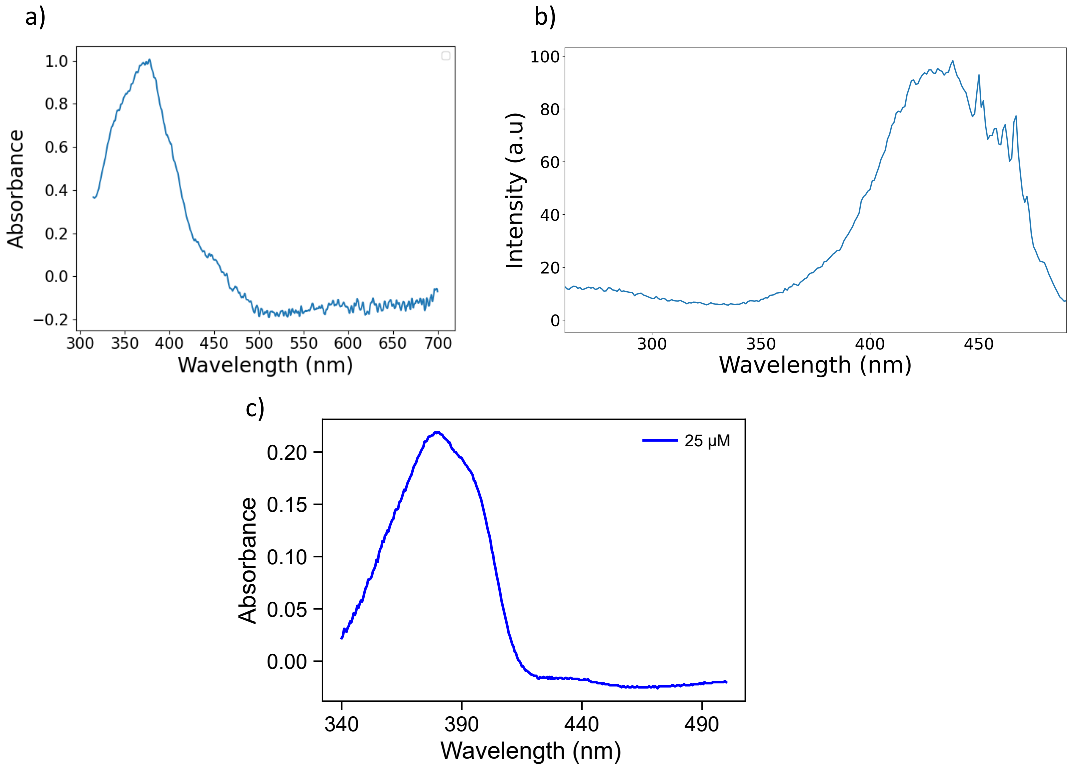

Figure S.7: a) Absorption spectrum of Ir(PPy)$_3$ in MeCN. b) Emission spectrum of **2a** in MeCN. c) Absorption spectrum of **2a** (25 μM) in MeCN.

Since both Ir(PPy)$_3$ and **2a** are absorbing at 340 nm, proper concentration ranges for both Ir(PPy)$_3$ and **2a** had to be identified so that inner filtering as well as aggregation effects could be neglected. First, fluorescence emission spectra were recorded for Ir(PPy)$_3$, at concentrations up to 200 μM. Within this concentration range the emission intensity of Ir(PPy)$_3$ increased linearly with concentration, with no indication of aggregation or inner filtering effects (Figure S.8a). Second, although the absorption of **2a** is low at 340 nm, there was a possibility that the fluorescence intensity would not be negligible at higher concentrations. To investigate this, fluorescence emission spectra of **2a** at 340 nm excitation were recorded at different concentrations. For concentrations up to 125 μM, a linear increase in the fluorescence emission was observed (Figure S.8b). Above 125 μM the emission intensity became saturated and even started decreasing with higher concentrations (Figure S.8c), likely attributed to an inner filtering effect.

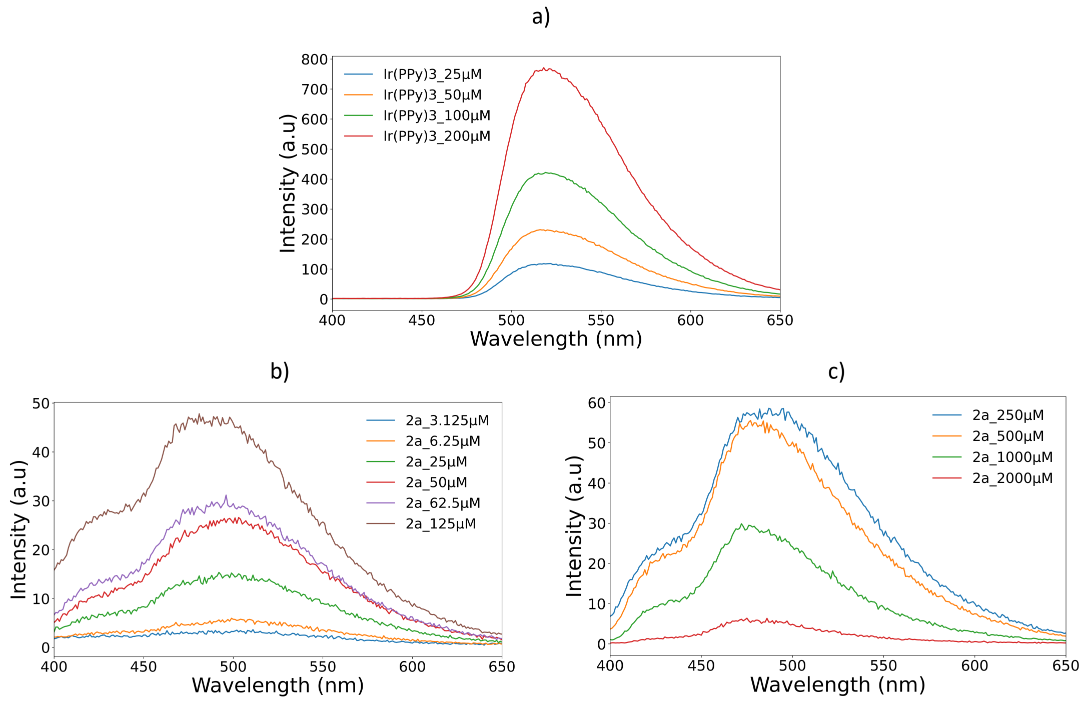

Figure S.8: a) Fluorescence emission spectra of $Ir(PPy)_3$ in MeCN at various concentrations with 340 nm excitation. b) & c) Fluorescence emission spectra of **2a** at different concentrations with excitation at 340 nm.

Hence, to avoid inner filtering and aggregation effects in the Stern-Volmer measurements, the concentrations of $Ir(PPy)_3$ and **2a** were kept at 100 μM and within 0-100 μM, respectively, where linear responses to concentration changes of the individual compounds were observed. Figure S.9a shows the recorded fluorescence emission spectra of $Ir(PPy)_3$ (100 μM in MeCN) upon titration with **2a** from 0 to 100 μM. In the recorded spectra, a gradual rise of a shoulder in the blue side can be noted, likely attributed to fluorescence emission from **2a**. The main emission peak centered around 520 nm in Figure S.9a should, however, originate from $Ir(PPy)_3$ emission. We note in these spectra that the $Ir(PPy)_3$ fluorescence is significantly quenched by addition of **2a**, and a Stern-Volmer plot yields a close to linear dependence on the concentration of **2a**, as shown in Figure S.9b.

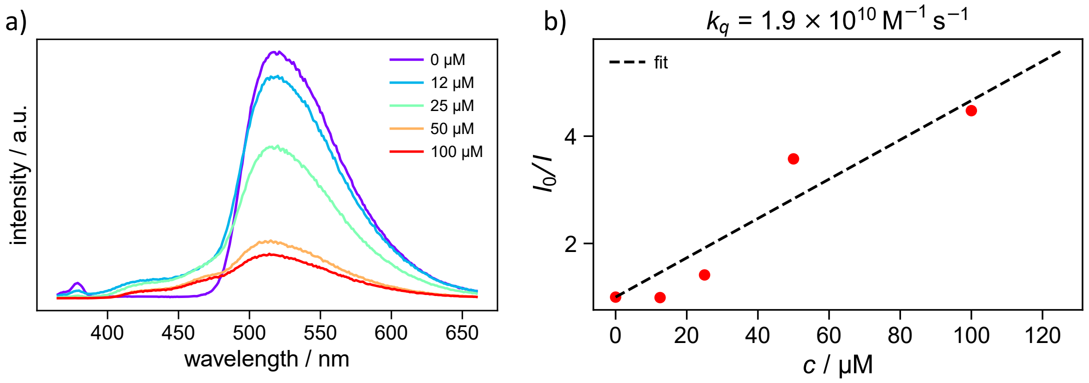

Figure S.9: a) Overlapped fluorescence emission spectra of $Ir(PPy)_3$ in non-degassed MeCN (100 μM) with different concentrations of **2a** with excitation at 340 nm. b) Stern-Volmer plot with the quenching rate constant (k_q) calculated from the linear fit, using $\tau_0 = 1.9$ μs for $Ir(PPy)_3$[1] under oxygen-free conditions.

F Cyclic Voltammetry

Cyclic voltammetry was performed in a 30 mL glass cell containing tetrabutylammonium hexafluorophosphate (NBu_4PF_6, 0.1 M) and sample (5.0 mM) in MeCN (10 mL). The working electrode was a glassy carbon electrode (cylindrical shape, 2 mm diameter and 3.14 mm^2 surface area), polished using a cotton polishing cloth with slurries of progressively finer alumina particles (1, 0.3 and 0.05 μm) purchased from BUEHLER, IL. The quasi-reference electrode was a silver wire, and the counter electrode was a Pt sheet. Sample solutions were purged with N_2 for 10 min prior to the experiment. All cyclic voltammetry measurements were performed with a 50 mV/s scan rate over a potential window between +2.5 V to -2.5 V using a modulab potentiostat (Solartron Analytical, AMETEK) and recorded potentials were referenced to that of ferrocene. Figure S.10 shows the cyclic voltammogram of ferrocene using this setup. Ferrocene was purified by vacuum sublimation at 80°C prior to measurement.

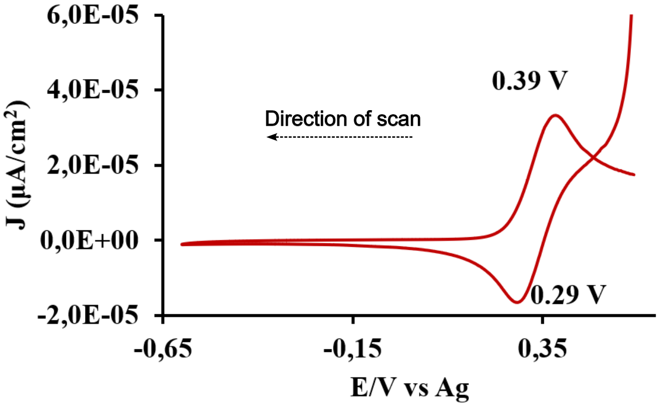

Figure S.10: Cyclic voltammogram of vacuum sublimed ferrocene. Performed with [Ferrocene] = 5 mM and [NBu_4PF_6] = 100 mM at a scan rate of 10 mV/s starting at 0.6 V. $E_{1/2} = 0.34$ V.

G Copies of ^{1}H, ^{13}C, ^{19}F, gHSQC, and gHMBC spectra

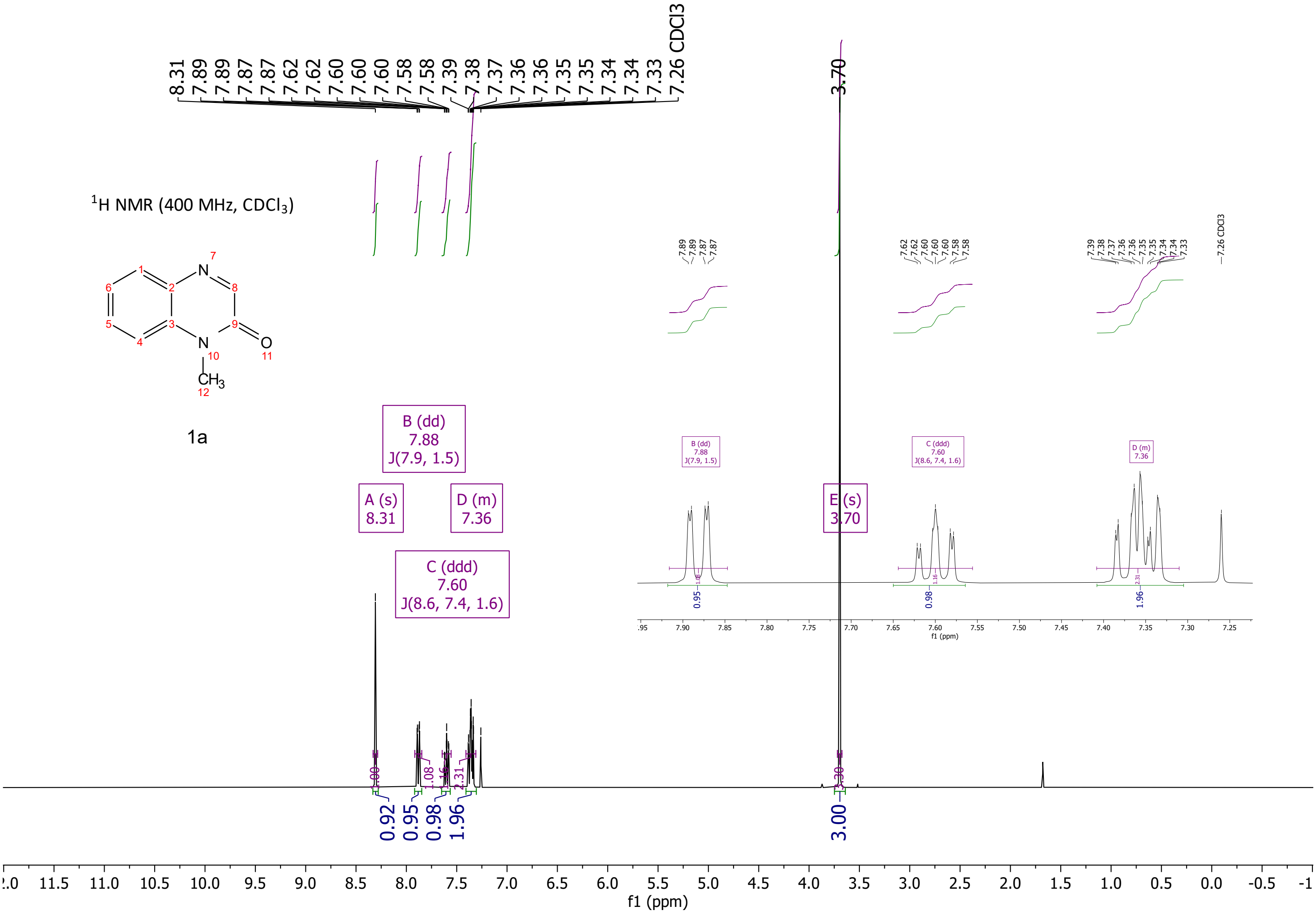

Figure S.11: ^{1}H NMR spectrum ($CDCl_3$, 400 MHz) of 1-methylquinoxalin-2(1H)-one, **1a**.

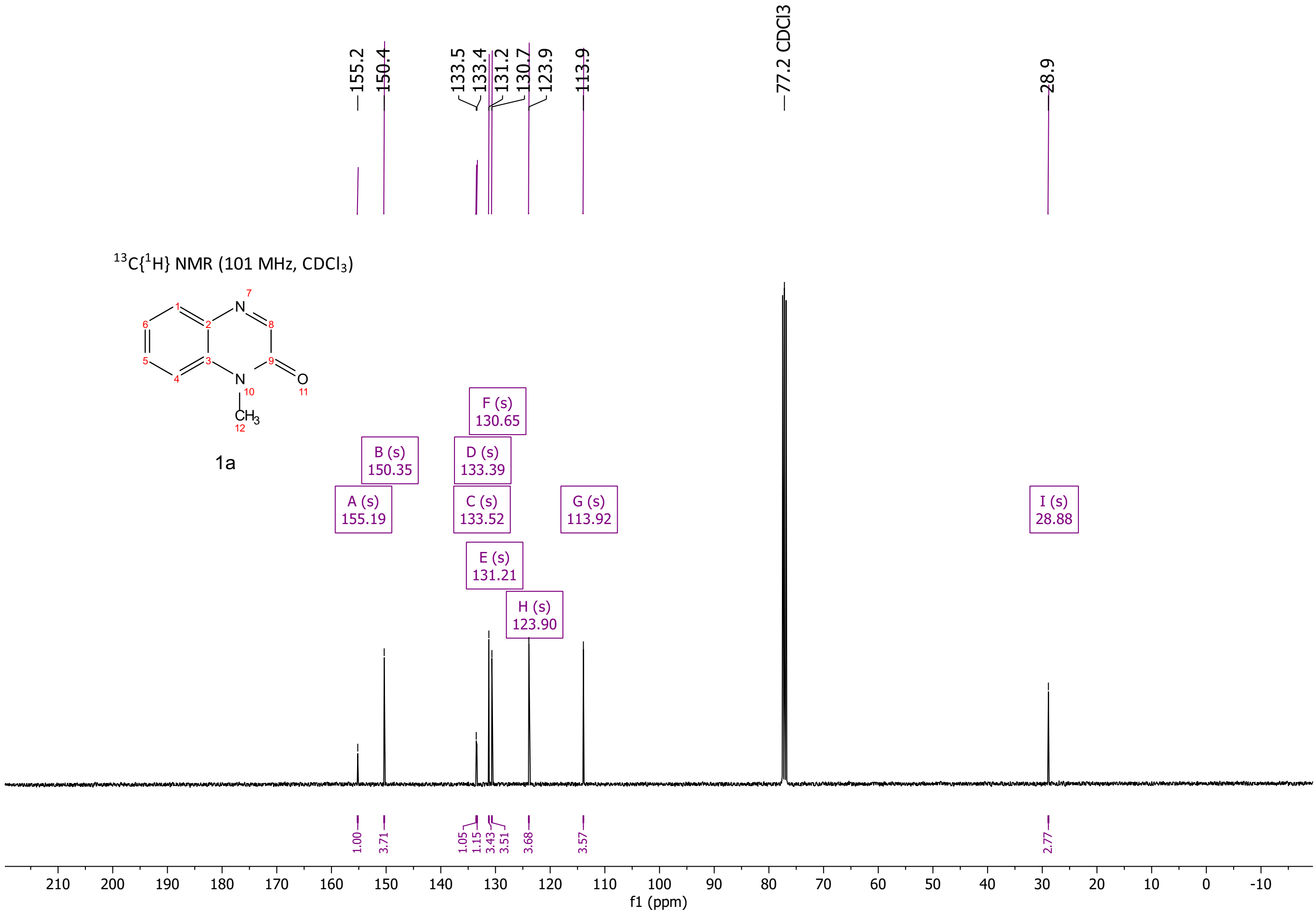

Figure S.12: $^{13}C\{^{1}H\}$ NMR spectrum ($CDCl_3$, 101 MHz) of 1-methylquinoxalin-2(1H)-one, **1a**.

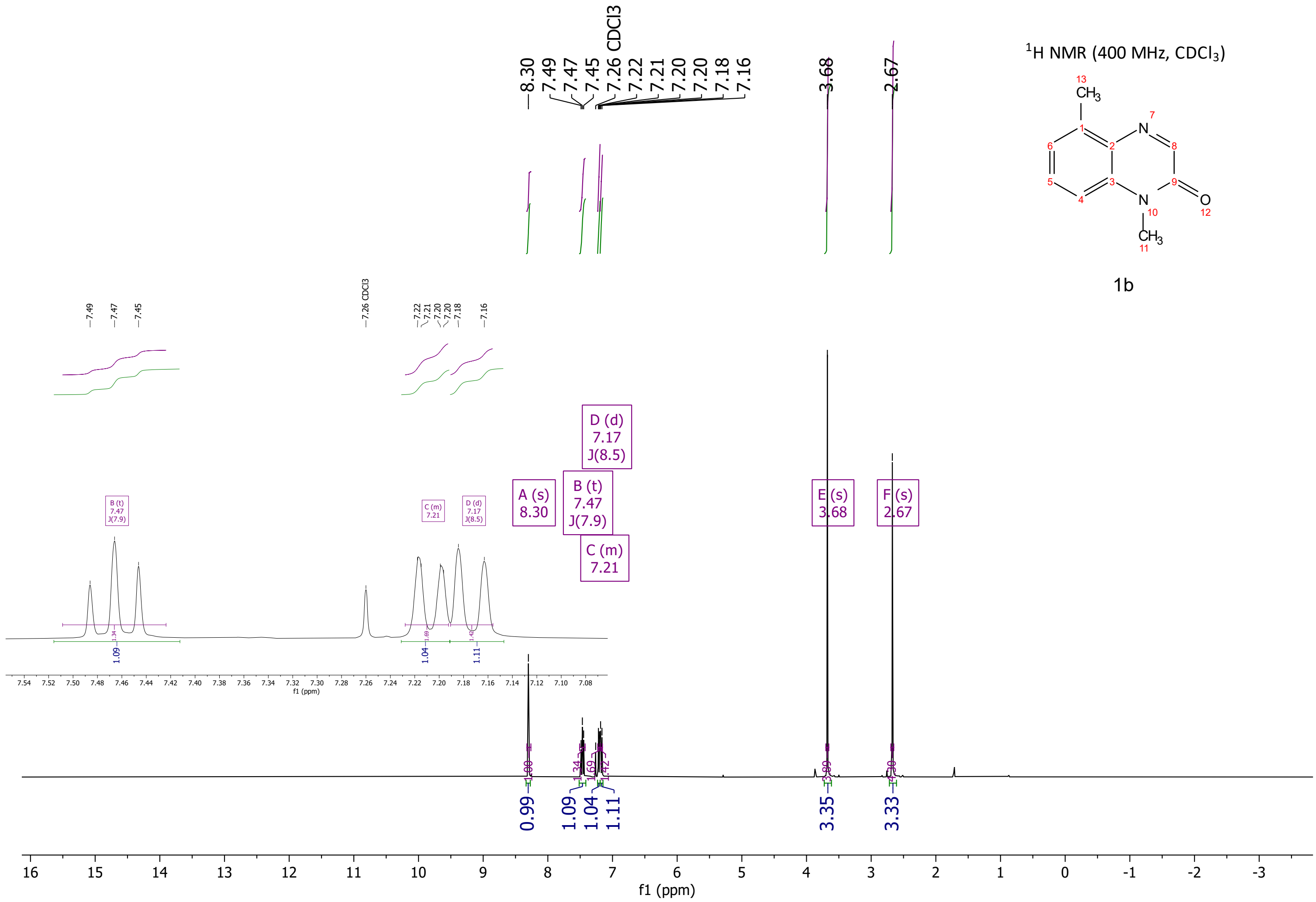

Figure S.13: ^{1}H NMR spectrum ($CDCl_3$, 400 MHz) of 1,5-dimethylquinoxalin-2(1H)-one, **1b**.

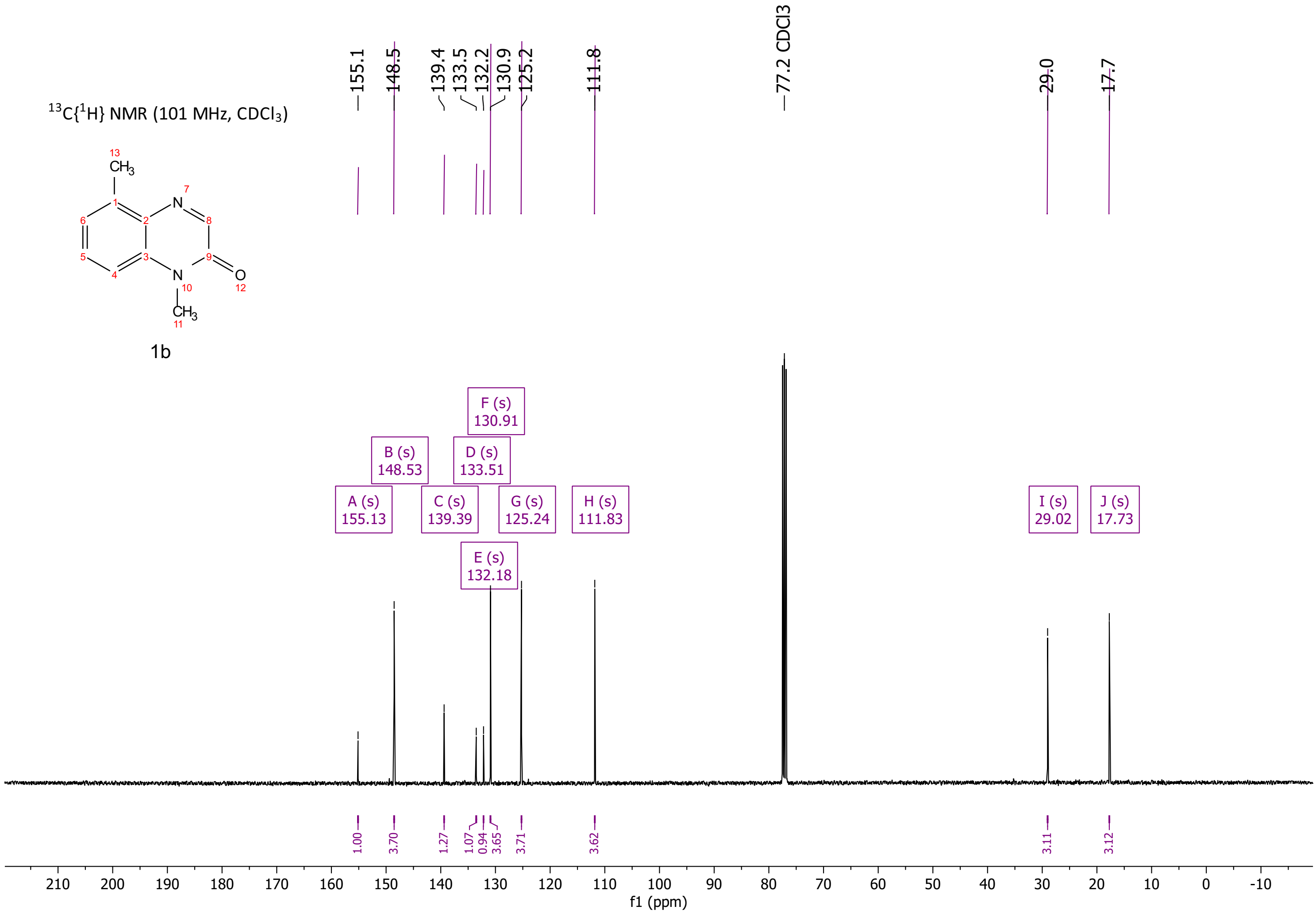

Figure S.14: $^{13}C\{^1H\}$ NMR spectrum ($CDCl_3$, 101 MHz) of 1,5-dimethylquinoxalin-2(1H)-one, **1b**.

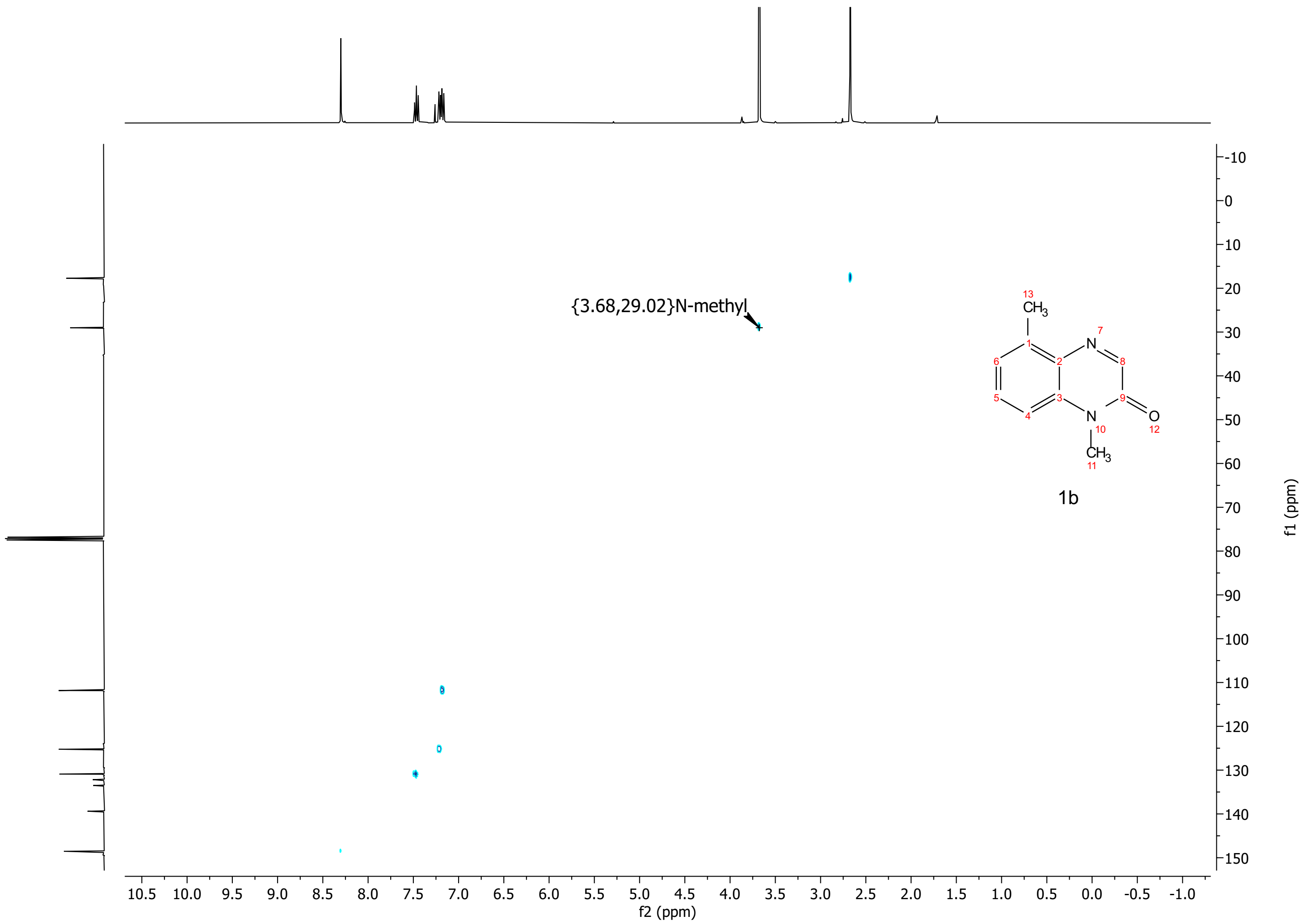

Figure S.15: gHSQC spectrum ($CDCl_3$) of 1,5-dimethylquinoxalin-2(1H)-one, **1b**.

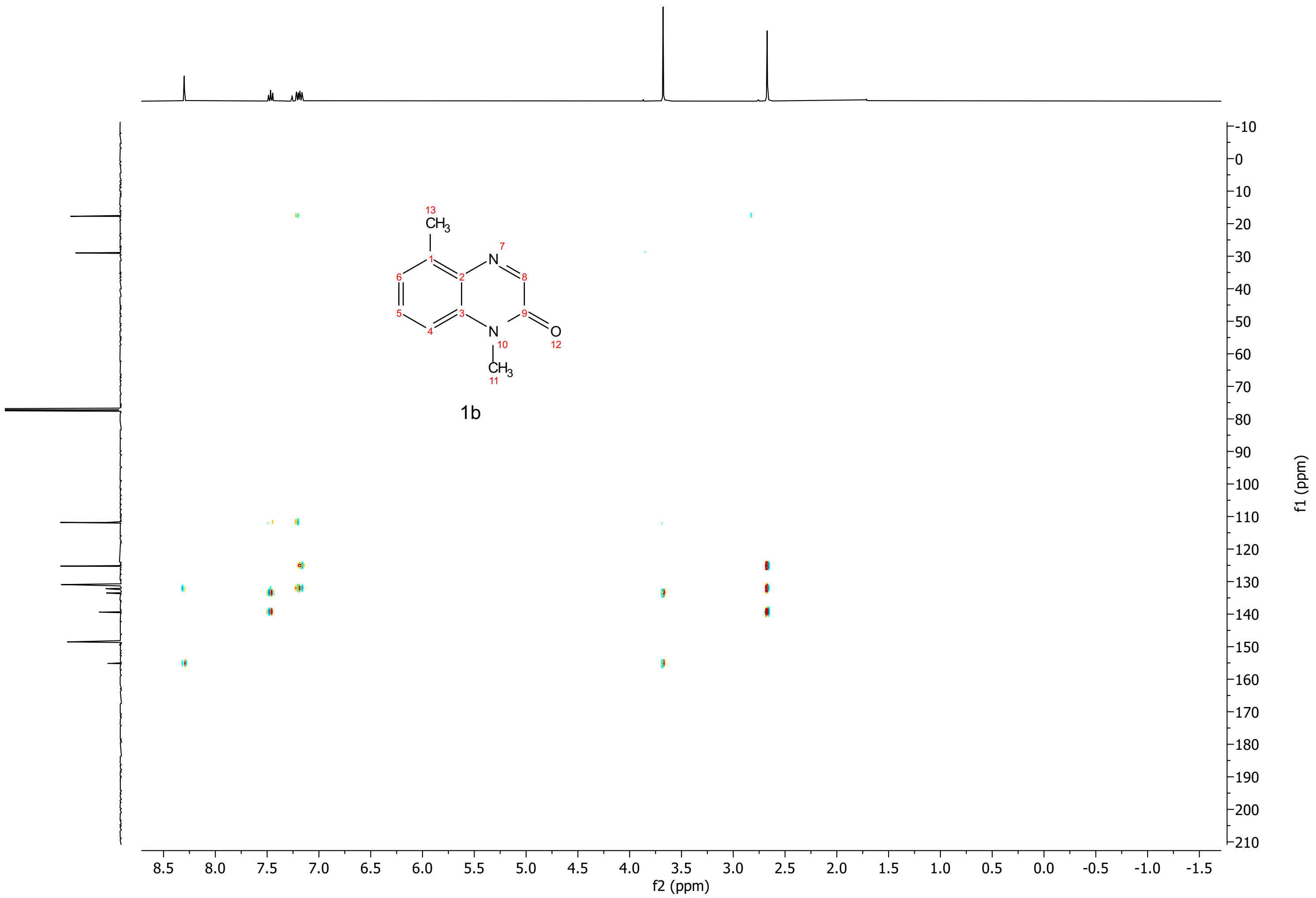

Figure S.16: gHMBC spectrum ($CDCl_3$) of 1,5-dimethylquinoxalin-2(1H)-one, **1b**.

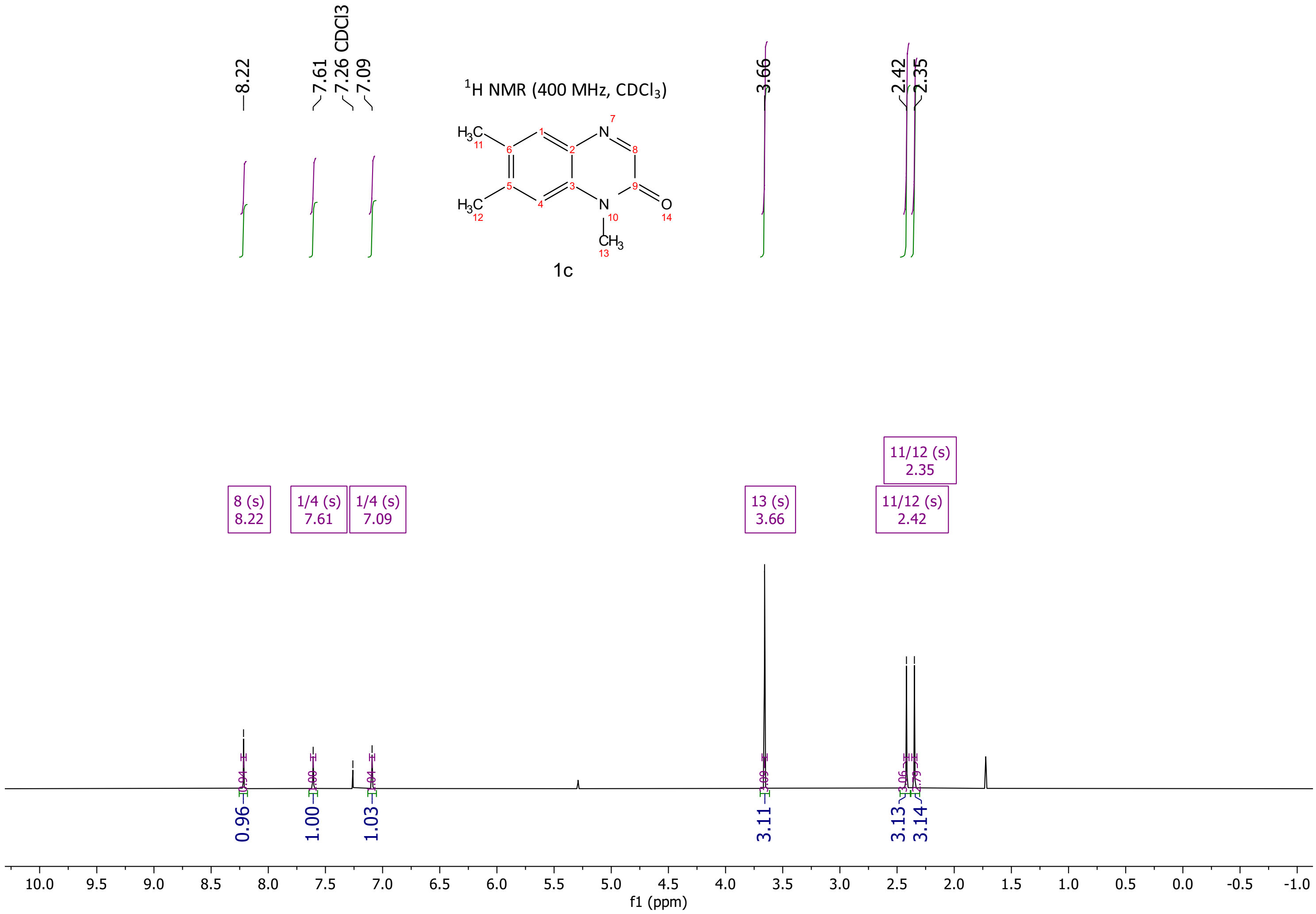

Figure S.17: ^{1}H NMR spectrum ($CDCl_3$, 400 MHz) of 1,6,7-trimethylquinoxalin-2(1H)-one, **1c**.

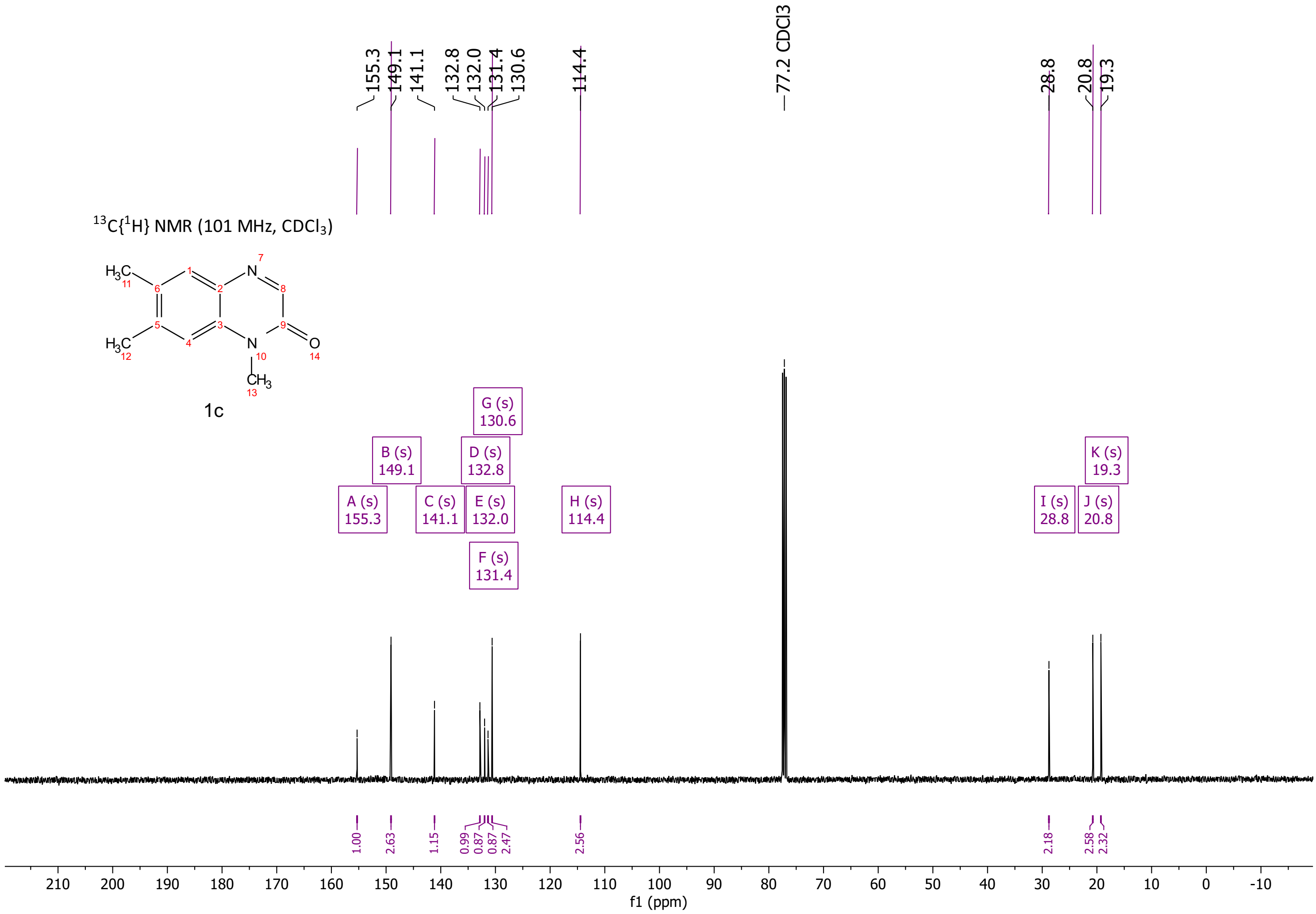

Figure S.18: $^{13}C\{^1H\}$ NMR spectrum ($CDCl_3$, 101 MHz) of 1,6,7-trimethylquinoxalin-2(1H)-one, **1c**.

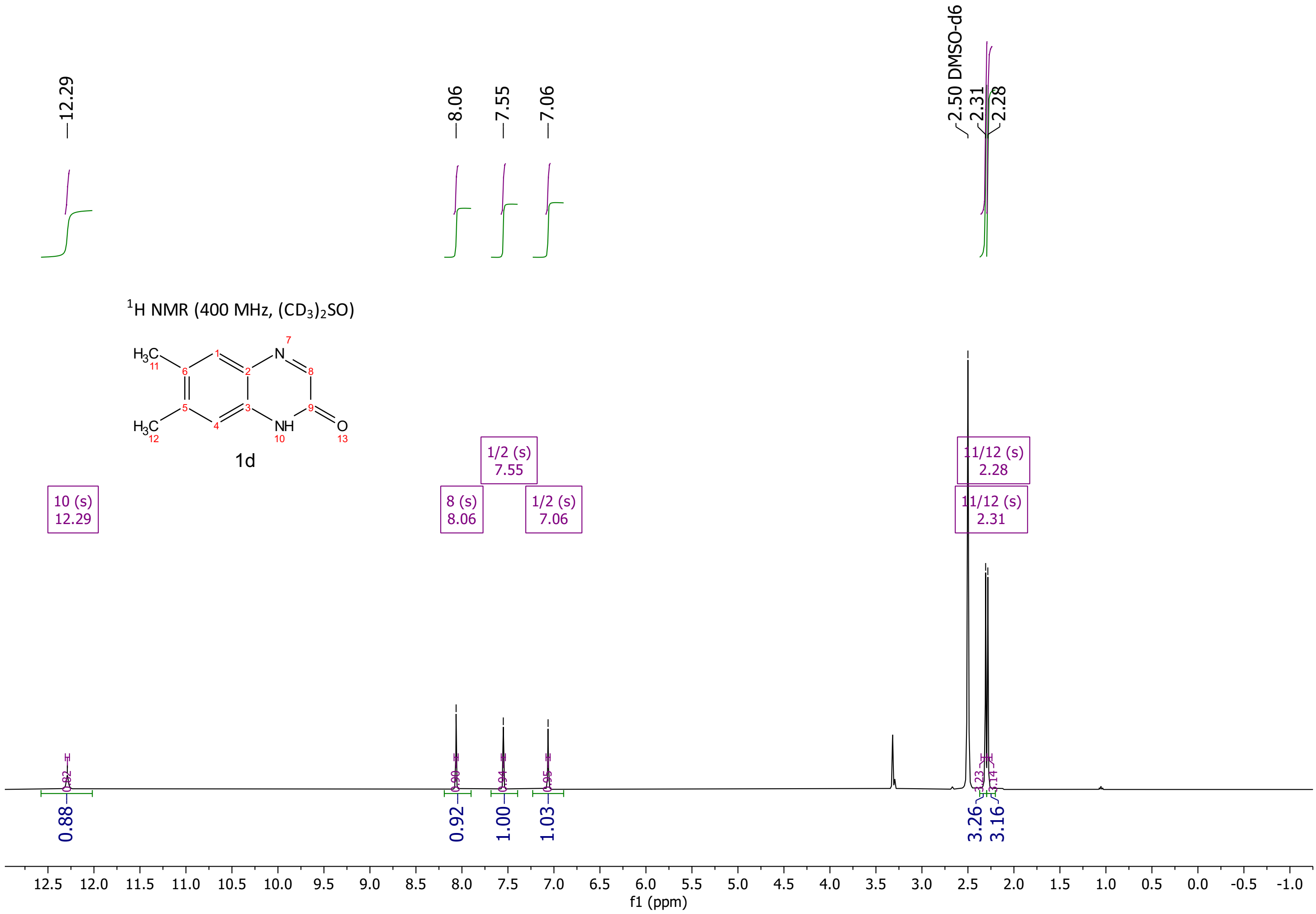

Figure S.19: ^{1}H NMR spectrum ($(CD_3)_2SO$, 400 MHz) of 6,7-dimethylquinoxalin-2(1H)-one, **1d**.

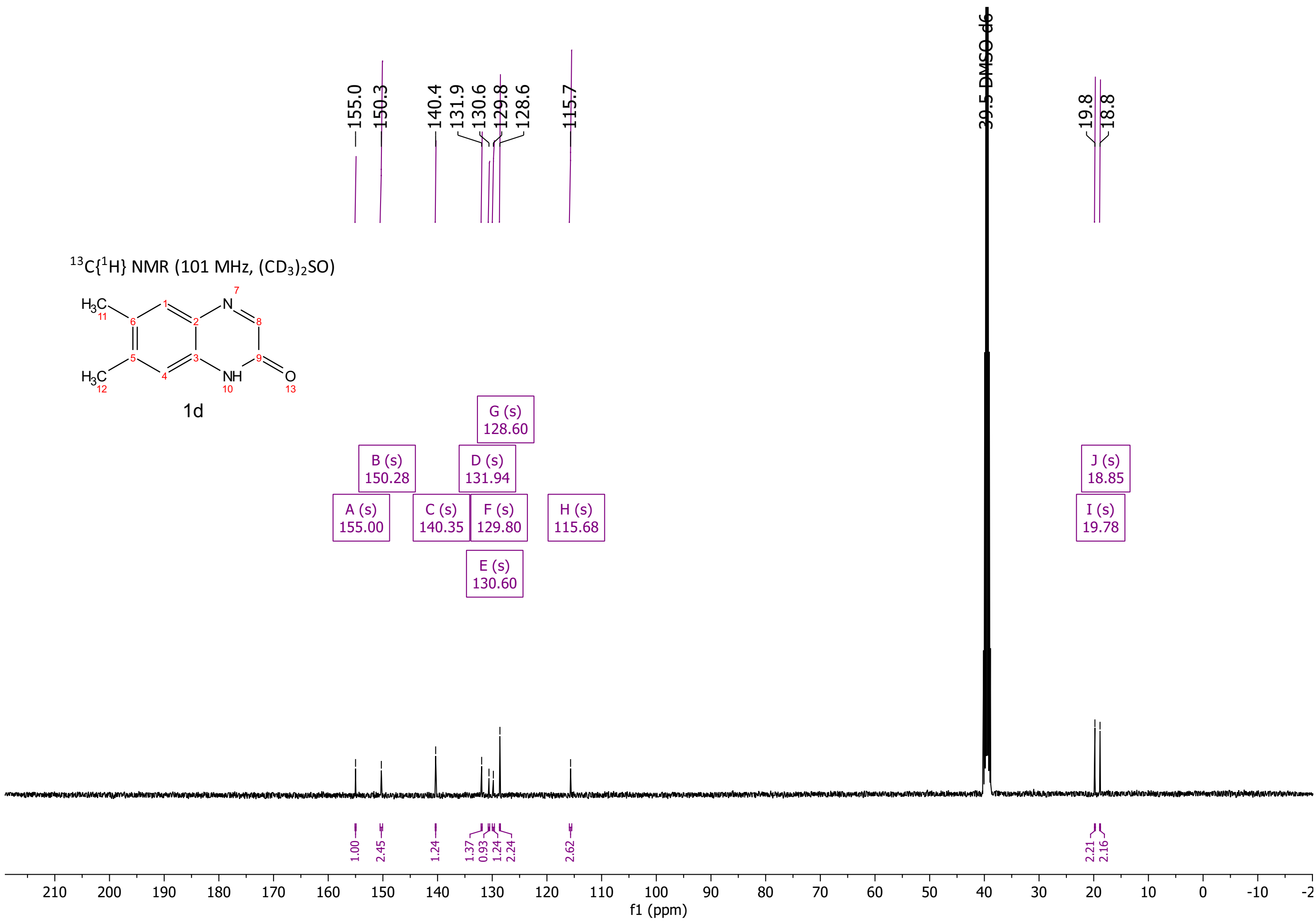

Figure S.20: $^{13}C\{^1H\}$ NMR spectrum ($(CD_3)_2SO$, 101 MHz) of 6,7-dimethylquinoxalin-2(1H)-one, **1d**.

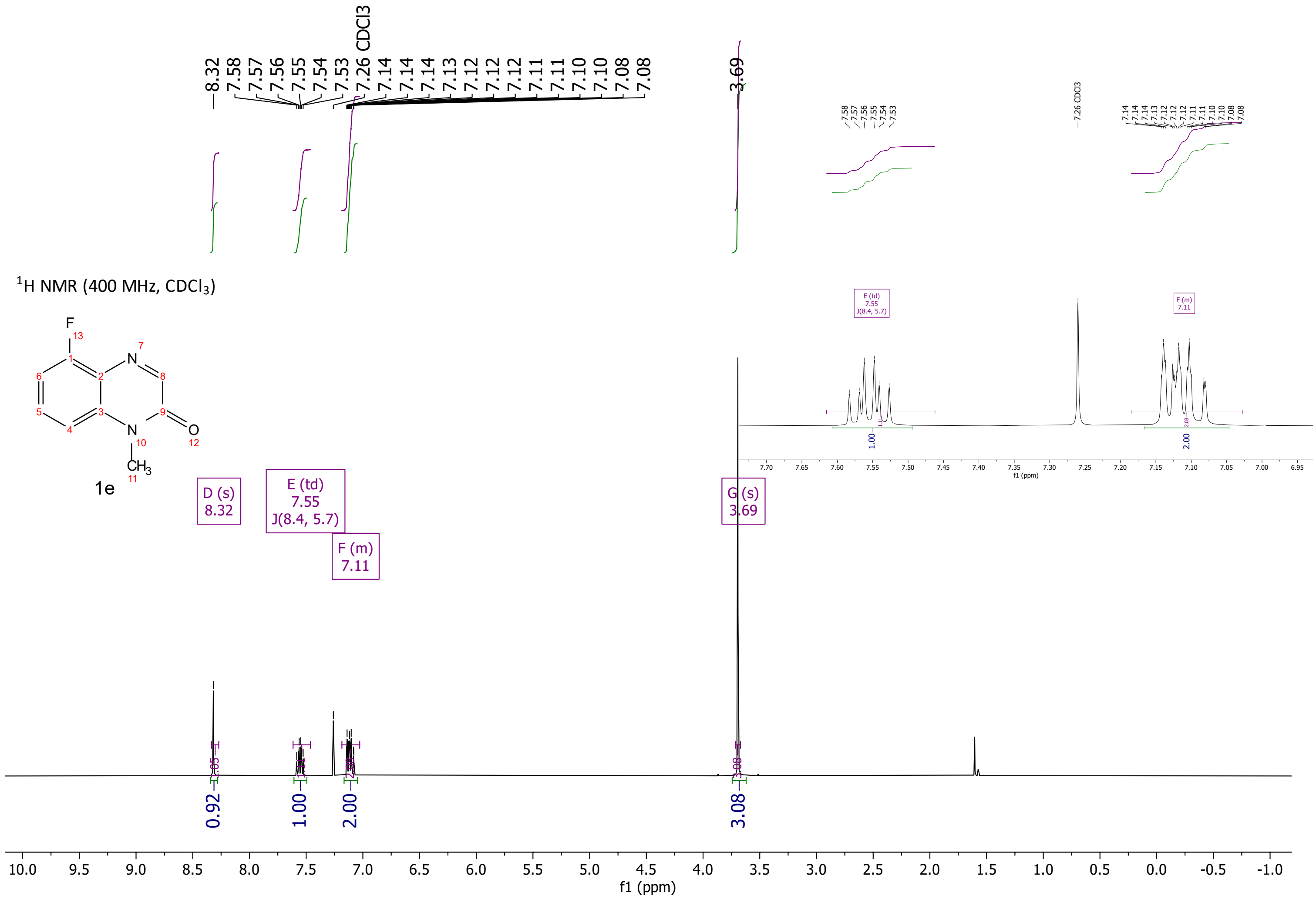

Figure S.21: ^{1}H NMR spectrum ($CDCl_3$, 400 MHz) of 5-Fluoro-1-methyl-2(1H)-quinoxalinone, **1e**.

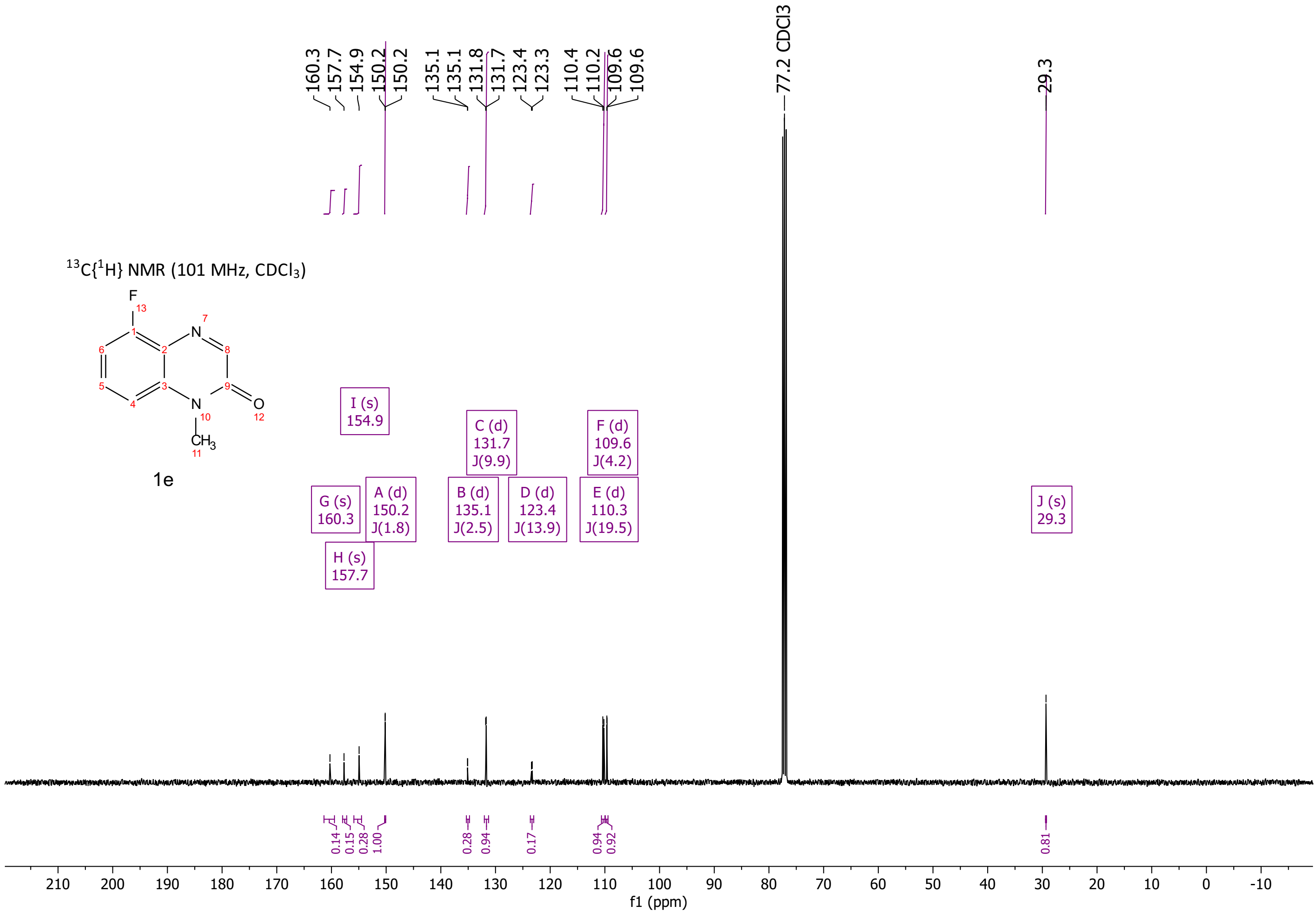

Figure S.22: ^{13}C{^{1}H} NMR spectrum ($CDCl_3$, 101 MHz) of 5-Fluoro-1-methyl-2(1H)-quinoxalinone, **1e**.

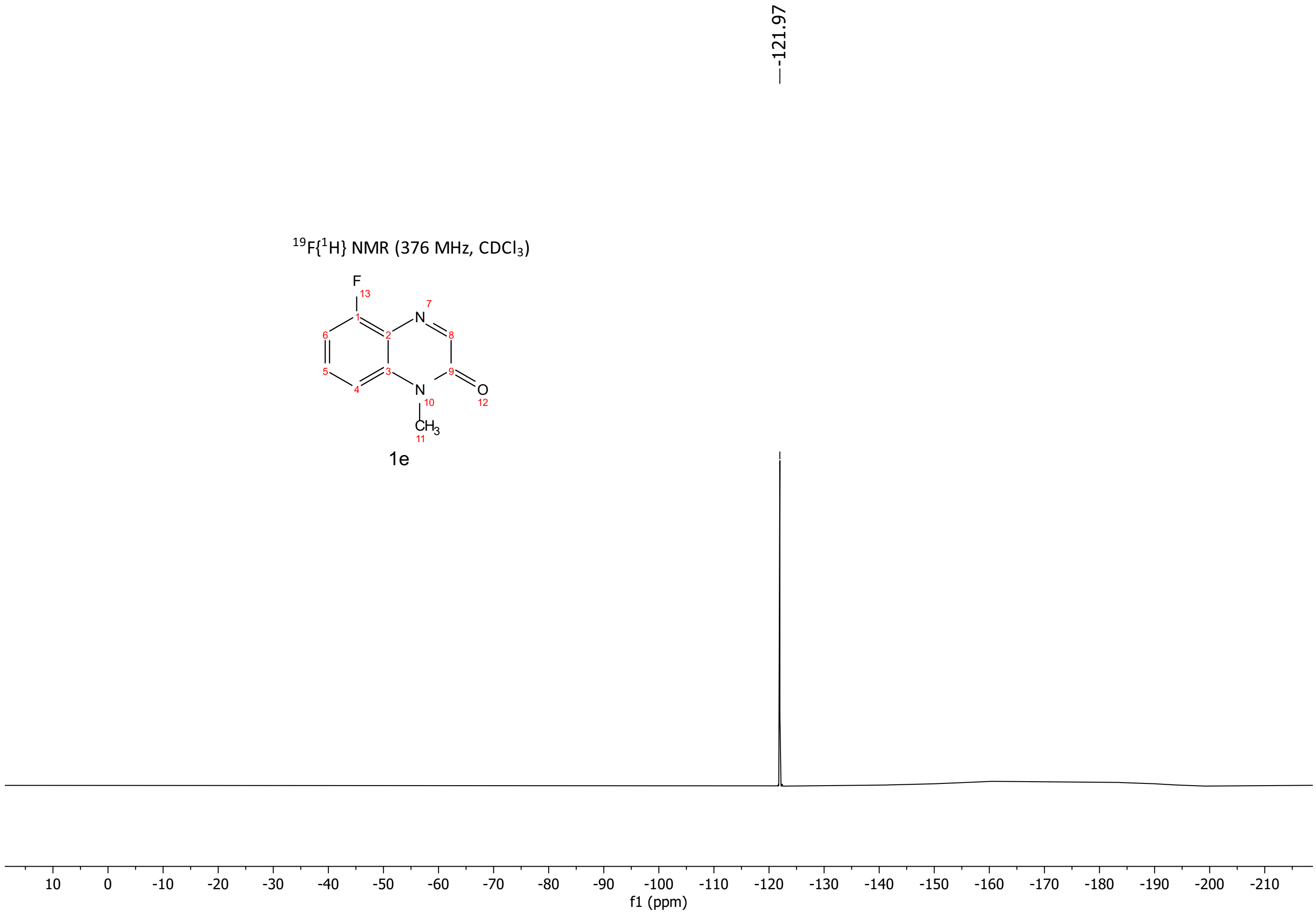

Figure S.23: $^{19}F\{^1H\}$ NMR spectrum ($CDCl_3$, 376 MHz) of 5-Fluoro-1-methyl-2(1H)-quinoxalinone, **1e**.

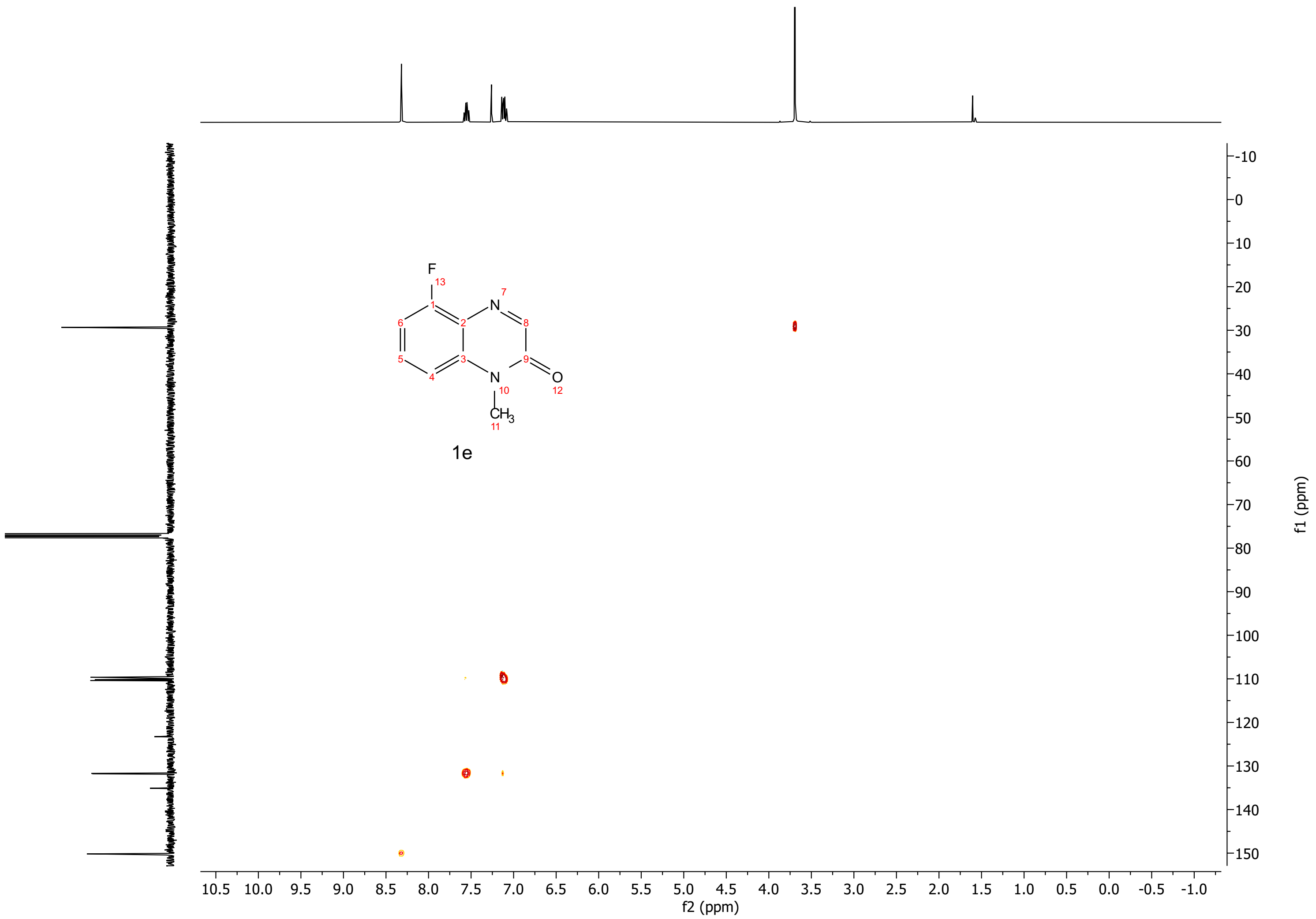

Figure S.24: gHSQC spectrum ($CDCl_3$) of 5-Fluoro-1-methyl-2(1H)-quinoxalinone, **1e**.

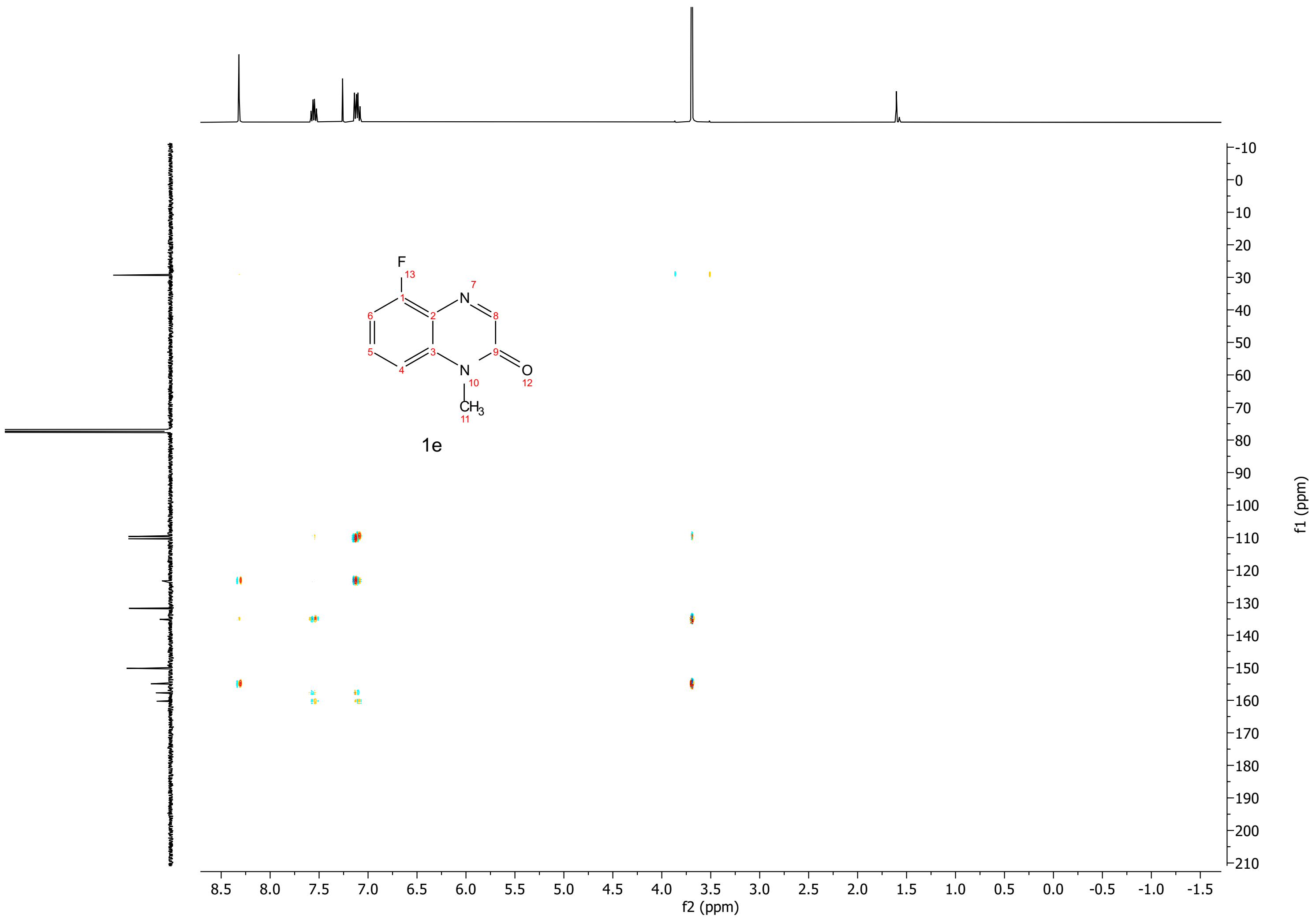

Figure S.25: gHMBC spectrum ($CDCl_3$) of 5-Fluoro-1-methyl-2(1H)-quinoxalinone, **1e**.

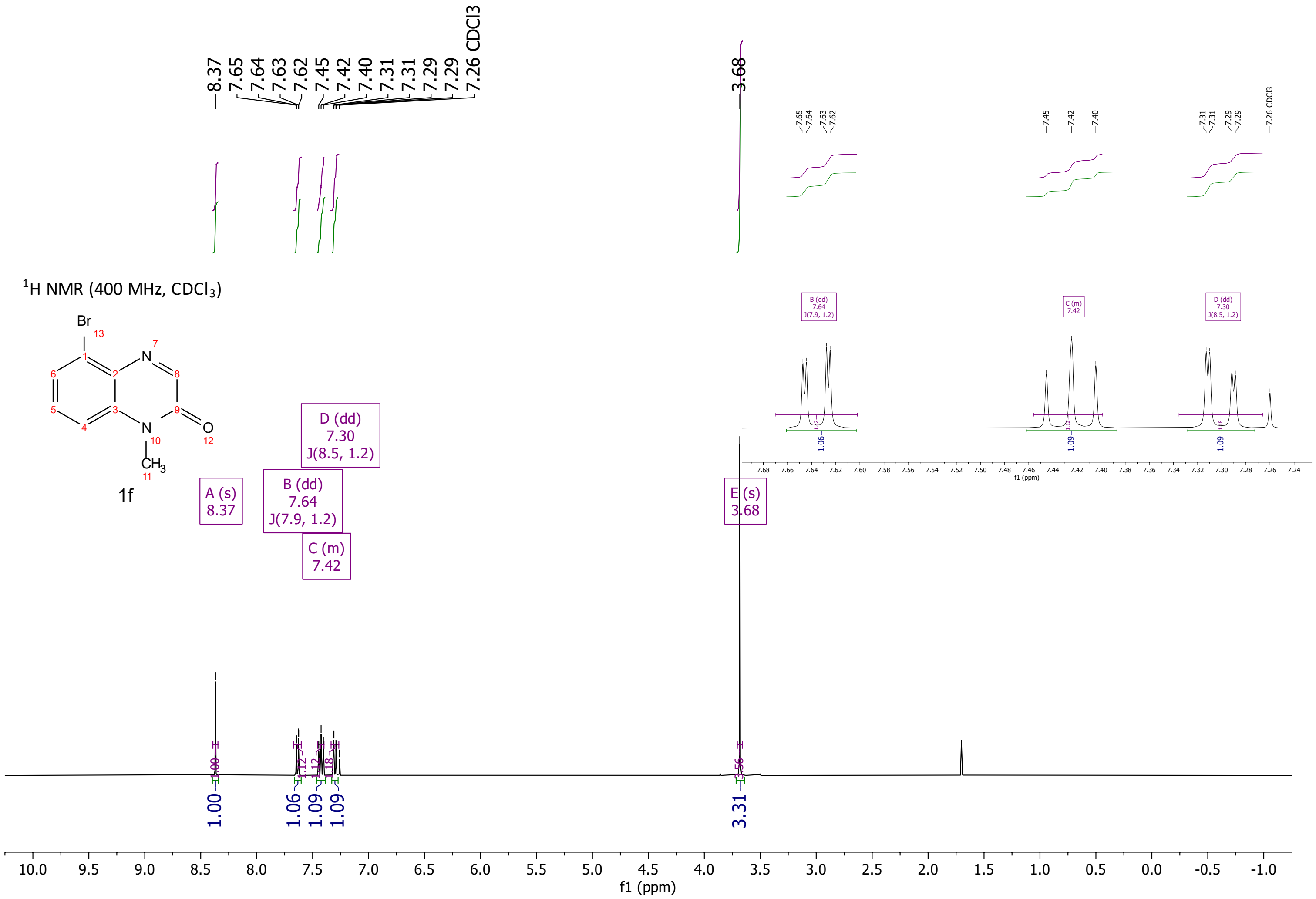

Figure S.26: ^{1}H NMR spectrum ($CDCl_3$, 400 MHz) of 5-bromo-N-methylquinoxalin-2(1H)-one, **1f**.

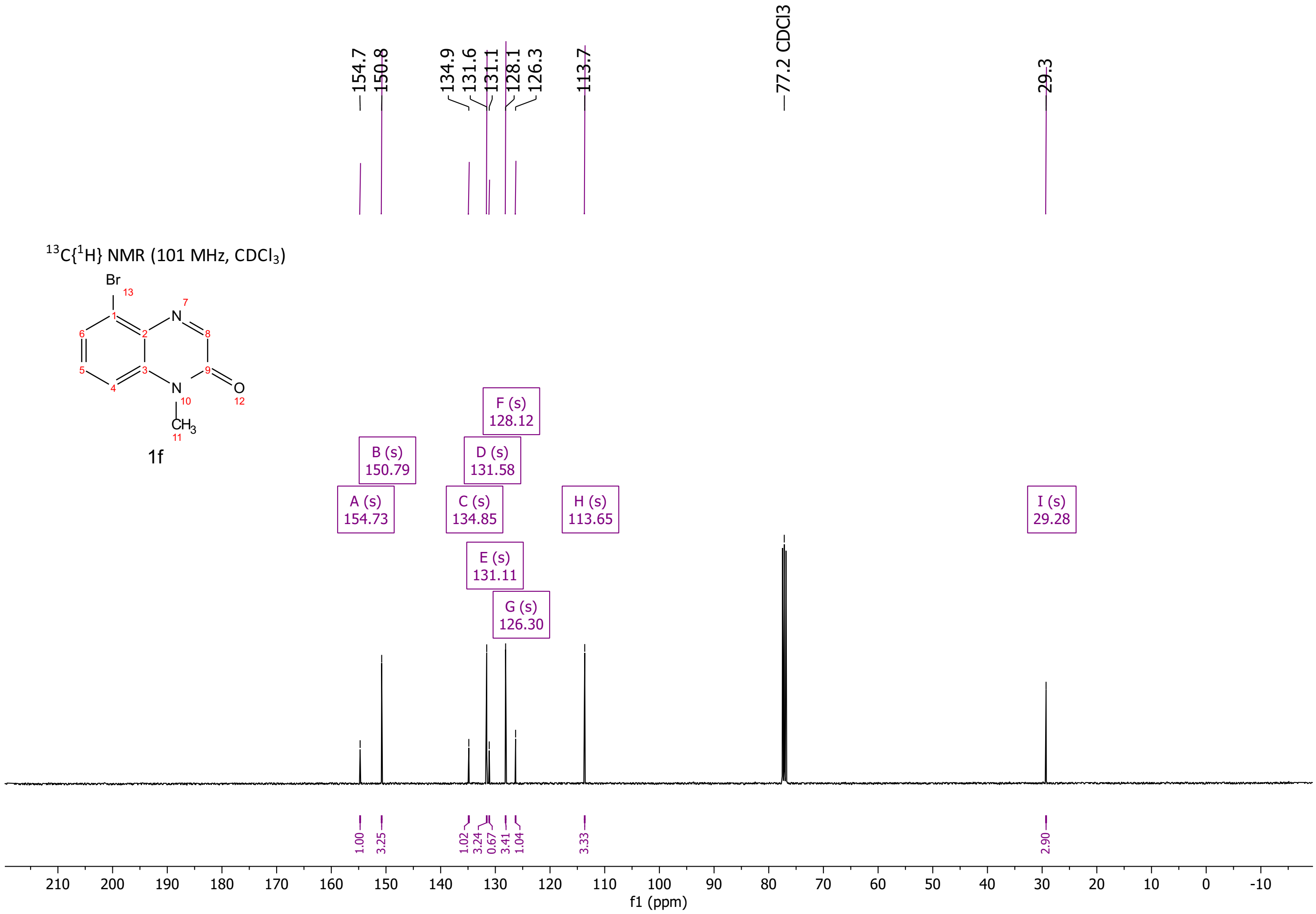

Figure S.27: $^{13}C\{^{1}H\}$ NMR spectrum ($CDCl_3$, 101 MHz) of 5-bromo-*N*-methylquinoxalin-2(1H)-one, **1f**.

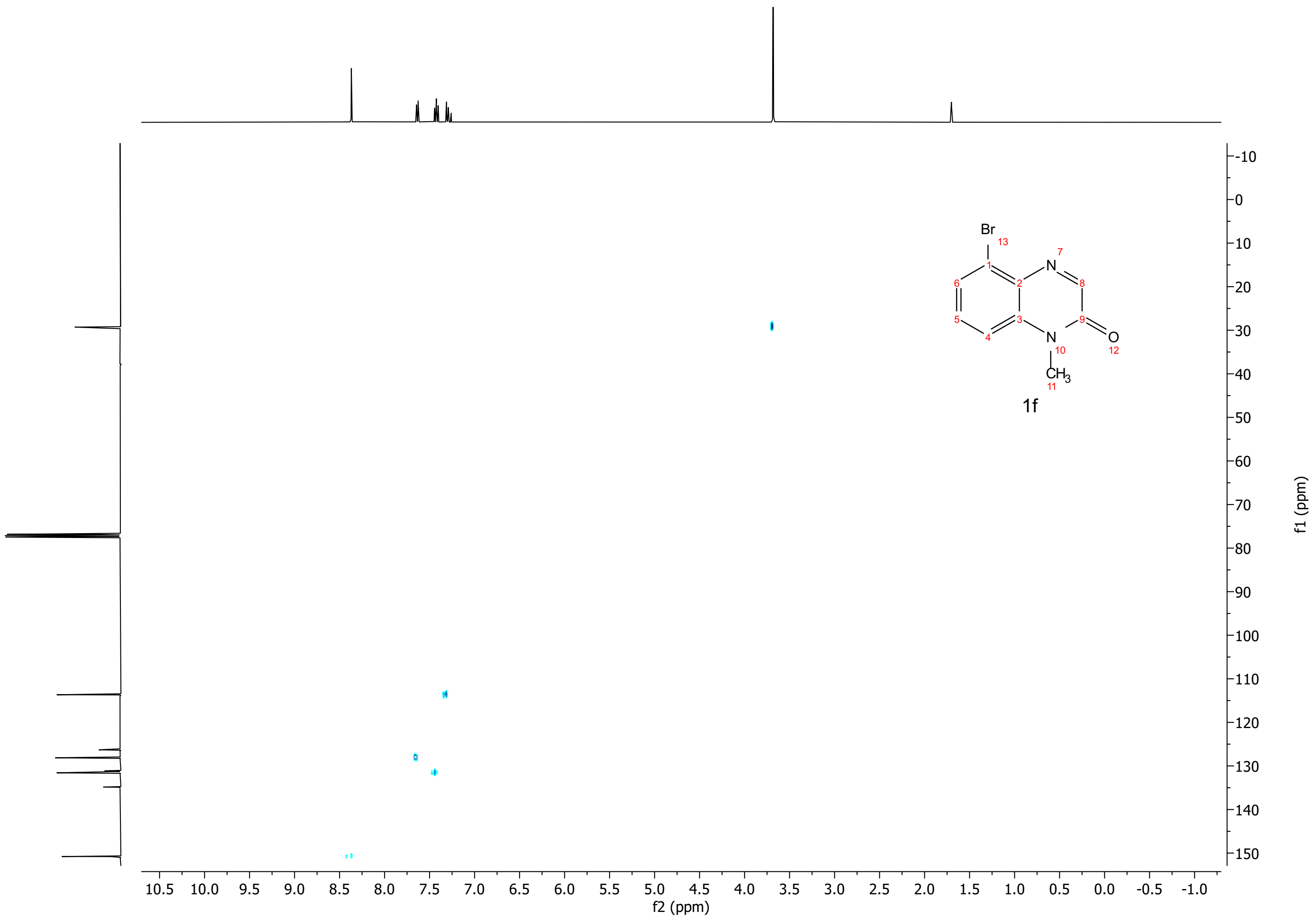

Figure S.28: gHSQC spectrum ($CDCl_3$) of 5-bromo-N-methylquinoxalin-2(1H)-one, **1f**.

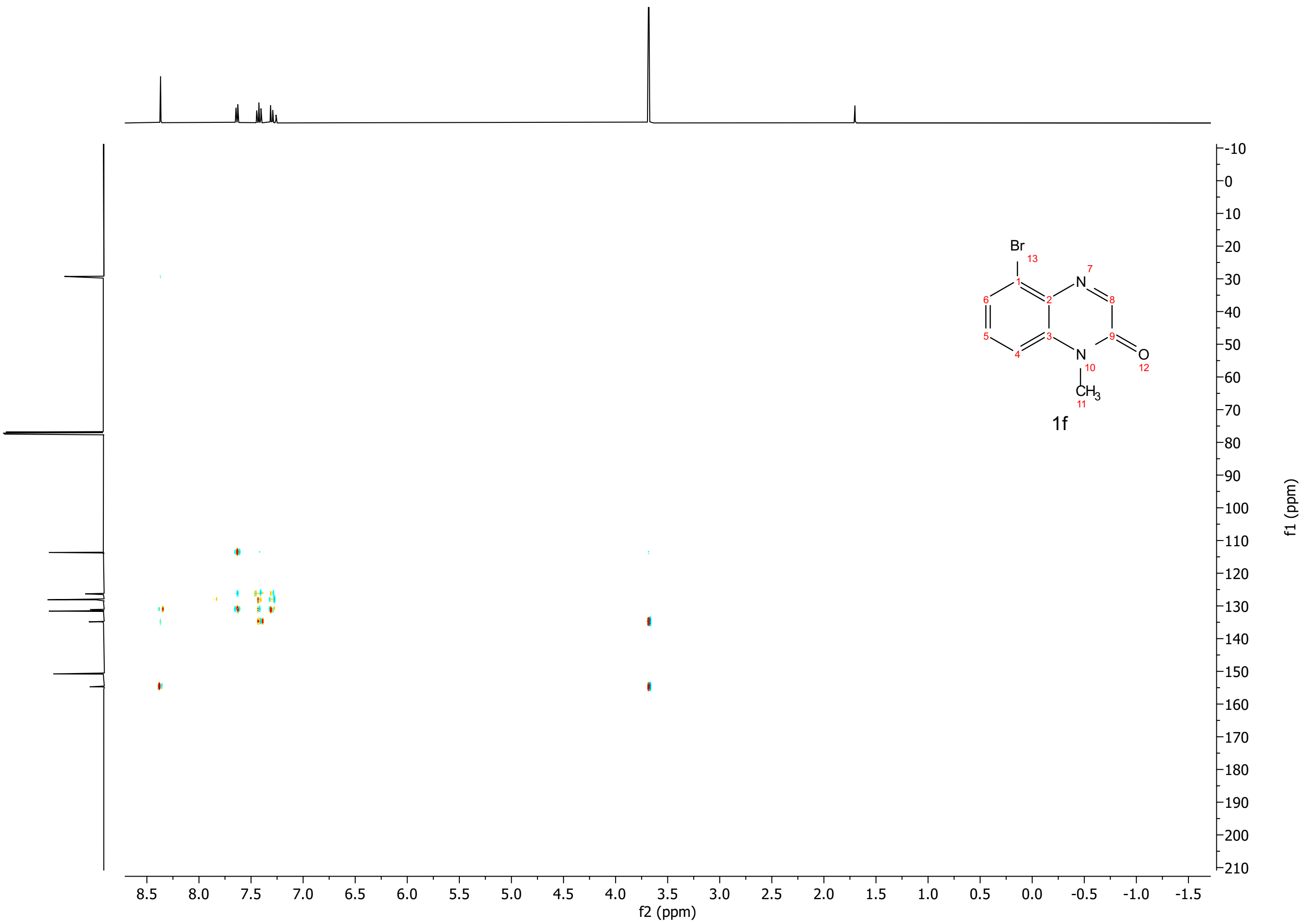

Figure S.29: gHMBC spectrum ($CDCl_3$) of 5-bromo-N-methylquinoxalin-2(1H)-one, **1f**.

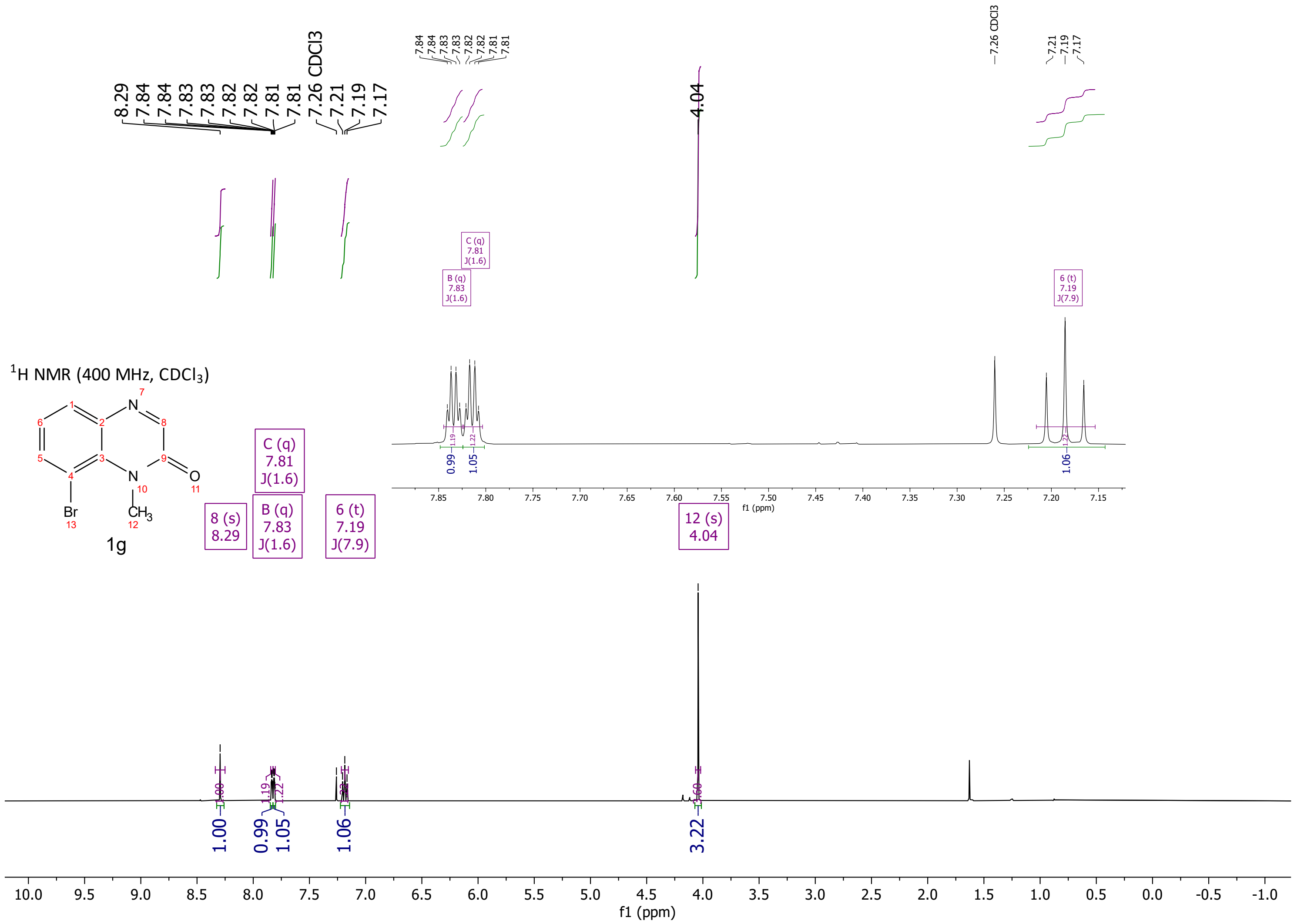

Figure S.30: ^{1}H NMR spectrum ($CDCl_3$, 400 MHz) of 8-bromo-1-methylquinoxalin-2(1H)-one, **1g**.

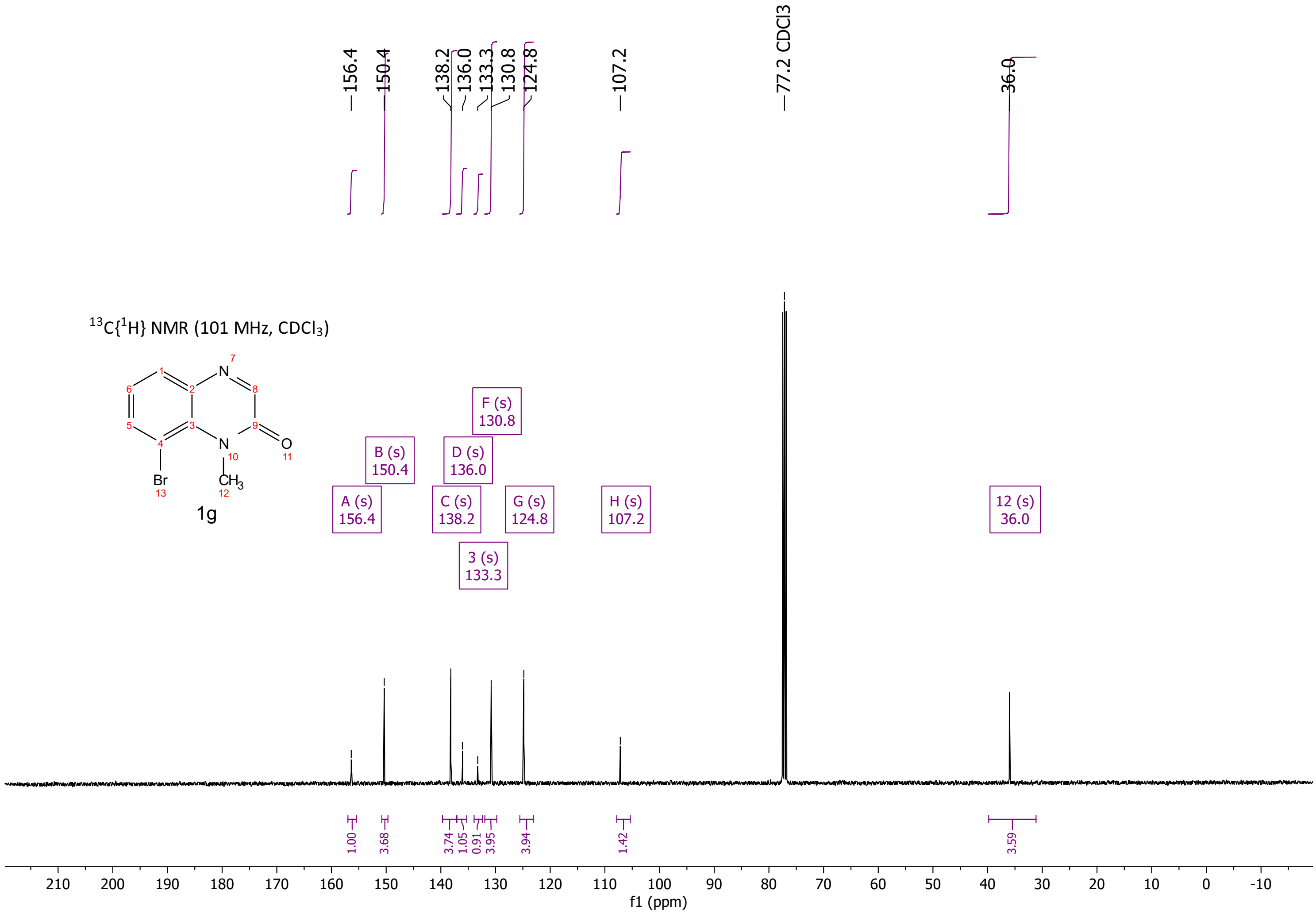

Figure S.31: $^{13}C\{^{1}H\}$ NMR spectrum ($CDCl_3$, 101 MHz) of 8-bromo-1-methylquinoxalin-2(1H)-one, **1f**.

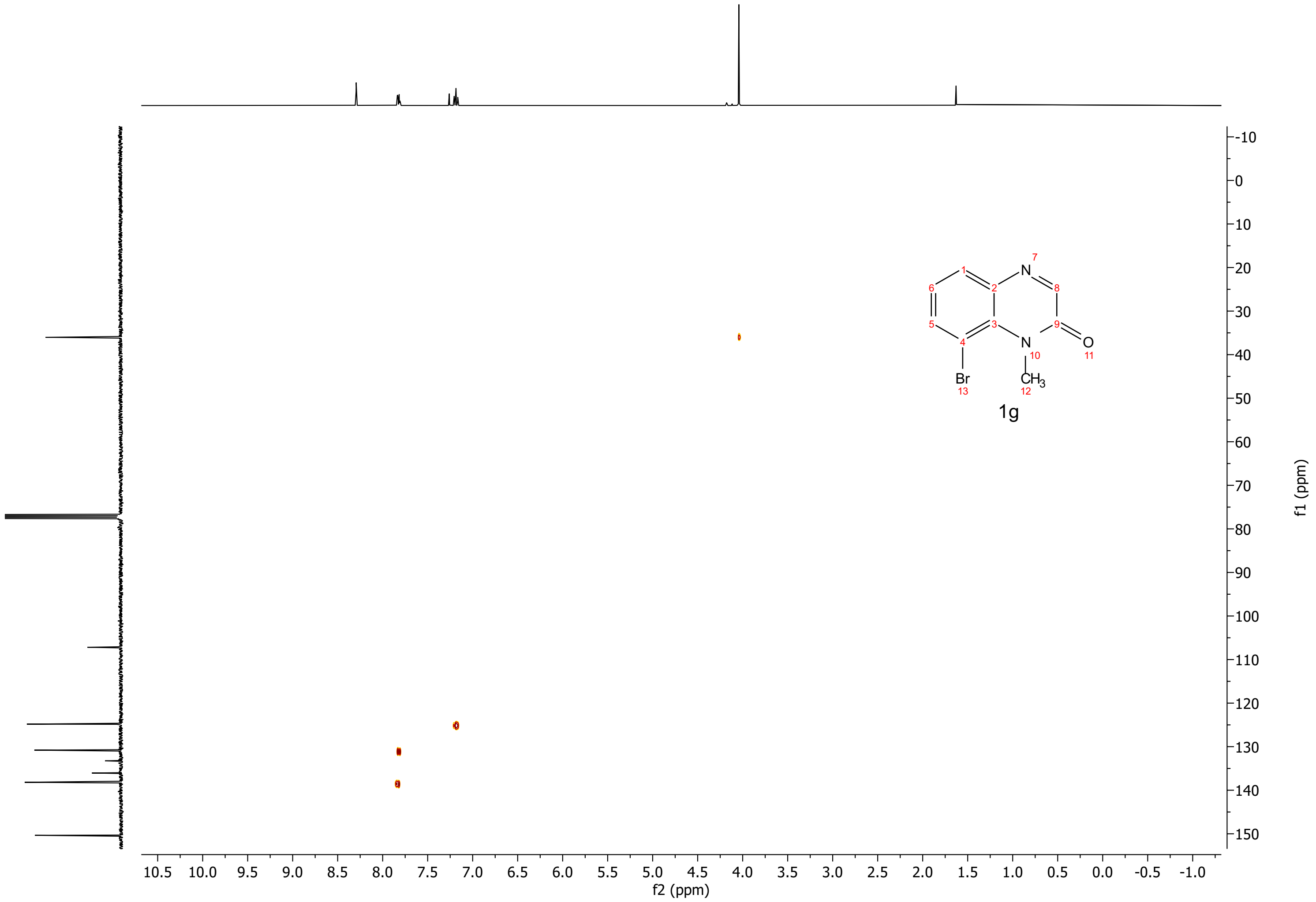

Figure S.32: gHSQC spectrum ($CDCl_3$) of 8-bromo-1-methylquinoxalin-2(1H)-one, **1g**.

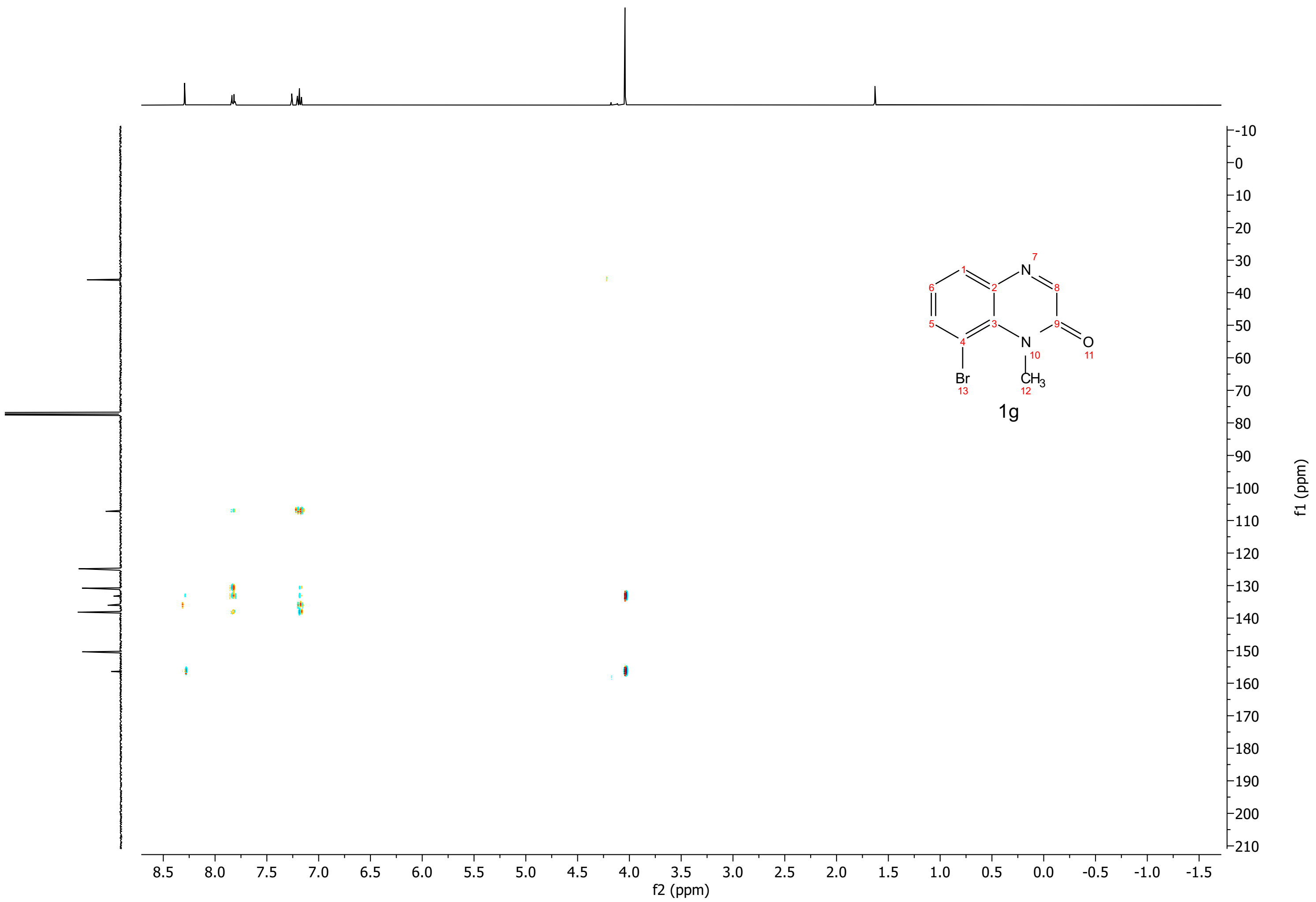

Figure S.33: gHMBC spectrum ($CDCl_3$) of 8-bromo-1-methylquinoxalin-2(1H)-one, **1g**.

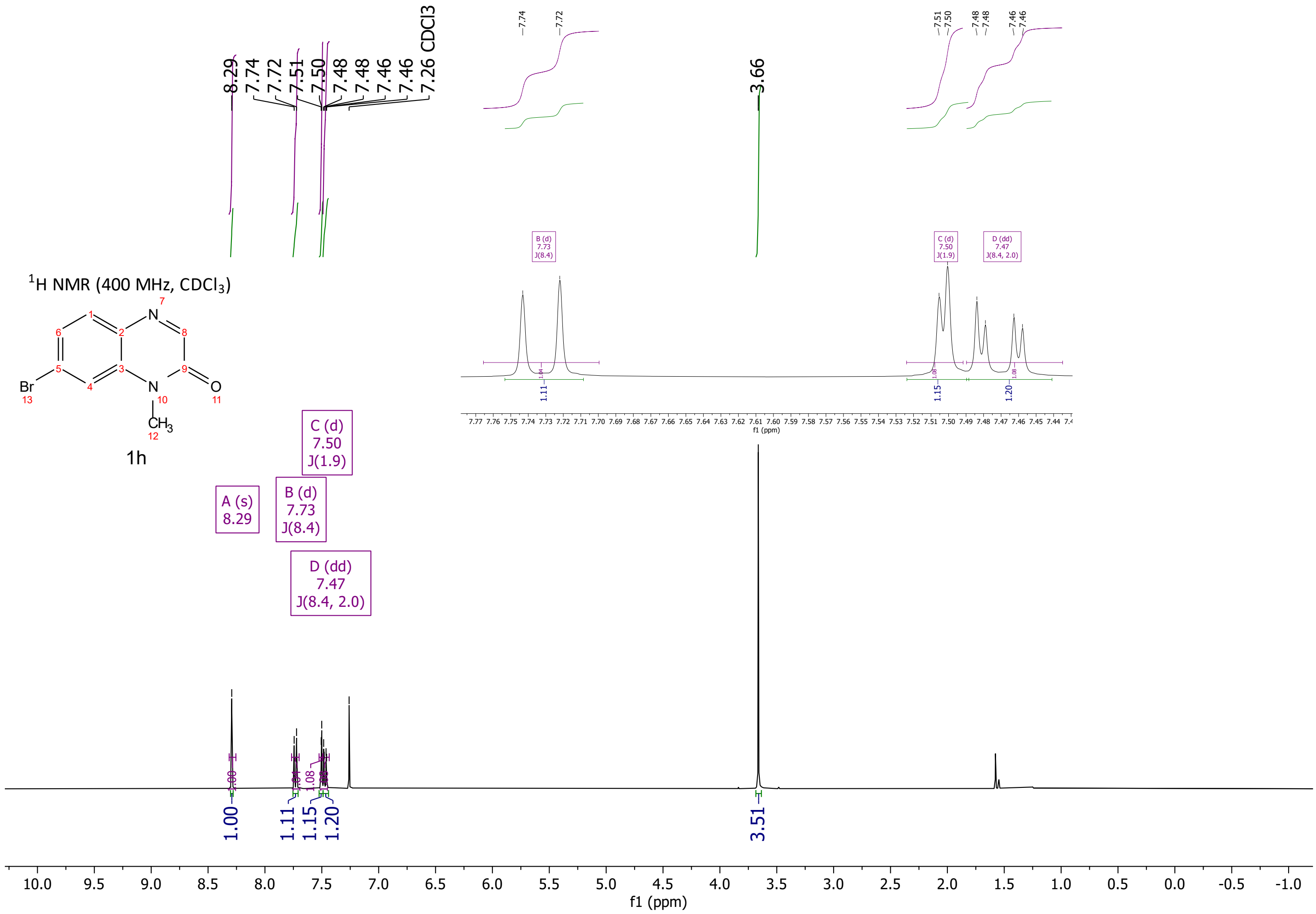

Figure S.34: ^{1}H NMR spectrum ($CDCl_3$, 400 MHz) of 7-bromo-1-methylquinoxalin-2(1H)-one, **1h**.

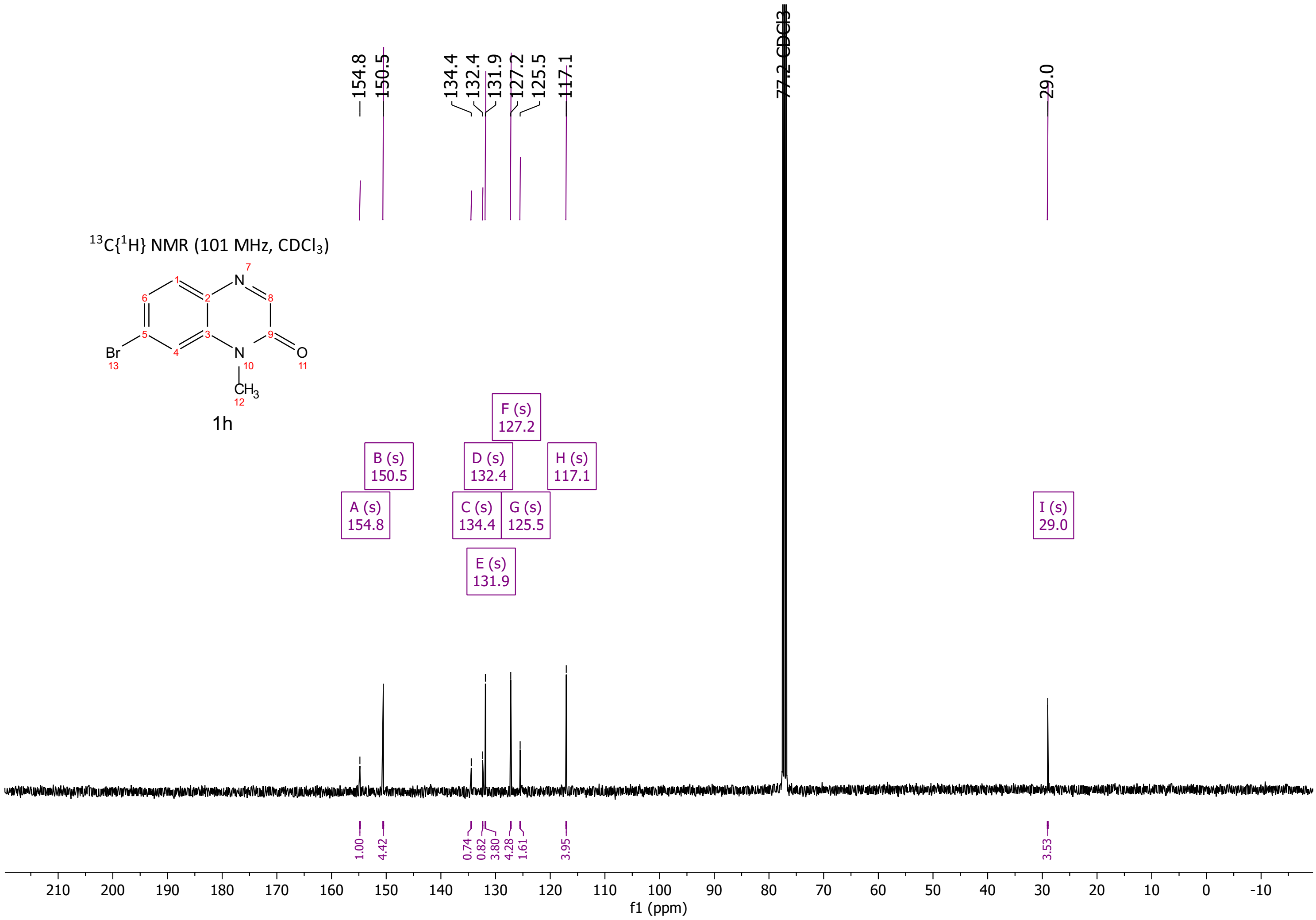

Figure S.35: $^{13}C\{^1H\}$ NMR spectrum ($CDCl_3$, 101 MHz) of 7-bromo-1-methylquinoxalin-2(1H)-one, **1h**.

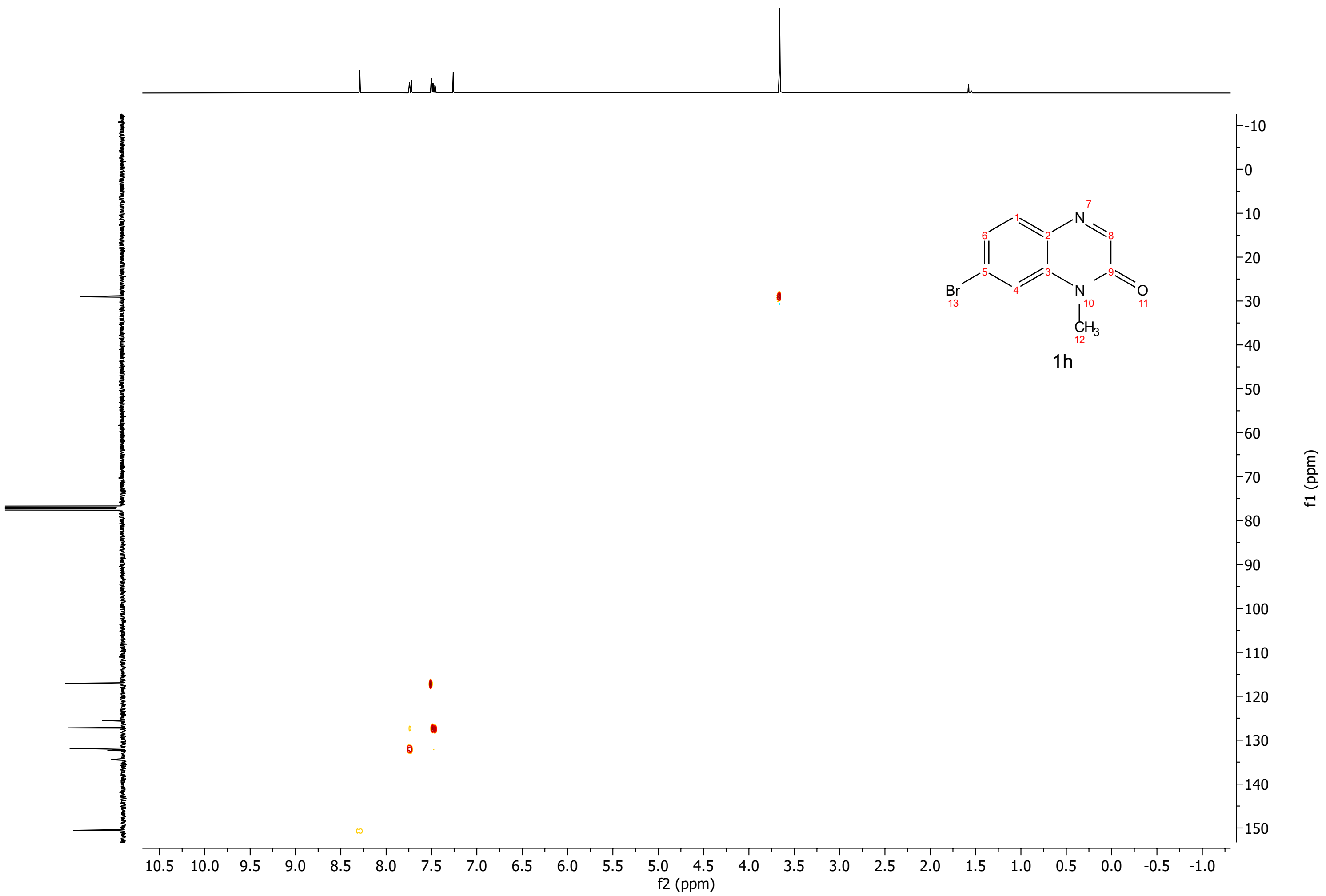

Figure S.36: gHSQC spectrum ($CDCl_3$) of 7-bromo-1-methylquinoxalin-2(1H)-one, **1h**.

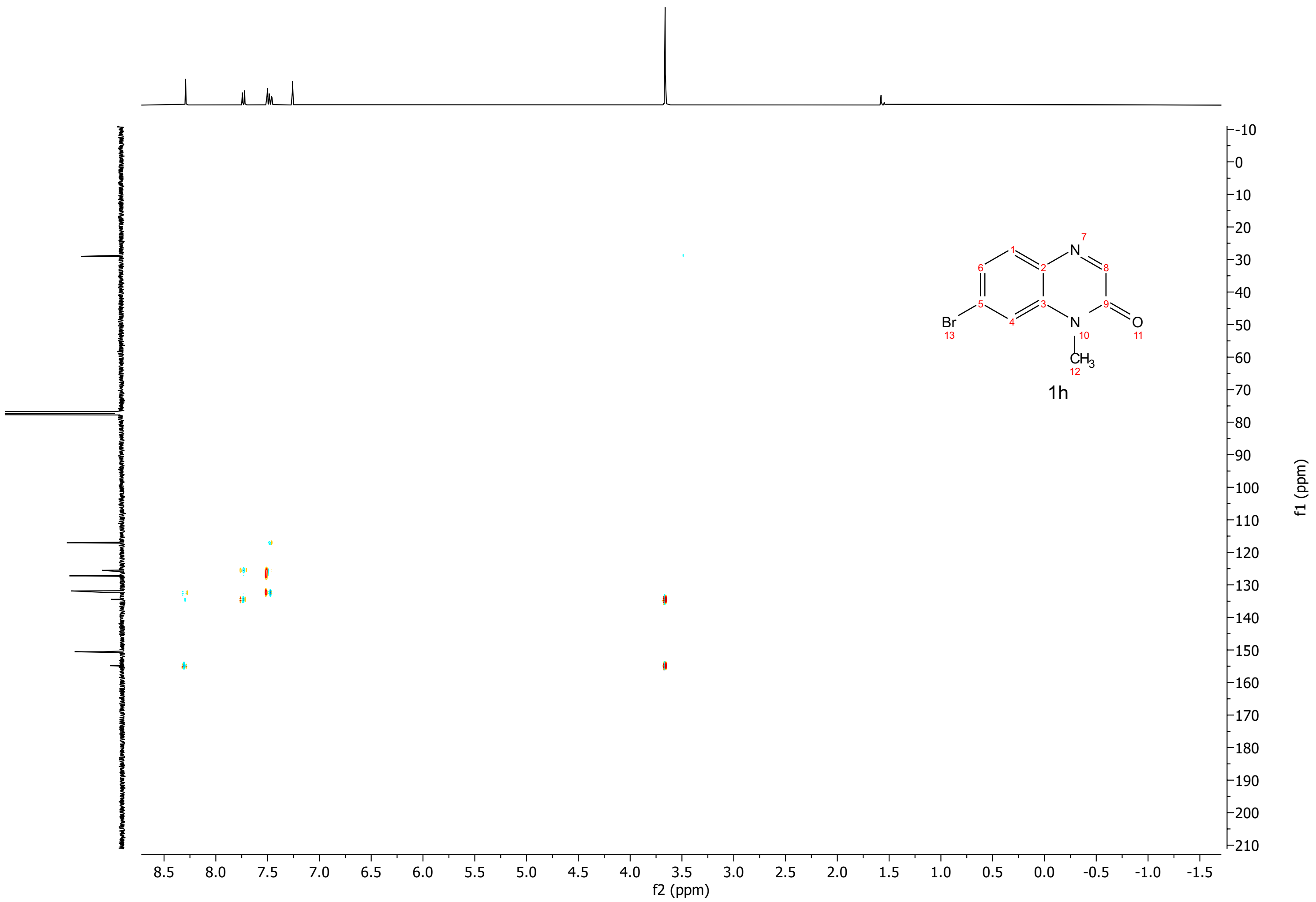

Figure S.37: gHMBC spectrum ($CDCl_3$) of 7-bromo-1-methylquinoxalin-2(1H)-one, **1h**.

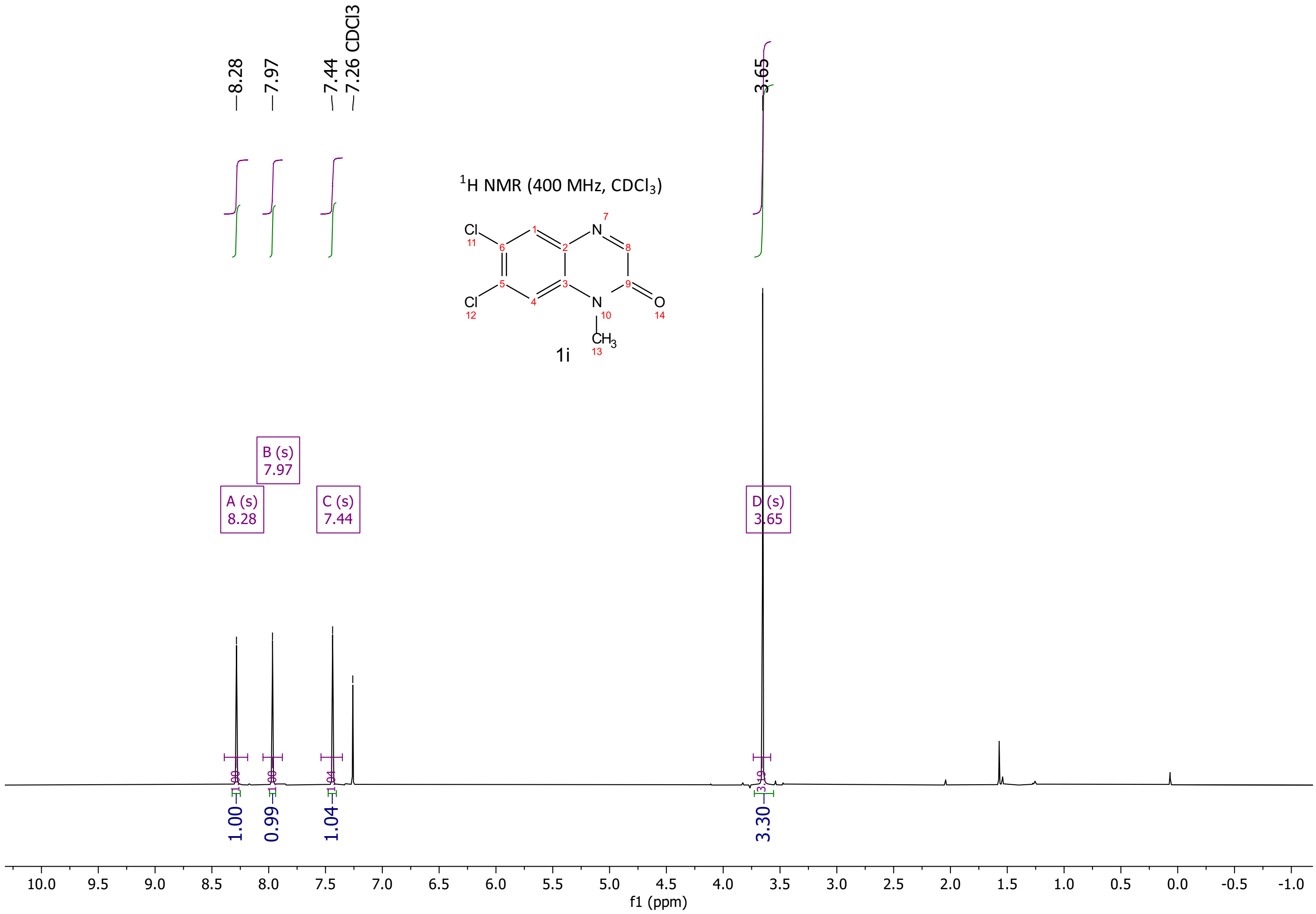

Figure S.38: ^{1}H NMR spectrum ($CDCl_3$, 400 MHz) of 6,7-dichloro-1-methylquinoxalin-2(1H)-one, **1i**.

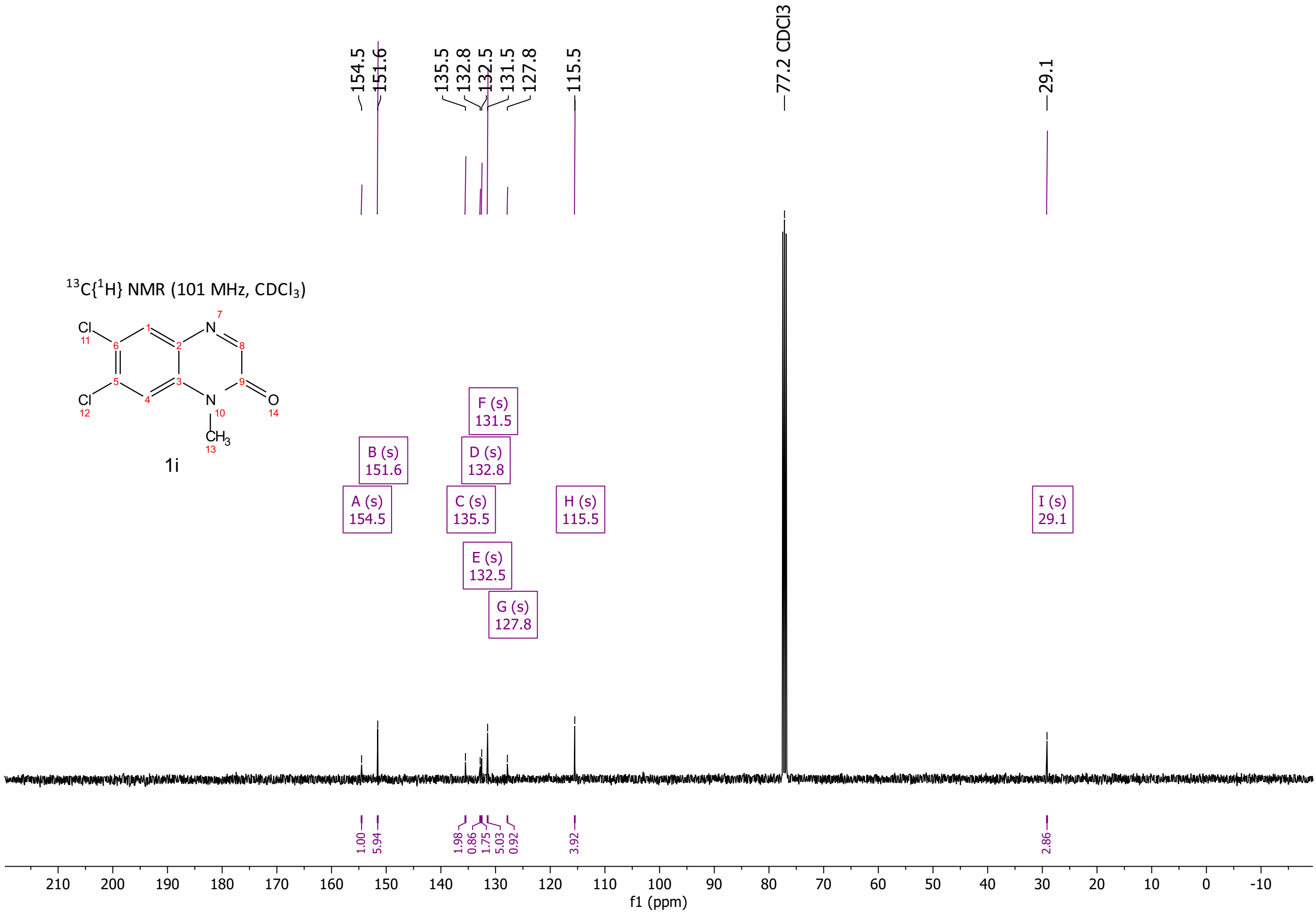

Figure S.39: ^{13}C{^{1}H} NMR spectrum ($CDCl_3$, 101 MHz) of 6,7-dichloro-1-methylquinoxalin-2(1H)-one, **1i**.

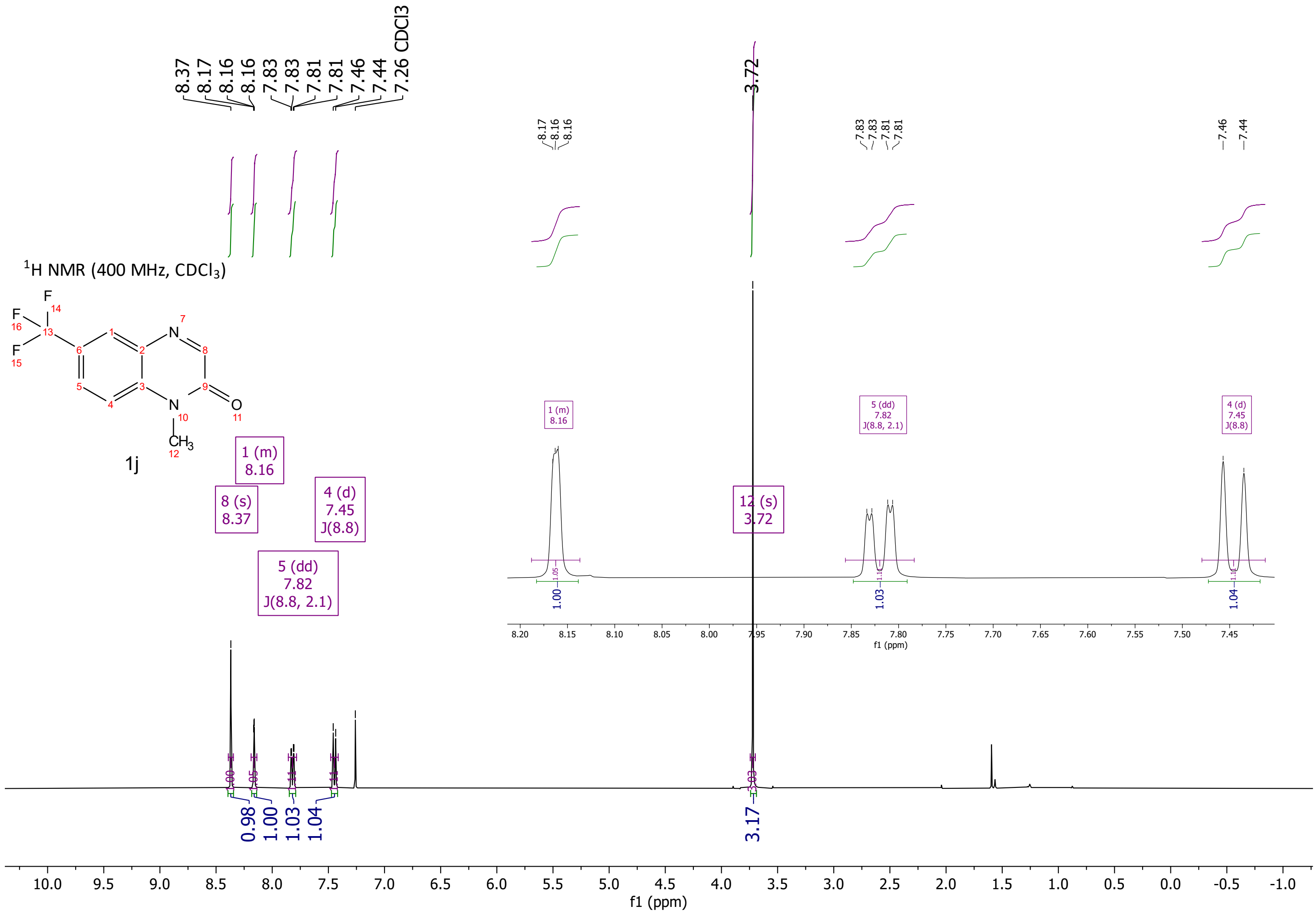

Figure S.40: ^{1}H NMR spectrum (CDCl$_3$, 400 MHz) of 1-Methyl-6-(trifluoromethyl)-2(1H)-quinoxalinone, **1j**.

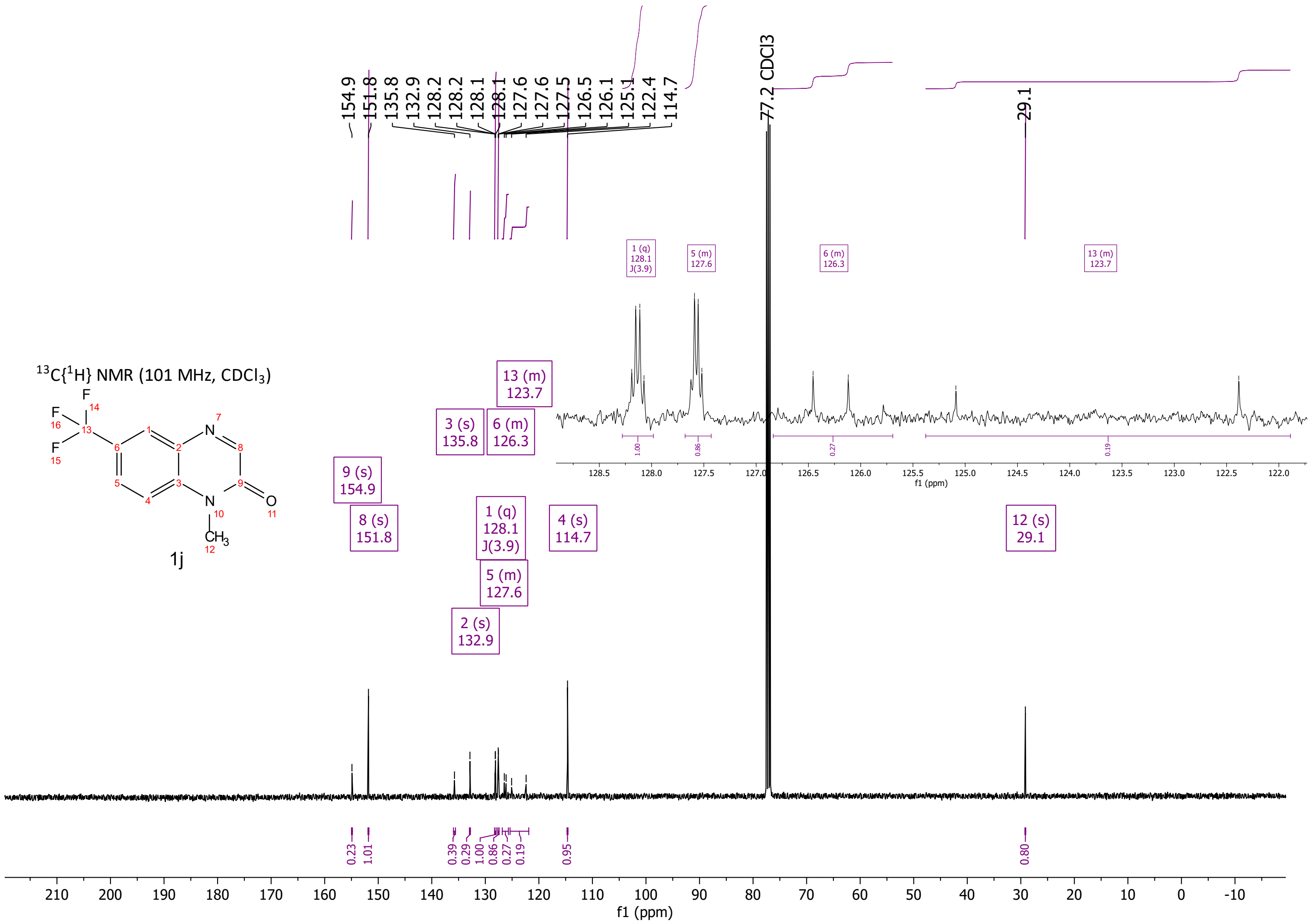

Figure S.41: $^{13}C\{^1H\}$ NMR spectrum ($CDCl_3$, 101 MHz) of 1-Methyl-6-(trifluoromethyl)-2(1H)-quinoxalinone, **1j**.

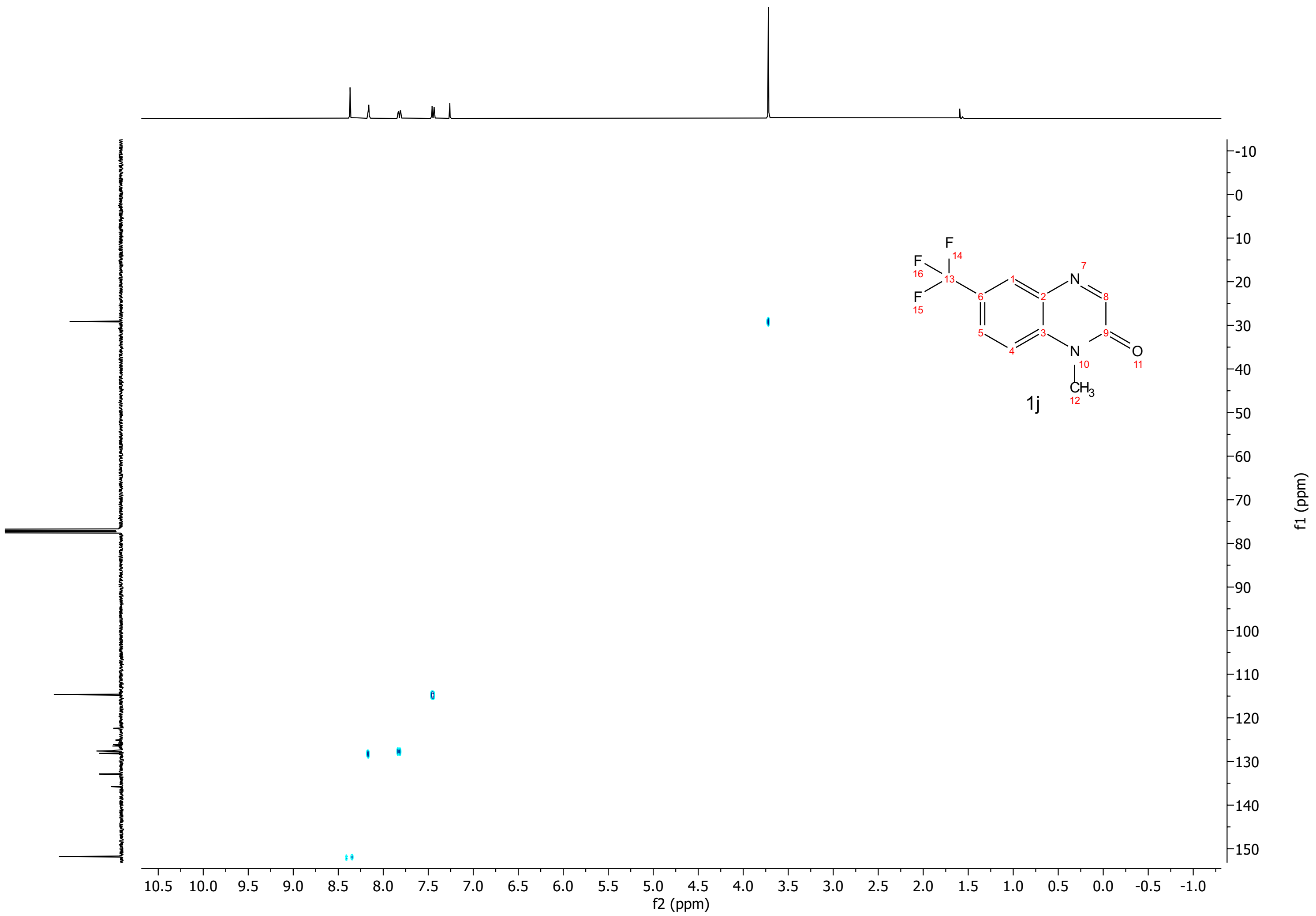

Figure S.42: gHSQC spectrum ($CDCl_3$) of 1-Methyl-6-(trifluoromethyl)-2(1H)-quinoxalinone, **1j**.

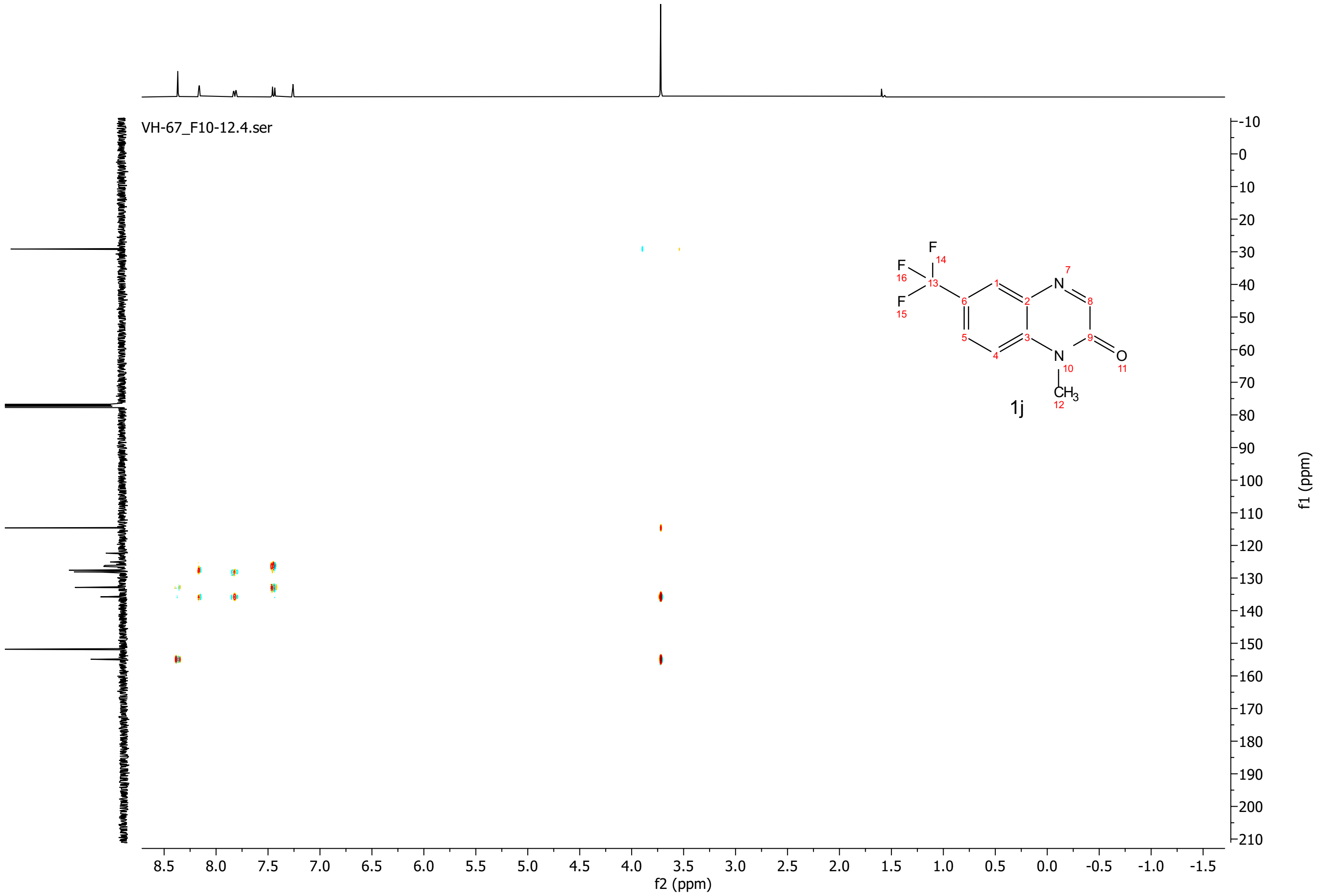

Figure S.43: gHMBC spectrum ($CDCl_3$) of 1-Methyl-6-(trifluoromethyl)-2(1H)-quinoxalinone, **1j**.

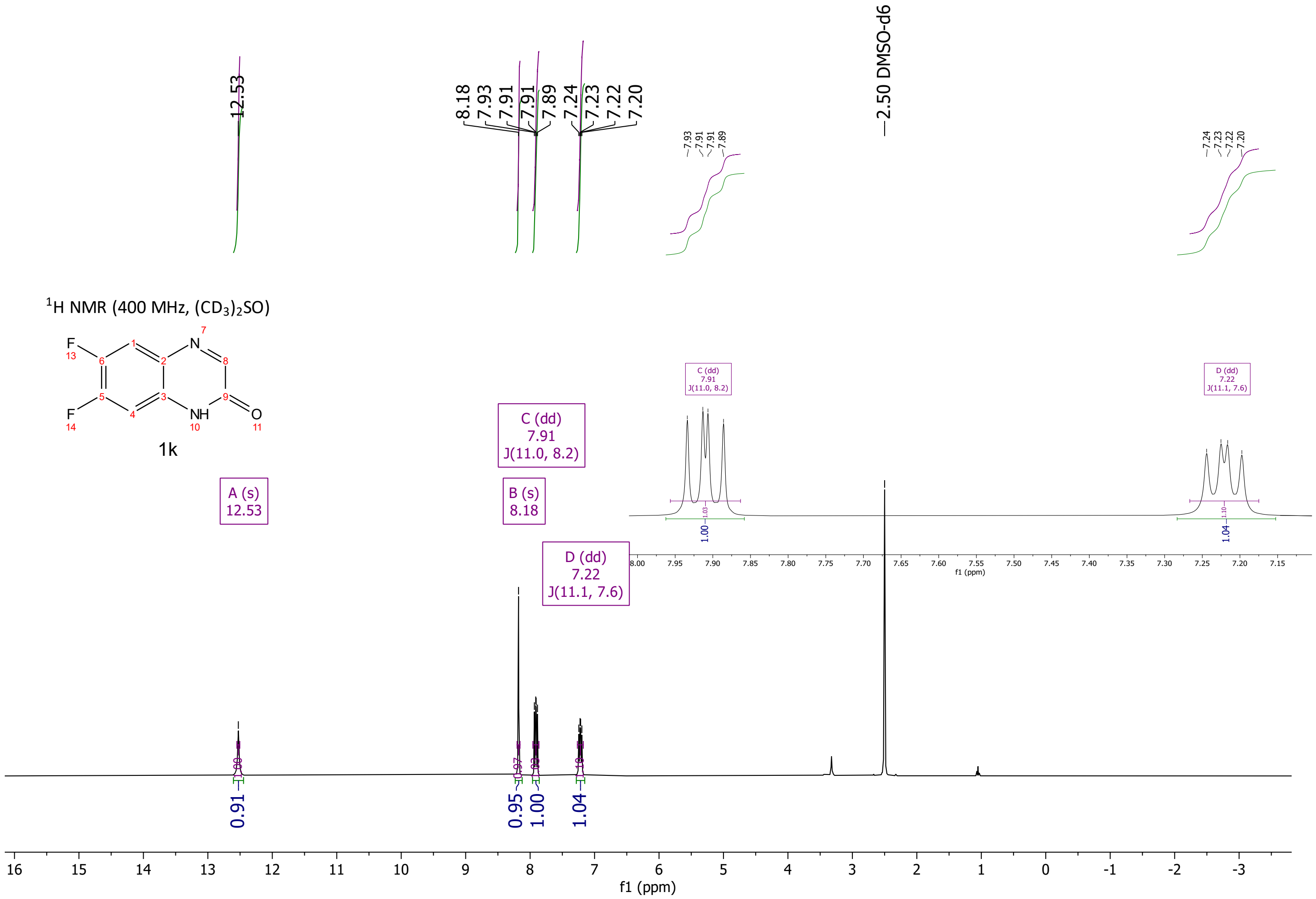

Figure S.44: ^{1}H NMR spectrum ($(CD_3)_2SO$, 400 MHz) of 6,7-difluoroquinoxalin-2(1H)-one, **1k**.

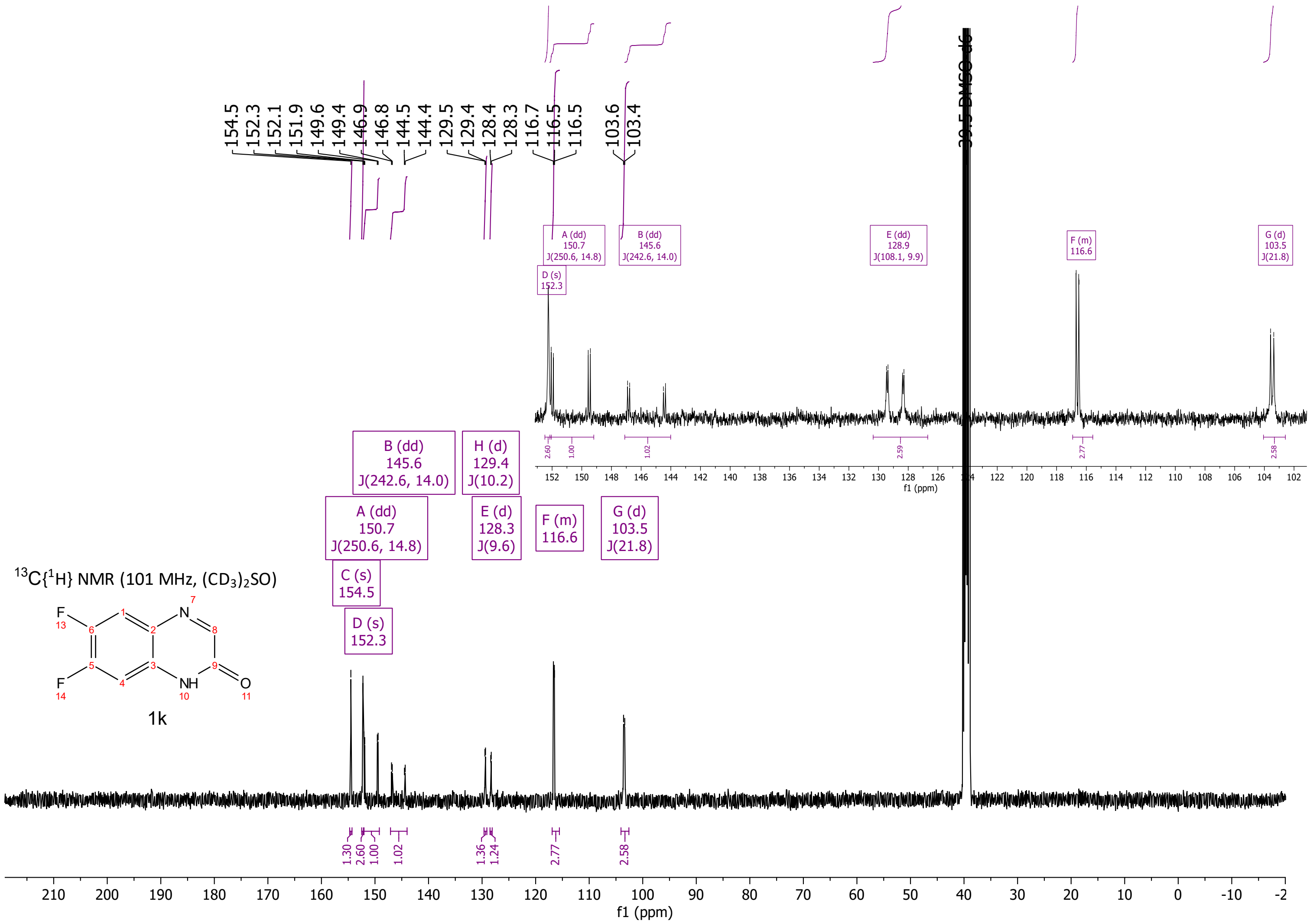

Figure S.45: $^{13}C\{^1H\}$ NMR spectrum ($(CD_3)_2SO$, 101 MHz) of 6,7-difluoroquinoxalin-2(1H)-one, **1k**.

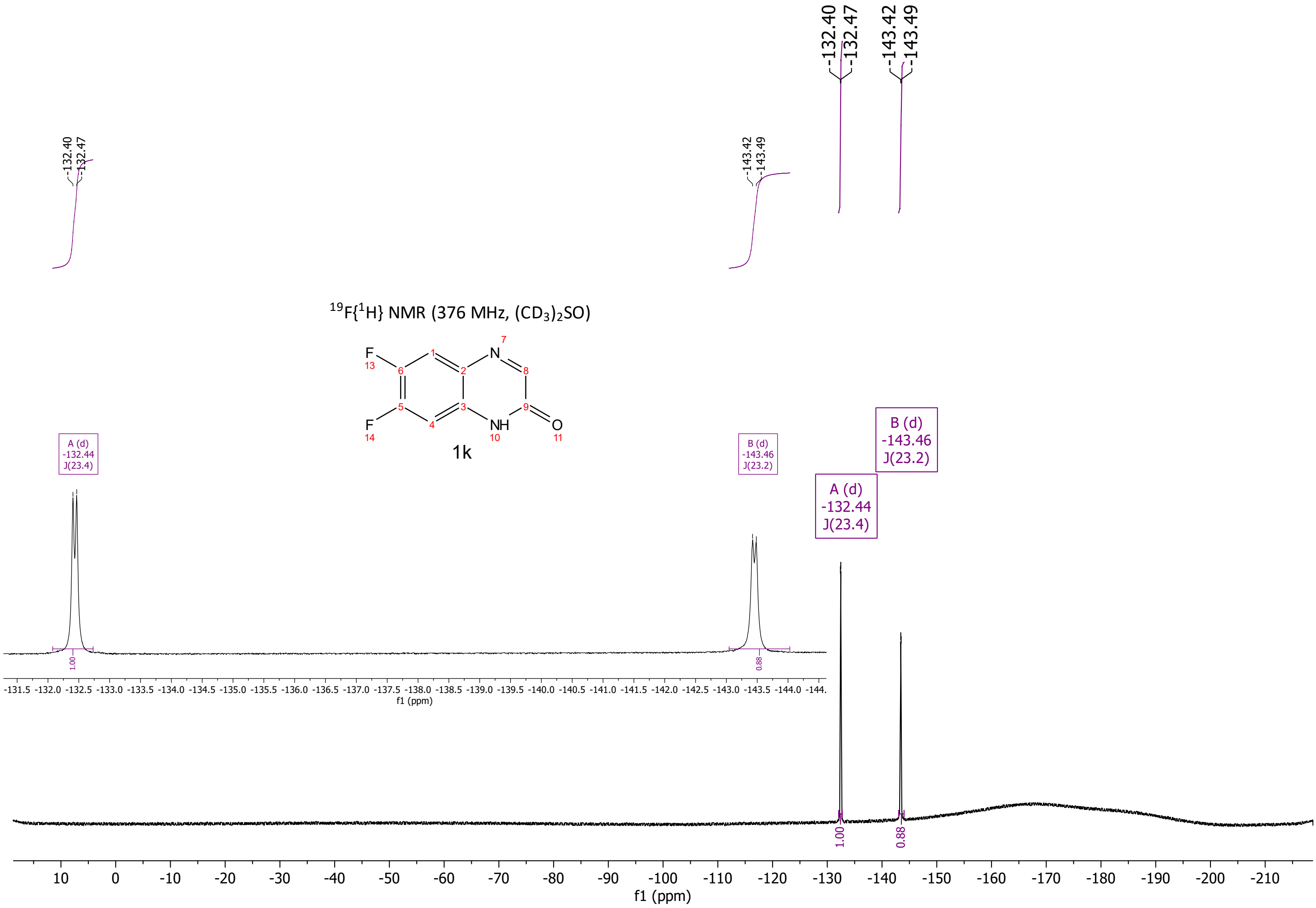

Figure S.46: ^{19}F{^{1}H} NMR spectrum ($(CD_3)_2SO$, 376 MHz) of 6,7-difluoroquinoxalin-2(1H)-one, **1k**.

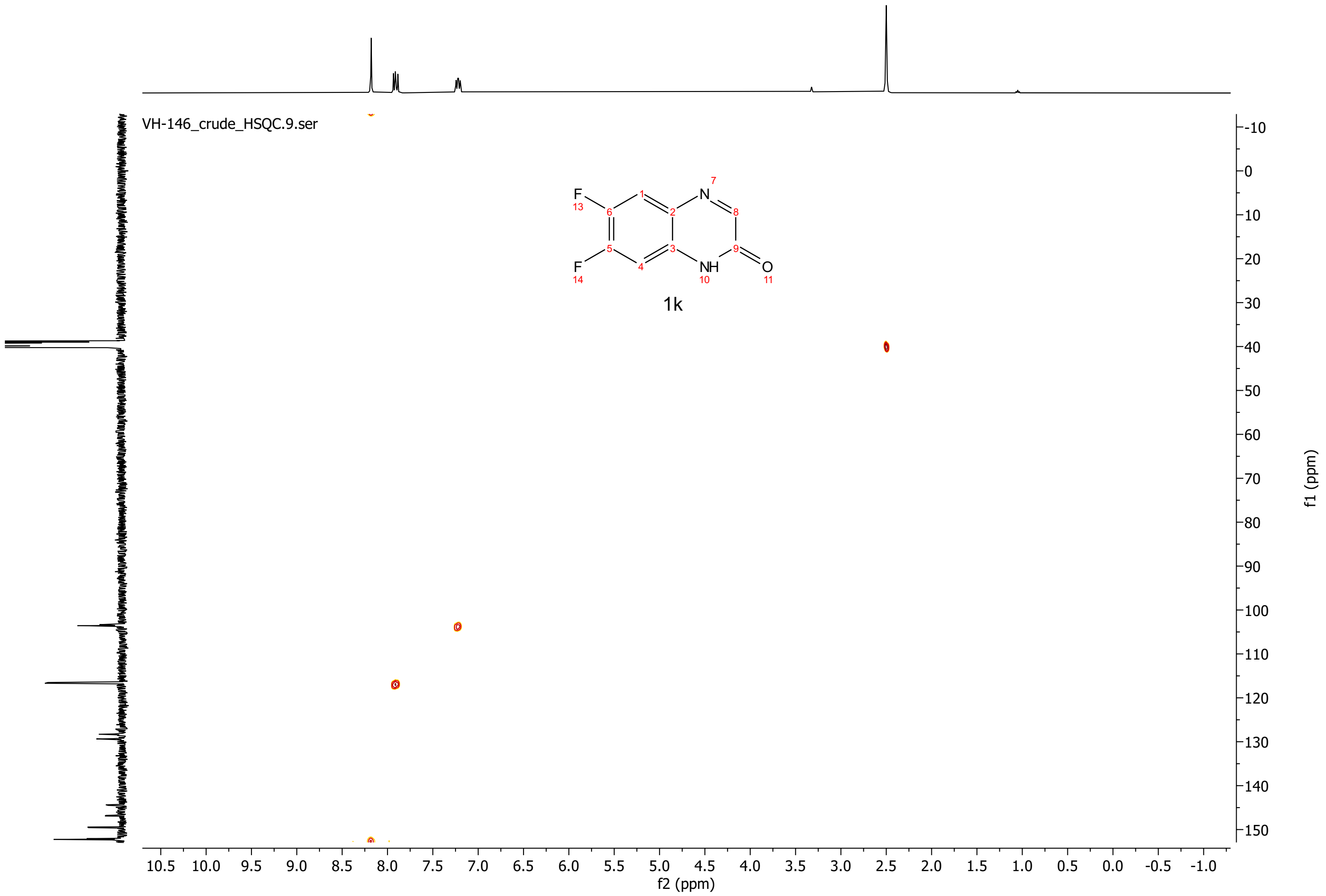

Figure S.47: gHSQC spectrum ($(CD_3)_2SO$) of 6,7-difluoroquinoxalin-2(1H)-one, **1k**.

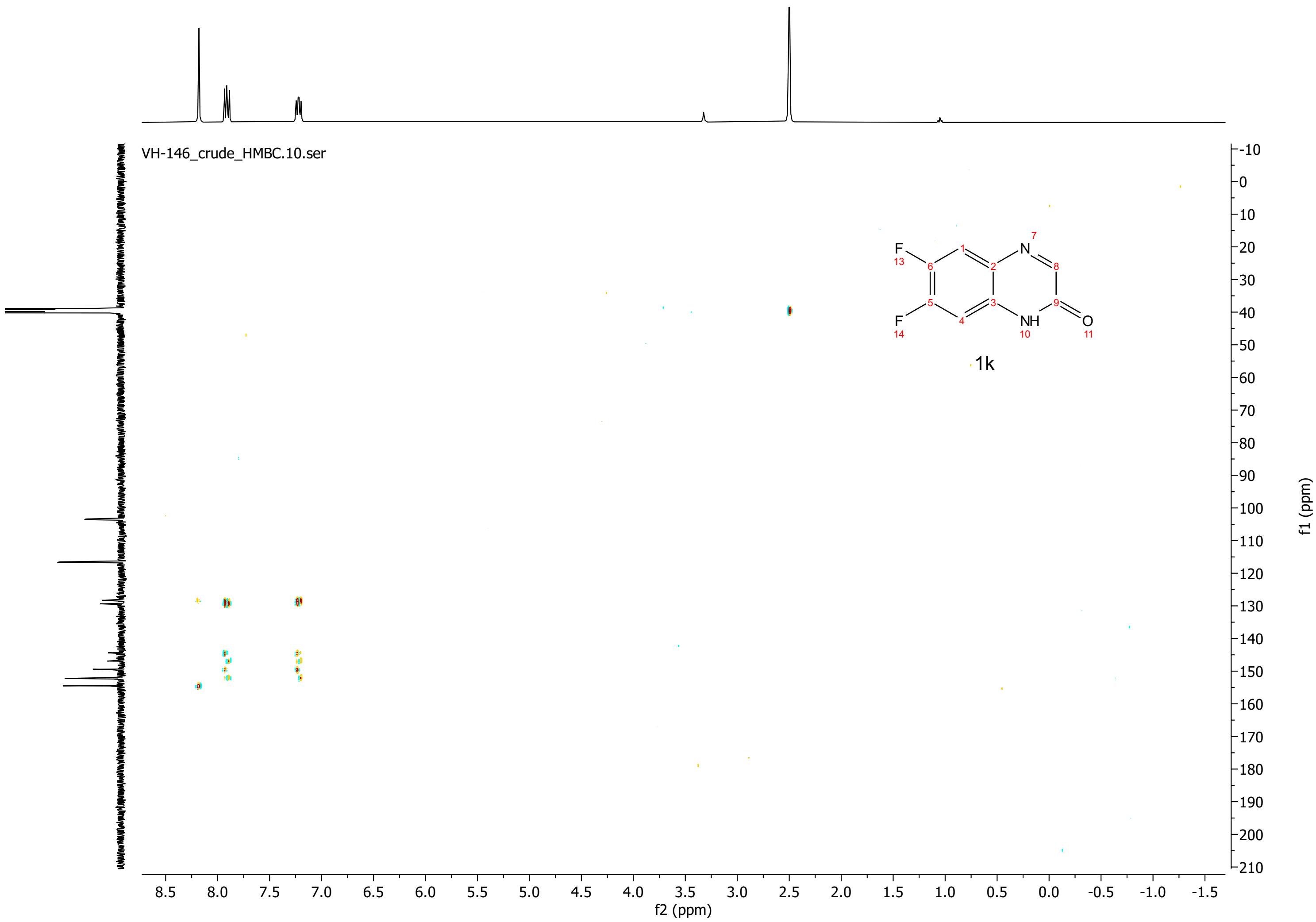

Figure S.48: gHMBC spectrum ($(CD_3)_2SO$) of 6,7-difluoroquinoxalin-2(1H)-one, **1k**.

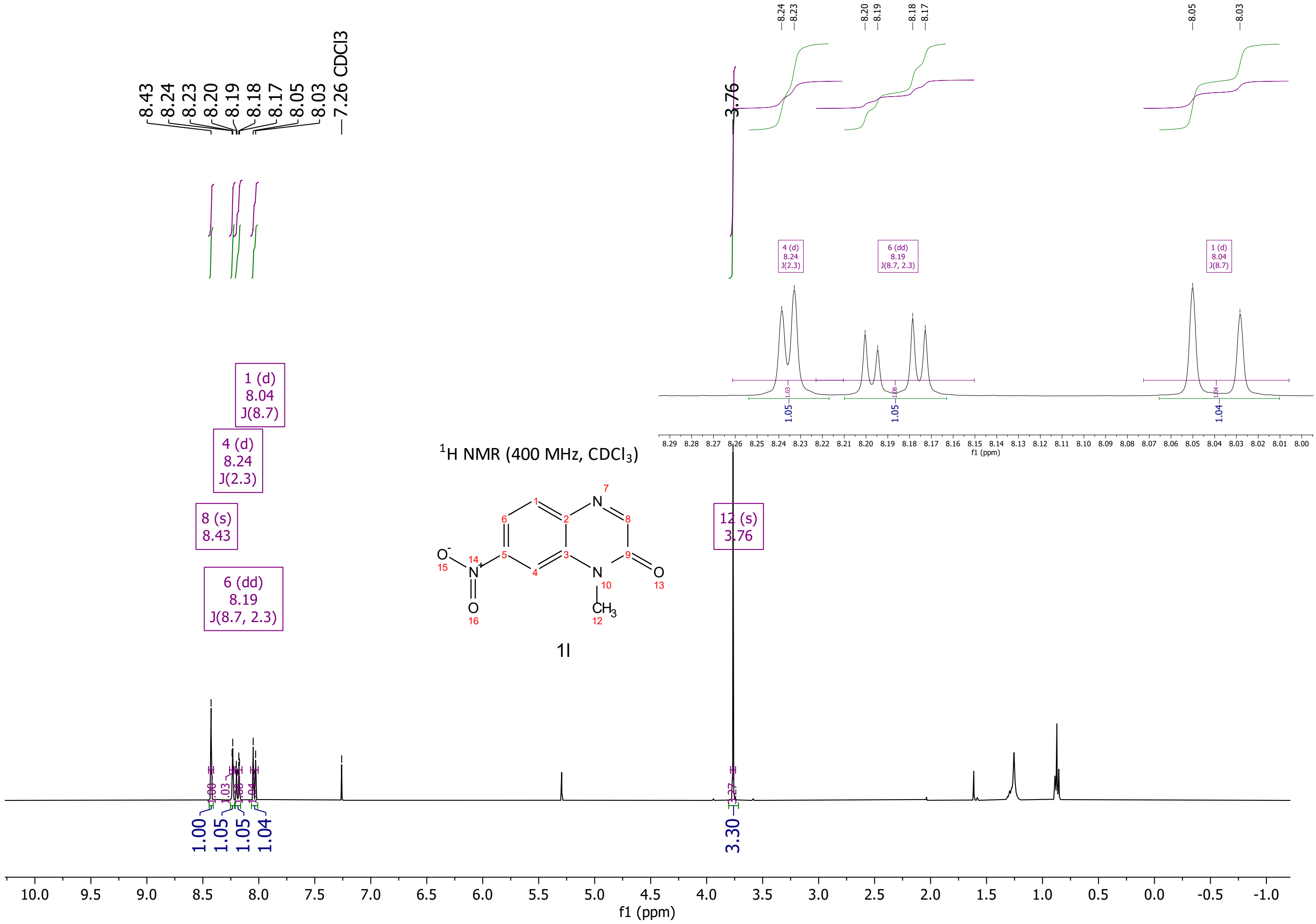

Figure S.49: ^{1}H NMR spectrum ($CDCl_3$, 400 MHz) of 1-methyl-7-nitroquinoxalin-2(1H)-one, **1l**.

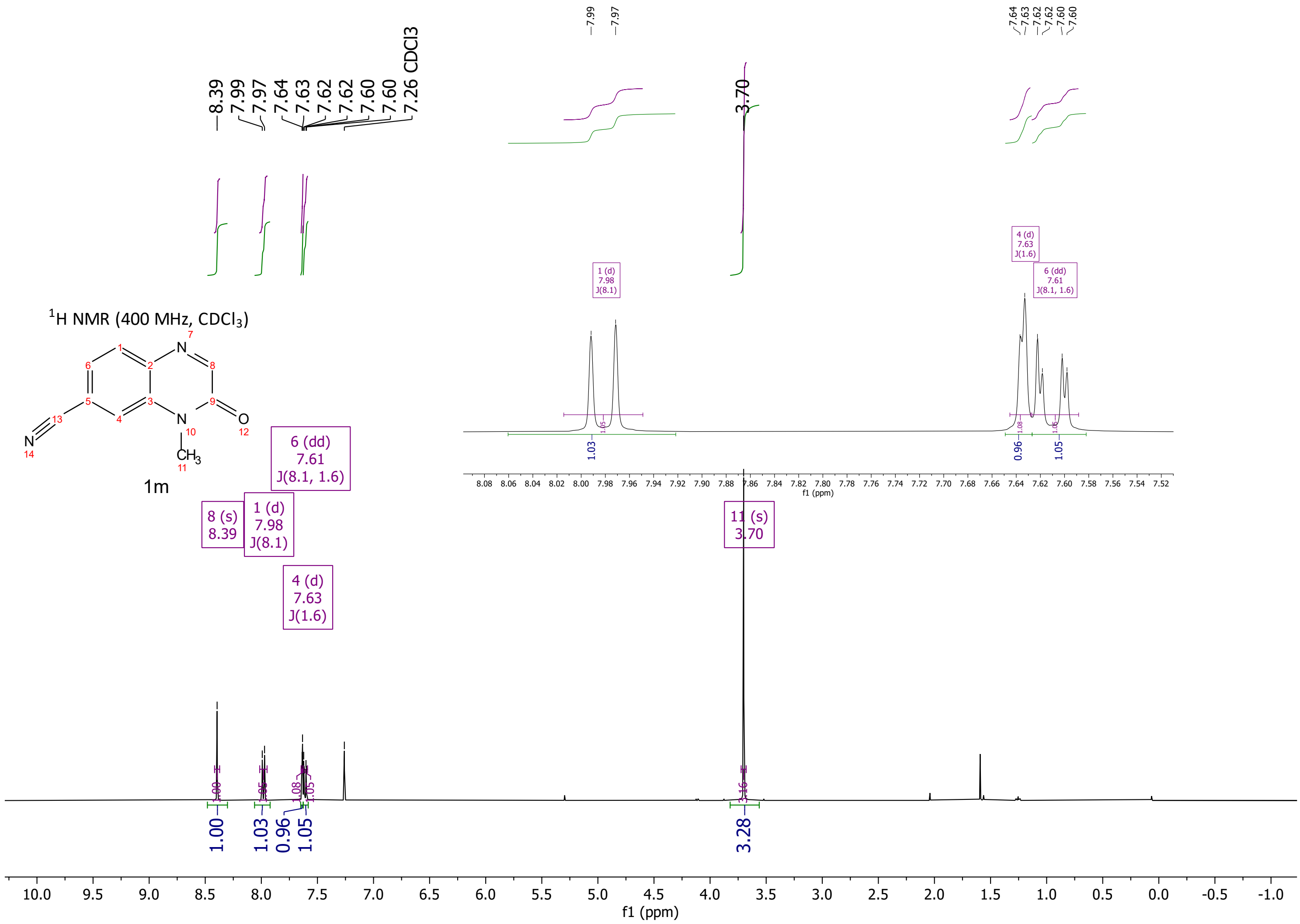

Figure S.50: ^{1}H NMR spectrum ($CDCl_3$, 400 MHz) of 4-methyl-3-oxo-3,4-dihydroquinoxaline-6-carbonitrile, **1m**.

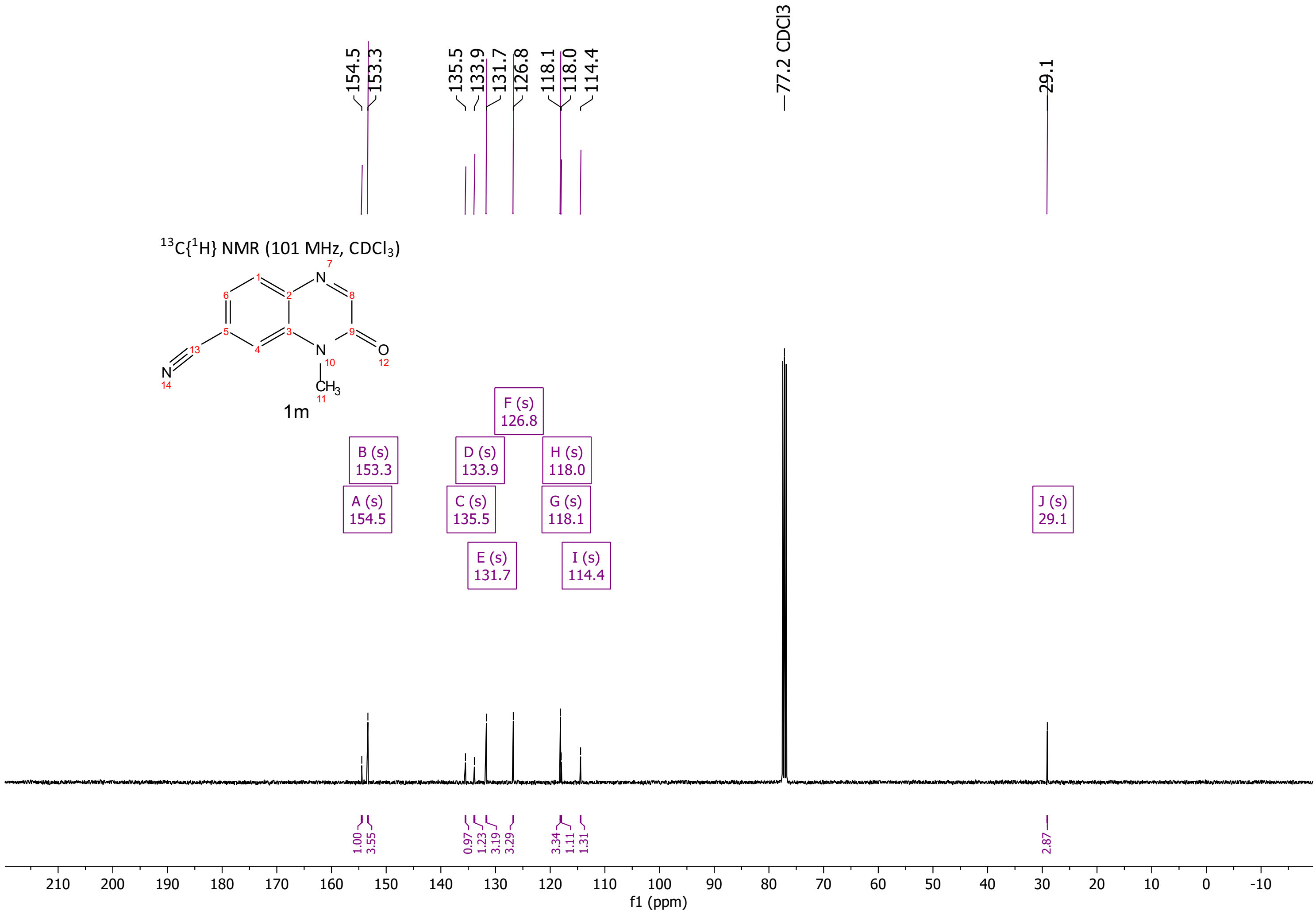

Figure S.51: $^{13}C\{^1H\}$ NMR spectrum ($CDCl_3$, 101 MHz) of 4-methyl-3-oxo-3,4-dihydroquinoxaline-6-carbonitrile, **1m**.

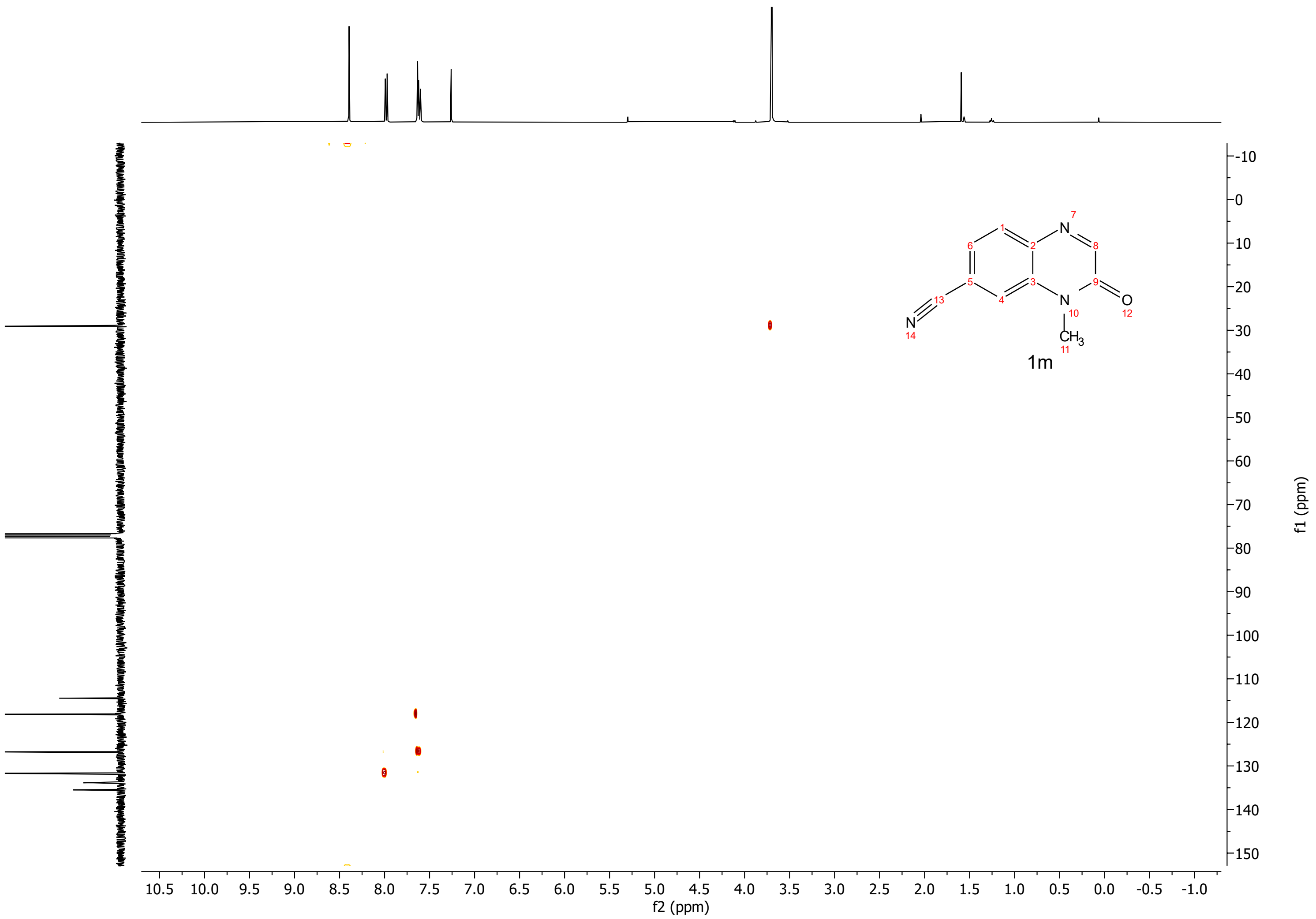

Figure S.52: gHSQC spectrum ($CDCl_3$) of 4-methyl-3-oxo-3,4-dihydroquinoxaline-6-carbonitrile, **1m**.

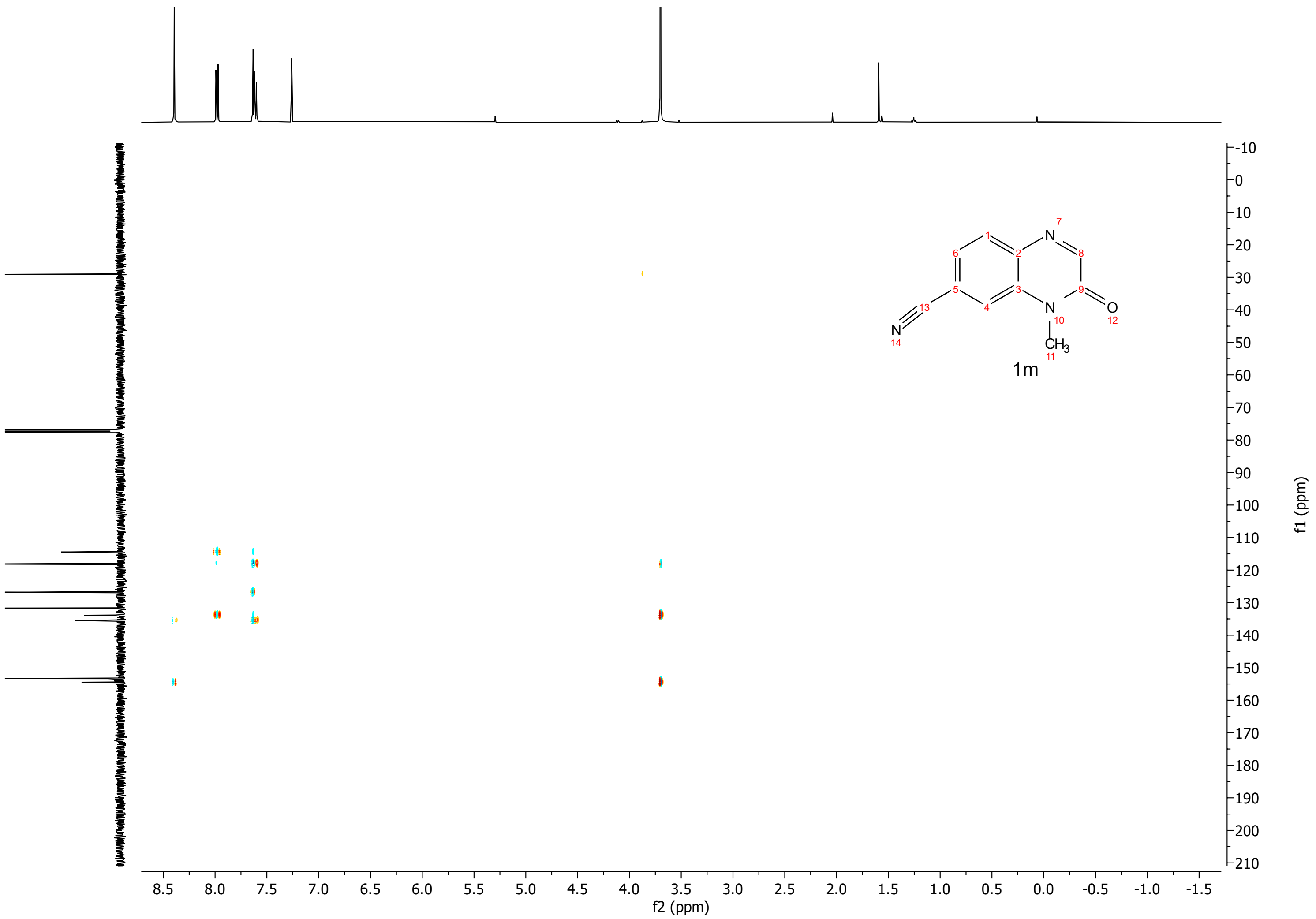

Figure S.53: gHMBC spectrum ($CDCl_3$) of 4-methyl-3-oxo-3,4-dihydroquinoxaline-6-carbonitrile, **1m**.

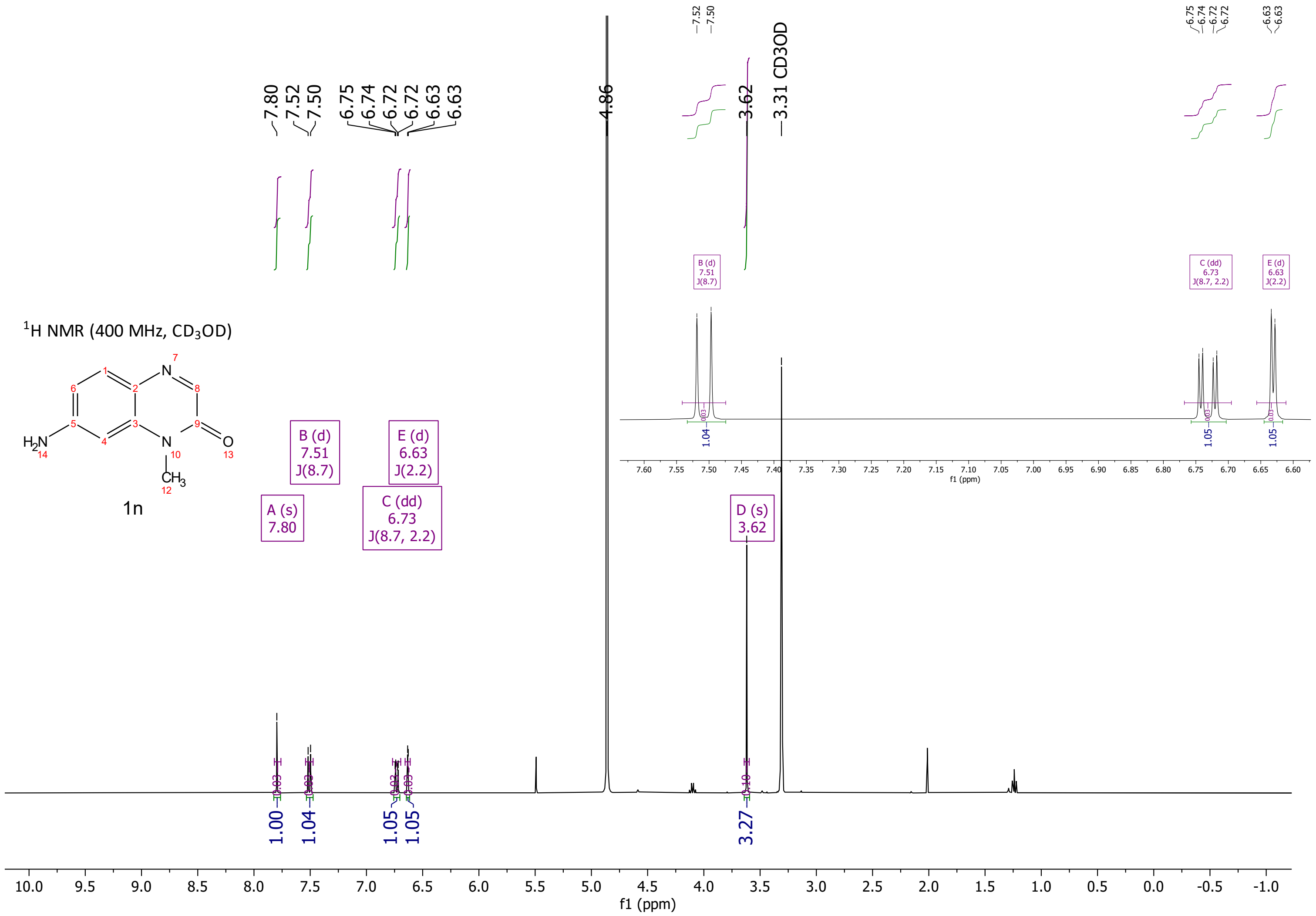

Figure S.54: ^{1}H NMR spectrum (CD_3OD, 400 MHz) of 7-amino-1-methylquinoxalin-2(1H)-one, **1n**.

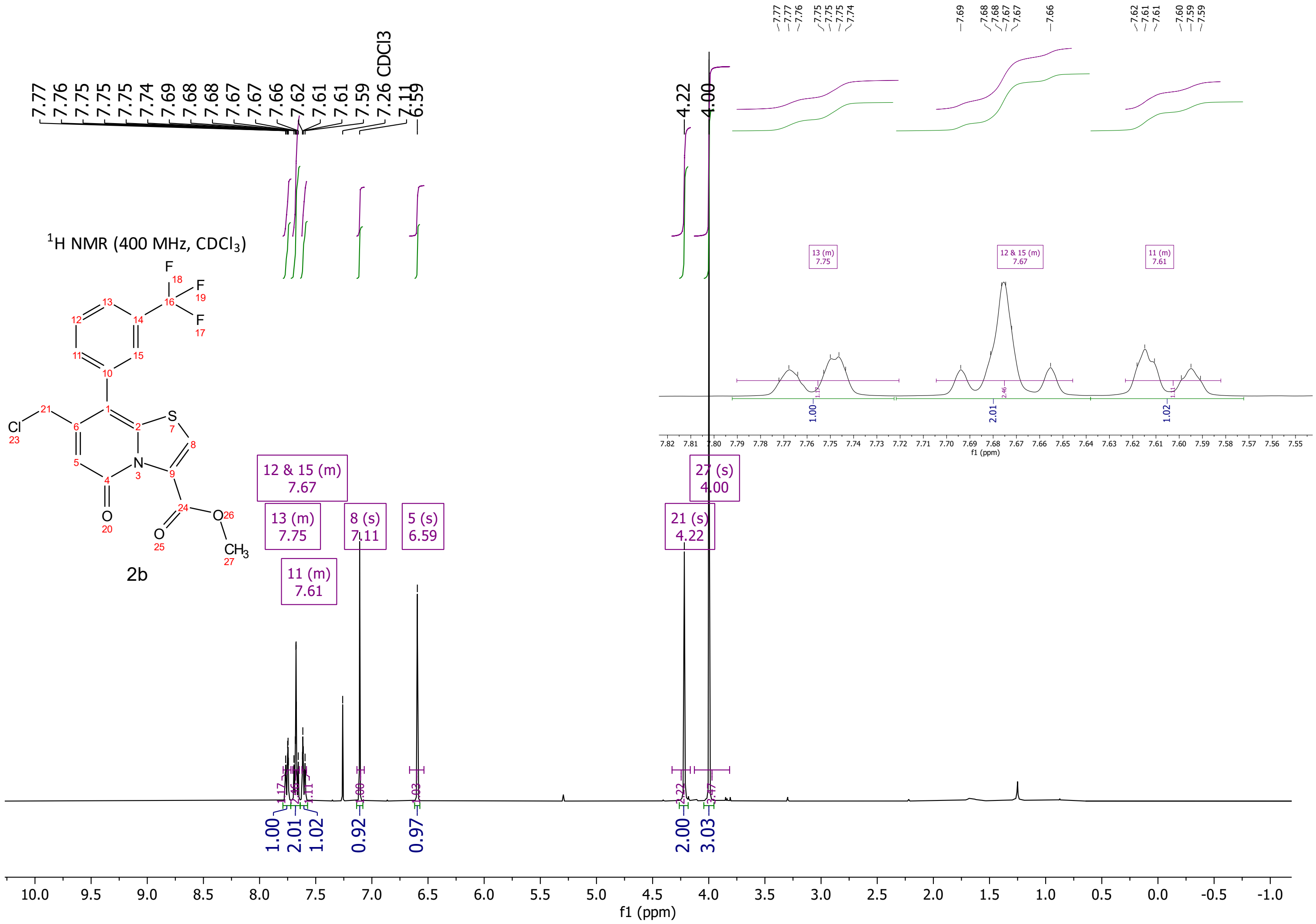

Figure S.55: ^1H NMR spectrum ($CDCl_3$, 400 MHz) of methyl 7-(chloromethyl)-5-oxo-8-(3-(trifluoromethyl)phenyl)-5H-thiazolo[3,2-a]pyridine-3-carboxylate, **2b**.

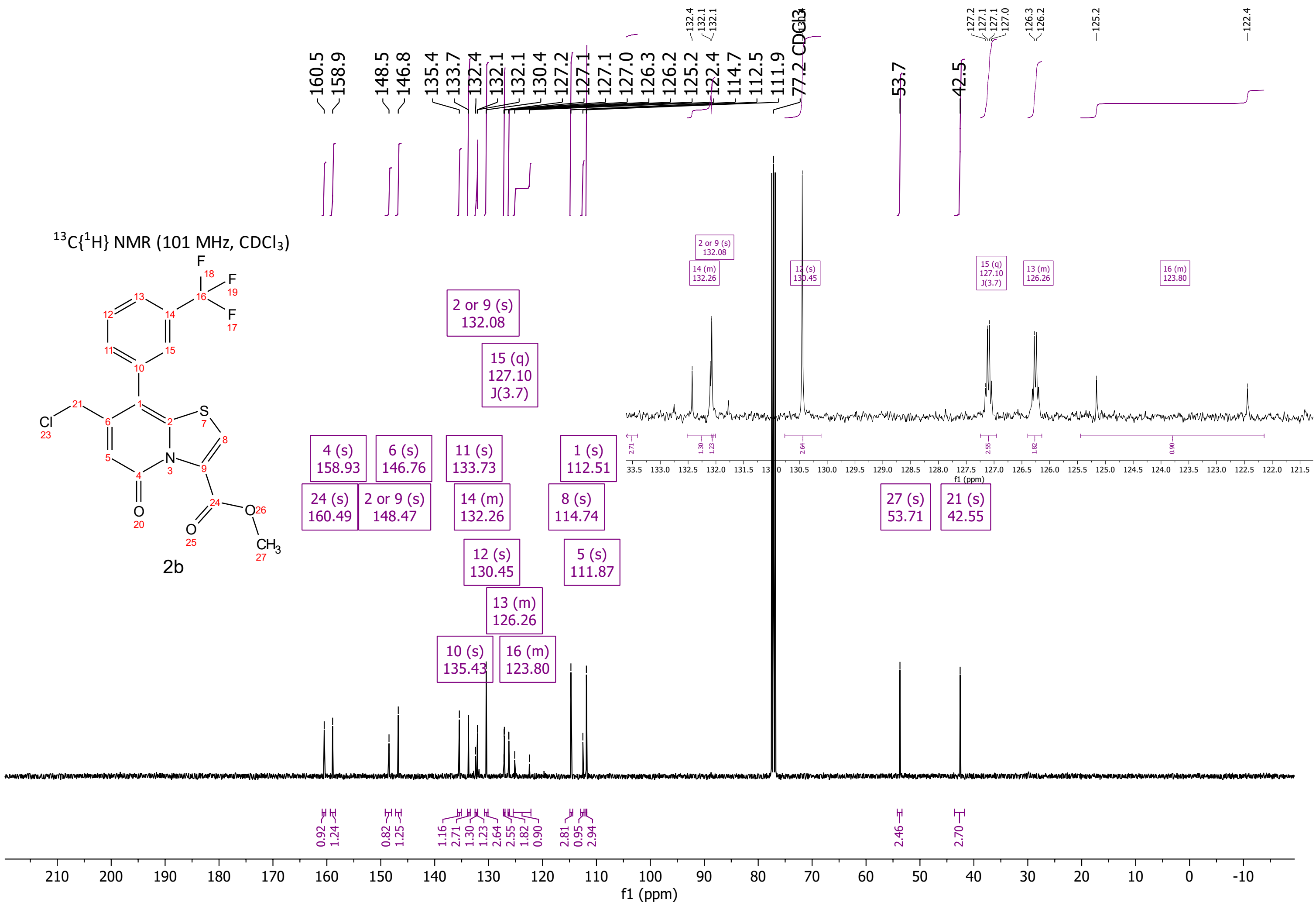

Figure S.56: $^{13}C\{^1H\}$ NMR spectrum ($CDCl_3$, 101 MHz) of methyl 7-(chloromethyl)-5-oxo-8-(3-(trifluoromethyl)phenyl)-5H-thiazolo[3,2-a]pyridine-3-carboxylate, **2b**.

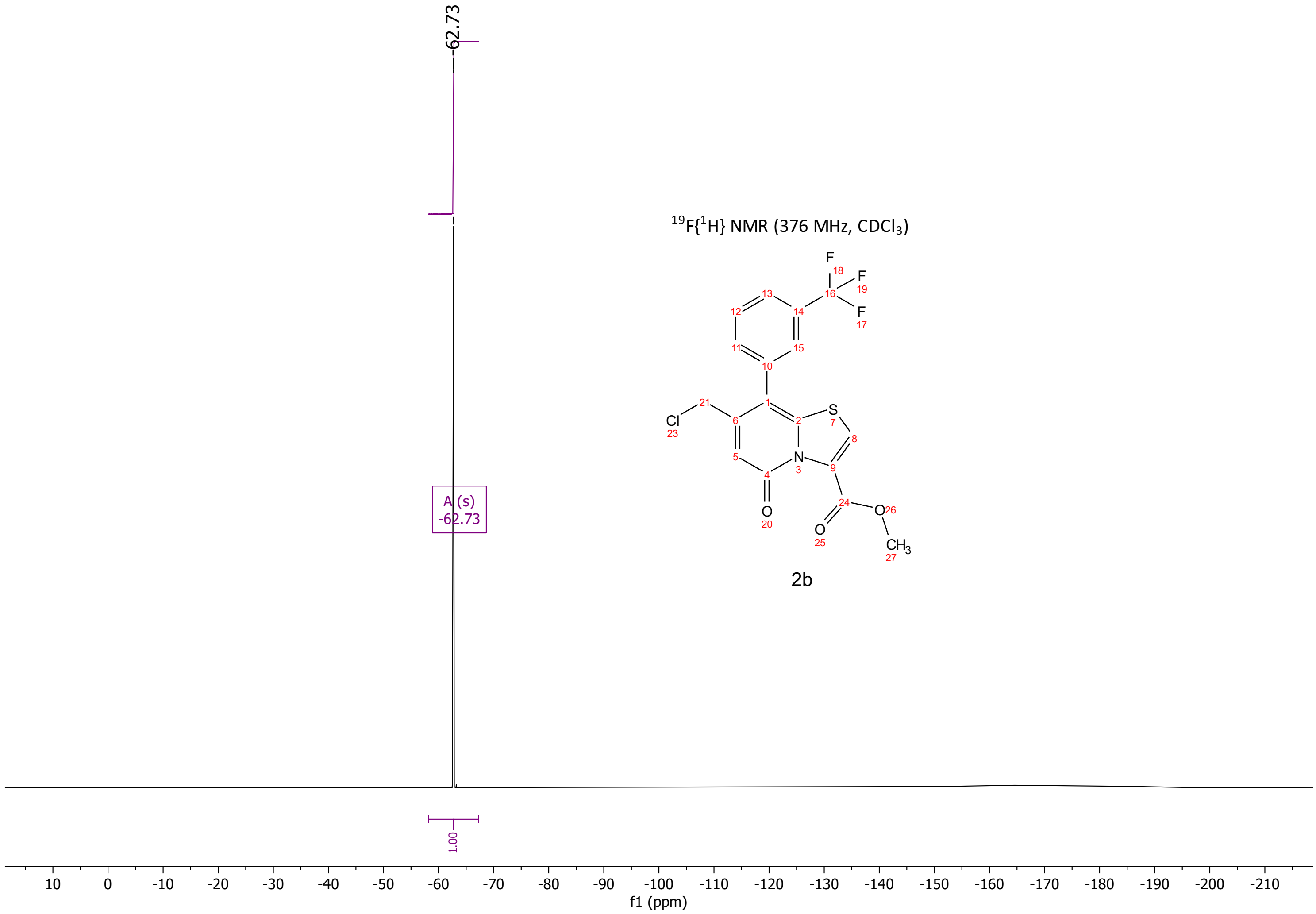

Figure S.57: $^{19}F\{^{1}H\}$ NMR spectrum ($CDCl_3$, 376 MHz) of methyl 7-(chloromethyl)-5-oxo-8-(3-(trifluoromethyl)phenyl)-5H-thiazolo[3,2-a]pyridine-3-carboxylate, **2b**.

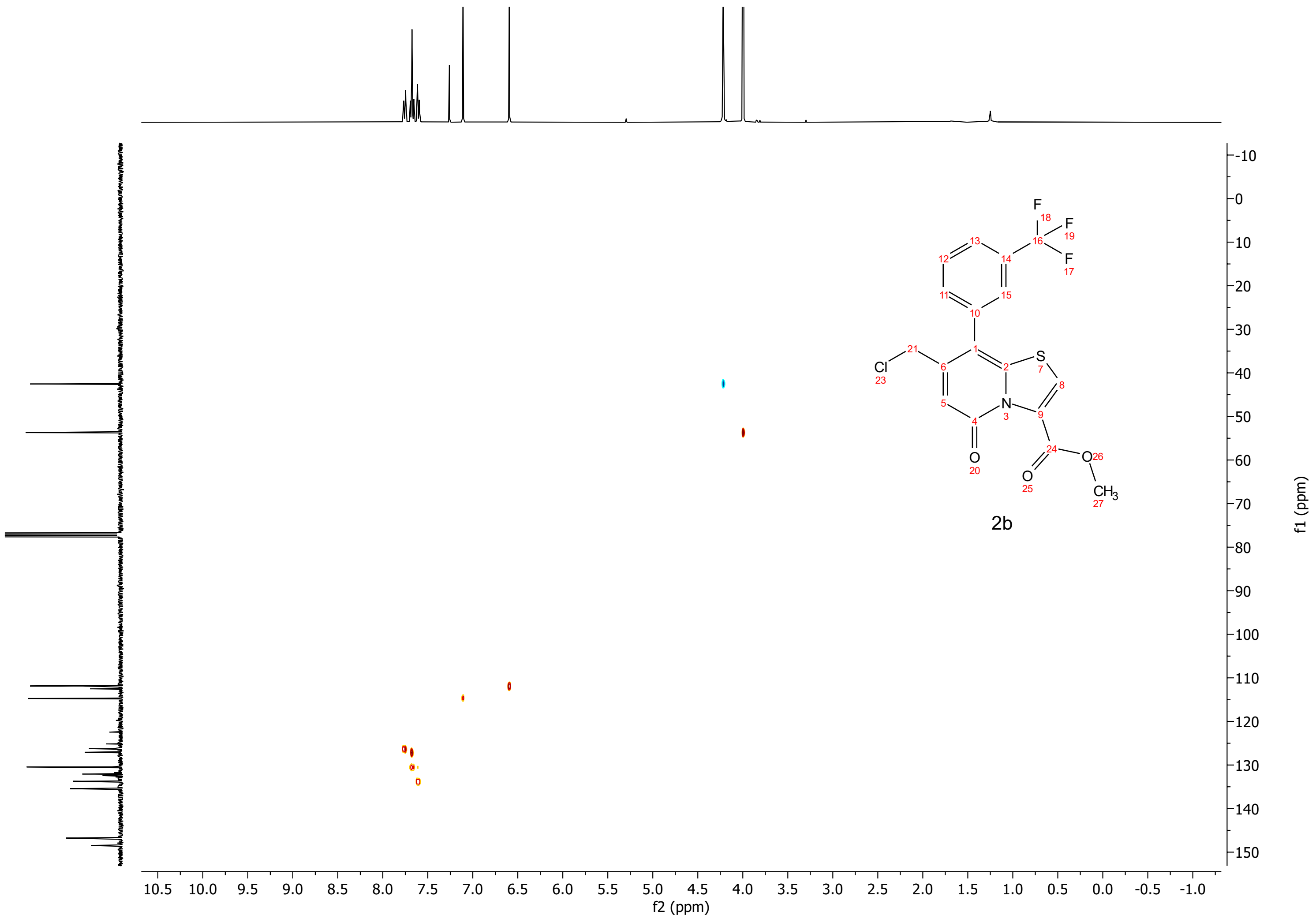

Figure S.58: gHSQC spectrum ($CDCl_3$) of methyl 7-(chloromethyl)-5-oxo-8-(3-(trifluoromethyl)phenyl)-5H-thiazolo[3,2-a]pyridine-3-carboxylate, **2b**.

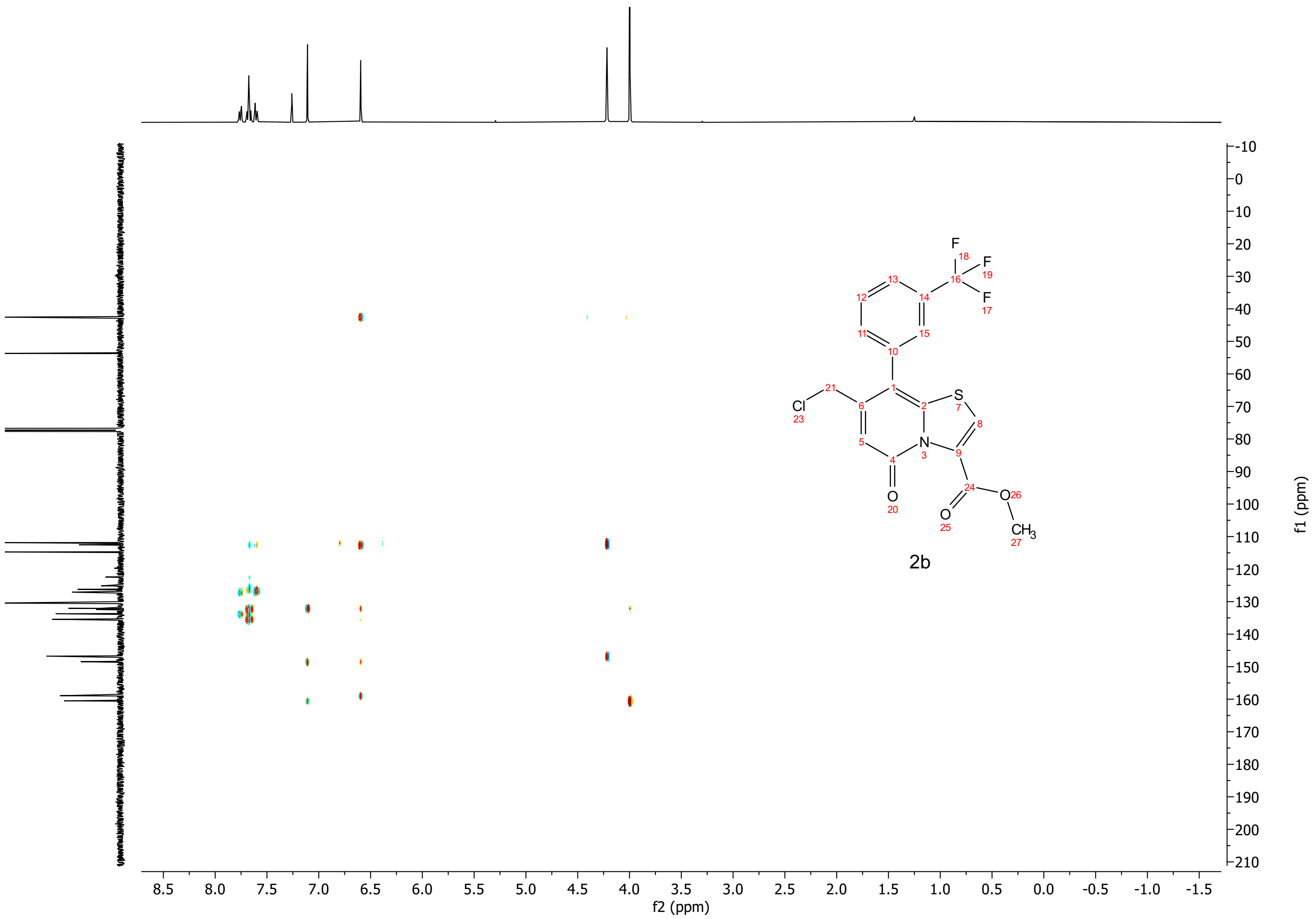

Figure S.59: gHMBC spectrum ($CDCl_3$) of methyl 7-(chloromethyl)-5-oxo-8-(3-(trifluoromethyl)phenyl)-5H-thiazolo[3,2-a]pyridine-3-carboxylate, **2b**.

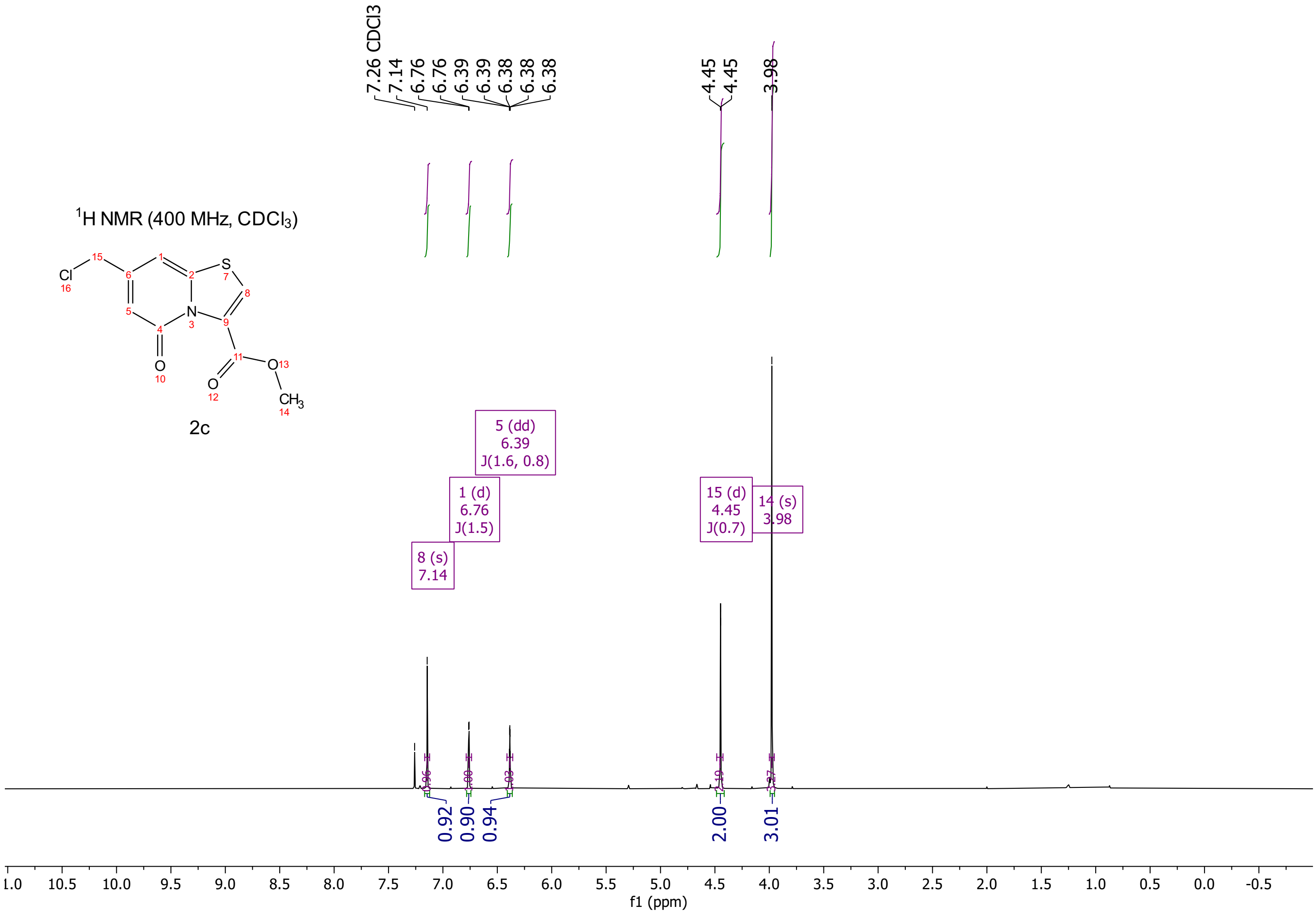

Figure S.60: ^{1}H NMR spectrum (CDCl$_3$, 400 MHz) of methyl 7-(chloromethyl)-5-oxo-5H-thiazolo[3,2-a]pyridine-3-carboxylate, **2c**.

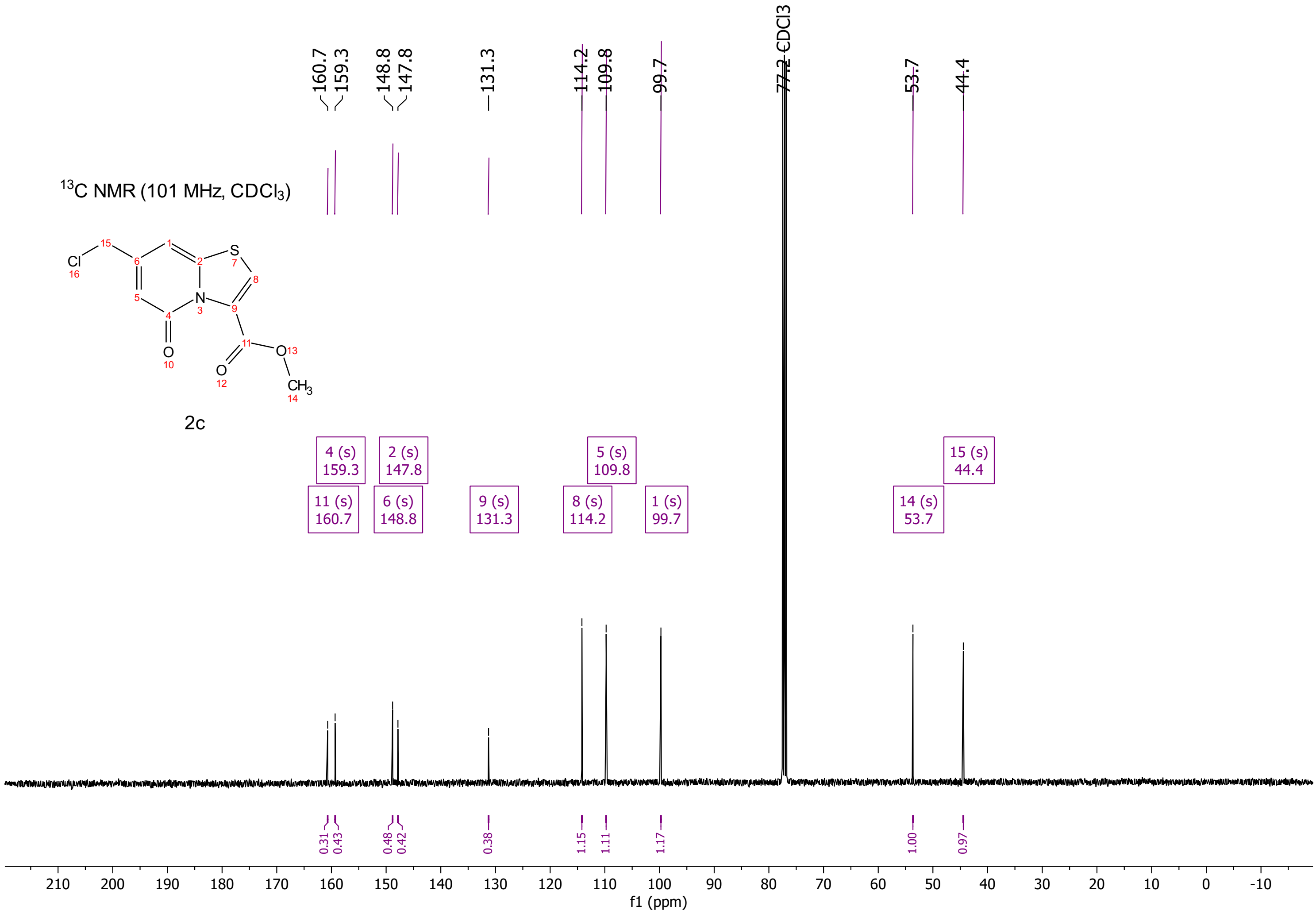

Figure S.61: $^{13}C\{^{1}H\}$ NMR spectrum ($CDCl_3$, 101 MHz) of methyl 7-(chloromethyl)-5-oxo-5H-thiazolo[3,2-a]pyridine-3-carboxylate, **2c**.

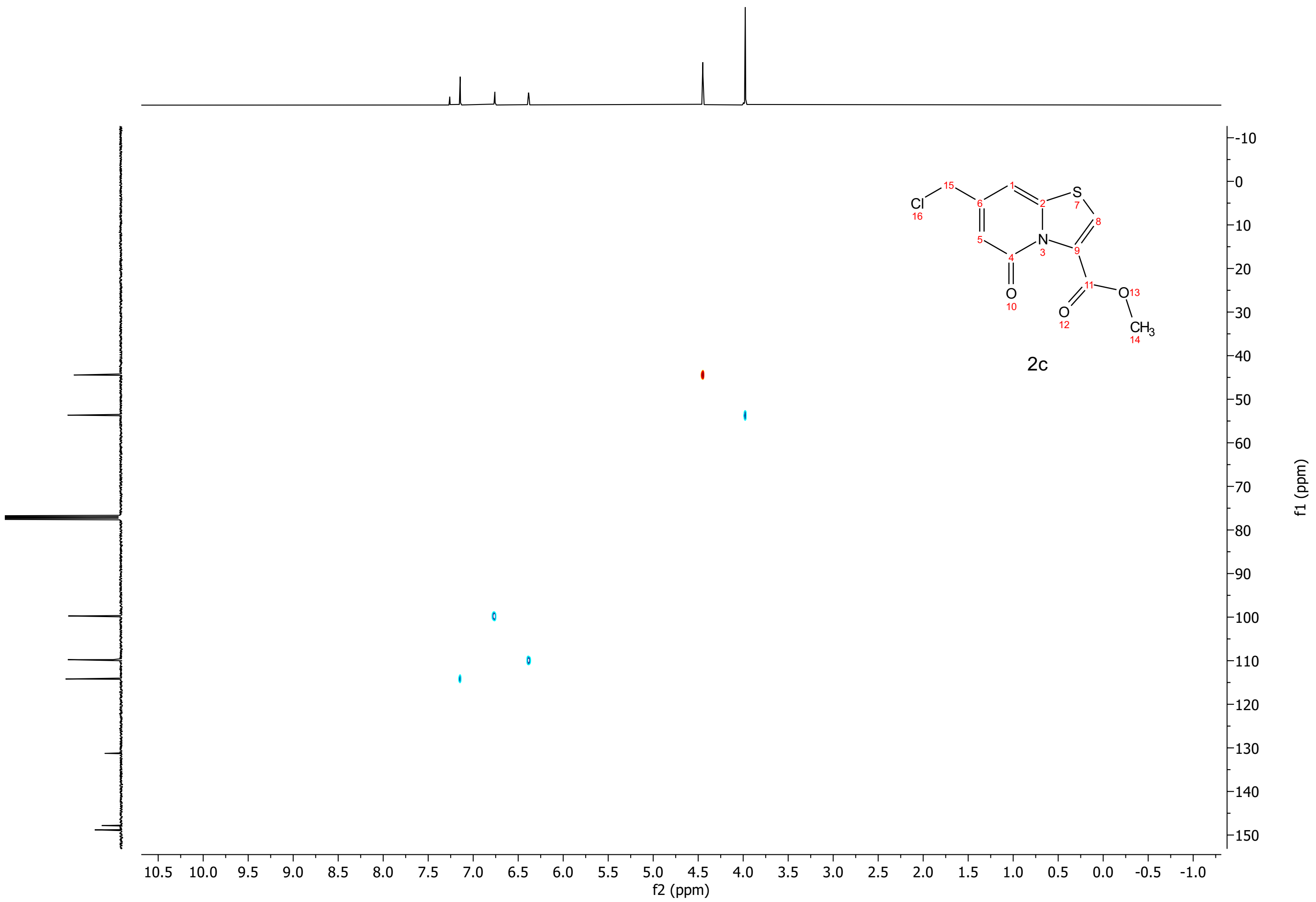

Figure S.62: gHSQC spectrum ($CDCl_3$) of methyl 7-(chloromethyl)-5-oxo-5H-thiazolo[3,2-a]pyridine-3-carboxylate, **2c**.

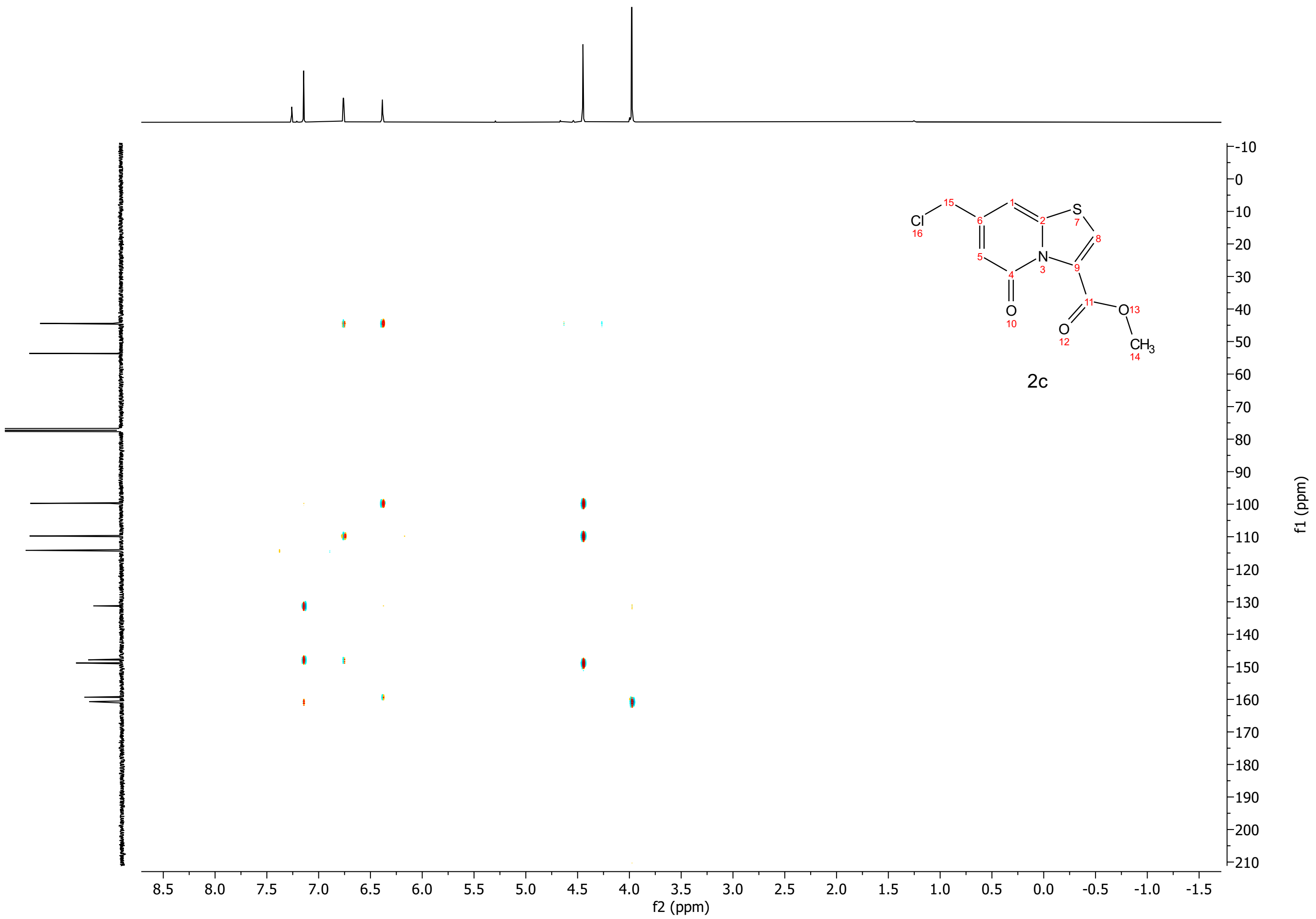

Figure S.63: gHMBC spectrum ($CDCl_3$) of methyl 7-(chloromethyl)-5-oxo-5H-thiazolo[3,2-a]pyridine-3-carboxylate, **2c**.

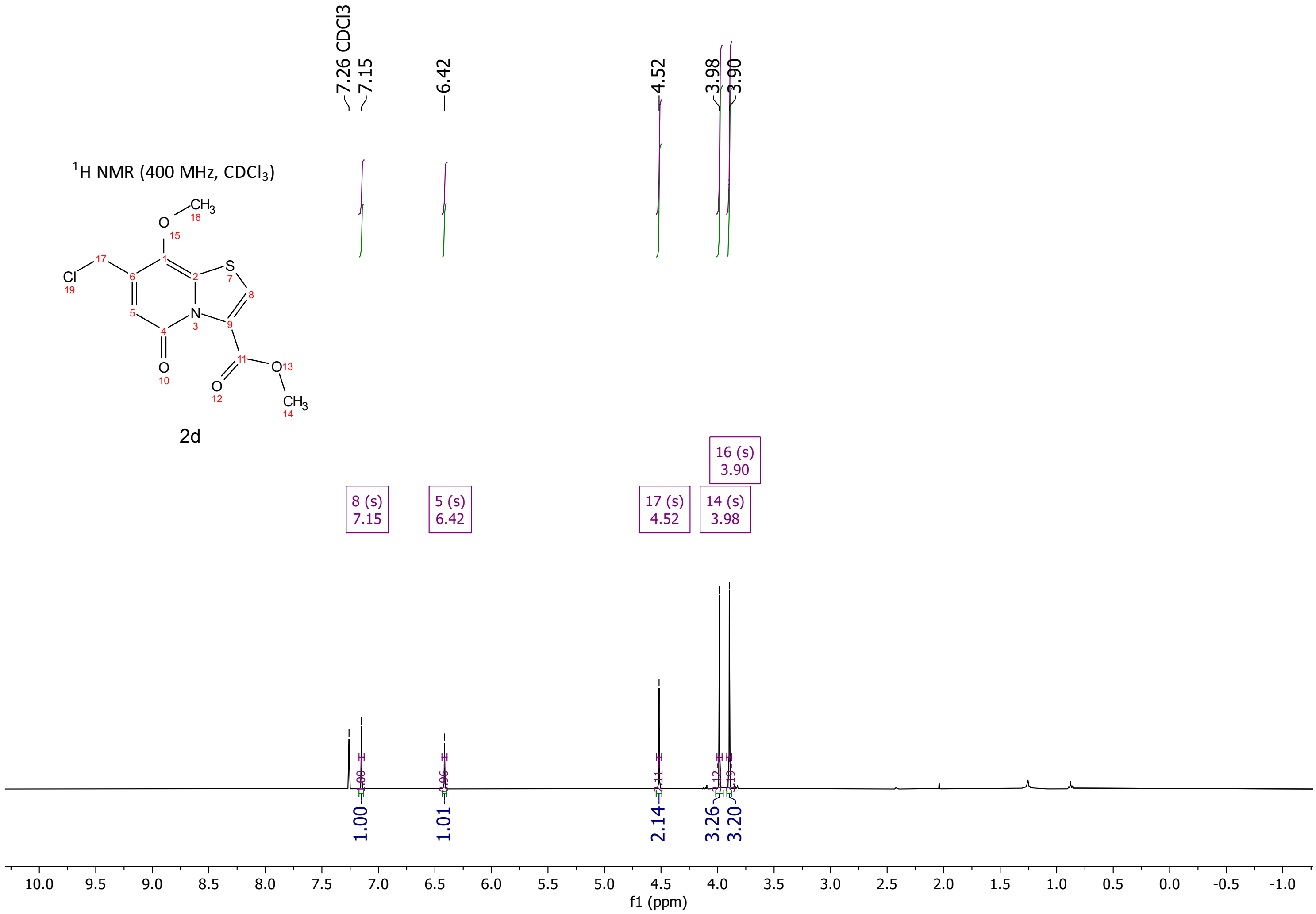

Figure S.64: ^{1}H NMR spectrum ($CDCl_3$, 400 MHz) of methyl 7-(chloromethyl)-8-methoxy-5-oxo-5H-thiazolo[3,2- a]pyridine-3-carboxylate, **2d**.

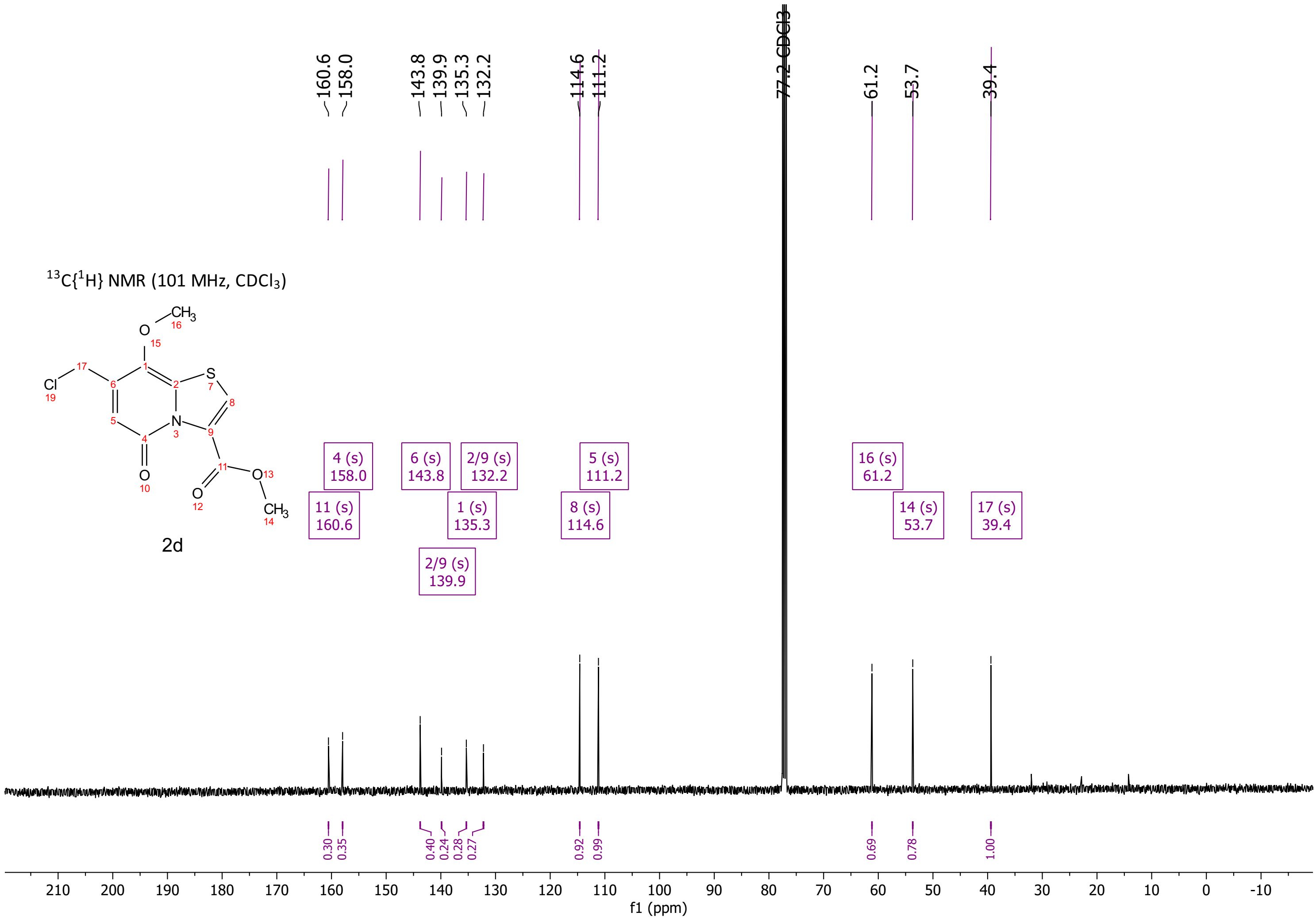

Figure S.65: $^{13}C\{^1H\}$ NMR spectrum ($CDCl_3$, 101 MHz) of methyl 7-(chloromethyl)-8- methoxy-5-oxo-5H-thiazolo[3,2- a]pyridine-3-carboxylate, **2d**.

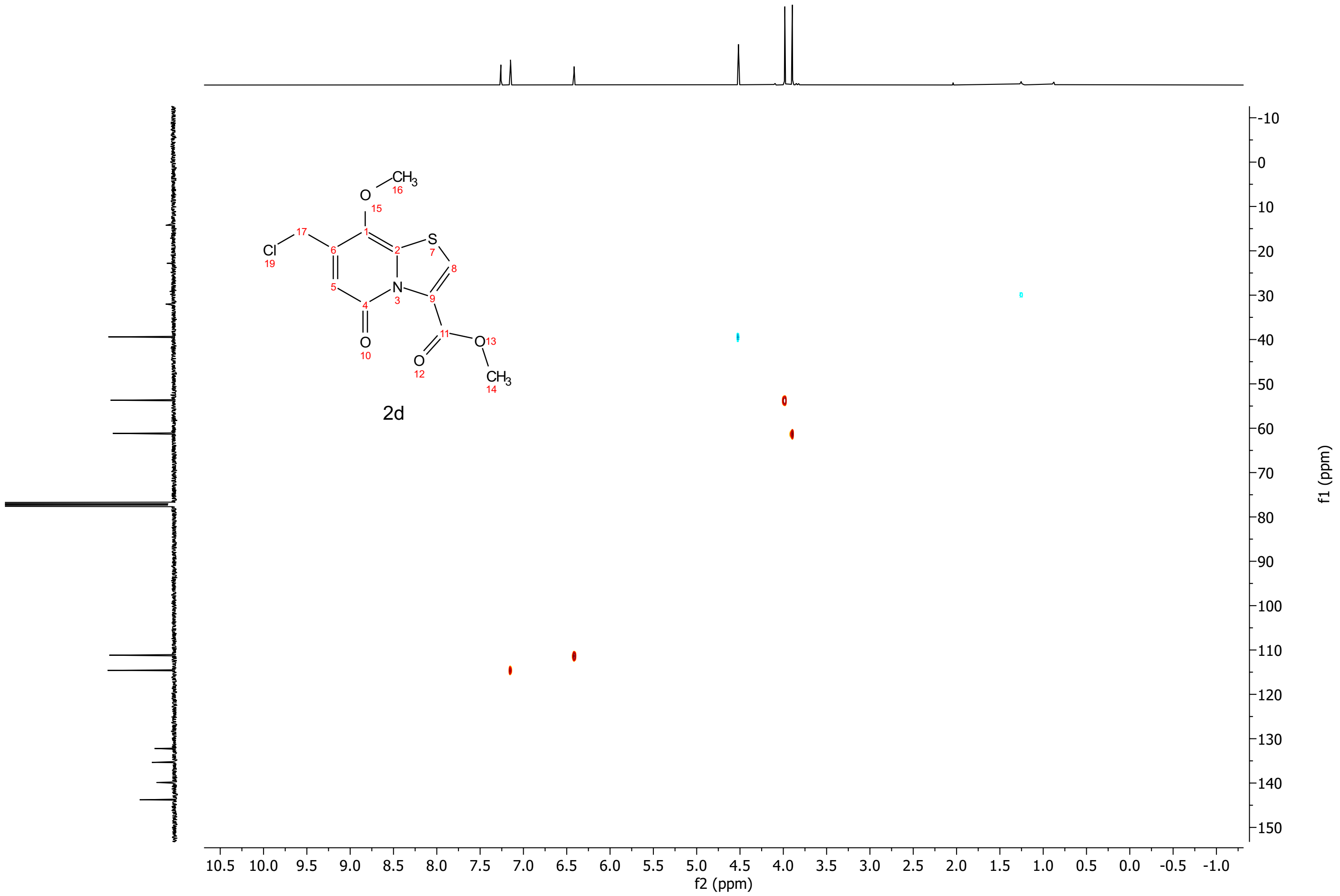

Figure S.66: gHSQC spectrum ($CDCl_3$) of methyl 7-(chloromethyl)-8- methoxy-5-oxo-5H-thiazolo[3,2- a]pyridine-3-carboxylate, **2d**.

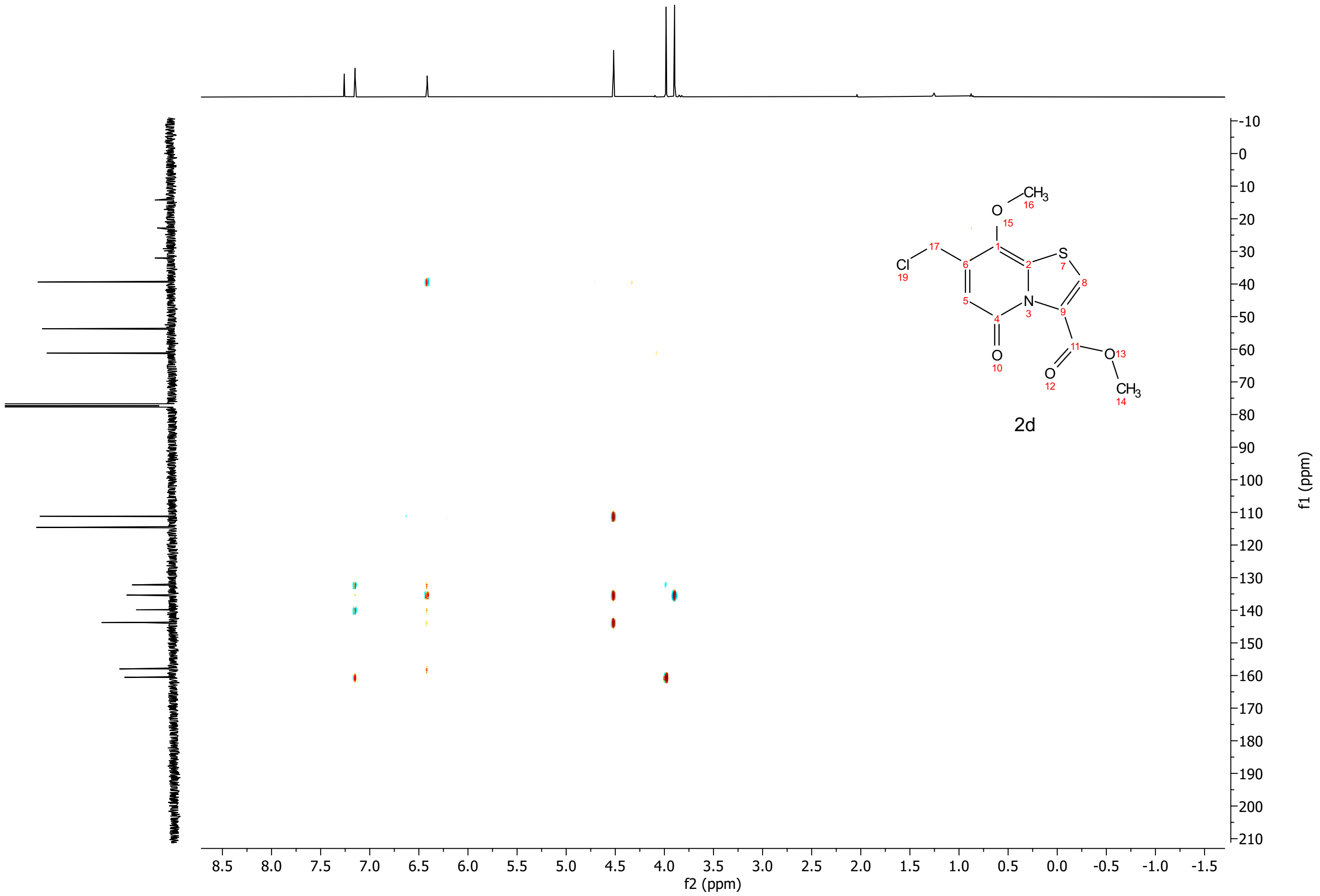

Figure S.67: gHMBC spectrum ($CDCl_3$) of methyl 7-(chloromethyl)-8- methoxy-5-oxo-5H-thiazolo[3,2- a]pyridine-3-carboxylate, **2d**.

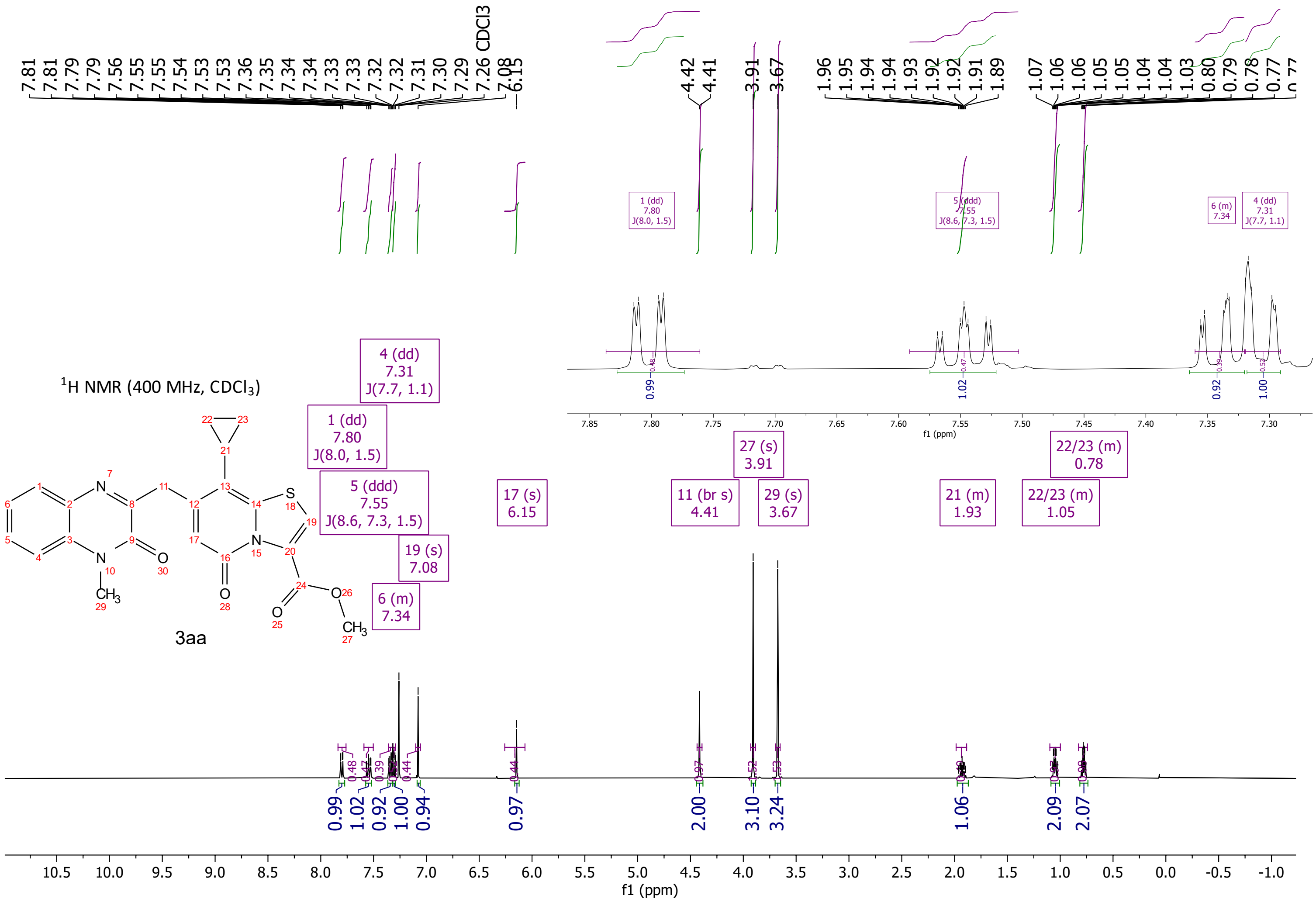

Figure S.68: ^{1}H NMR spectrum ($CDCl_3$, 400 MHz) of methyl 8-cyclopropyl-7-((4-methyl-3-oxo-3,4-dihydroquinoxalin-2-yl)methyl)-5-oxo-5H-thiazolo[3,2-a]pyridine-3-carboxylate, **3aa**.

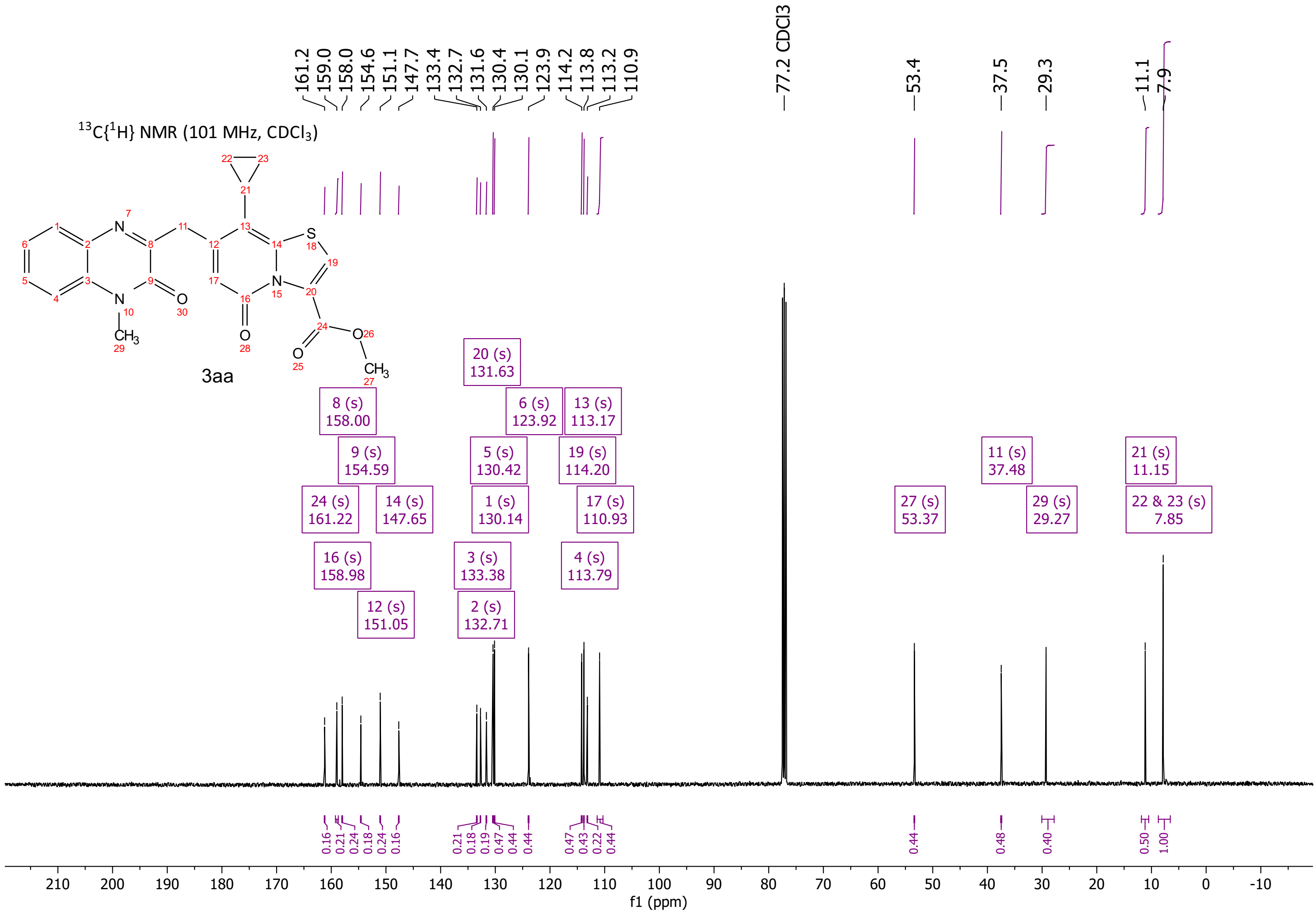

Figure S.69: ${}^{13}C\{{}^{1}H\}$ NMR spectrum ($CDCl_3$, 101 MHz) of methyl 8-cyclopropyl-7-((4-methyl-3-oxo-3,4-dihydroquinoxalin-2-yl)methyl)-5-oxo-5H-thiazolo[3,2-a]pyridine-3-carboxylate, **3aa**.

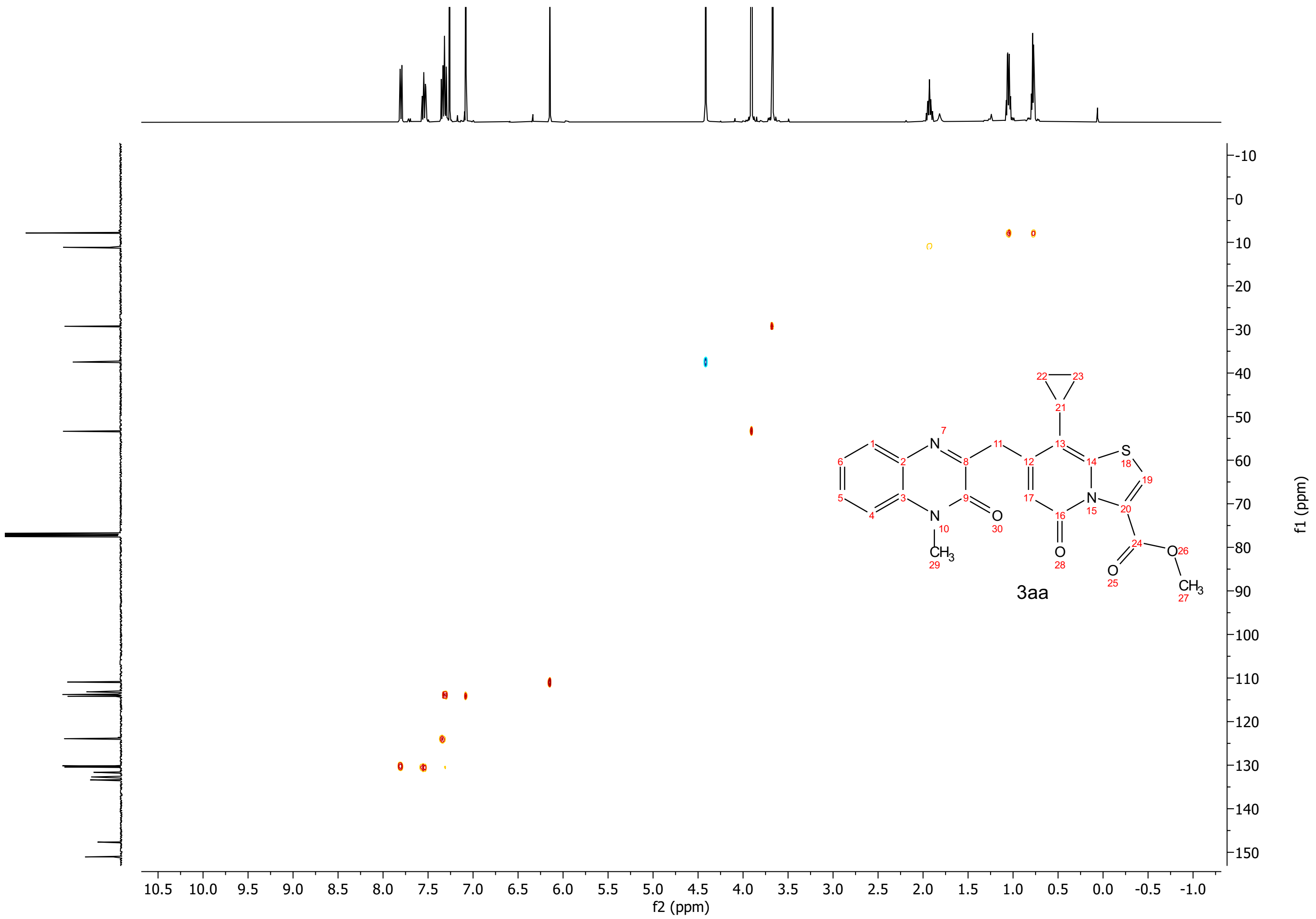

Figure S.70: gHSQC spectrum ($CDCl_3$) of methyl 8-cyclopropyl-7-((4-methyl-3-oxo-3,4-dihydroquinoxalin-2-yl)methyl)-5-oxo-5H-thiazolo[3,2-a]pyridine-3-carboxylate, **3aa**.

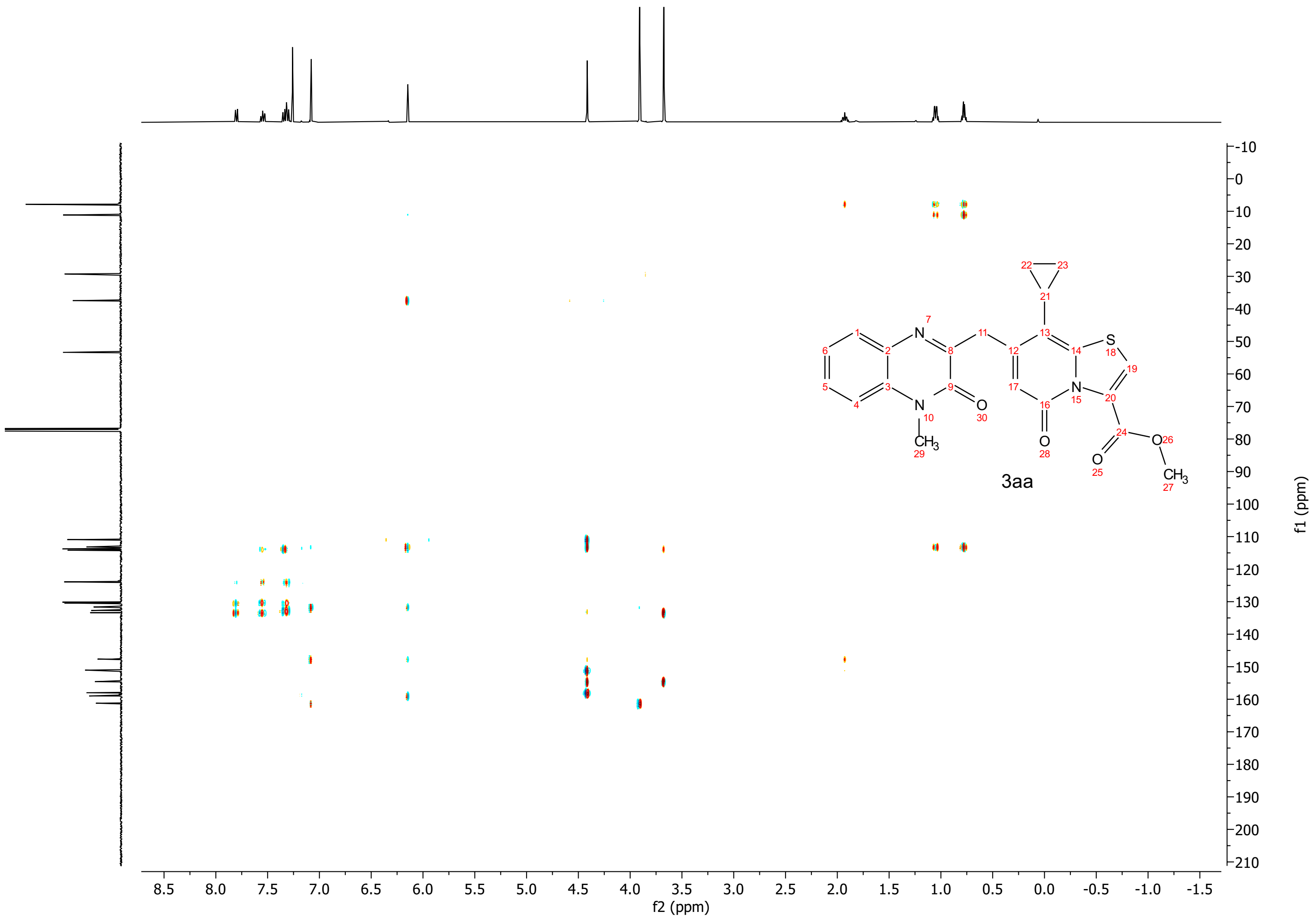

Figure S.71: gHMBC spectrum ($CDCl_3$) of methyl 8-cyclopropyl-7-((4-methyl-3-oxo-3,4-dihydroquinoxalin-2-yl)methyl)-5-oxo-5H-thiazolo[3,2-a]pyridine-3-carboxylate, **3aa**.

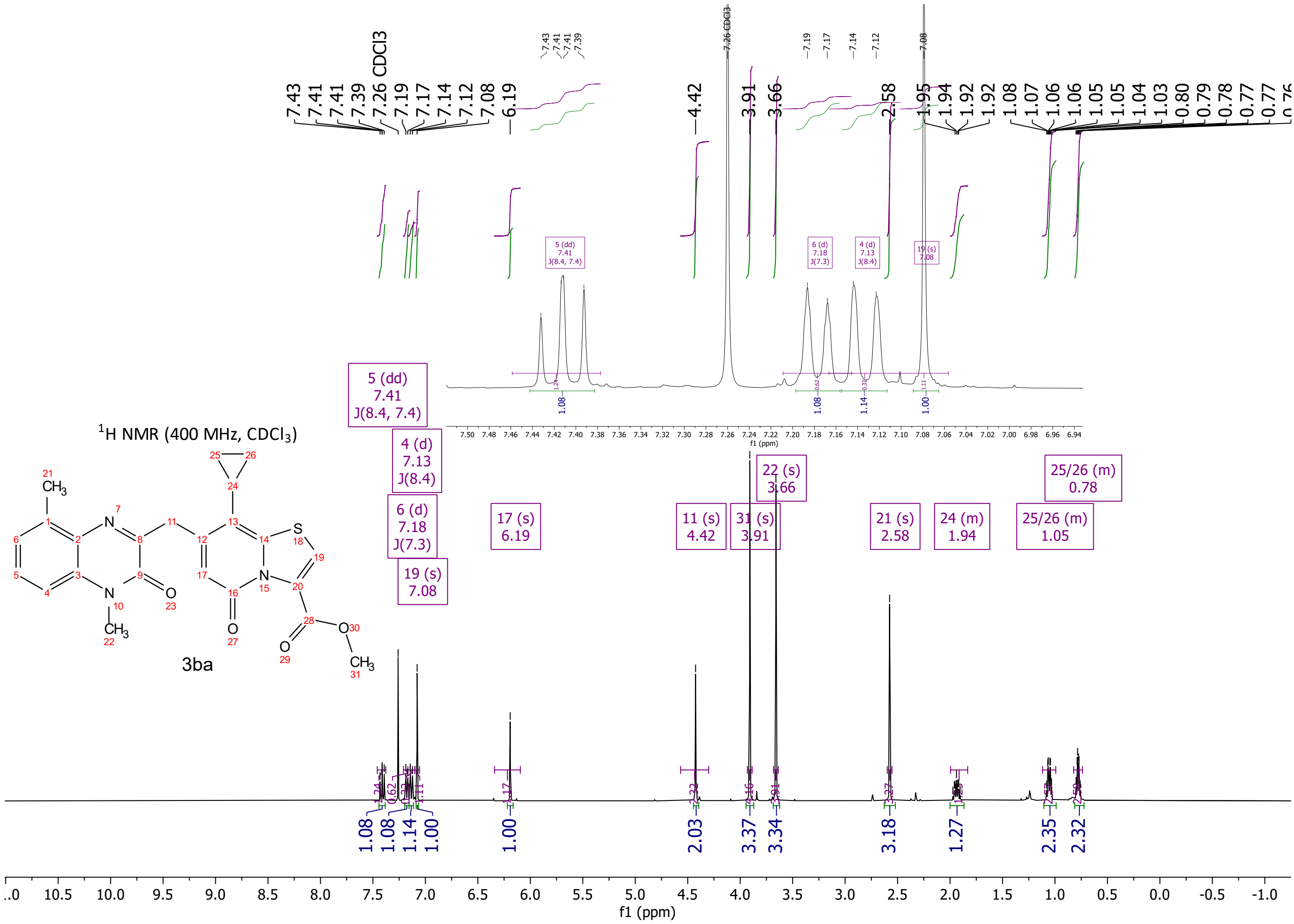

Figure S.72: ^{1}H NMR spectrum (CDCl$_3$, 400 MHz) of methyl 8-cyclopropyl-7-((4,8-dimethyl-3-oxo-3,4-dihydroquinoxalin-2-yl)methyl)-5-oxo-5H-thiazolo[3,2-a]pyridine-3-carboxylate, **3ba**.

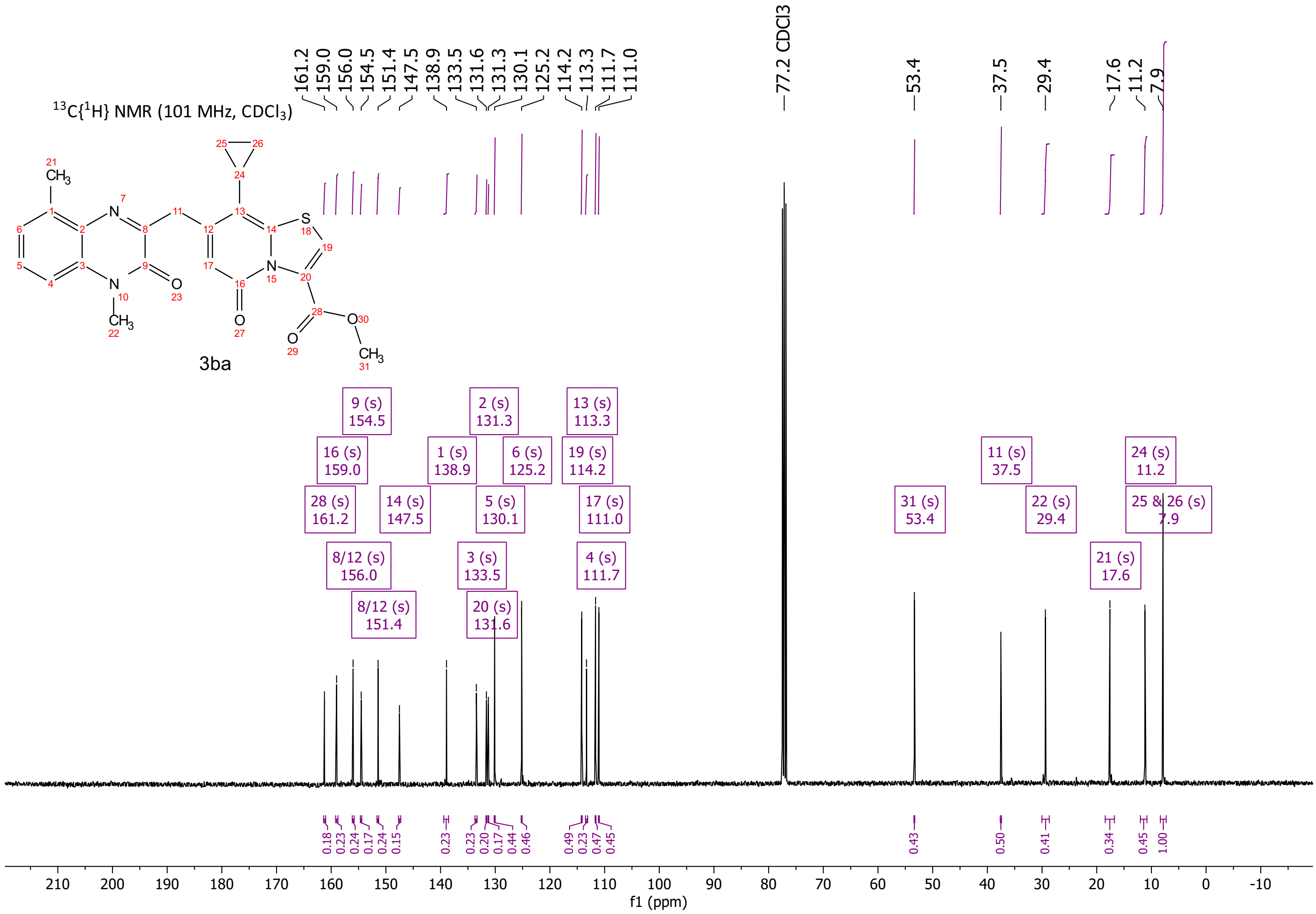

Figure S.73: $^{13}C\{^{1}H\}$ NMR spectrum ($CDCl_3$, 101 MHz) of methyl 8-cyclopropyl-7-((4,8-dimethyl-3-oxo-3,4-dihydroquinoxalin-2-yl)methyl)-5-oxo-5H-thiazolo[3,2-a]pyridine-3-carboxylate, **3ba**.

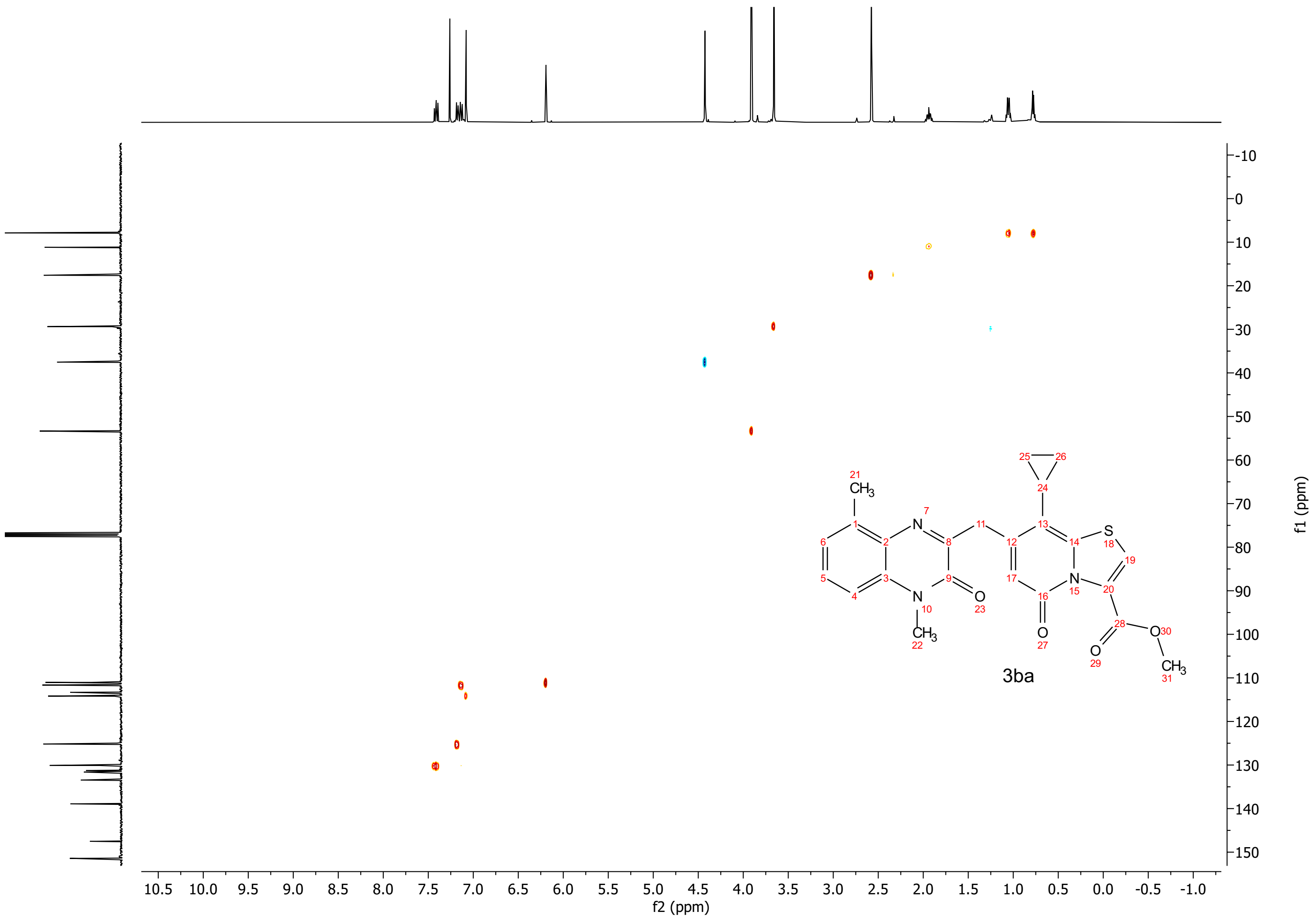

Figure S.74: gHSQC spectrum ($CDCl_3$) of methyl 8-cyclopropyl-7-((4,8-dimethyl-3-oxo-3,4-dihydroquinoxalin-2-yl)methyl)-5-oxo-5H-thiazolo[3,2-a]pyridine-3-carboxylate, **3ba**.

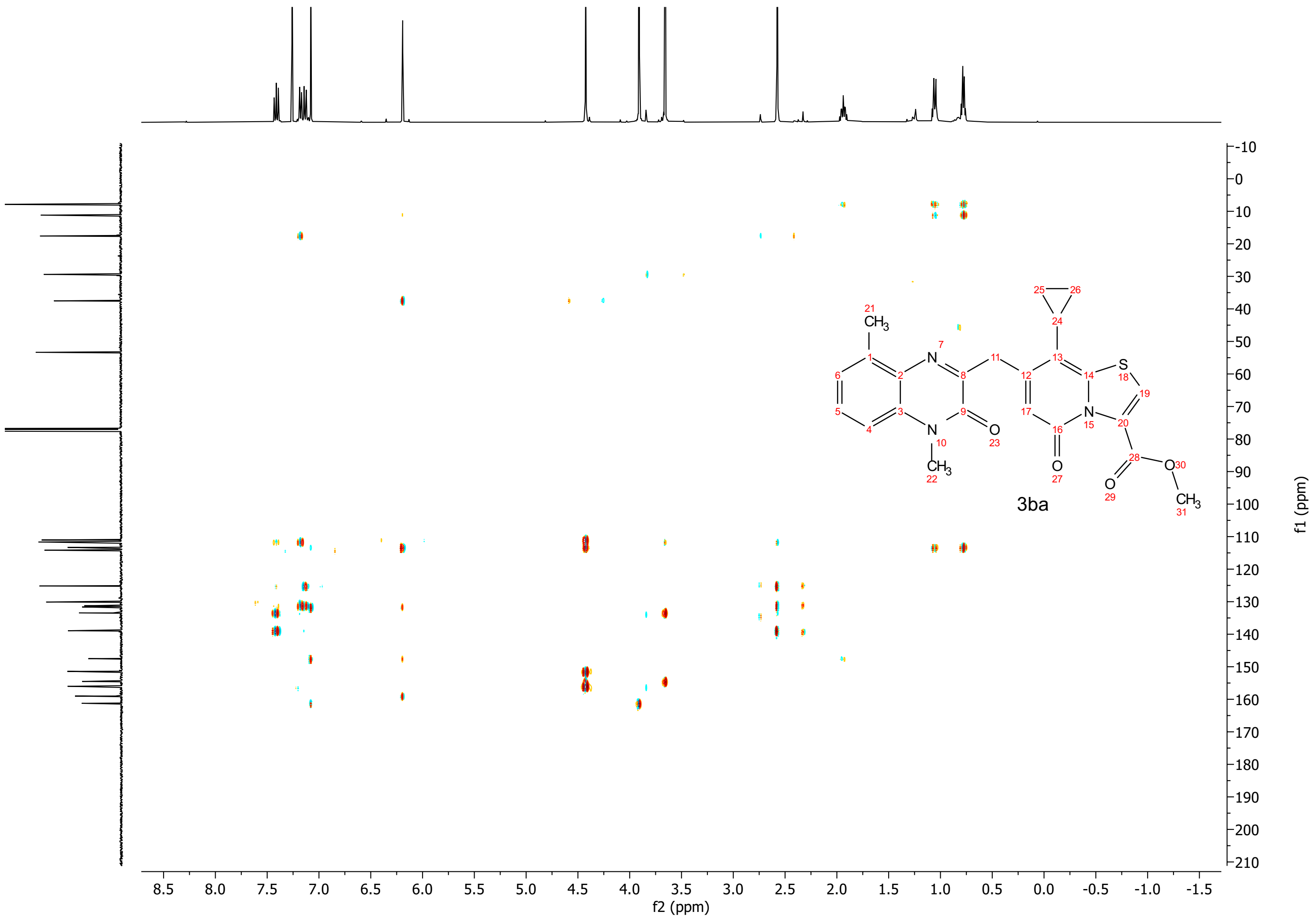

Figure S.75: gHMBC spectrum ($CDCl_3$) of methyl 8-cyclopropyl-7-((4,8-dimethyl-3-oxo-3,4-dihydroquinoxalin-2-yl)methyl)-5-oxo-5H-thiazolo[3,2-a]pyridine-3-carboxylate, **3ba**.

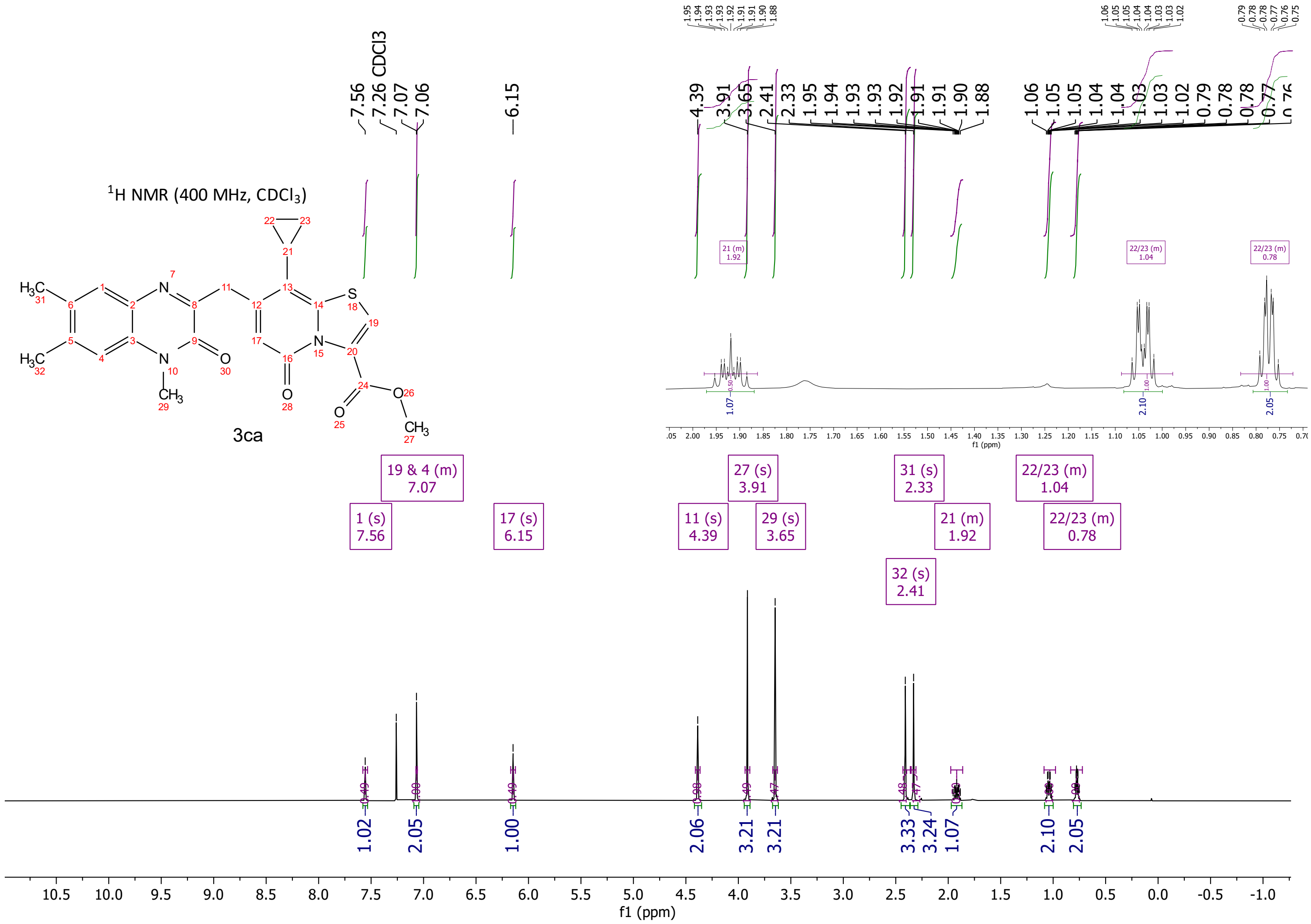

Figure S.76: ^{1}H NMR spectrum ($CDCl_3$, 400 MHz) of methyl 8-cyclopropyl-5-oxo-7-((4,6,7-trimethyl-3-oxo-3,4-dihydroquinoxalin-2-yl)methyl)-5H-thiazolo[3,2-a]pyridine-3-carboxylate, **3ca**.

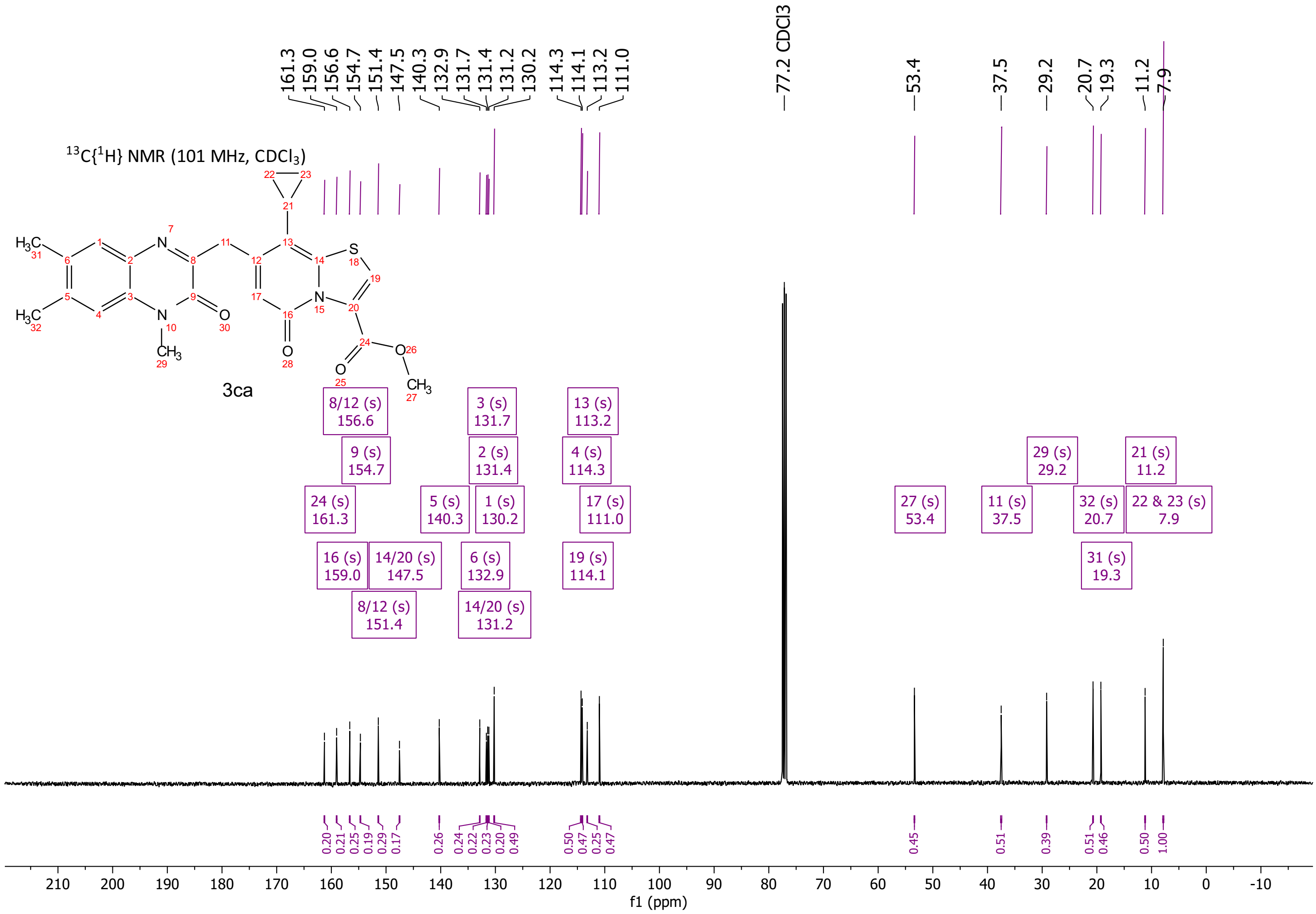

Figure S.77: $^{13}C\{^1H\}$ NMR spectrum ($CDCl_3$, 101 MHz) of methyl 8-cyclopropyl-5-oxo-7-((4,6,7-trimethyl-3-oxo-3,4-dihydroquinoxalin-2-yl)methyl)-5H-thiazolo[3,2-a]pyridine-3-carboxylate, **3ca**.

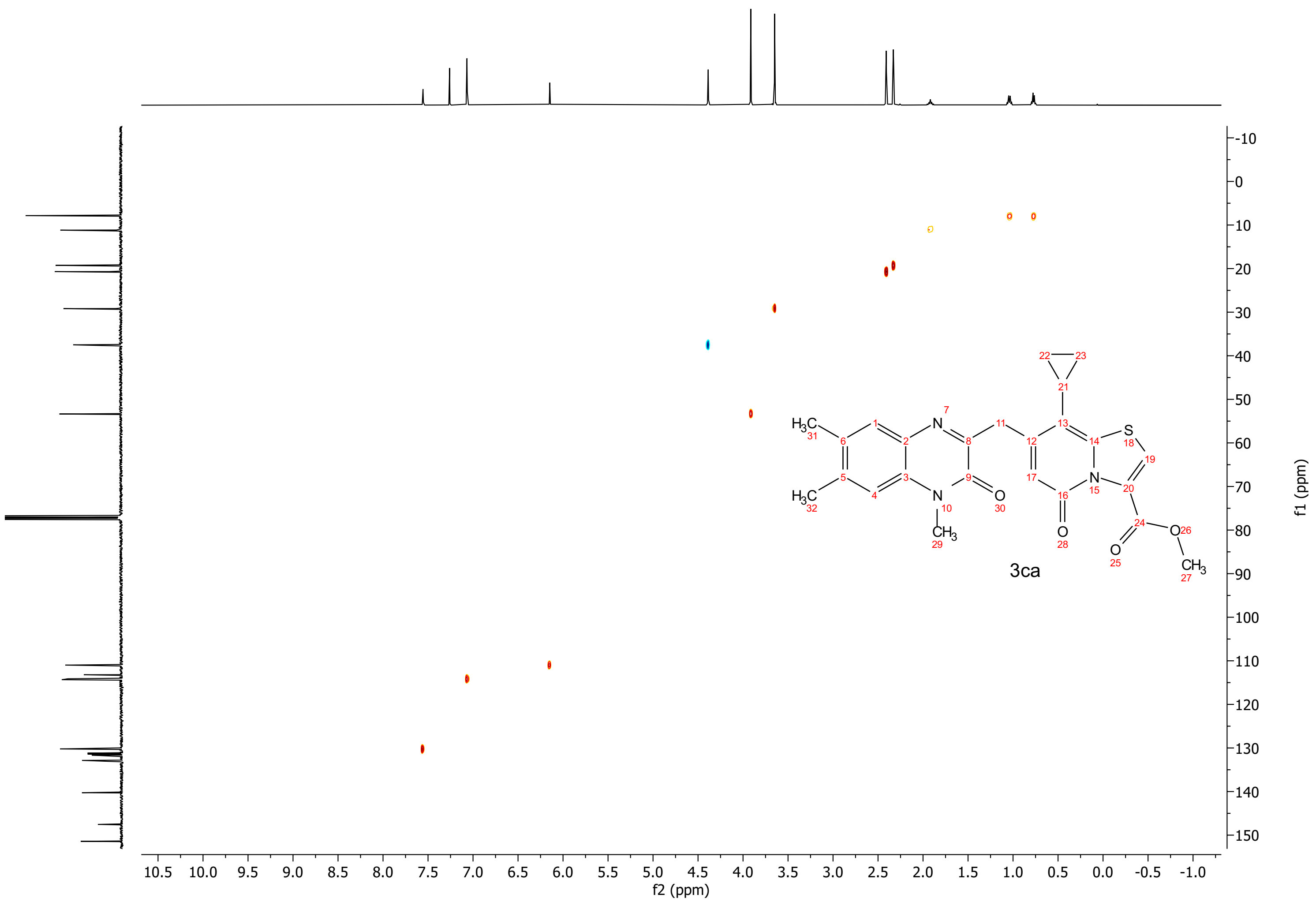

Figure S.78: gHSQC spectrum ($CDCl_3$) of methyl 8-cyclopropyl-5-oxo-7-((4,6,7-trimethyl-3-oxo-3,4-dihydroquinoxalin-2-yl)methyl)-5H-thiazolo[3,2-a]pyridine-3-carboxylate, **3ca**.

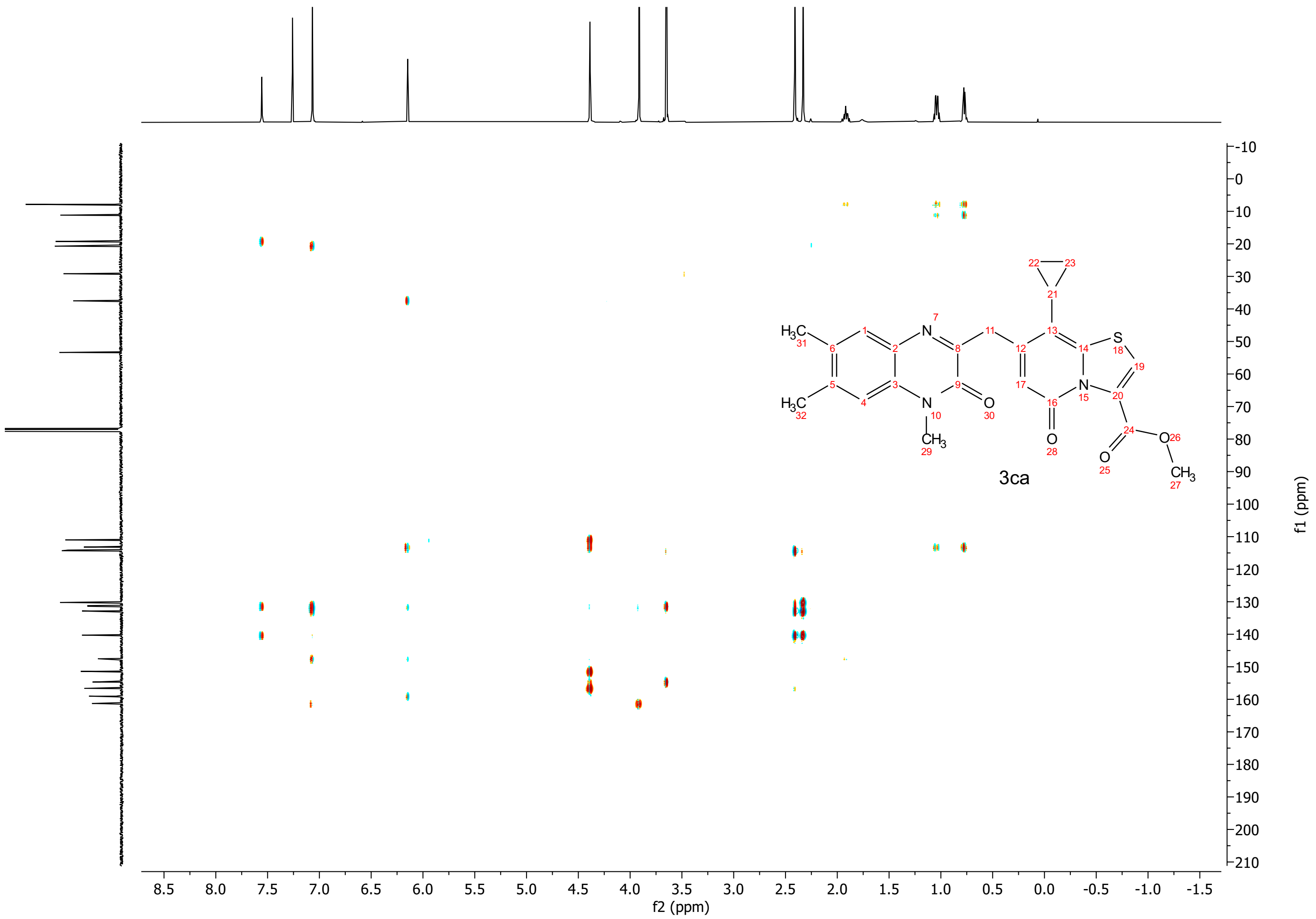

Figure S.79: gHMBC spectrum ($CDCl_3$) of methyl 8-cyclopropyl-5-oxo-7-((4,6,7-trimethyl-3-oxo-3,4-dihydroquinoxalin-2-yl)methyl)-5H-thiazolo[3,2-a]pyridine-3-carboxylate, **3ca**.

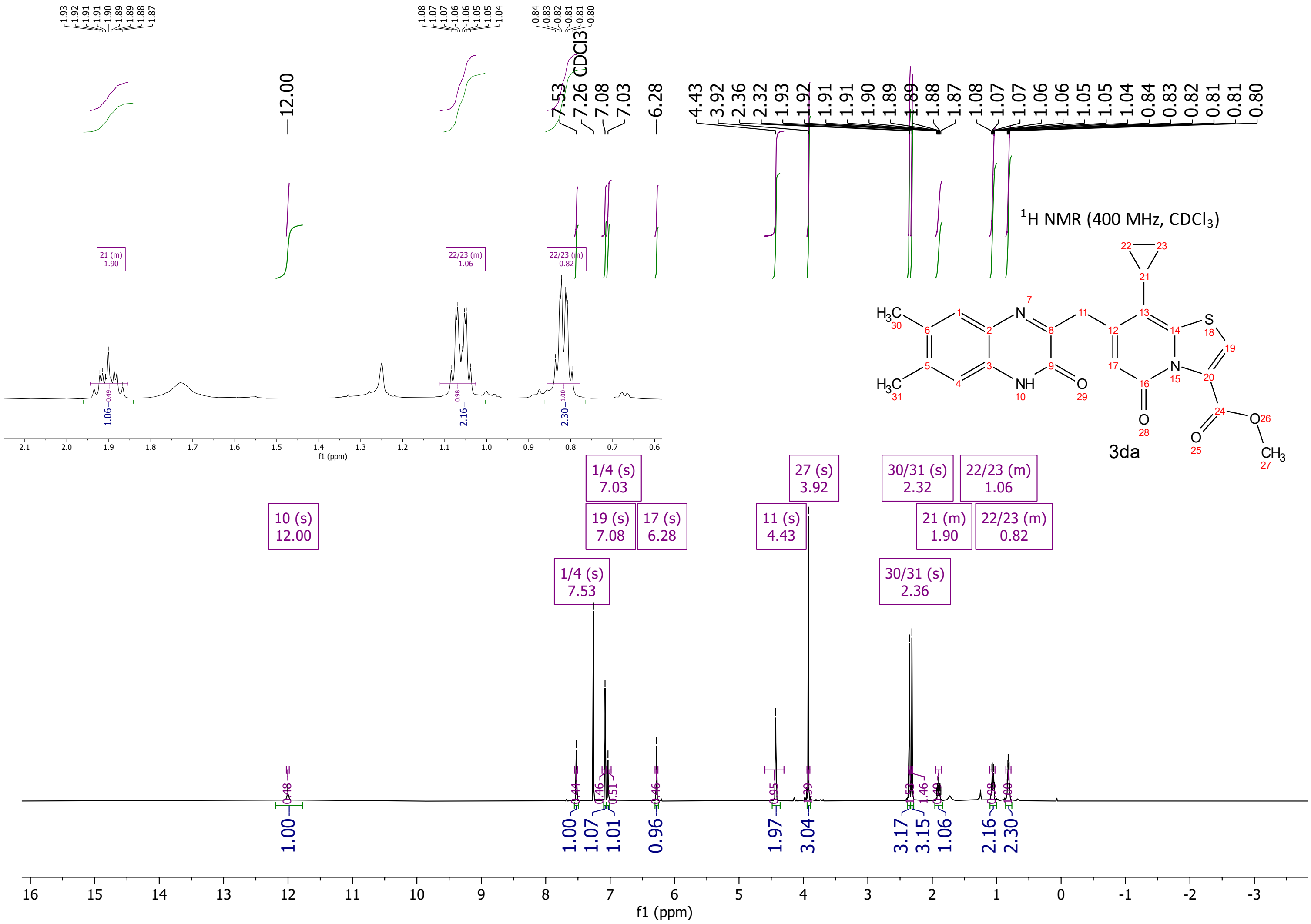

Figure S.80: ^{1}H NMR spectrum ($CDCl_3$, 400 MHz) of methyl 8-cyclopropyl-7-((6,7-dimethyl-3-oxo-3,4-dihydroquinoxalin-2-yl)methyl)-5-oxo-5H-thiazolo[3,2-a]pyridine-3-carboxylate, **3da**.

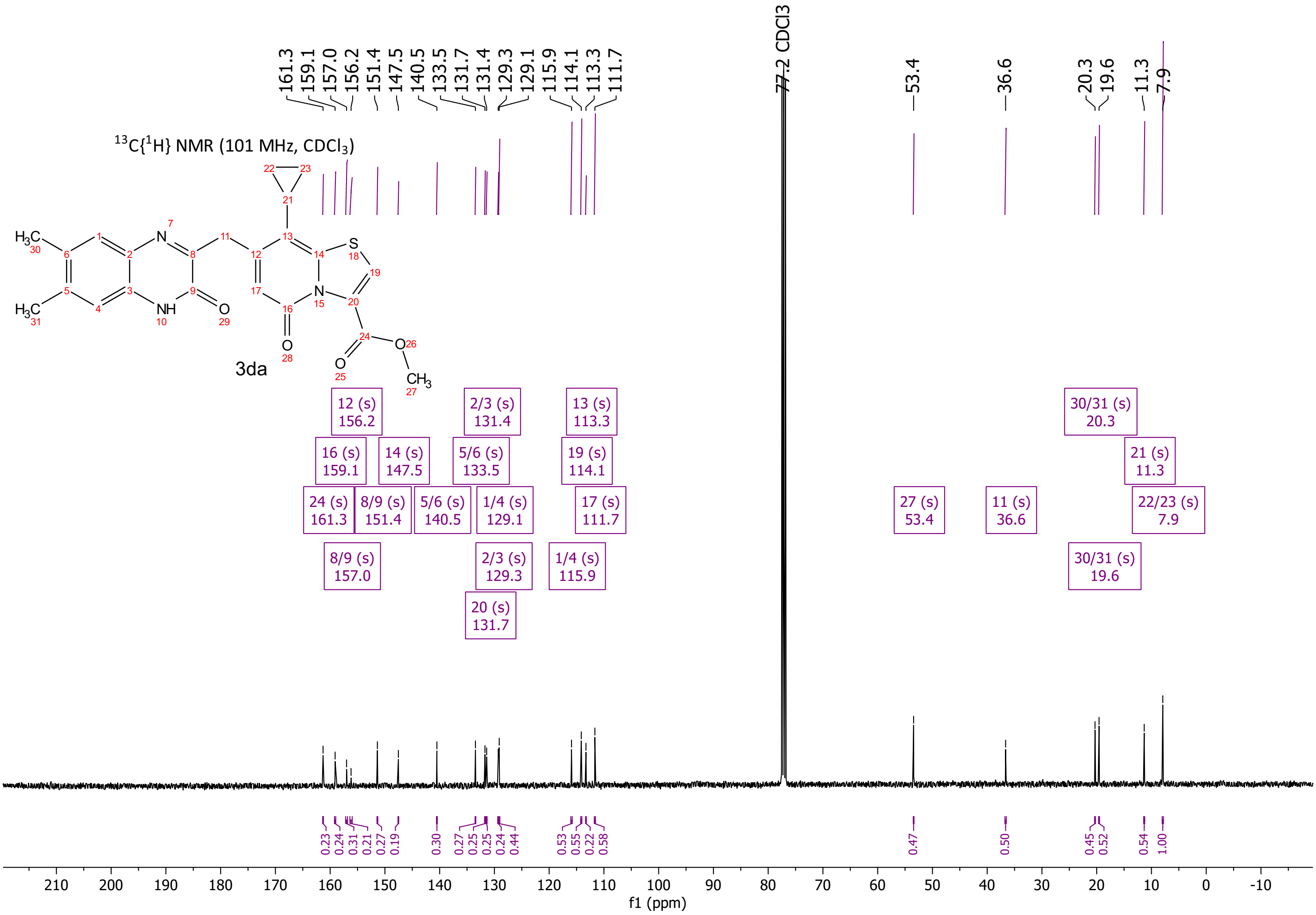

Figure S.81: $^{13}C\{^{1}H\}$ NMR spectrum ($CDCl_3$, 101 MHz) of methyl 8-cyclopropyl-7-((6,7-dimethyl-3-oxo-3,4-dihydroquinoxalin-2-yl)methyl)-5-oxo-5H-thiazolo[3,2-a]pyridine-3-carboxylate, **3da**.

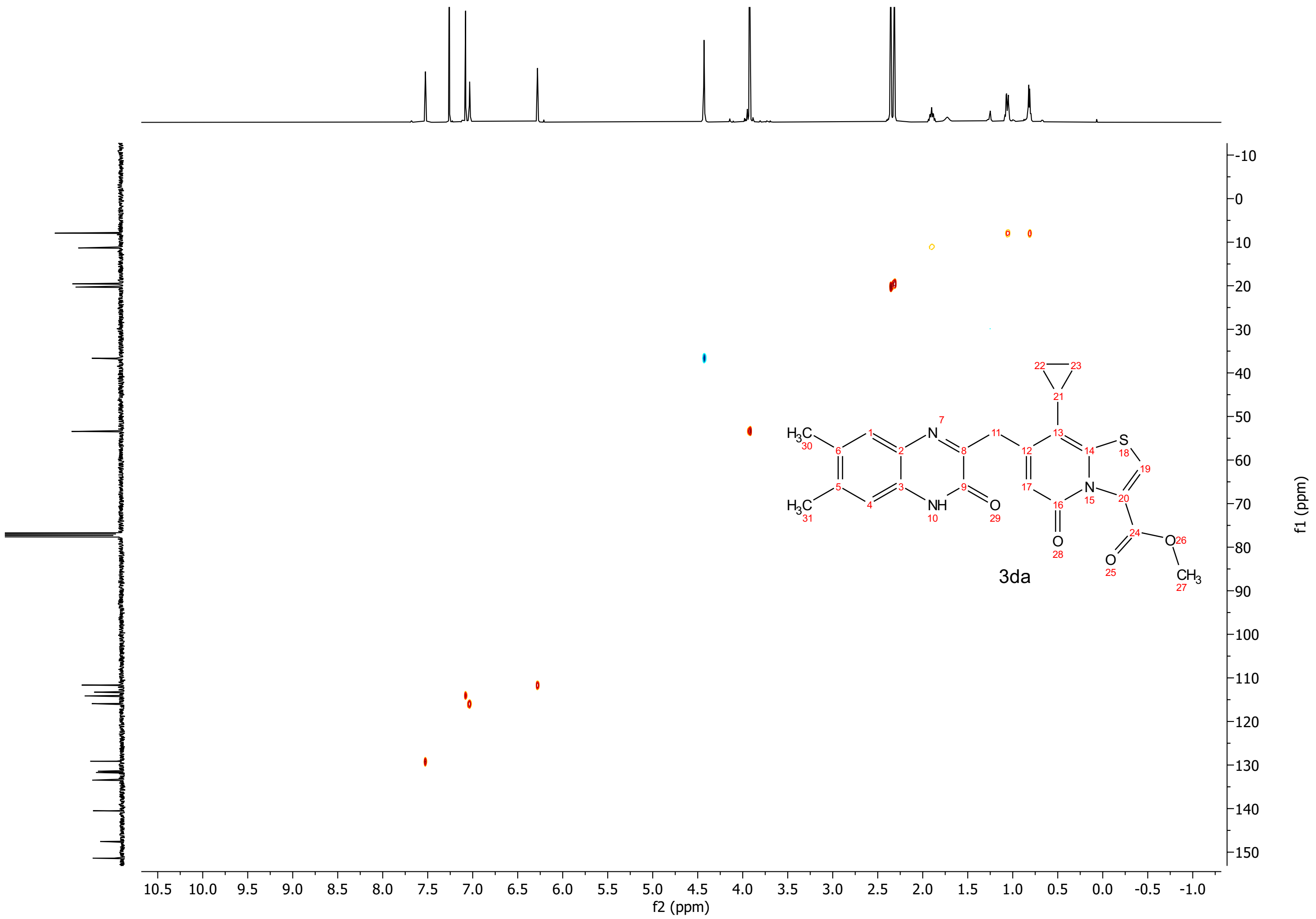

Figure S.82: gHSQC spectrum ($CDCl_3$) of methyl 8-cyclopropyl-7-((6,7-dimethyl-3-oxo-3,4-dihydroquinoxalin-2-yl)methyl)-5-oxo-5H-thiazolo[3,2-a]pyridine-3-carboxylate, **3da**.

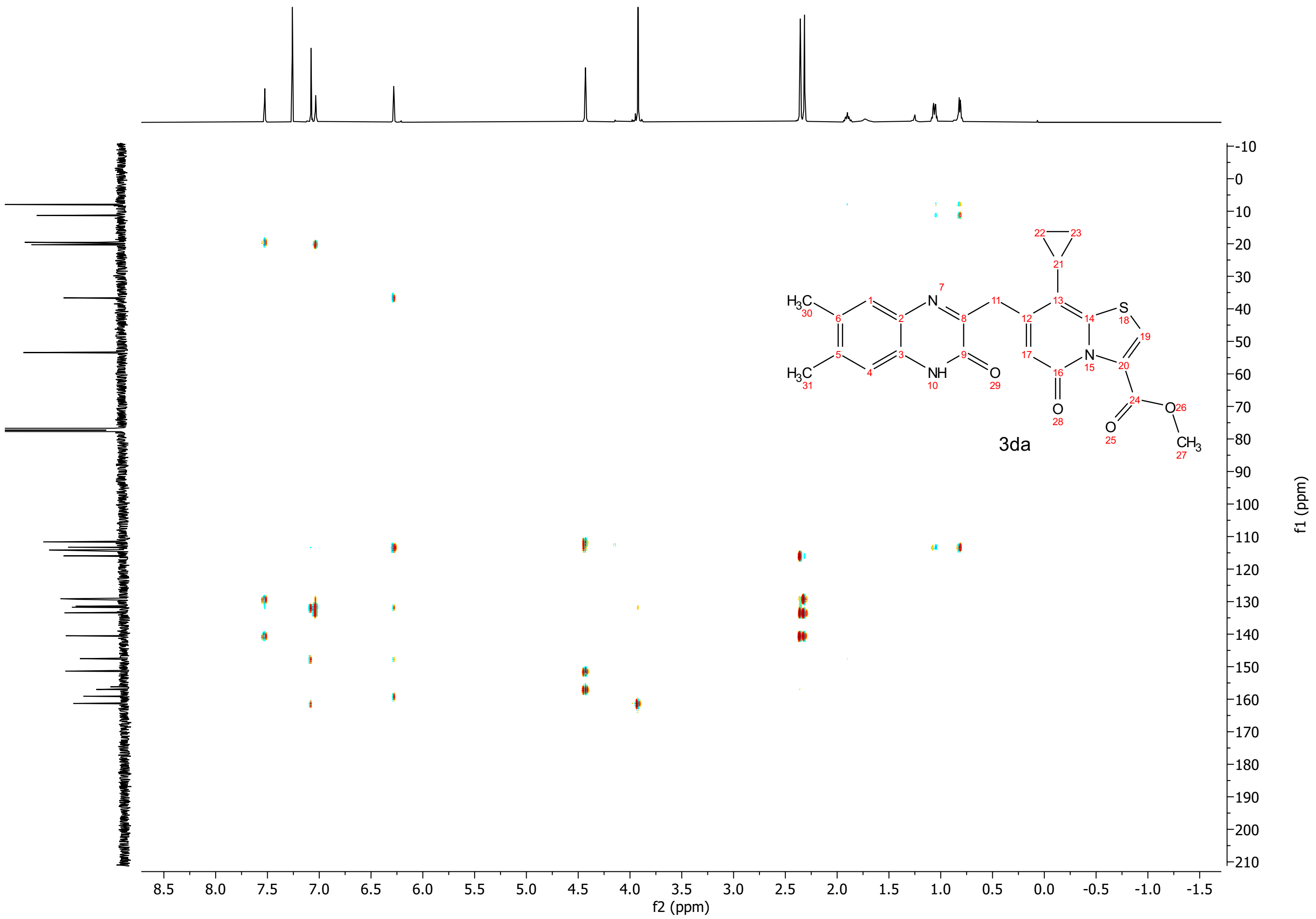

Figure S.83: gHMBC spectrum ($CDCl_3$) of methyl 8-cyclopropyl-7-((6,7-dimethyl-3-oxo-3,4-dihydroquinoxalin-2-yl)methyl)-5-oxo-5H-thiazolo[3,2-a]pyridine-3-carboxylate, **3da**.

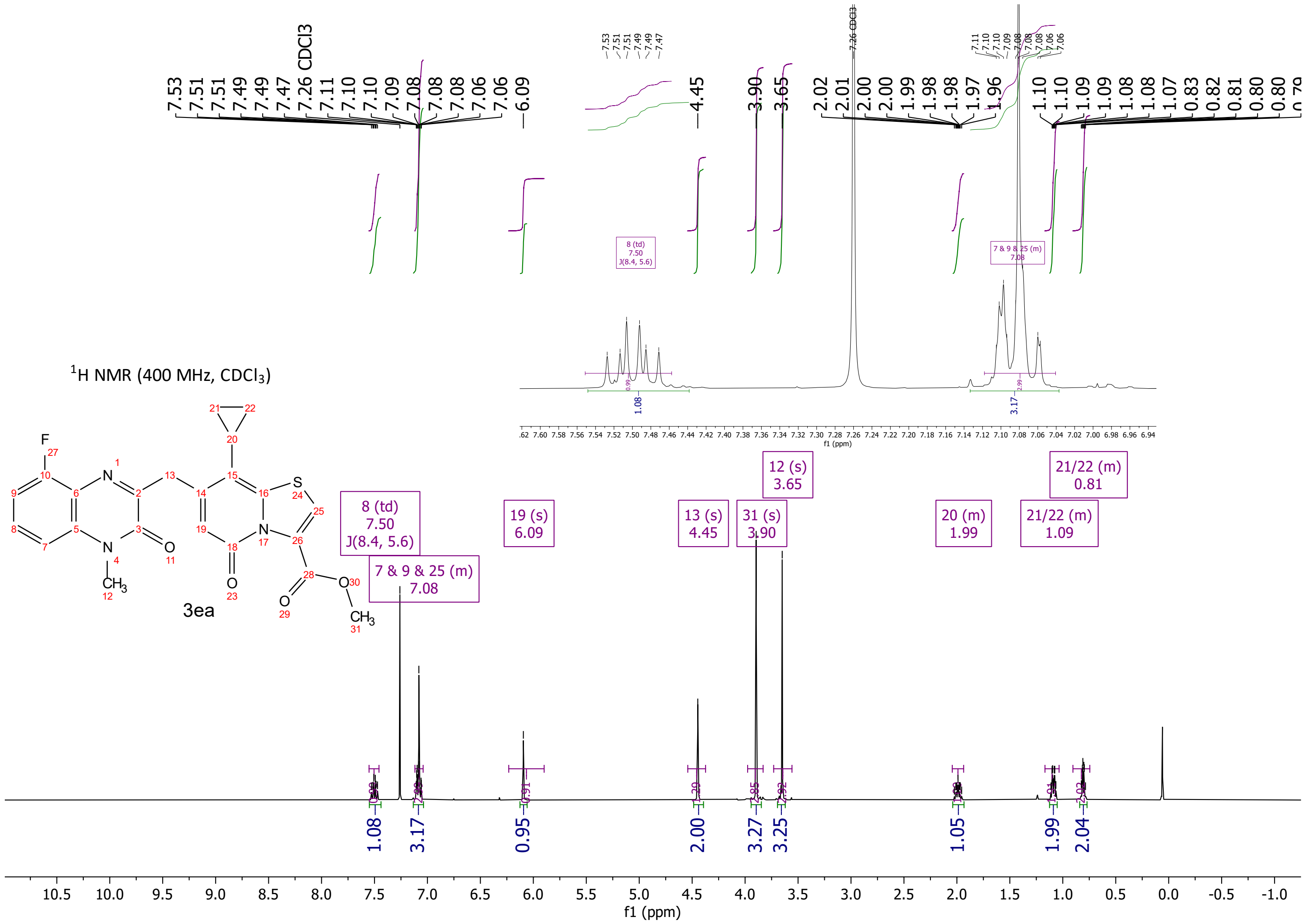

Figure S.84: ^{1}H NMR spectrum ($CDCl_3$, 400 MHz) of methyl 8-cyclopropyl-7-((8-fluoro-4-methyl-3-oxo-3,4-dihydroquinoxalin-2-yl)methyl)-5-oxo-5H-thiazolo[3,2-a]pyridine-3-carboxylate, **3ea**.

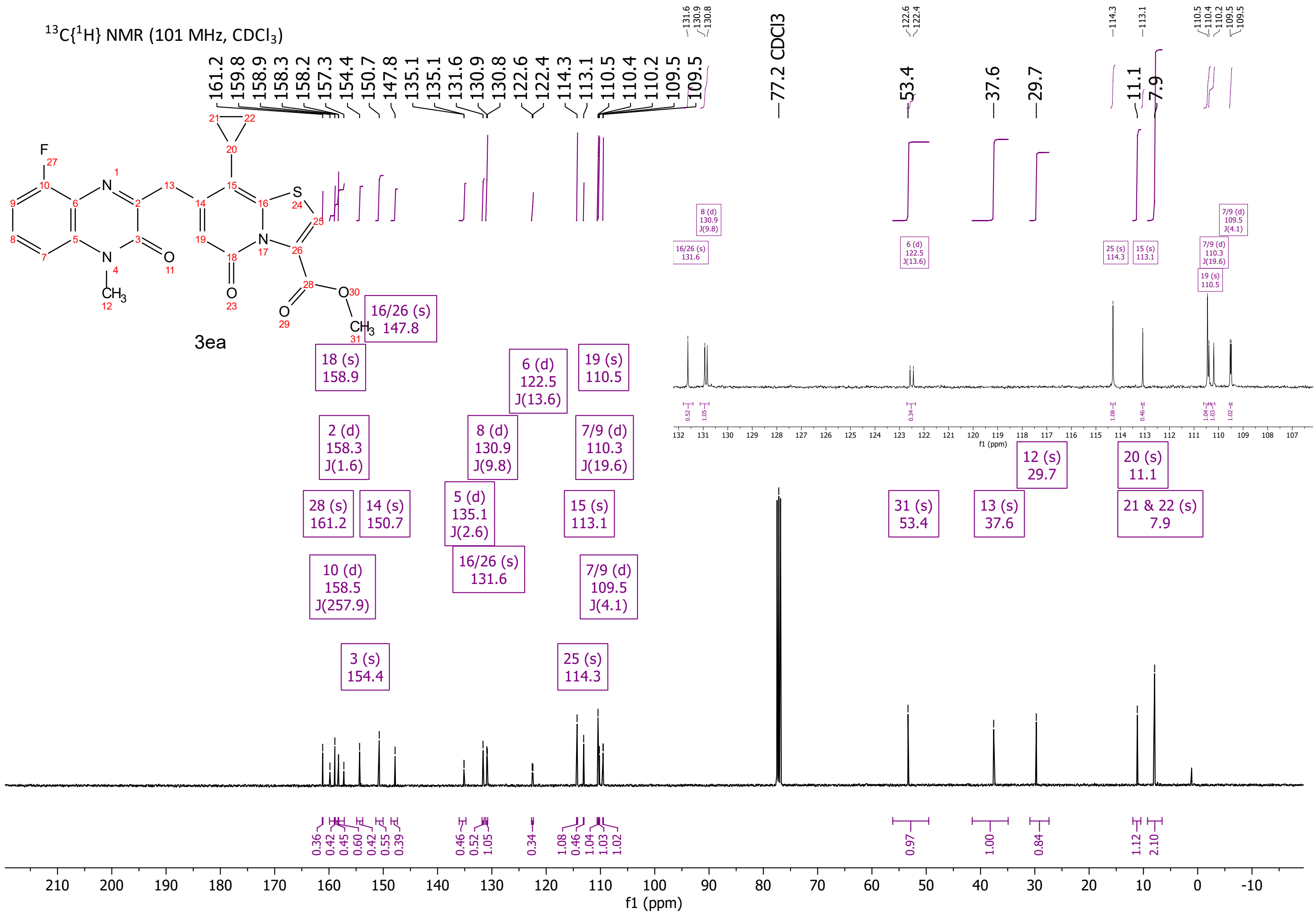

Figure S.85: $^{13}C\{^1H\}$ NMR spectrum ($CDCl_3$, 101 MHz) of methyl 8-cyclopropyl-7-((8-fluoro-4-methyl-3-oxo-3,4-dihydroquinoxalin-2-yl)methyl)-5-oxo-5H-thiazolo[3,2-a]pyridine-3-carboxylate, **3ea**.

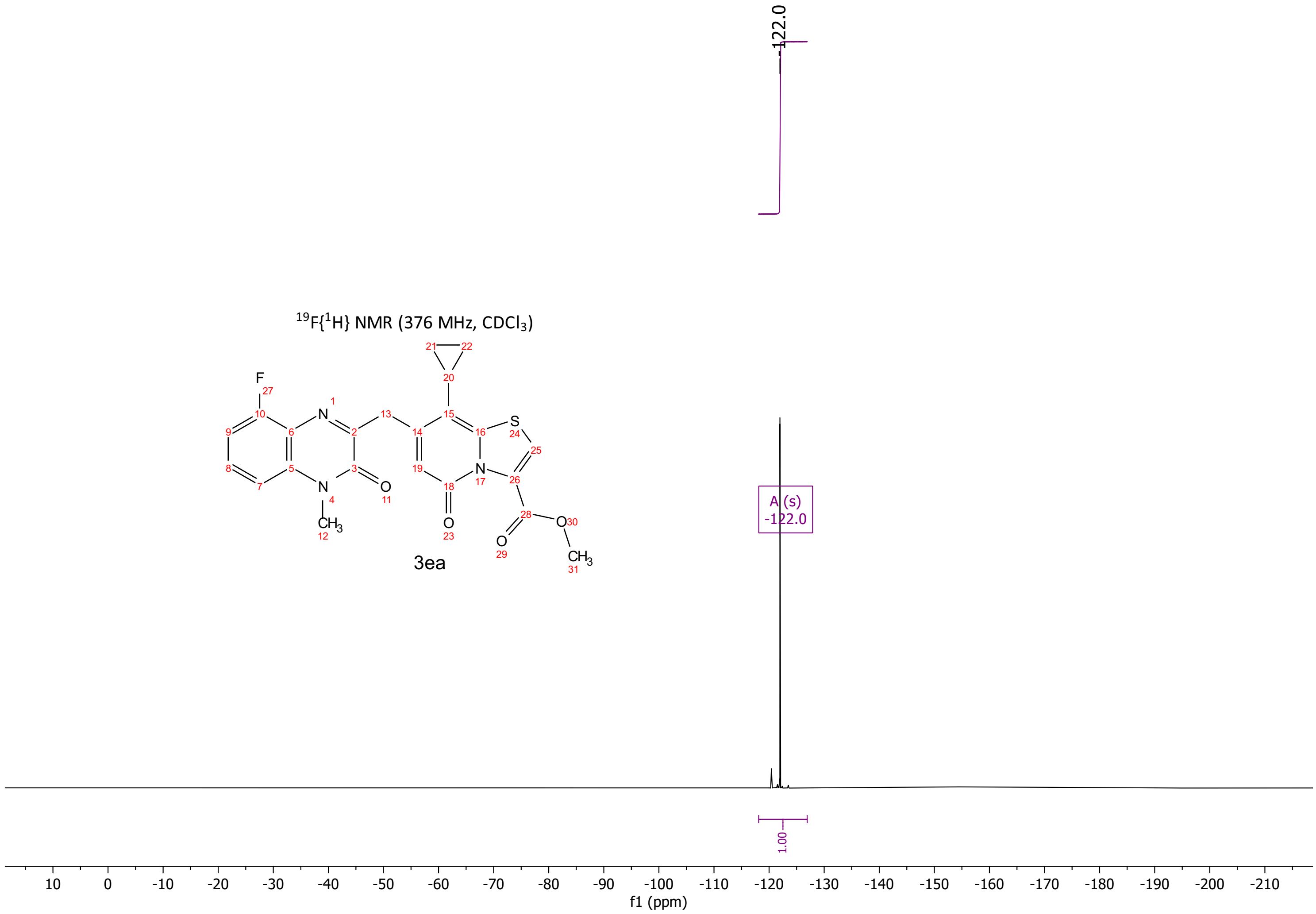

Figure S.86: $^{19}F\{^1H\}$ NMR spectrum ($CDCl_3$, 376 MHz) of methyl 8-cyclopropyl-7-((8-fluoro-4-methyl-3-oxo-3,4-dihydroquinoxalin-2-yl)methyl)-5-oxo-5H-thiazolo[3,2-a]pyridine-3-carboxylate, **3ea**.

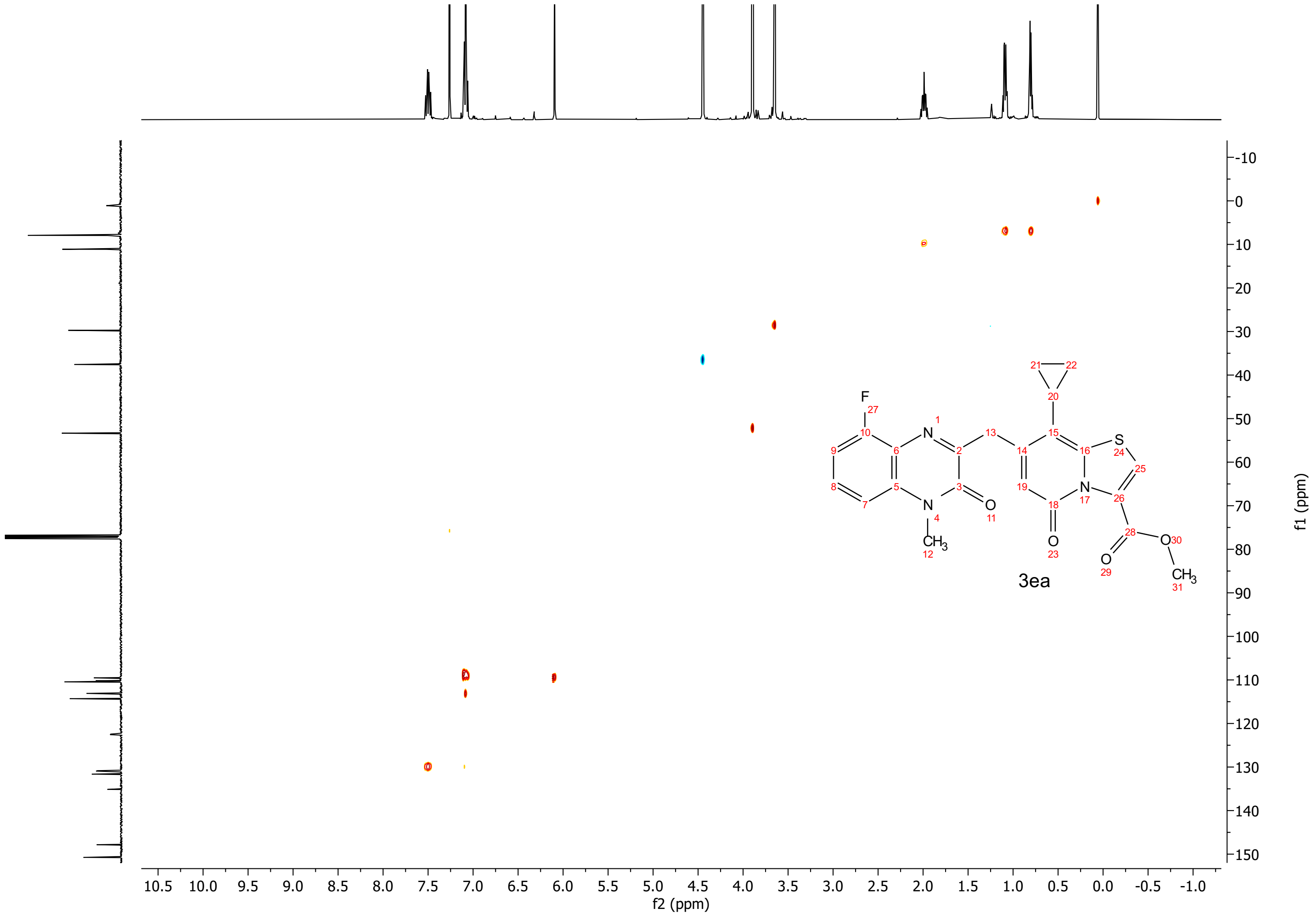

Figure S.87: gHSQC spectrum ($CDCl_3$) of methyl 8-cyclopropyl-7-((8-fluoro-4-methyl-3-oxo-3,4-dihydroquinoxalin-2-yl)methyl)-5-oxo-5H-thiazolo[3,2-a]pyridine-3-carboxylate, **3ea**.

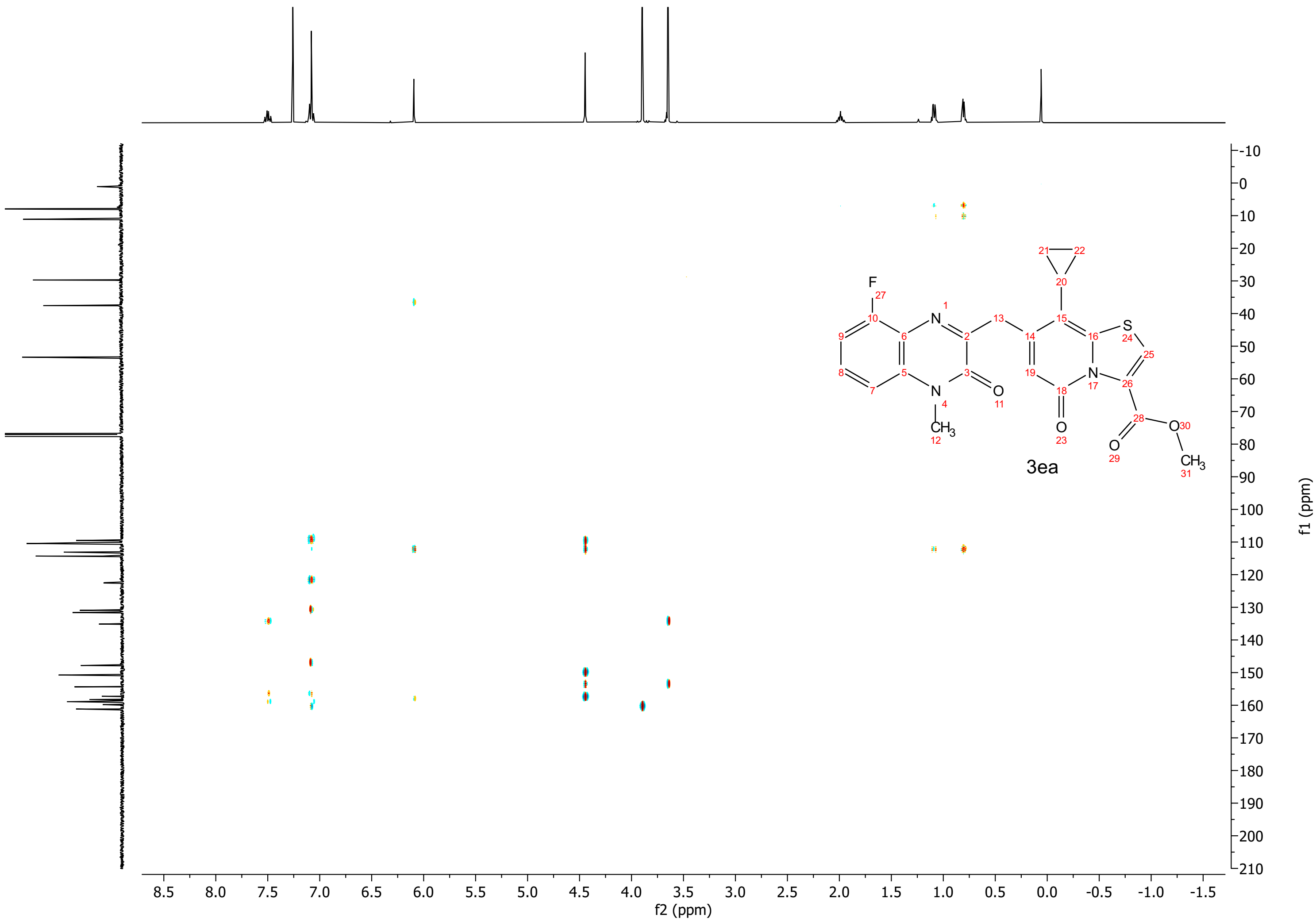

Figure S.88: gHMBC spectrum ($CDCl_3$) of methyl 8-cyclopropyl-7-((8-fluoro-4-methyl-3-oxo-3,4-dihydroquinoxalin-2-yl)methyl)-5-oxo-5H-thiazolo[3,2-a]pyridine-3-carboxylate, **3ea**.

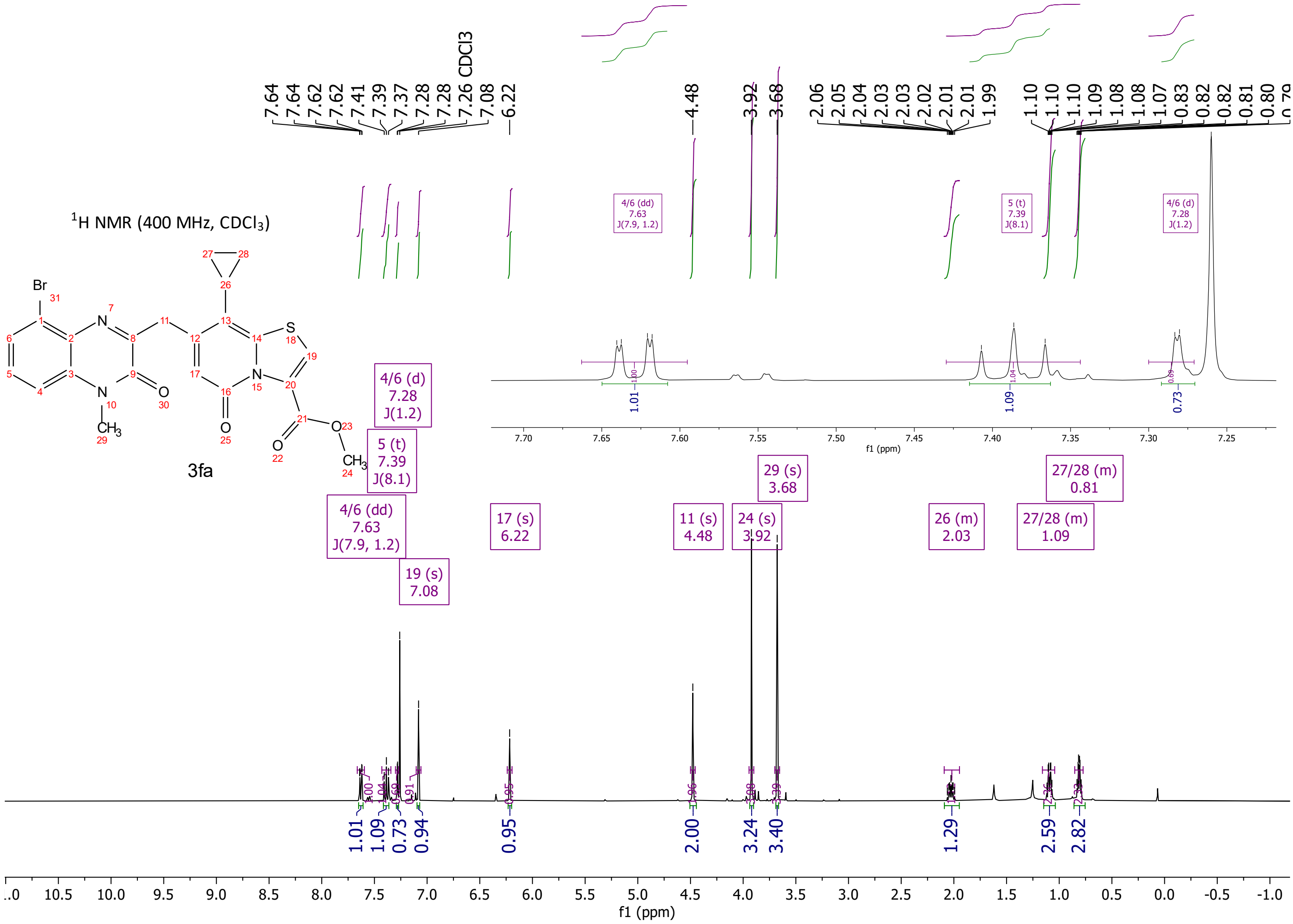

Figure S.89: ^{1}H NMR spectrum ($CDCl_3$, 400 MHz) of methyl 7-((8-bromo-4-methyl-3-oxo-3,4-dihydroquinoxalin-2-yl)methyl)-8-cyclopropyl-5-oxo-5H-thiazolo[3,2-a]pyridine-3-carboxylate, **3fa**.

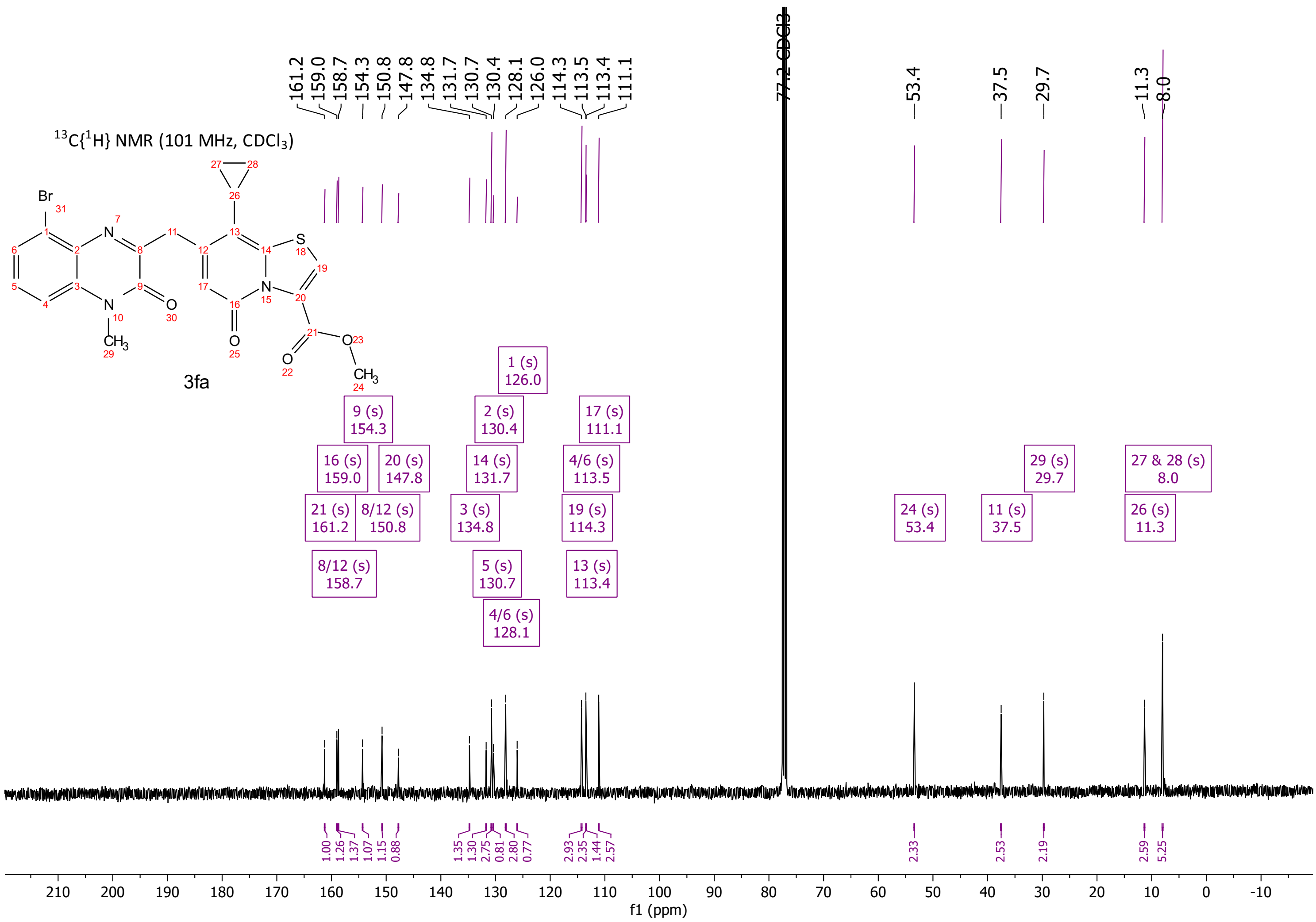

Figure S.90: $^{13}C\{^1H\}$ NMR spectrum ($CDCl_3$, 101 MHz) of methyl 7-((8-bromo-4-methyl-3-oxo-3,4-dihydroquinoxalin-2-yl)methyl)-8-cyclopropyl-5-oxo-5H-thiazolo[3,2-a]pyridine-3-carboxylate, **3fa**.

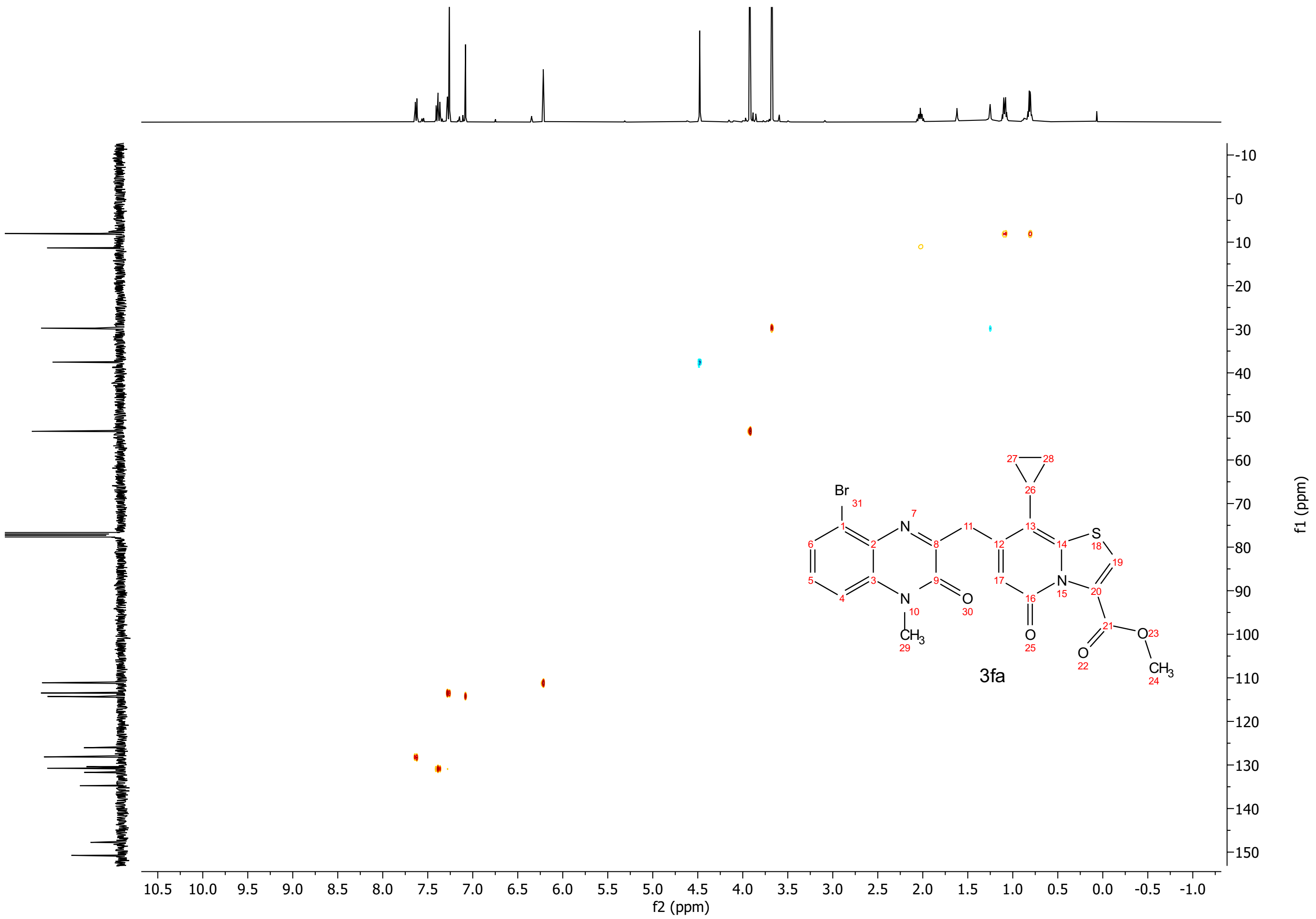

Figure S.91: gHSQC spectrum ($CDCl_3$) of methyl 7-((8-bromo-4-methyl-3-oxo-3,4-dihydroquinoxalin-2-yl)methyl)-8-cyclopropyl-5-oxo-5H-thiazolo[3,2-a]pyridine-3-carboxylate, **3fa**.

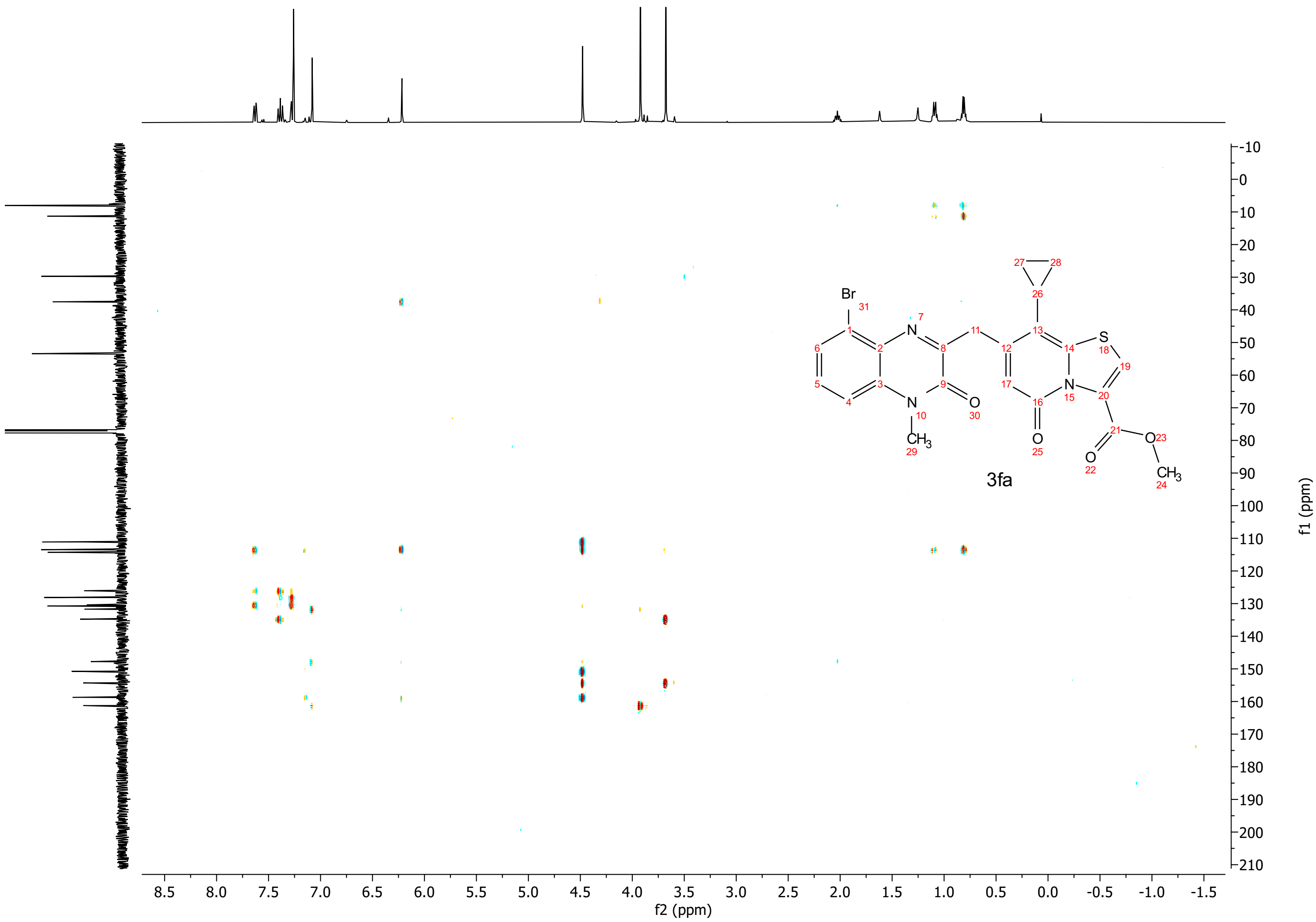

Figure S.92: gHMBC spectrum ($CDCl_3$) of methyl 7-((8-bromo-4-methyl-3-oxo-3,4-dihydroquinoxalin-2-yl)methyl)-8-cyclopropyl-5-oxo-5H-thiazolo[3,2-a]pyridine-3-carboxylate, **3fa**.

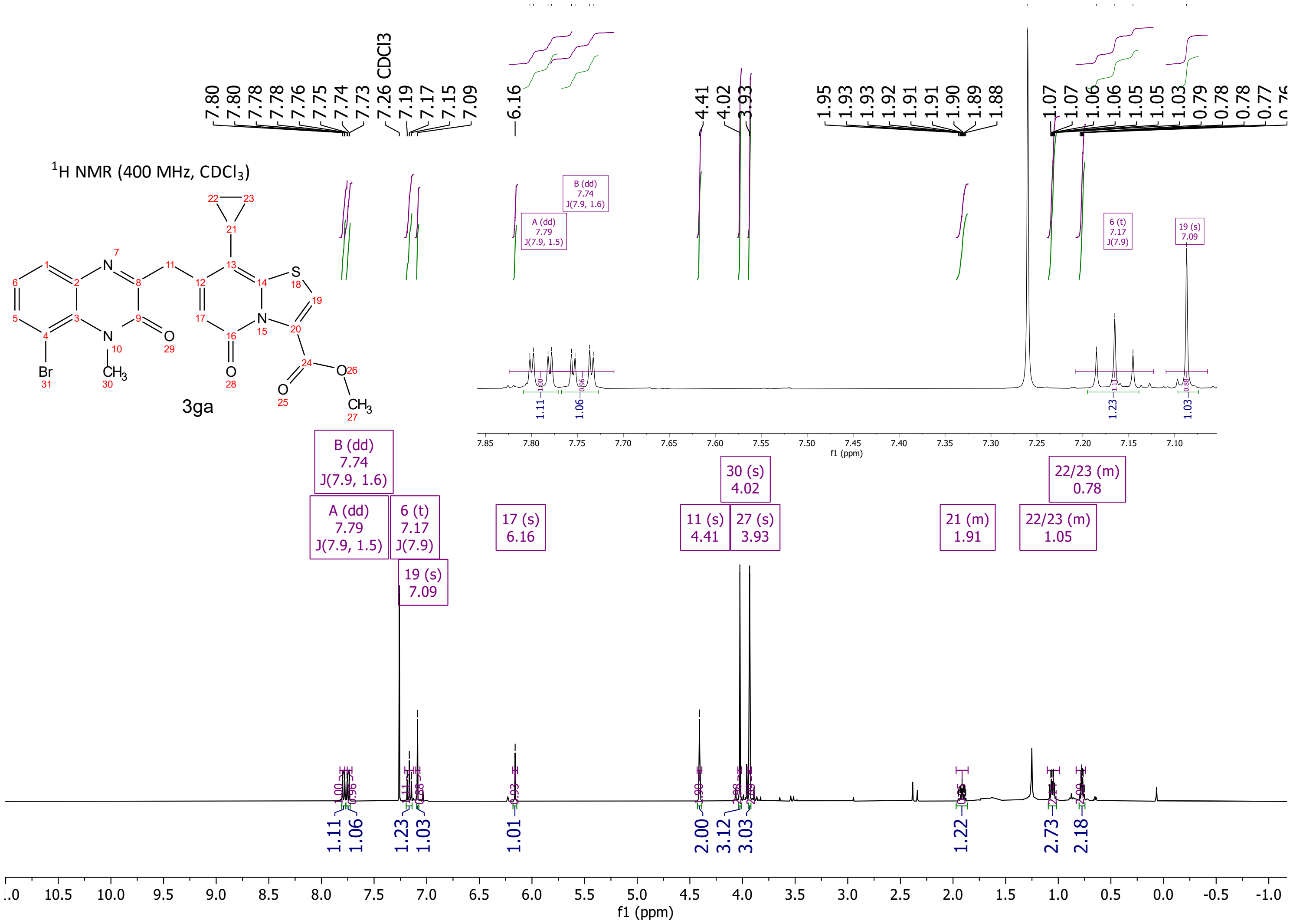

Figure S.93: ^{1}H NMR spectrum ($CDCl_3$, 400 MHz) of methyl 7-((5-bromo-4-methyl-3-oxo-3,4-dihydroquinoxalin-2-yl)methyl)-8-cyclopropyl-5-oxo-5H-thiazolo[3,2-a]pyridine-3-carboxylate, **3ga**.

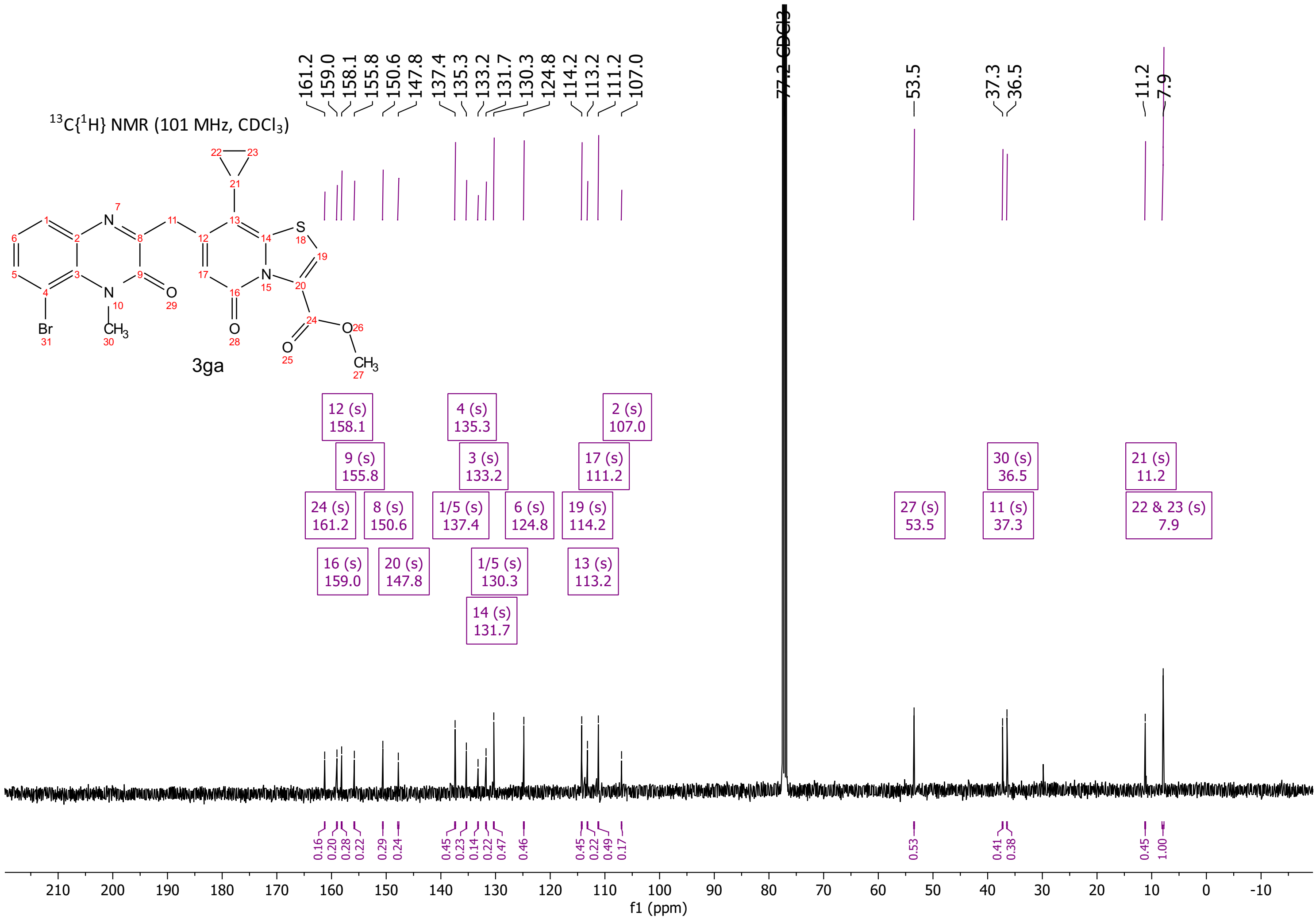

Figure S.94: $^{13}C\{^1H\}$ NMR spectrum ($CDCl_3$, 101 MHz) of methyl 7-((5-bromo-4-methyl-3-oxo-3,4-dihydroquinoxalin-2-yl)methyl)-8-cyclopropyl-5-oxo-5H-thiazolo[3,2-a]pyridine-3-carboxylate, **3ga**.

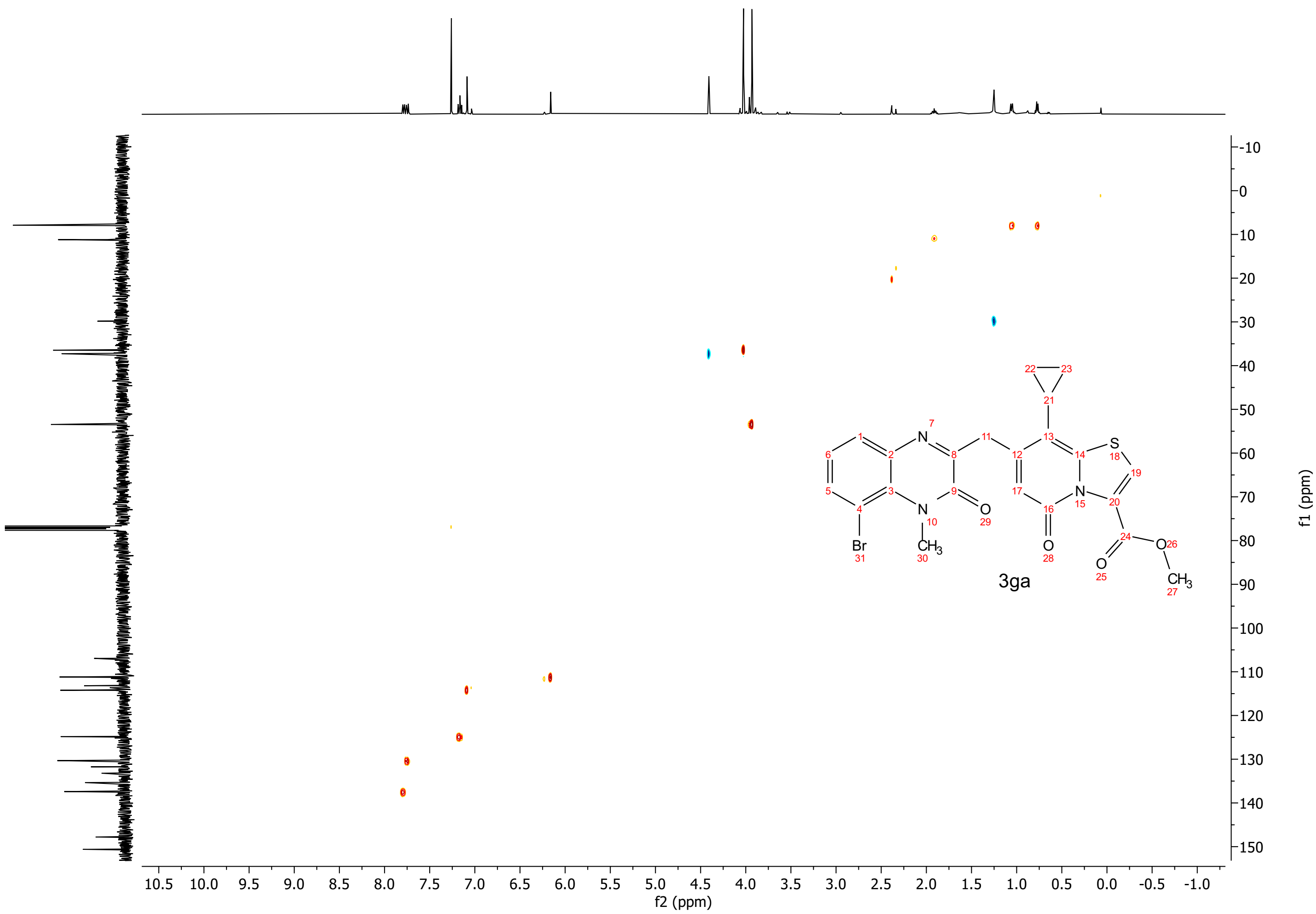

Figure S.95: gHSQC spectrum ($CDCl_3$) of methyl 7-((5-bromo-4-methyl-3-oxo-3,4-dihydroquinoxalin-2-yl)methyl)-8-cyclopropyl-5-oxo-5H-thiazolo[3,2-a]pyridine-3-carboxylate, **3ga**.

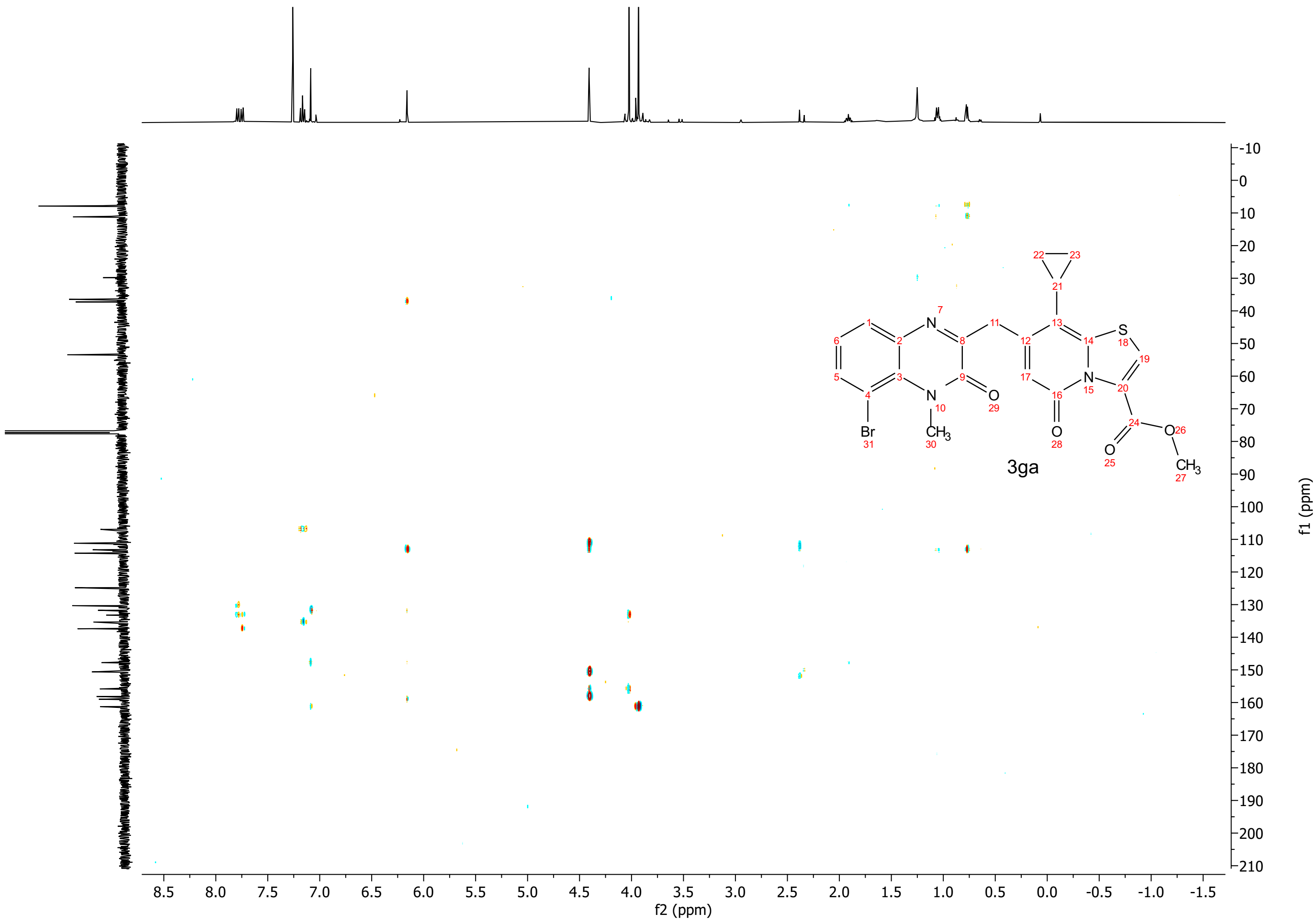

Figure S.96: gHMBC spectrum ($CDCl_3$) of methyl 7-((5-bromo-4-methyl-3-oxo-3,4-dihydroquinoxalin-2-yl)methyl)-8-cyclopropyl-5-oxo-5H-thiazolo[3,2-a]pyridine-3-carboxylate, **3ga**.

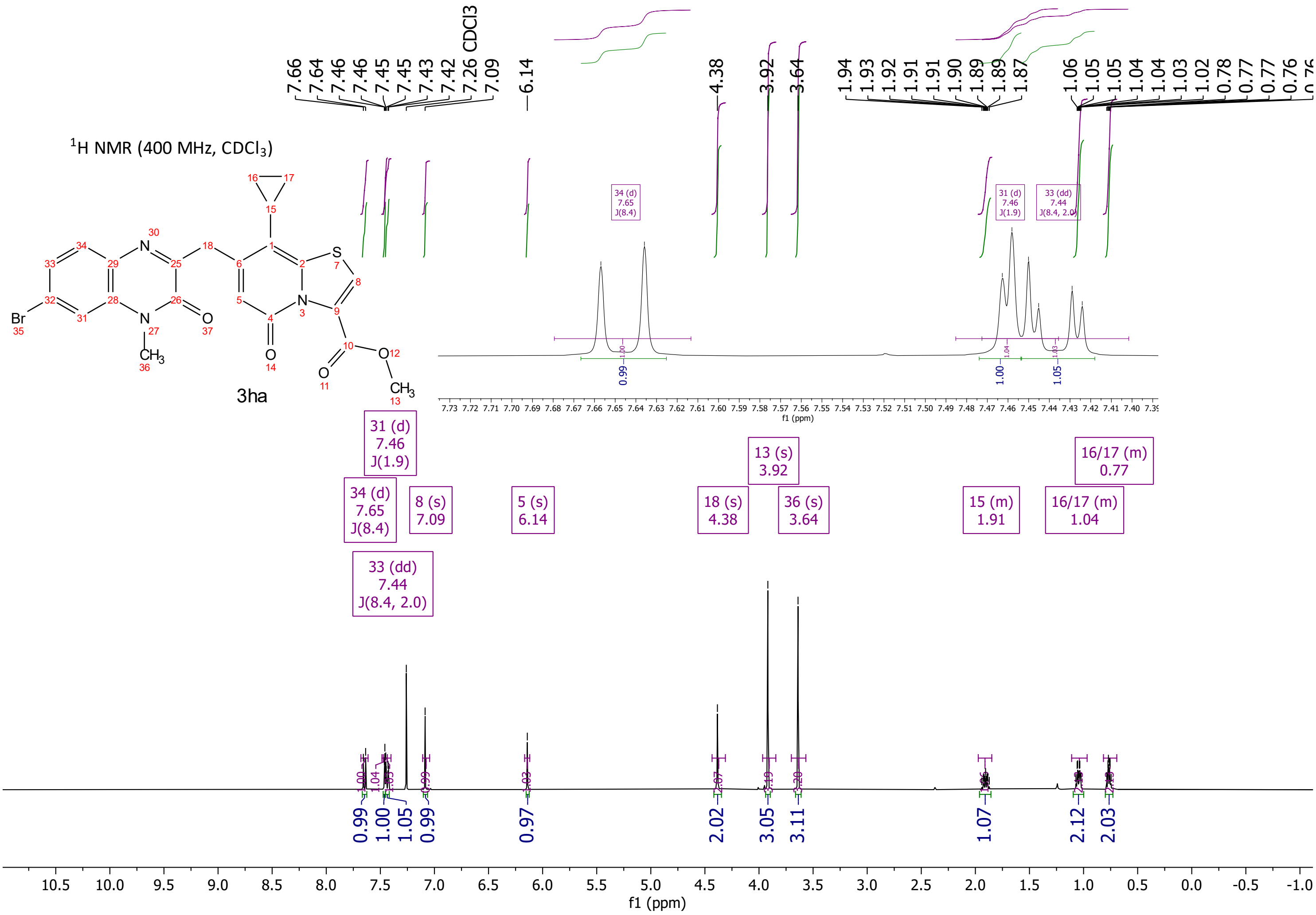

Figure S.97: ^{1}H NMR spectrum ($CDCl_3$, 400 MHz) of methyl 7-((6-bromo-4-methyl-3-oxo-3,4-dihydroquinoxalin-2-yl)methyl)-8-cyclopropyl-5-oxo-5H-thiazolo[3,2-a]pyridine-3-carboxylate, **3ha**.

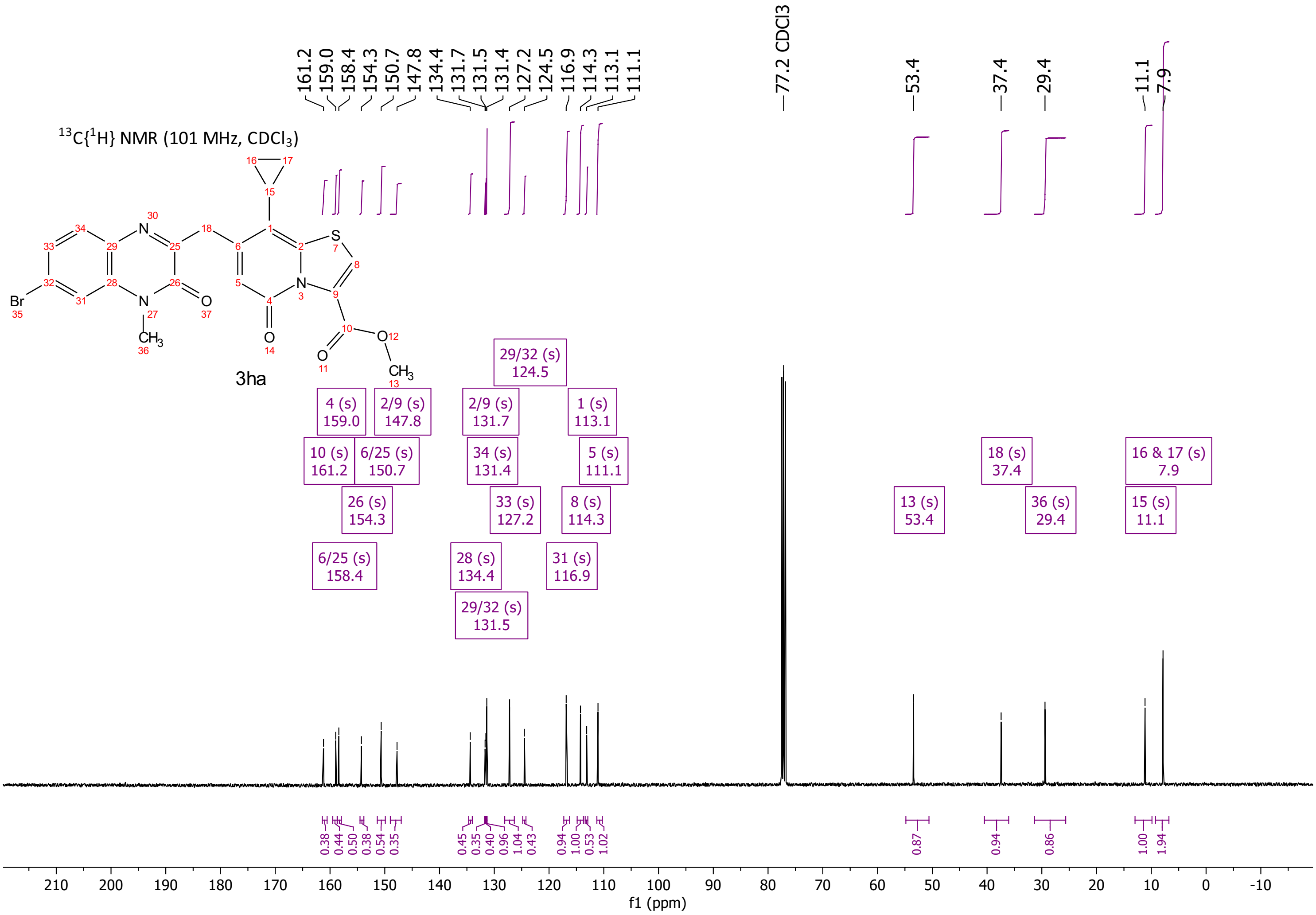

Figure S.98: ^{13}C{^{1}H} NMR spectrum (CDCl$_3$, 101 MHz) of methyl 7-((6-bromo-4-methyl-3-oxo-3,4-dihydroquinoxalin-2-yl)methyl)-8-cyclopropyl-5-oxo-5H-thiazolo[3,2-a]pyridine-3-carboxylate, **3ha**.

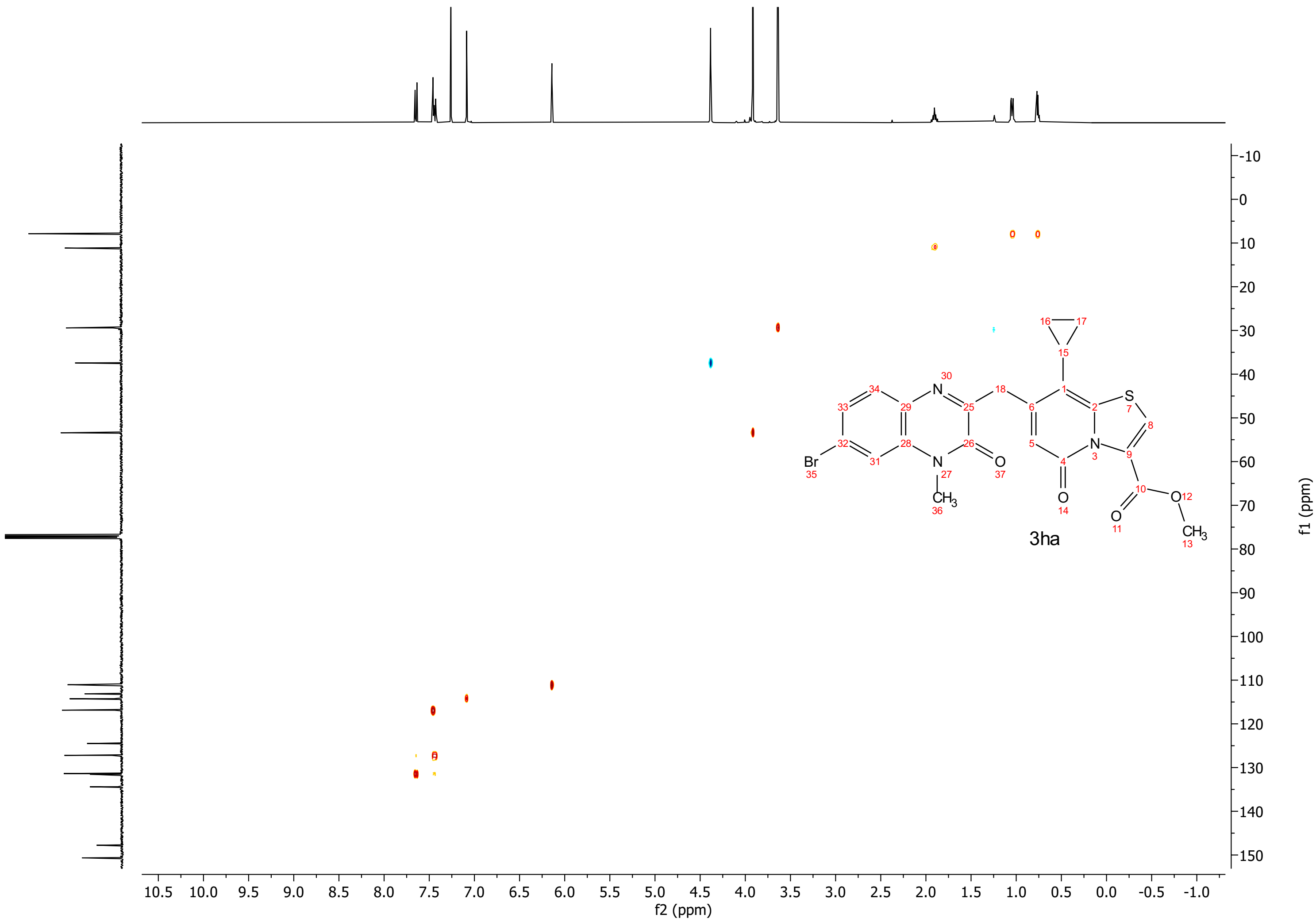

Figure S.99: gHSQC spectrum ($CDCl_3$) of methyl 7-((6-bromo-4-methyl-3-oxo-3,4-dihydroquinoxalin-2-yl)methyl)-8-cyclopropyl-5-oxo-5H-thiazolo[3,2-a]pyridine-3-carboxylate, **3ha**.

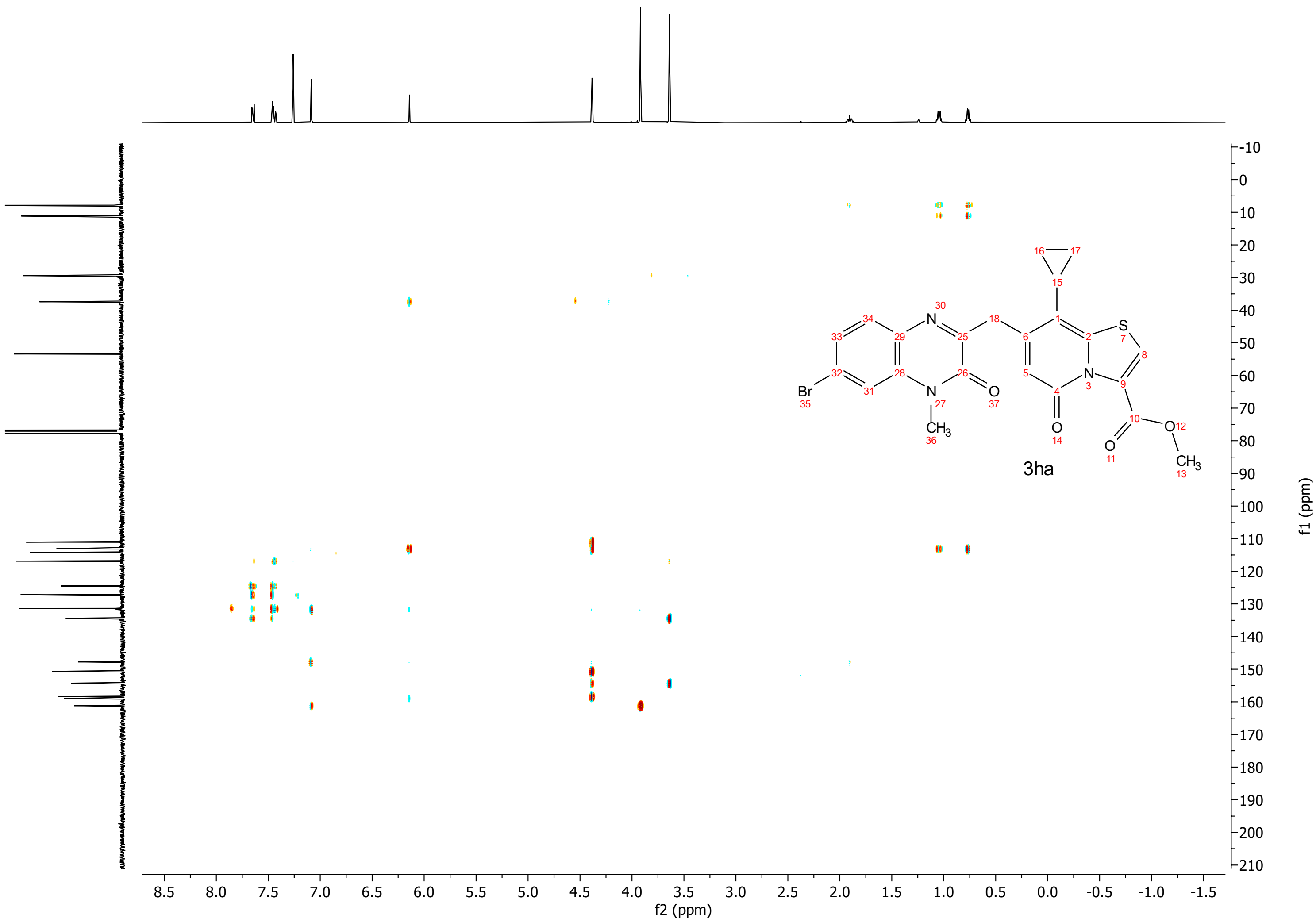

Figure S.100: gHMBC spectrum ($CDCl_3$) of methyl 7-((6-bromo-4-methyl-3-oxo-3,4-dihydroquinoxalin-2-yl)methyl)-8-cyclopropyl-5-oxo-5H-thiazolo[3,2-a]pyridine-3-carboxylate, **3ha**.

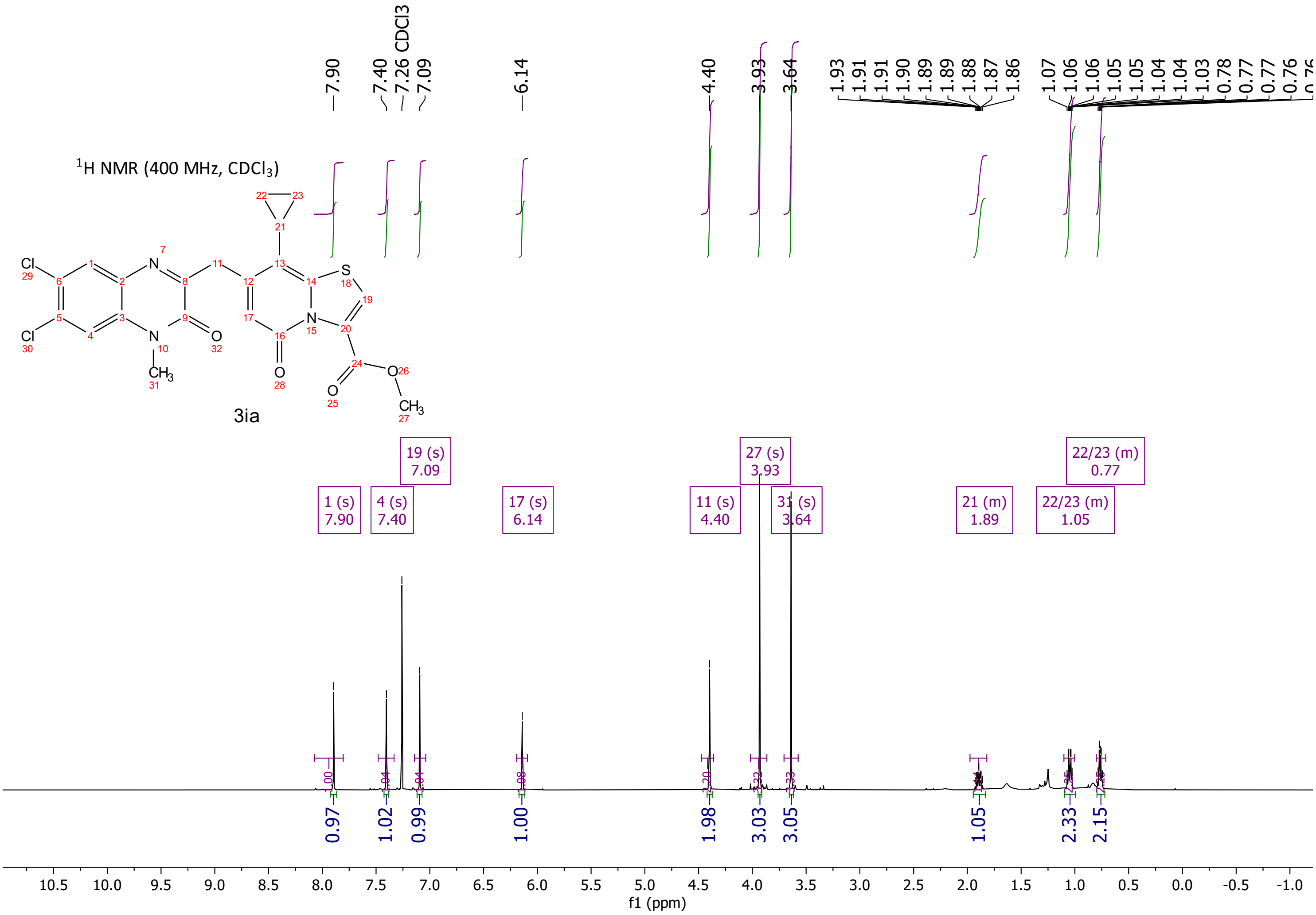

Figure S.101: ^{1}H NMR spectrum ($CDCl_3$, 400 MHz) of methyl 8-cyclopropyl-7-((6,7-dichloro-4-methyl-3-oxo-3,4-dihydroquinoxalin-2-yl)methyl)-5-oxo-5H-thiazolo[3,2-a]pyridine-3-carboxylate, **3ia**.

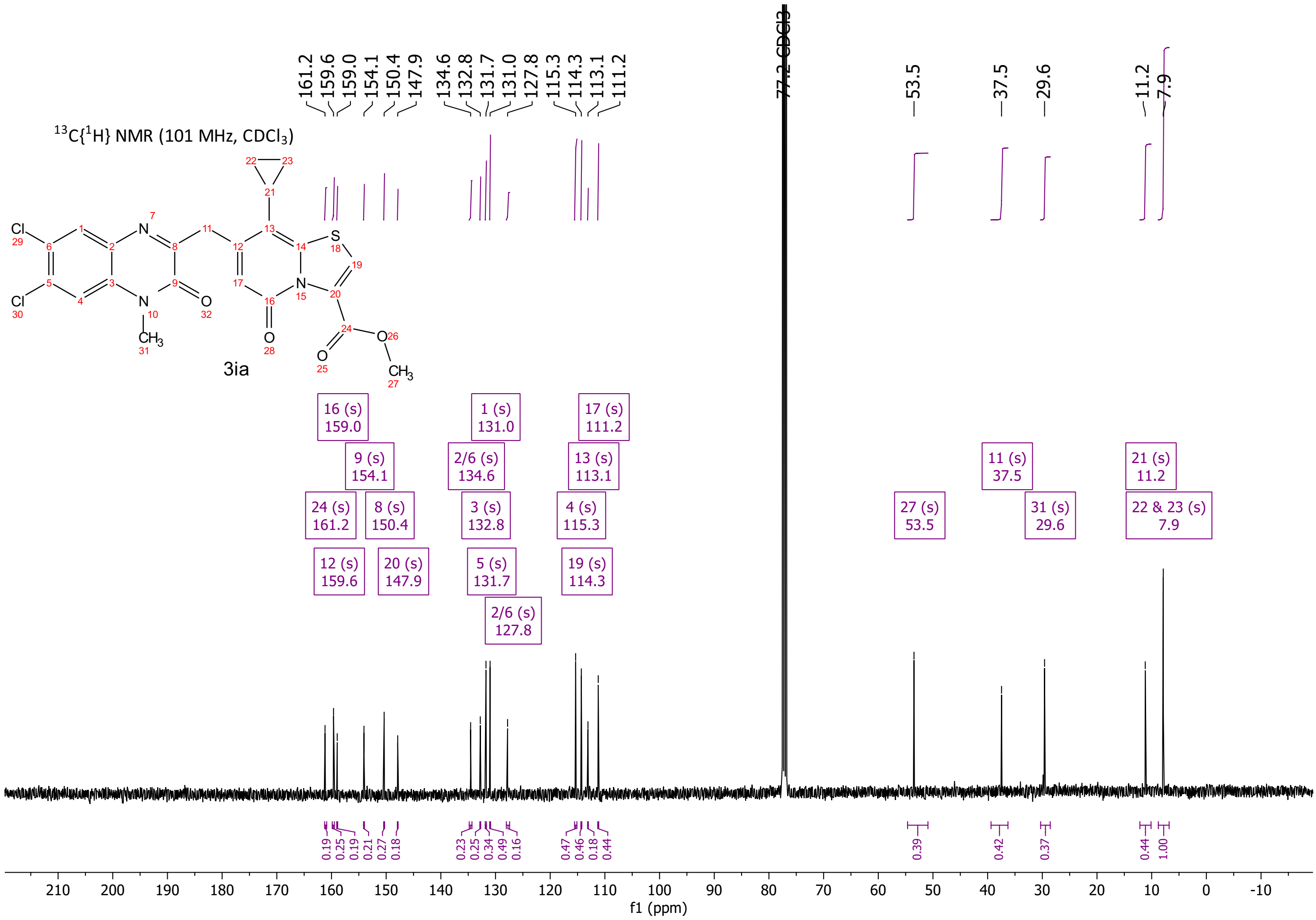

Figure S.102: $^{13}C\{^1H\}$ NMR spectrum ($CDCl_3$, 101 MHz) of methyl 8-cyclopropyl-7-((6,7-dichloro-4-methyl-3-oxo-3,4-dihydroquinoxalin-2-yl)methyl)-5-oxo-5H-thiazolo[3,2-a]pyridine-3-carboxylate, **3ia**.

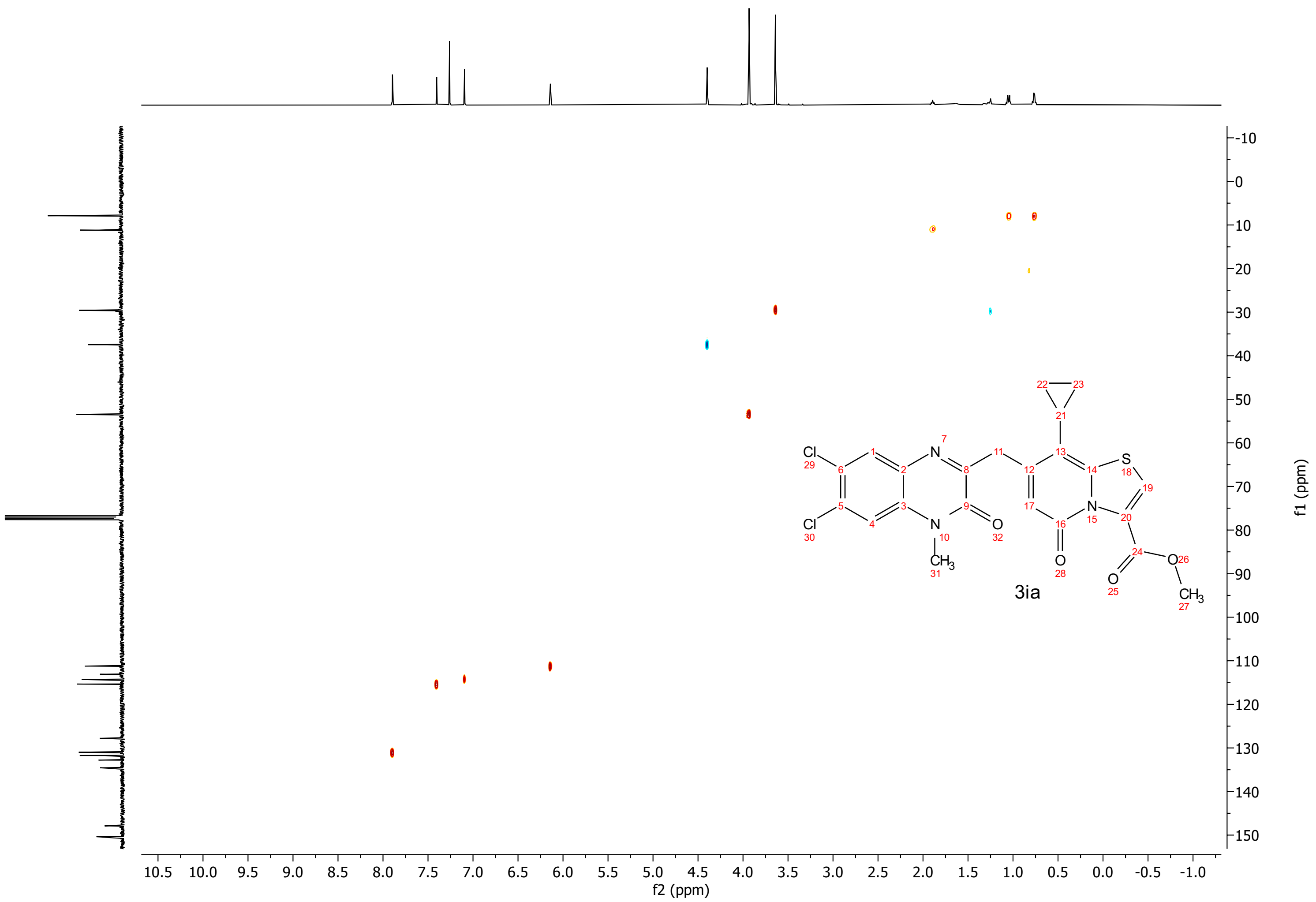

Figure S.103: gHSQC spectrum ($CDCl_3$) of methyl 8-cyclopropyl-7-((6,7-dichloro-4-methyl-3-oxo-3,4-dihydroquinoxalin-2-yl)methyl)-5-oxo-5H-thiazolo[3,2-a]pyridine-3-carboxylate, **3ia**.

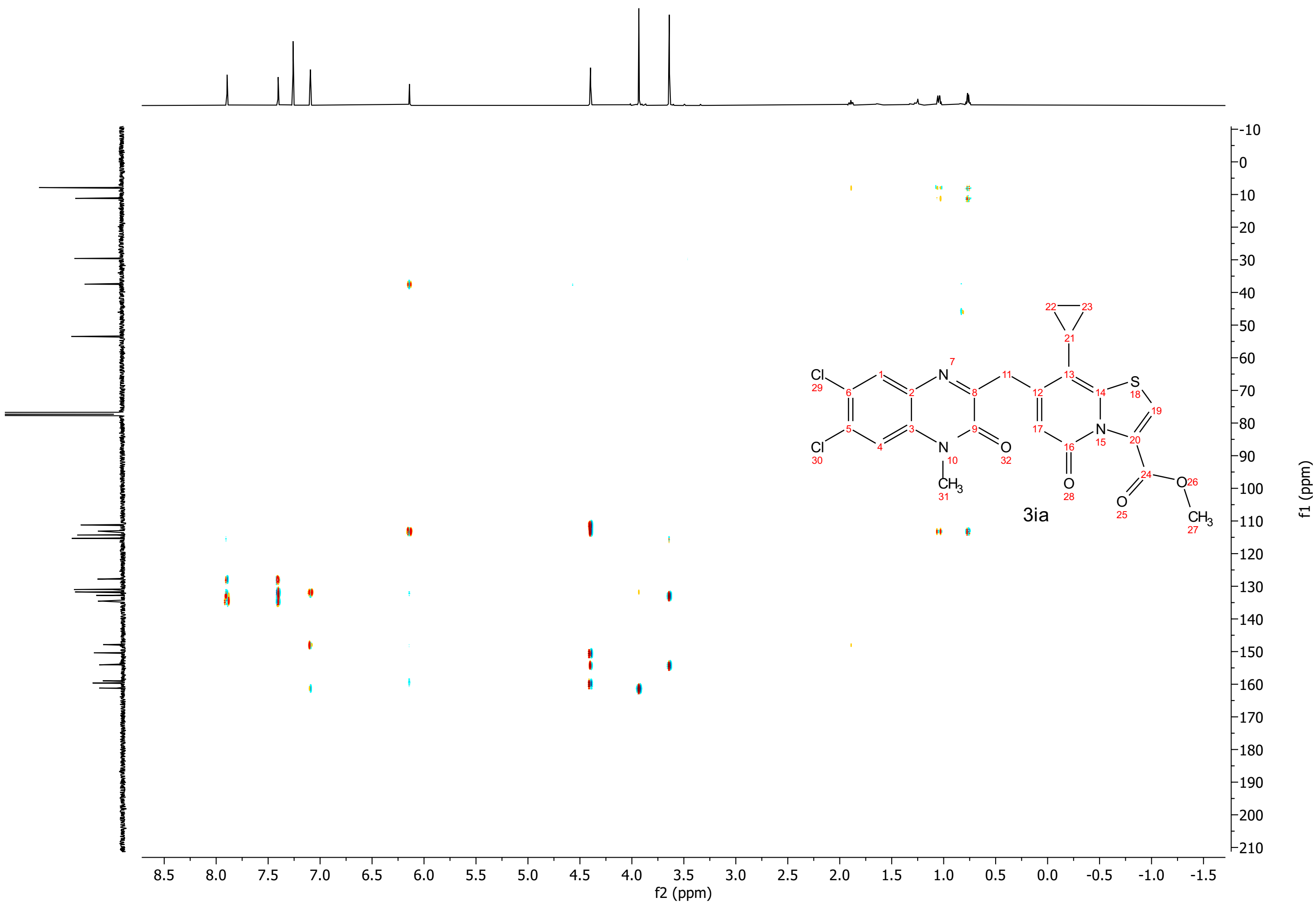

Figure S.104: gHMBC spectrum ($CDCl_3$) of methyl 8-cyclopropyl-7-((6,7-dichloro-4-methyl-3-oxo-3,4-dihydroquinoxalin-2-yl)methyl)-5-oxo-5H-thiazolo[3,2-a]pyridine-3-carboxylate, **3ia**.

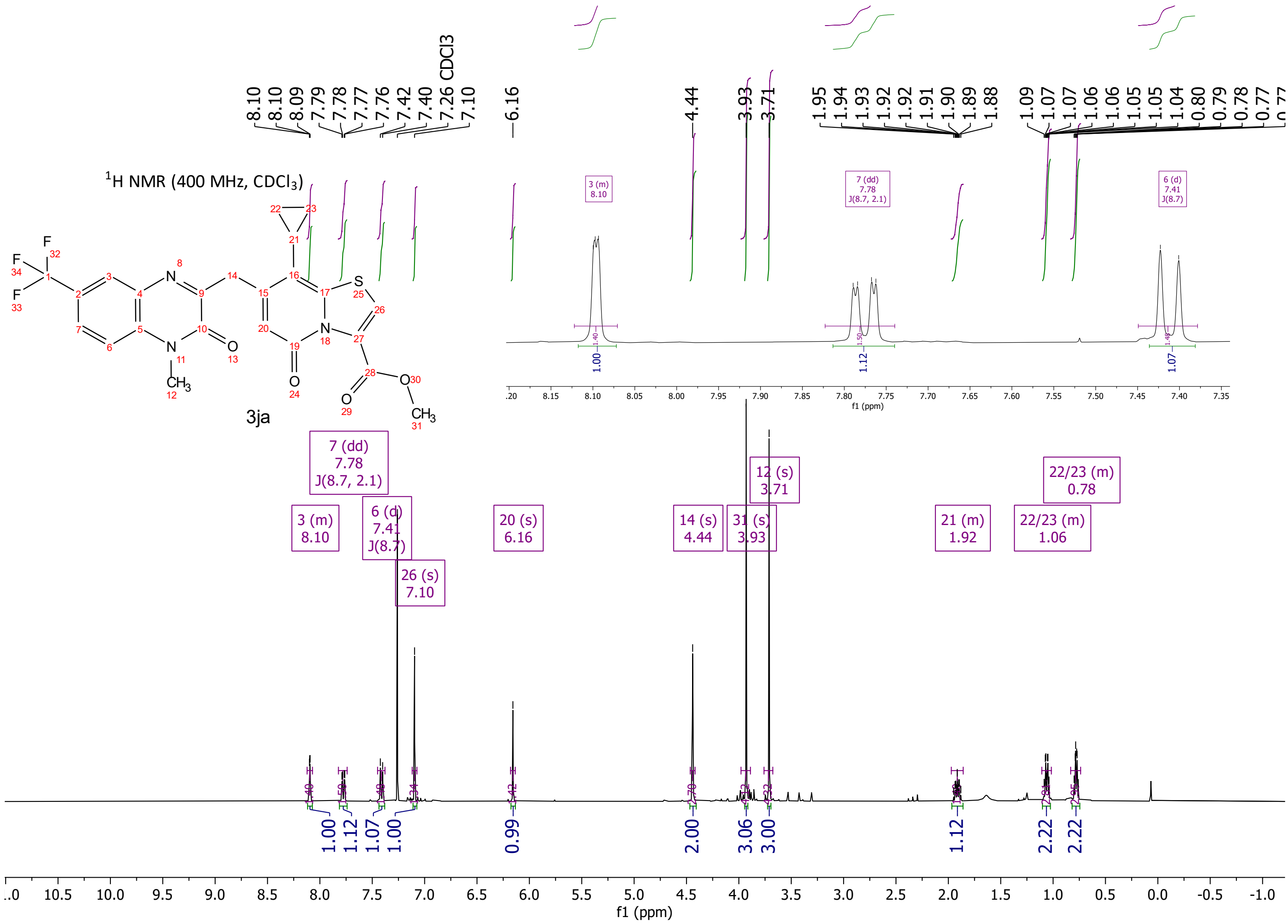

Figure S.105: ^{1}H NMR spectrum ($CDCl_3$, 400 MHz) of methyl 8-cyclopropyl-7-((4-methyl-3-oxo-7-(trifluoromethyl)-3,4-dihydroquinoxalin-2-yl)methyl)-5-oxo-5H-thiazolo[3,2-a]pyridine-3-carboxylate, **3ja**.

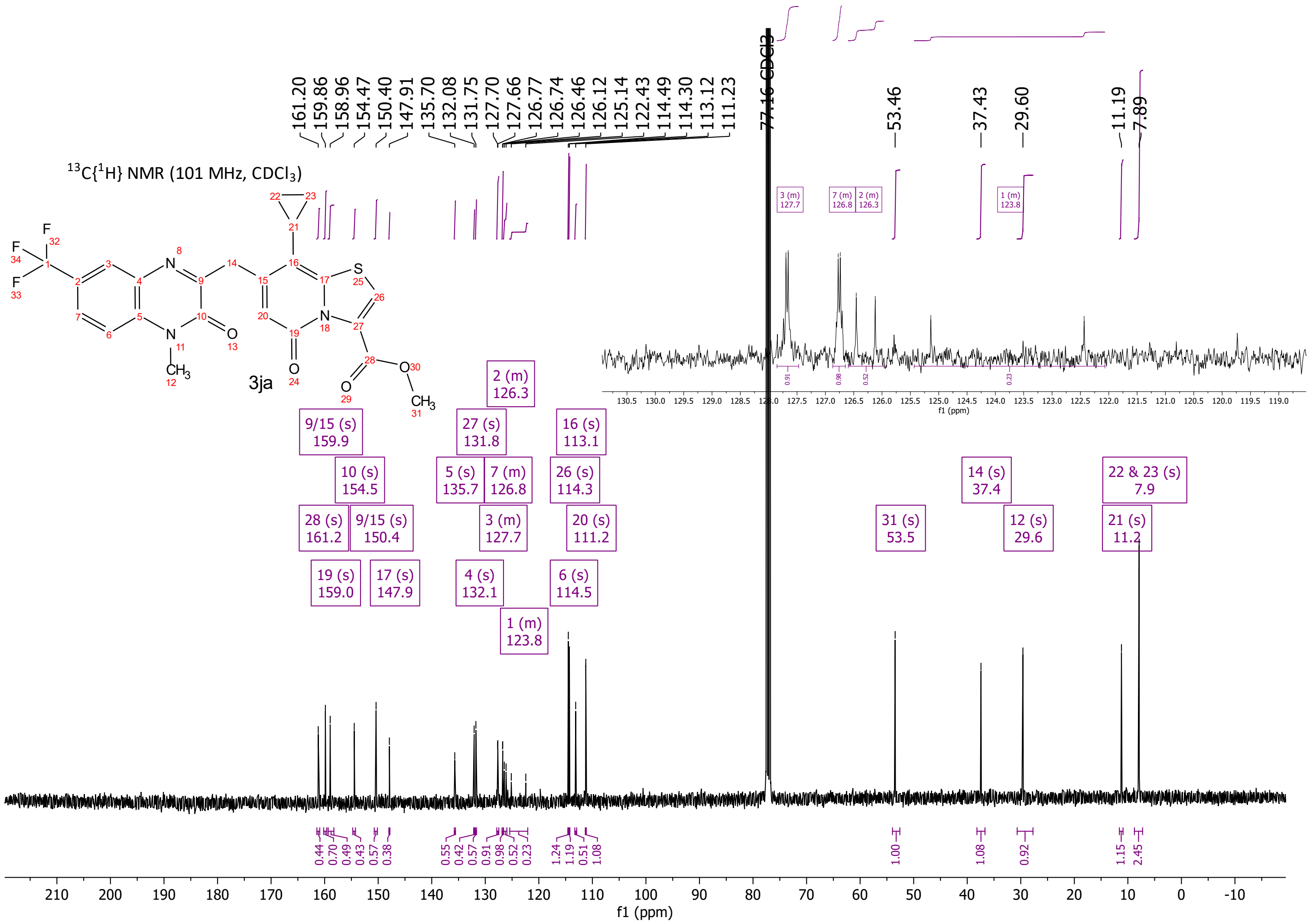

Figure S.106: $^{13}C\{^1H\}$ NMR spectrum ($CDCl_3$, 101 MHz) of methyl 8-cyclopropyl-7-((4-methyl-3-oxo-7-(trifluoromethyl)-3,4-dihydroquinoxalin-2-yl)methyl)-5-oxo-5H-thiazolo[3,2-a]pyridine-3-carboxylate, **3ja**.

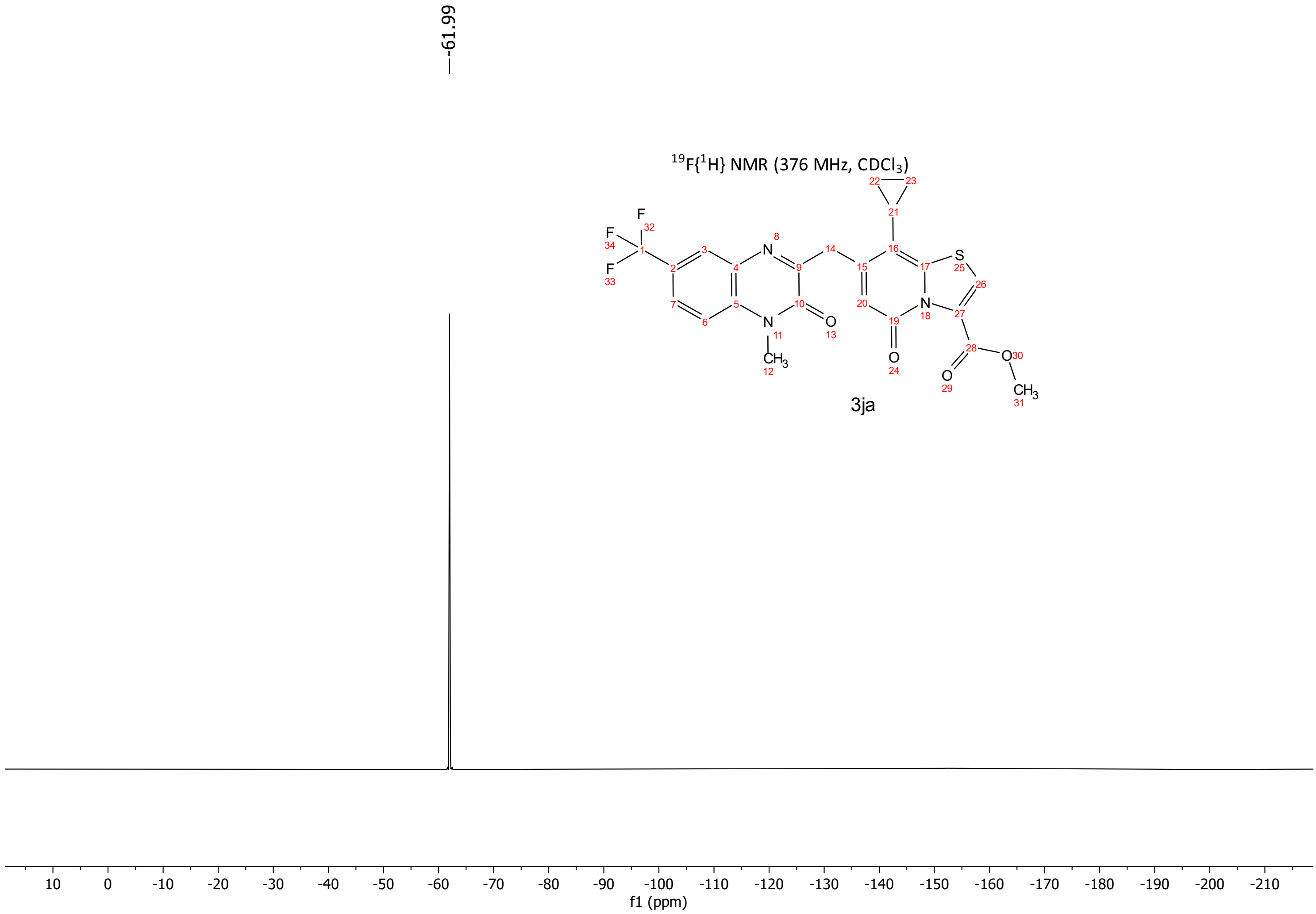

Figure S.107: ^{19}F{^{1}H} NMR spectrum (CDCl$_3$, 376 MHz) of methyl 8-cyclopropyl-7-((4-methyl-3-oxo-7-(trifluoromethyl)-3,4-dihydroquinoxalin-2-yl)methyl)-5-oxo-5H-thiazolo[3,2-a]pyridine-3-carboxylate, **3ja**.

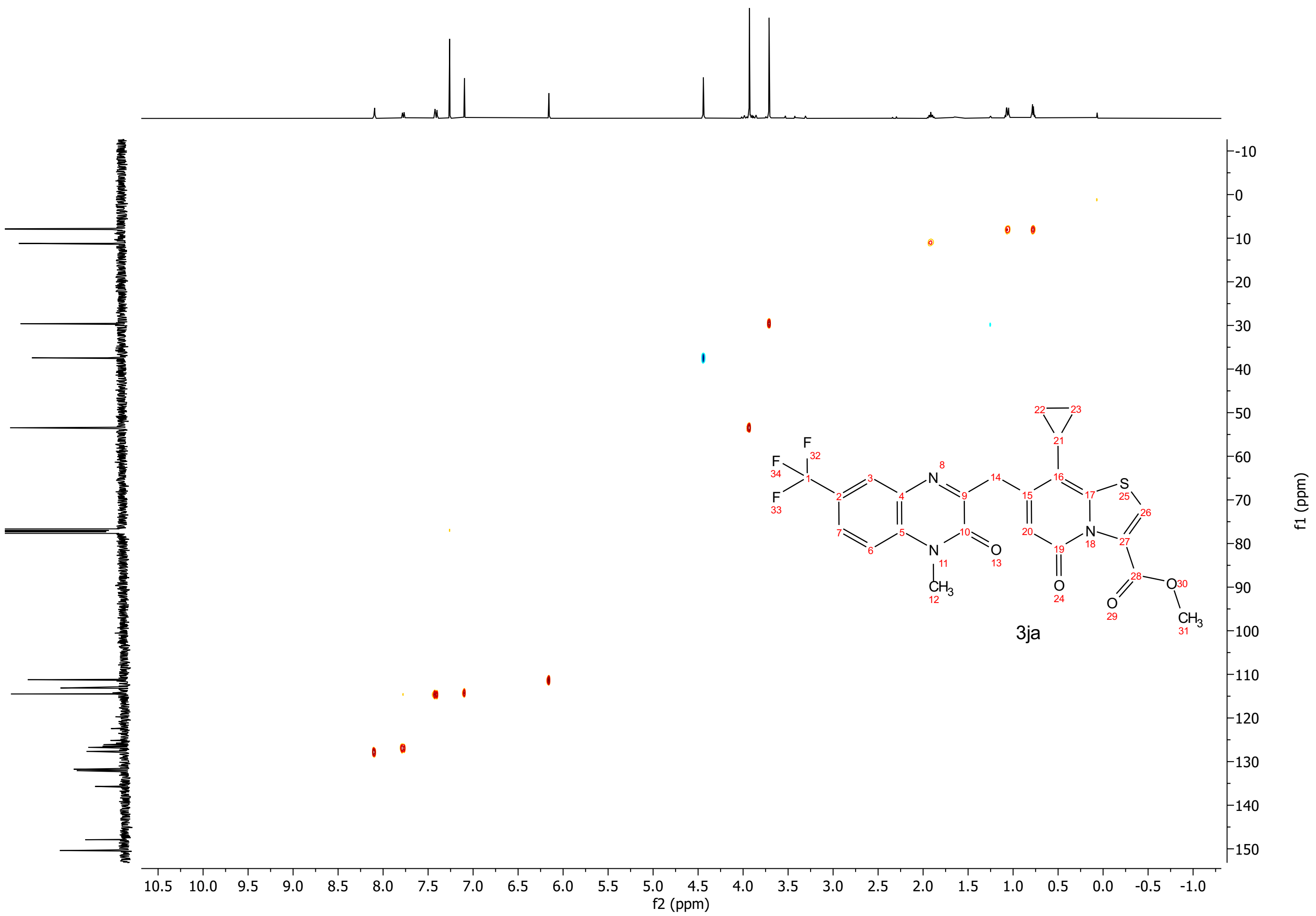

Figure S.108: gHSQC spectrum ($CDCl_3$) of methyl 8-cyclopropyl-7-((4-methyl-3-oxo-7-(trifluoromethyl)-3,4-dihydroquinoxalin-2-yl)methyl)-5-oxo-5H-thiazolo[3,2-a]pyridine-3-carboxylate, **3ja**.

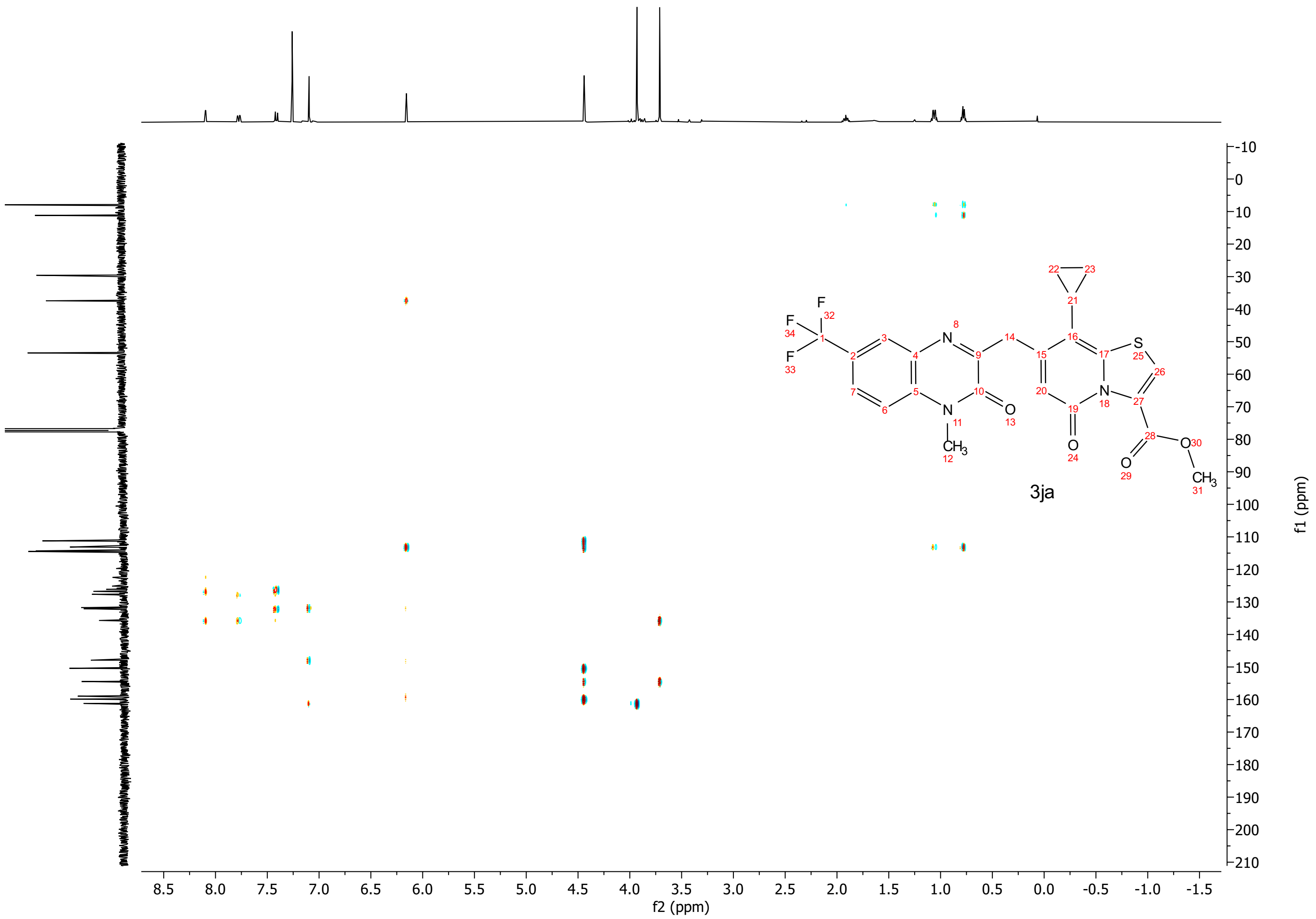

Figure S.109: gHMBC spectrum ($CDCl_3$) of methyl 8-cyclopropyl-7-((4-methyl-3-oxo-7-(trifluoromethyl)-3,4-dihydroquinoxalin-2-yl)methyl)-5-oxo-5H-thiazolo[3,2-a]pyridine-3-carboxylate, **3ja**.

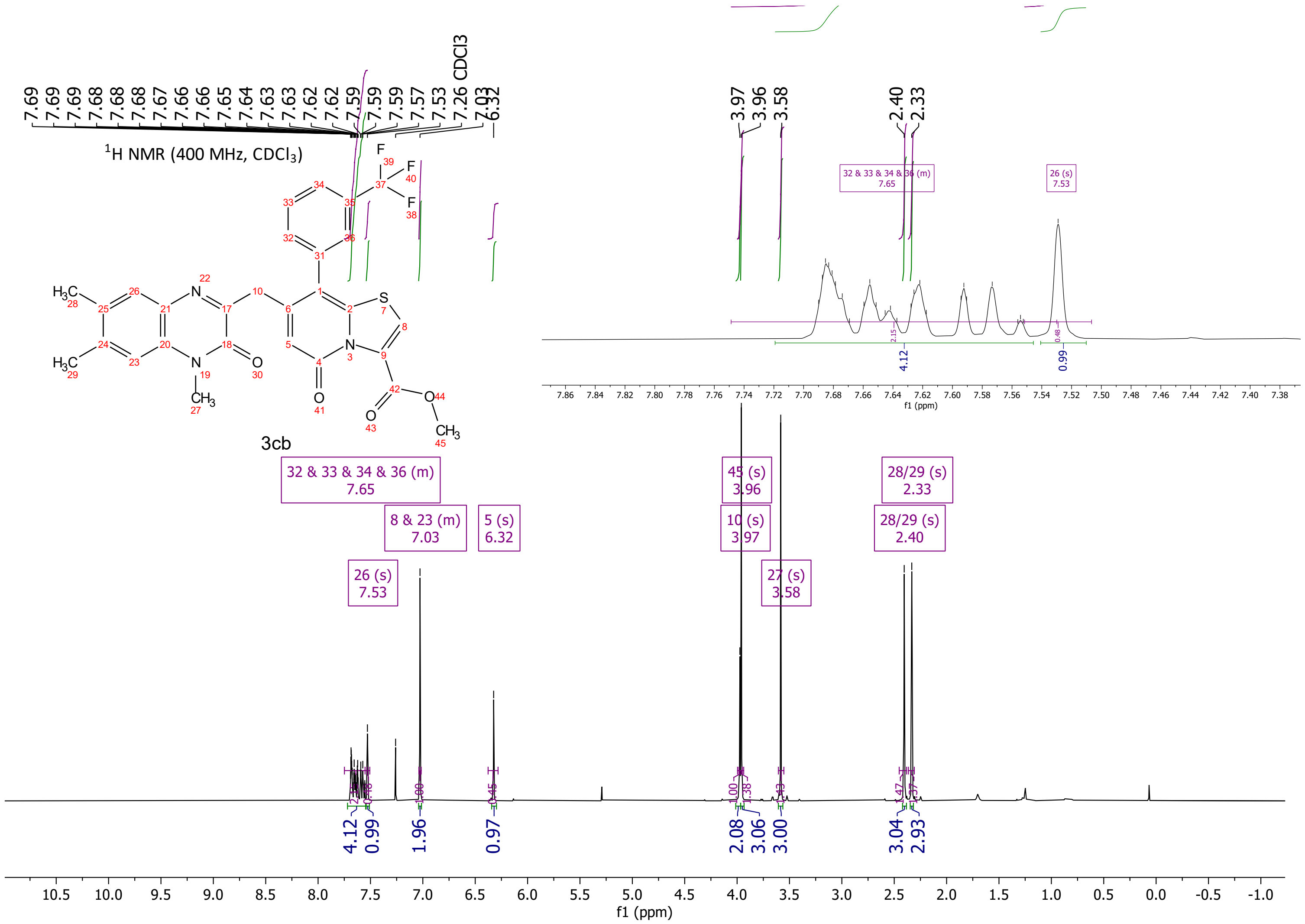

Figure S.110: ¹H NMR spectrum ($CDCl_3$, 400 MHz) of methyl 5-oxo-8-(3-(trifluoromethyl)phenyl)-7-((4,6,7-trimethyl-3-oxo-3,4-dihydroquinoxalin-2-yl)methyl)-5H-thiazolo[3,2-a]pyridine-3-carboxylate, **3cb**.

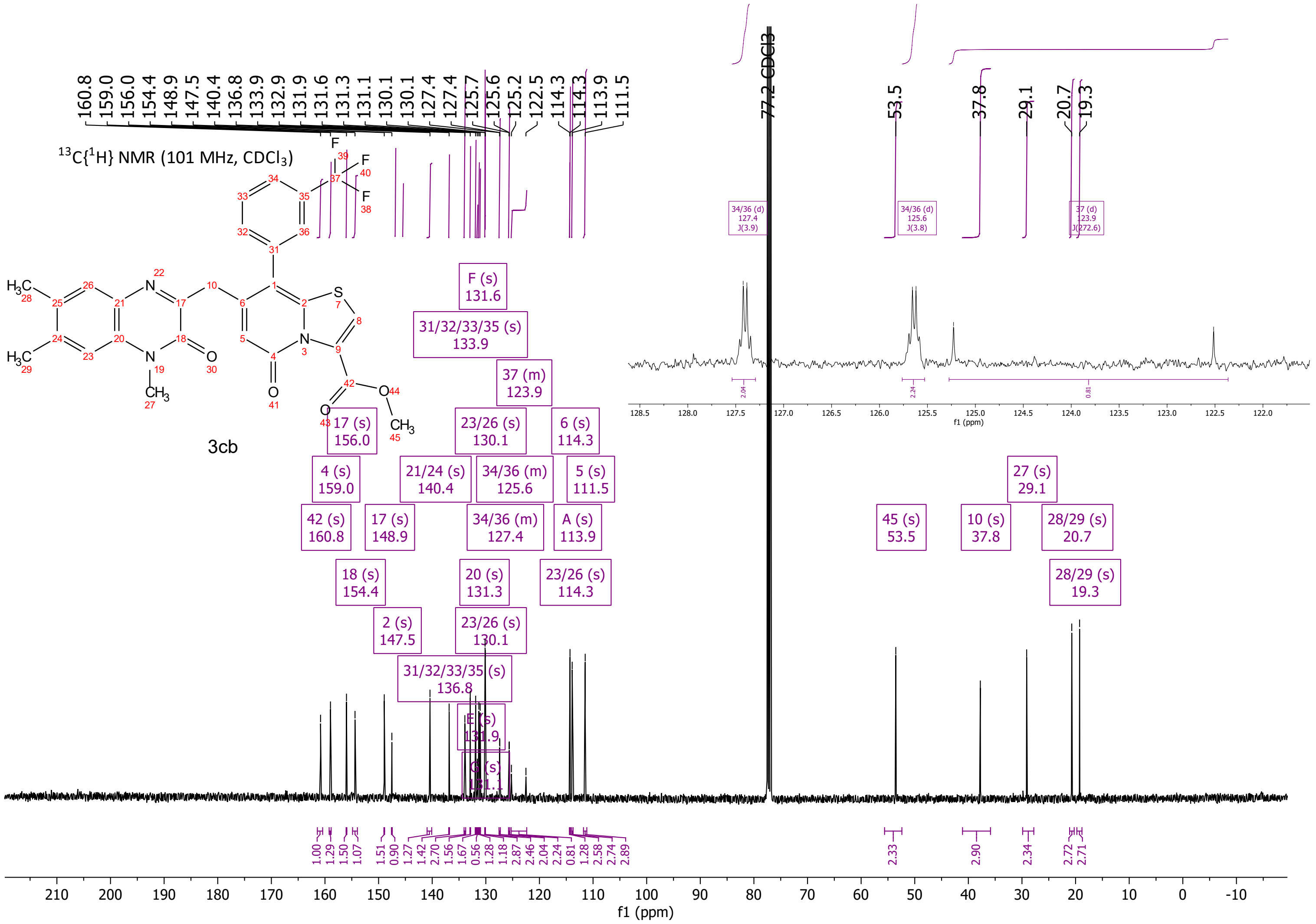

Figure S.111: $^{13}C\{^{1}H\}$ NMR spectrum ($CDCl_3$, 101 MHz) of methyl 5-oxo-8-(3-(trifluoromethyl)phenyl)-7-((4,6,7-trimethyl-3-oxo-3,4-dihydroquinoxalin-2-yl)methyl)-5H-thiazolo[3,2-a]pyridine-3-carboxylate, **3cb**.

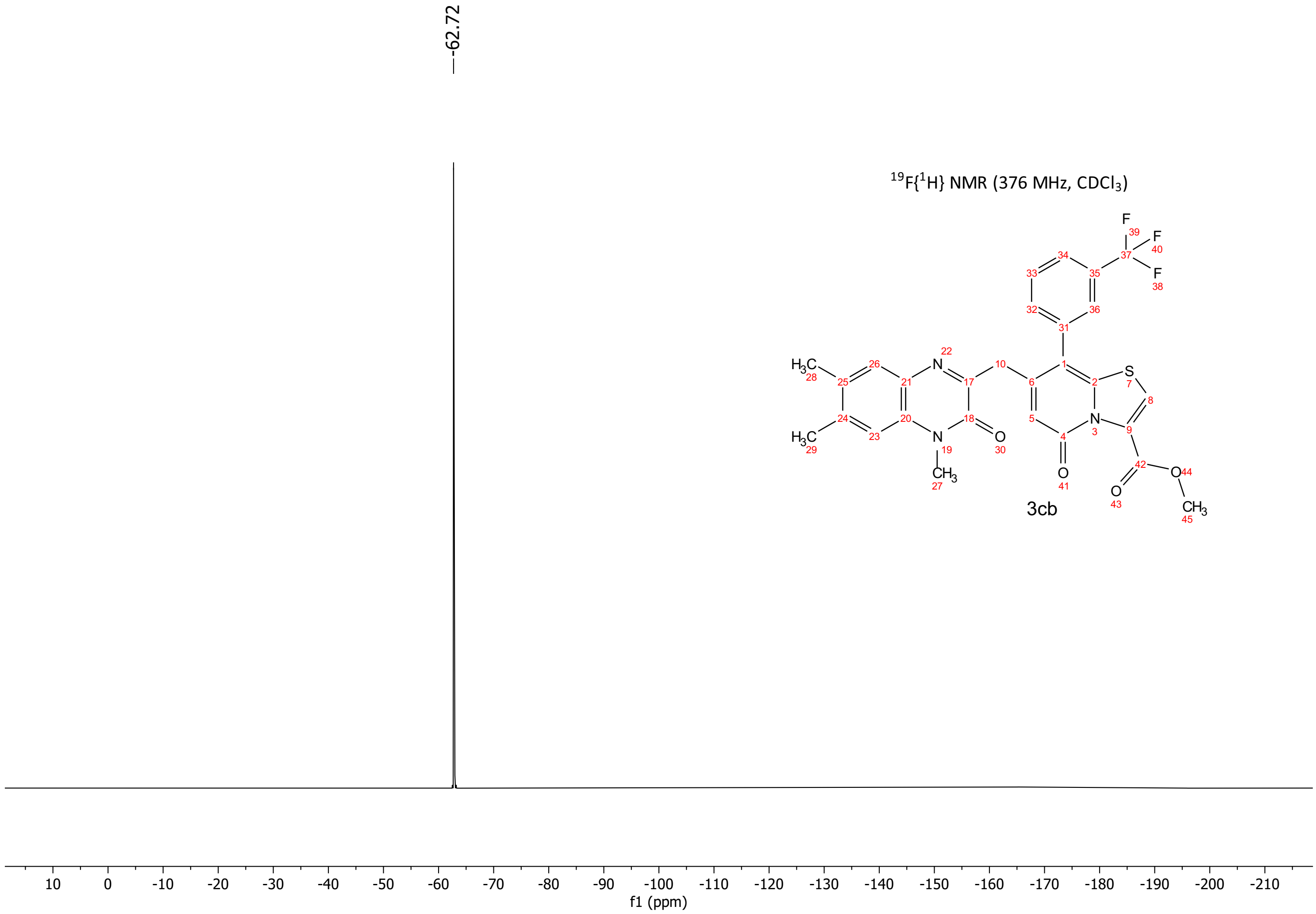

Figure S.112: $^{19}F\{^{1}H\}$ NMR spectrum ($CDCl_3$, 376 MHz) of methyl 5-oxo-8-(3-(trifluoromethyl)phenyl)-7-((4,6,7-trimethyl-3-oxo-3,4-dihydroquinoxalin-2-yl)methyl)-5H-thiazolo[3,2-a]pyridine-3-carboxylate, **3cb**.

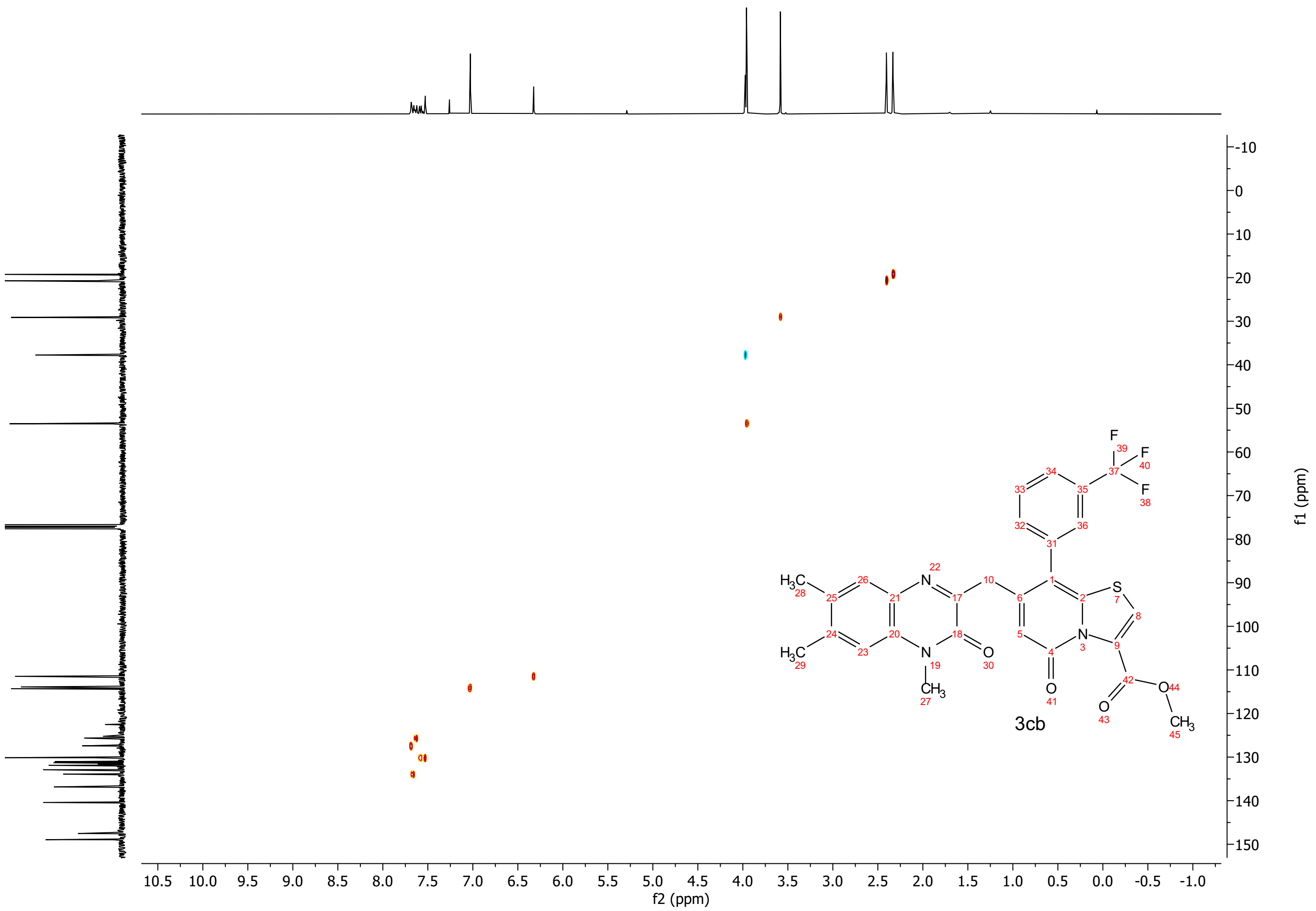

Figure S.113: gHSQC spectrum ($CDCl_3$) of methyl 5-oxo-8-(3-(trifluoromethyl)phenyl)-7-((4,6,7-trimethyl-3-oxo-3,4-dihydroquinoxalin-2-yl)methyl)-5H-thiazolo[3,2-a]pyridine-3-carboxylate, **3cb**.

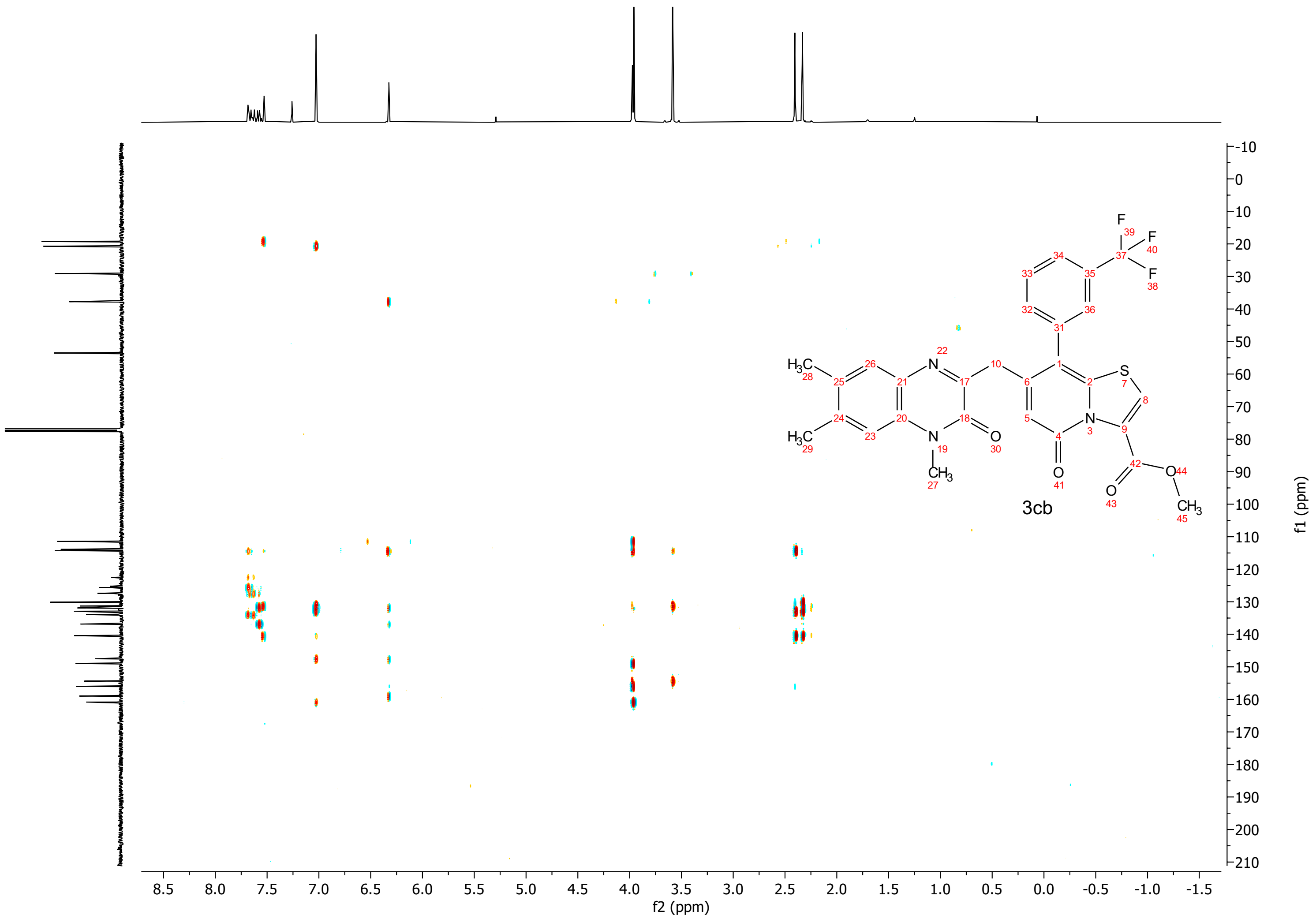

Figure S.114: gHMBC spectrum ($CDCl_3$) of methyl 5-oxo-8-(3-(trifluoromethyl)phenyl)-7-((4,6,7-trimethyl-3-oxo-3,4-dihydroquinoxalin-2-yl)methyl)-5H-thiazolo[3,2-a]pyridine-3-carboxylate, **3cb**.

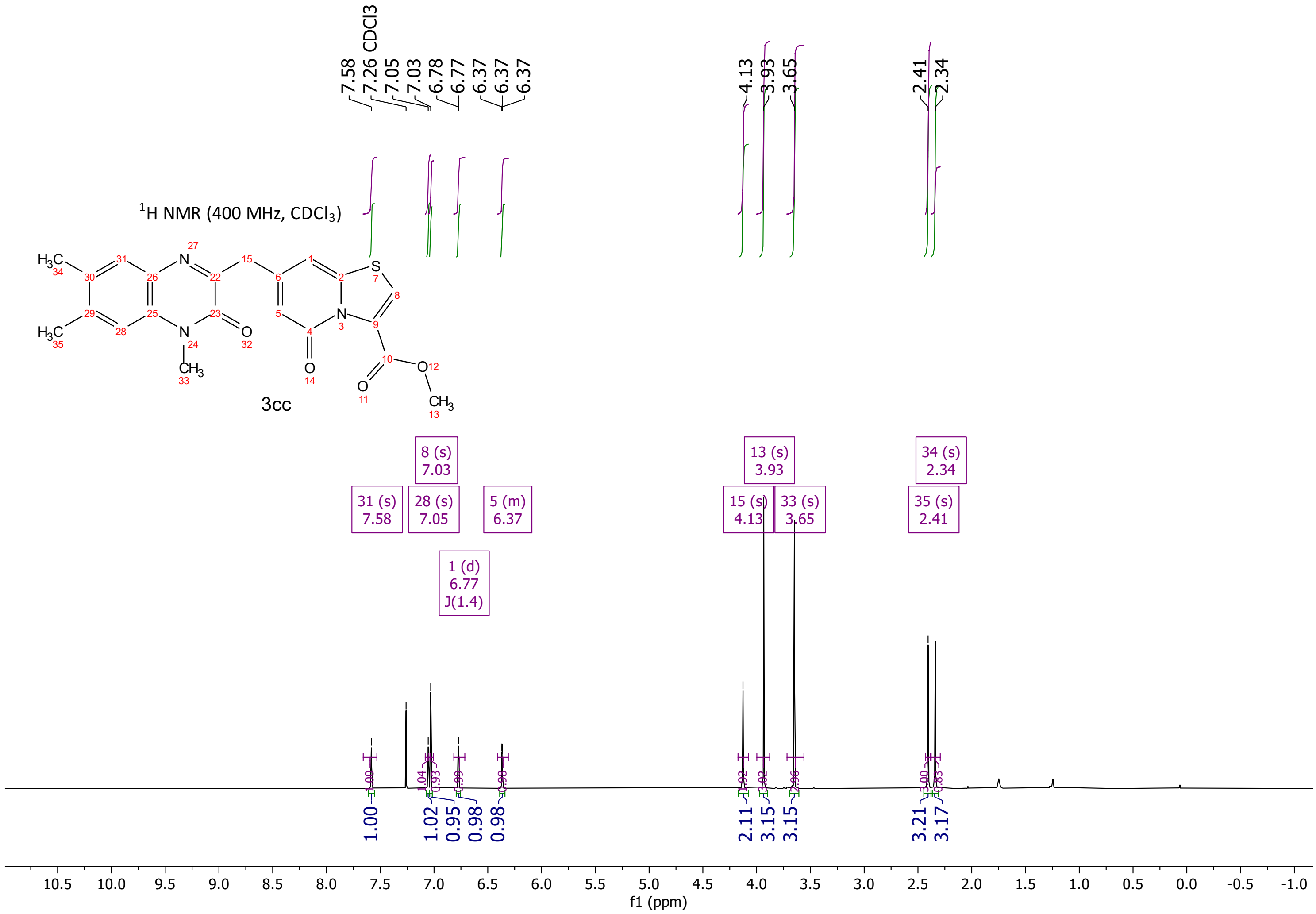

Figure S.115: ^{1}H NMR spectrum ($CDCl_3$, 400 MHz) of methyl 5-oxo-7-((4,6,7-trimethyl-3-oxo-3,4-dihydroquinoxalin-2-yl)methyl)-5H-thiazolo[3,2-a]pyridine-3-carboxylate, **3cc**.

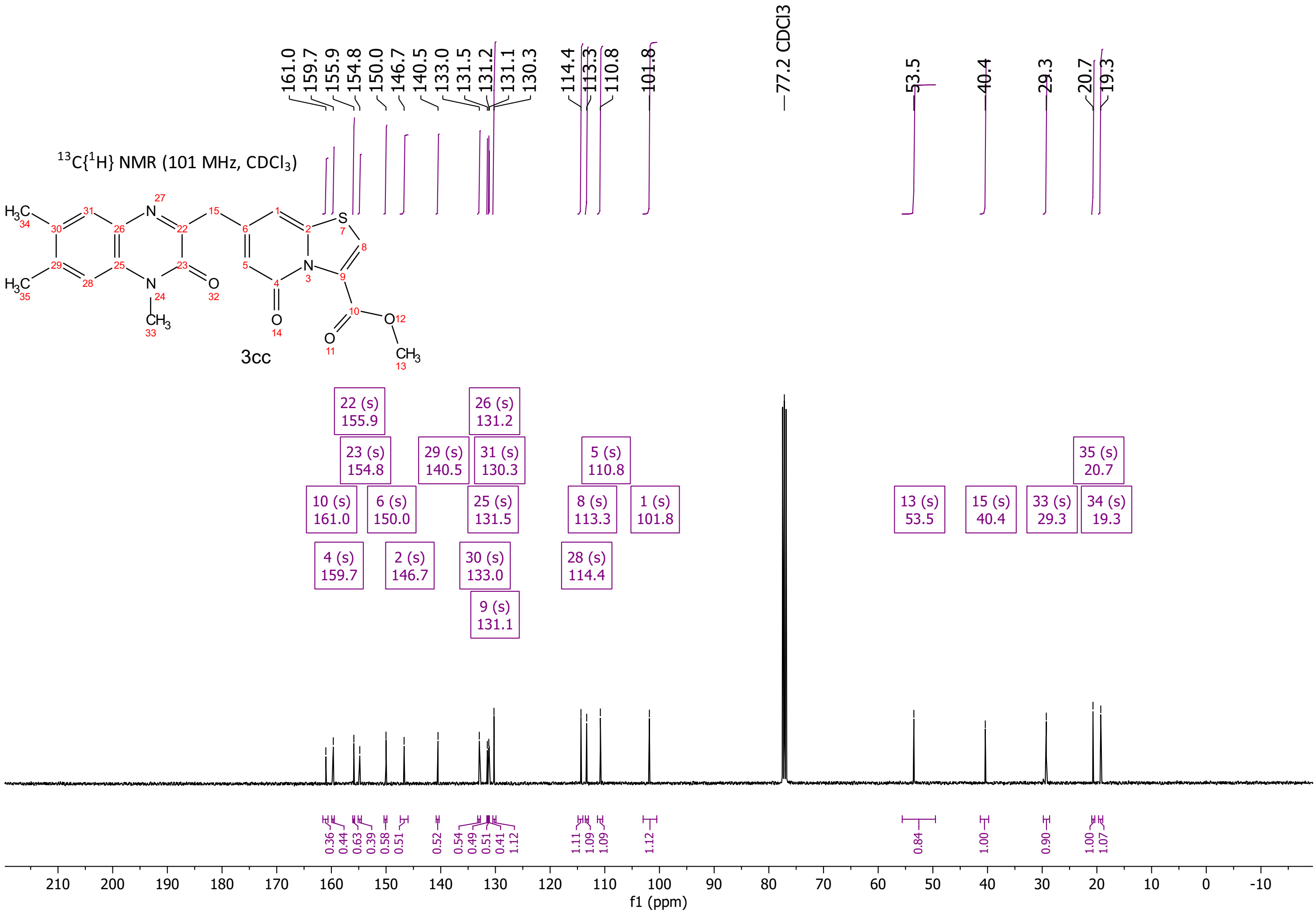

Figure S.116: $^{13}C\{^1H\}$ NMR spectrum ($CDCl_3$, 101 MHz) of methyl 5-oxo-7-((4,6,7-trimethyl-3-oxo-3,4-dihydroquinoxalin-2-yl)methyl)-5H-thiazolo[3,2-a]pyridine-3-carboxylate, **3cc**.

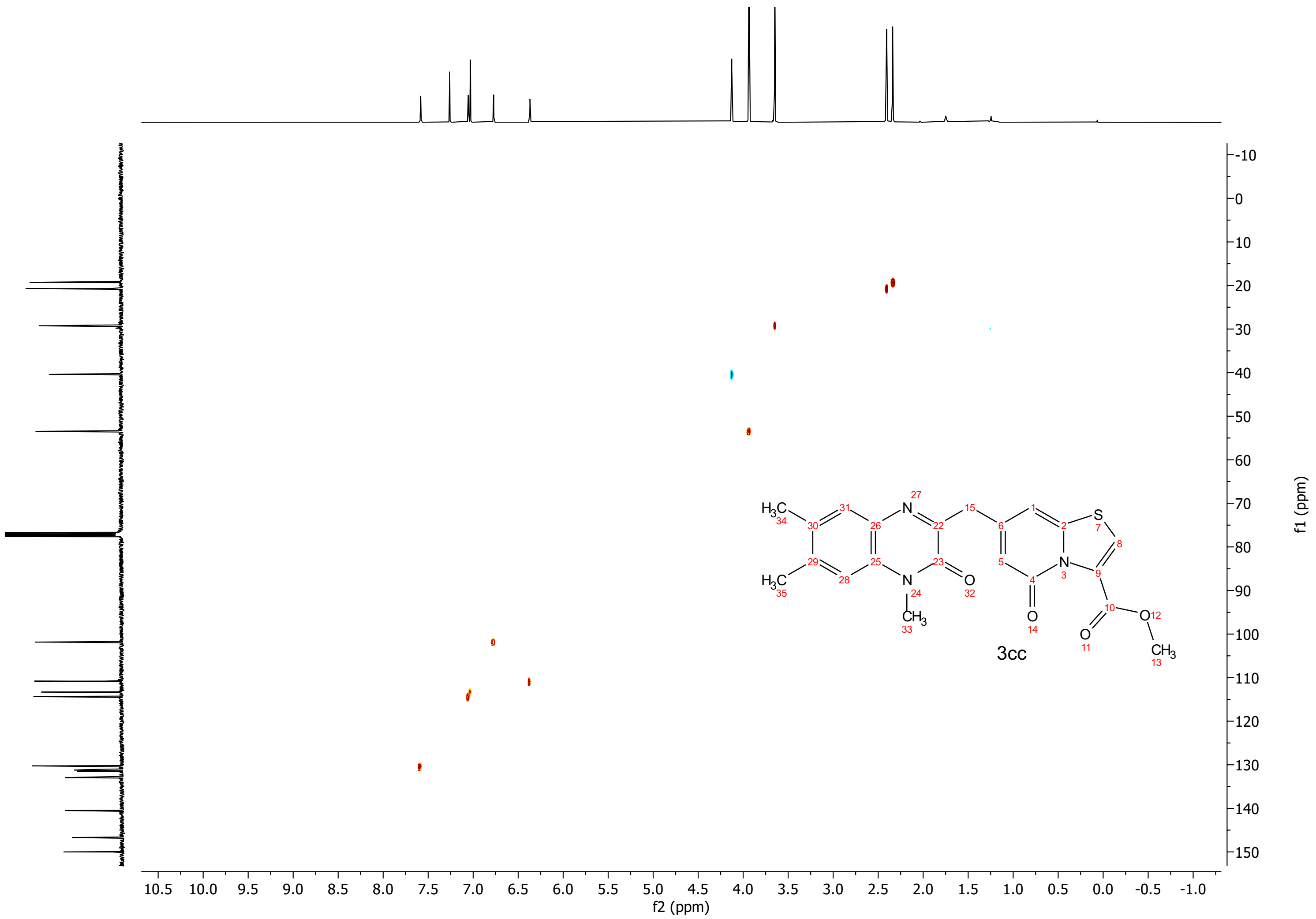

Figure S.117: gHSQC spectrum ($CDCl_3$) of methyl 5-oxo-7-((4,6,7-trimethyl-3-oxo-3,4-dihydroquinoxalin-2-yl)methyl)-5H-thiazolo[3,2-a]pyridine-3-carboxylate, **3cc**.

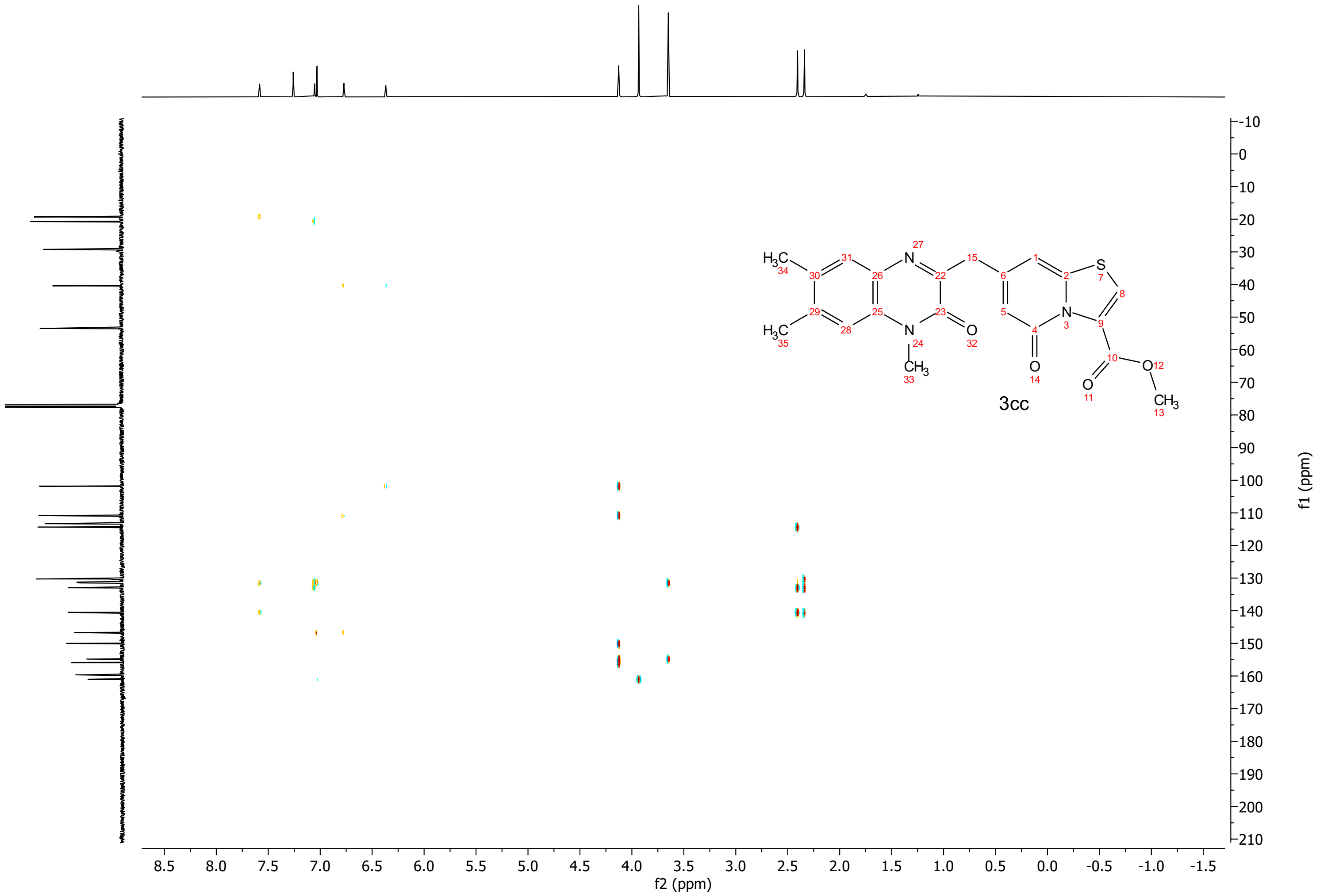

Figure S.118: gHMBC spectrum ($CDCl_3$) of methyl 5-oxo-7-((4,6,7-trimethyl-3-oxo-3,4-dihydroquinoxalin-2-yl)methyl)-5H-thiazolo[3,2-a]pyridine-3-carboxylate, **3cc**.

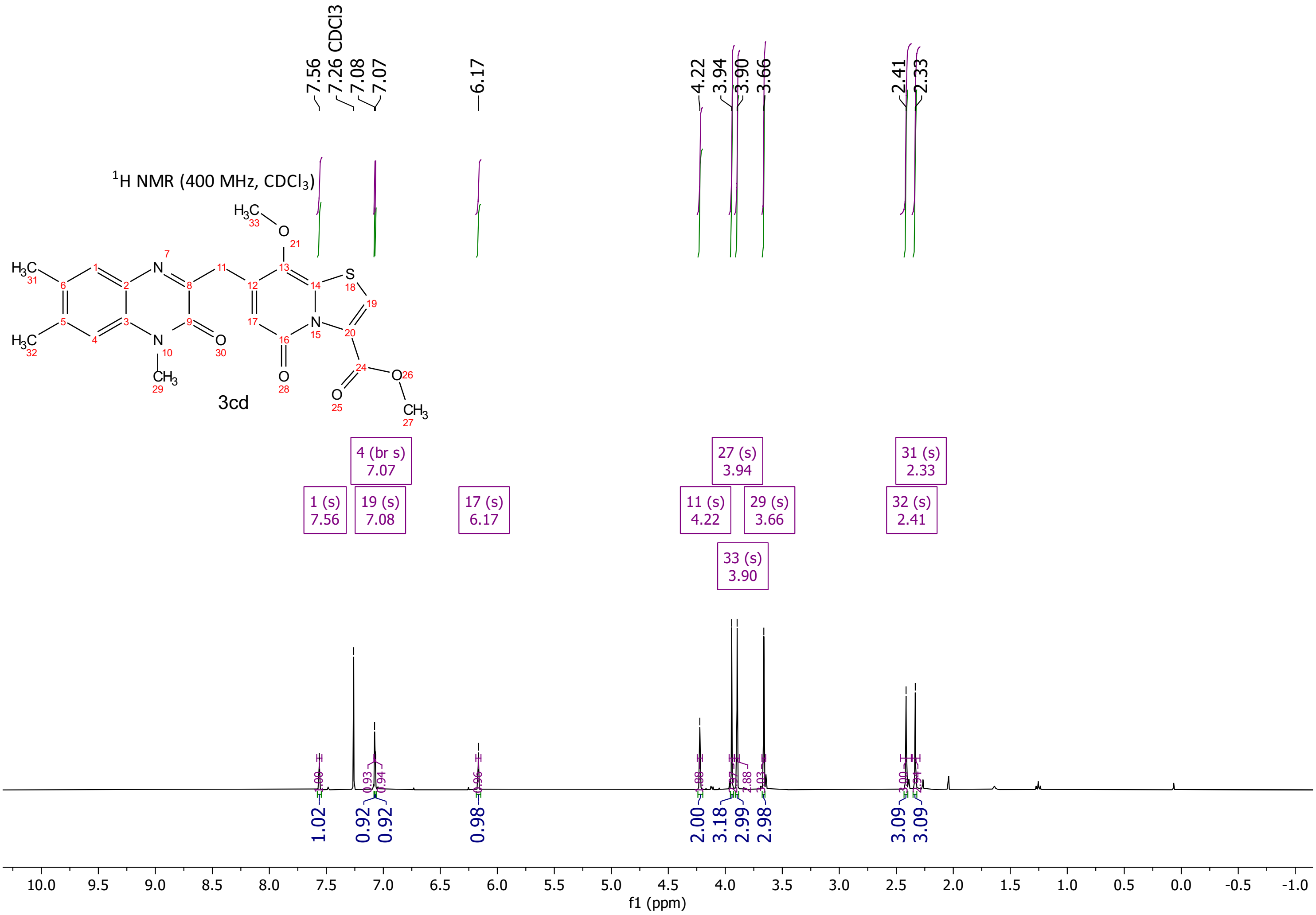

Figure S.119: ^{1}H NMR spectrum ($CDCl_3$, 400 MHz) of methyl 8-methoxy-5-oxo-7-((4,6,7-trimethyl-3-oxo-3,4-dihydroquinoxalin-2-yl)methyl)-5H-thiazolo[3,2-a]pyridine-3-carboxylate, **3cd**.

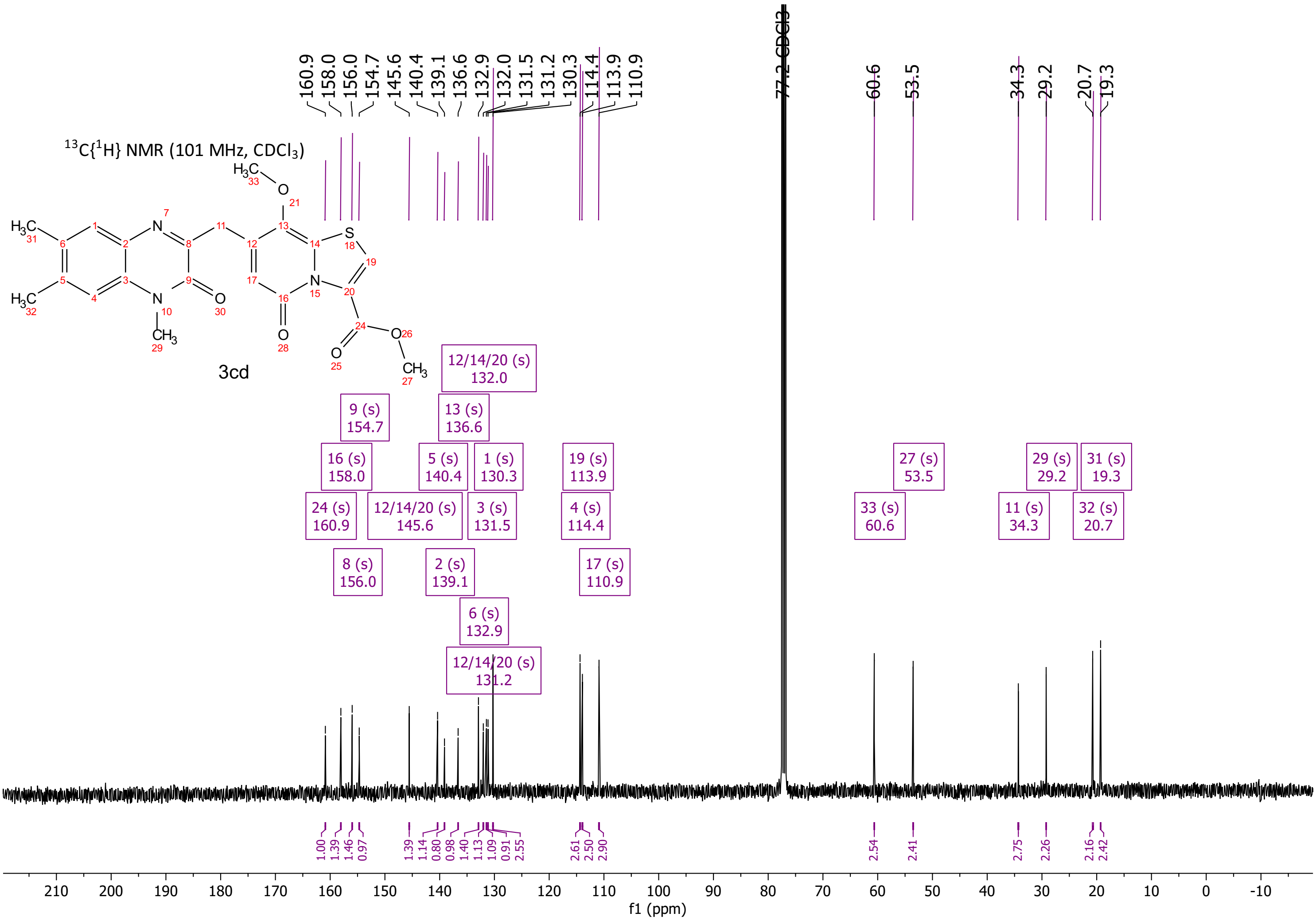

Figure S.120: ^{13}C{^{1}H} NMR spectrum ($CDCl_3$, 101 MHz) of methyl 8-methoxy-5-oxo-7-((4,6,7-trimethyl-3-oxo-3,4-dihydroquinoxalin-2-yl)methyl)-5H-thiazolo[3,2-a]pyridine-3-carboxylate, **3cd**.

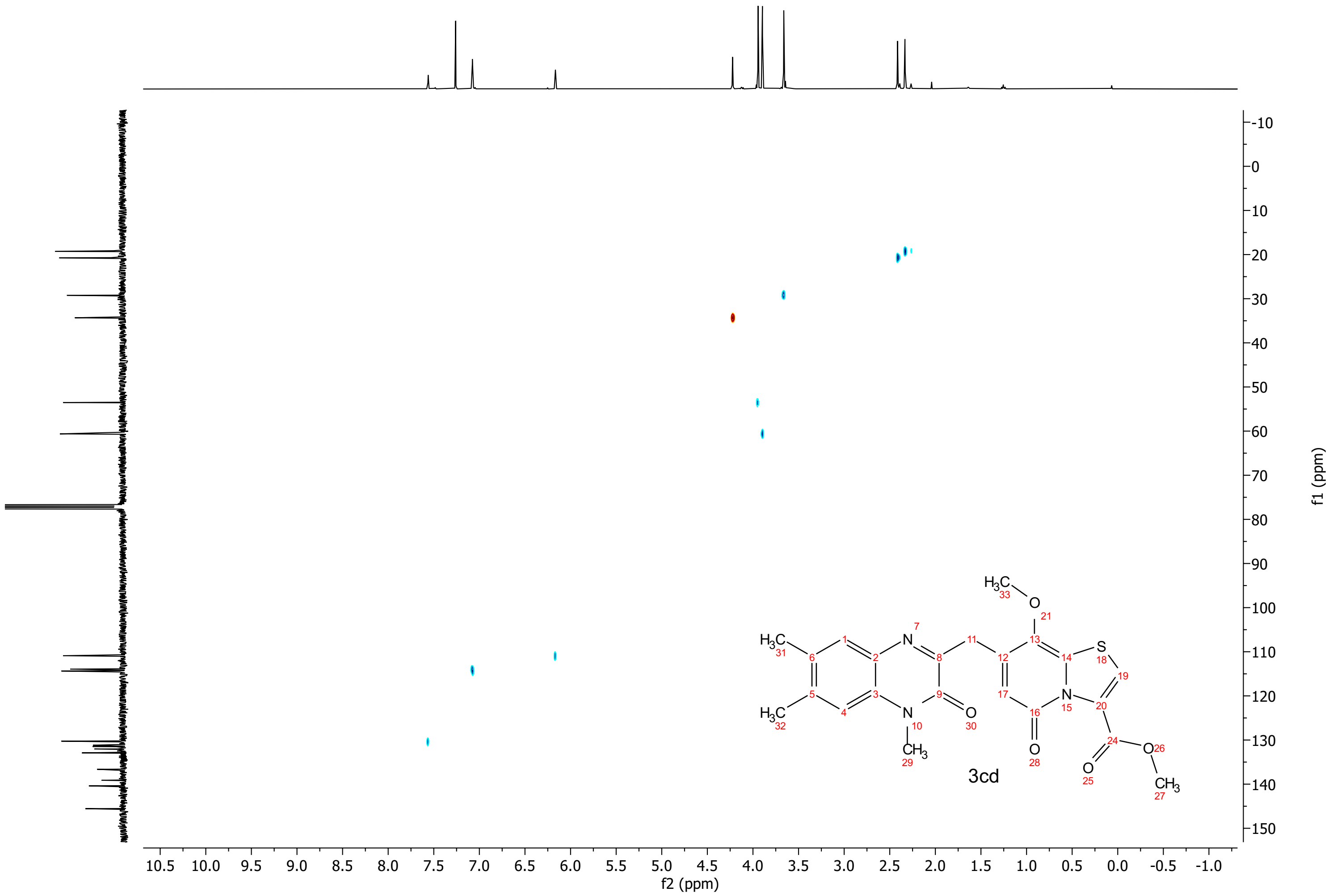

Figure S.121: gHSQC spectrum ($CDCl_3$) of methyl 8-methoxy-5-oxo-7-((4,6,7-trimethyl-3-oxo-3,4-dihydroquinoxalin-2-yl)methyl)-5H-thiazolo[3,2-a]pyridine-3-carboxylate, **3cd**.

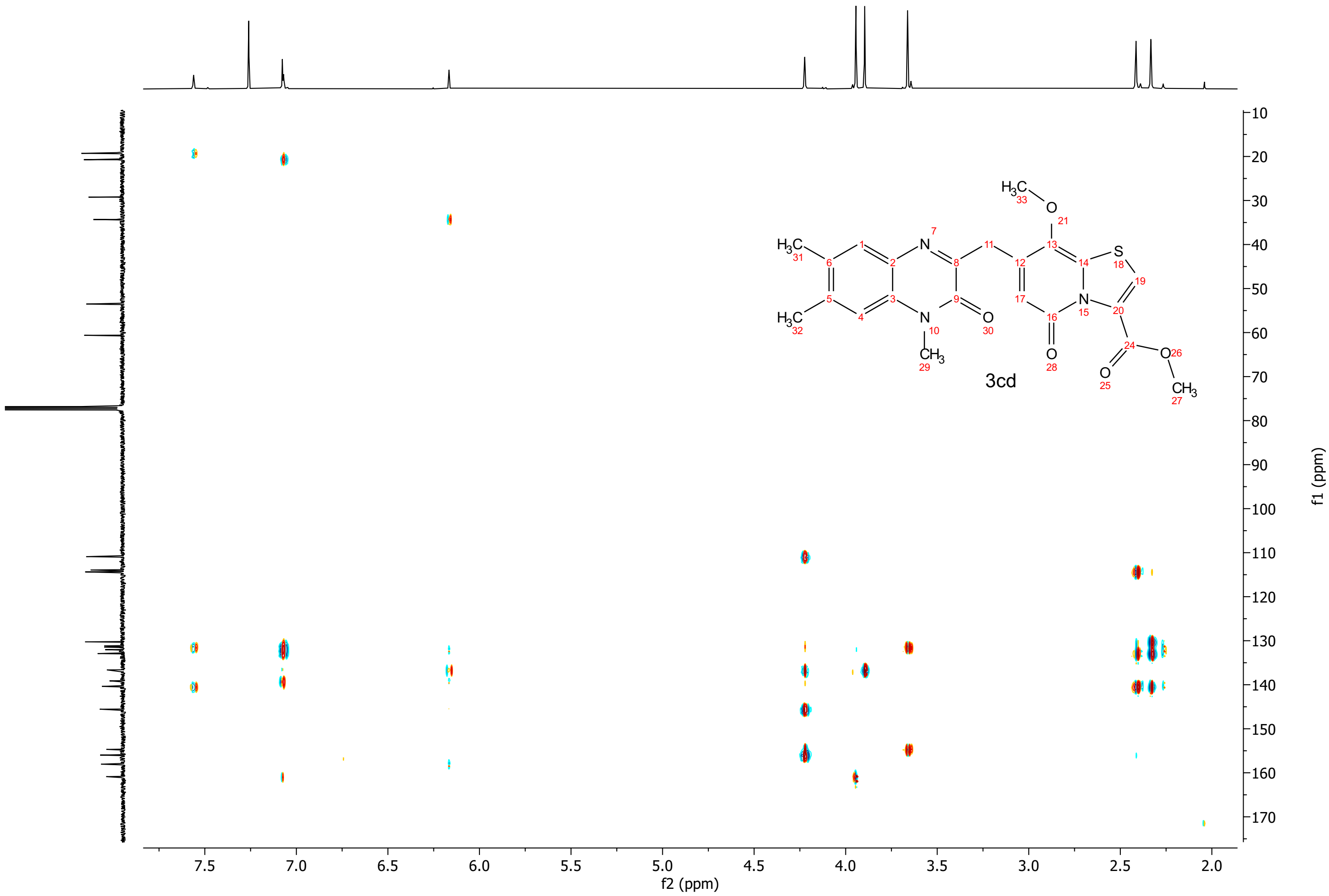

Figure S.122: gHMBC spectrum ($CDCl_3$) of methyl 8-methoxy-5-oxo-7-((4,6,7-trimethyl-3-oxo-3,4-dihydroquinoxalin-2-yl)methyl)-5H-thiazolo[3,2-a]pyridine-3-carboxylate, **3cd**.

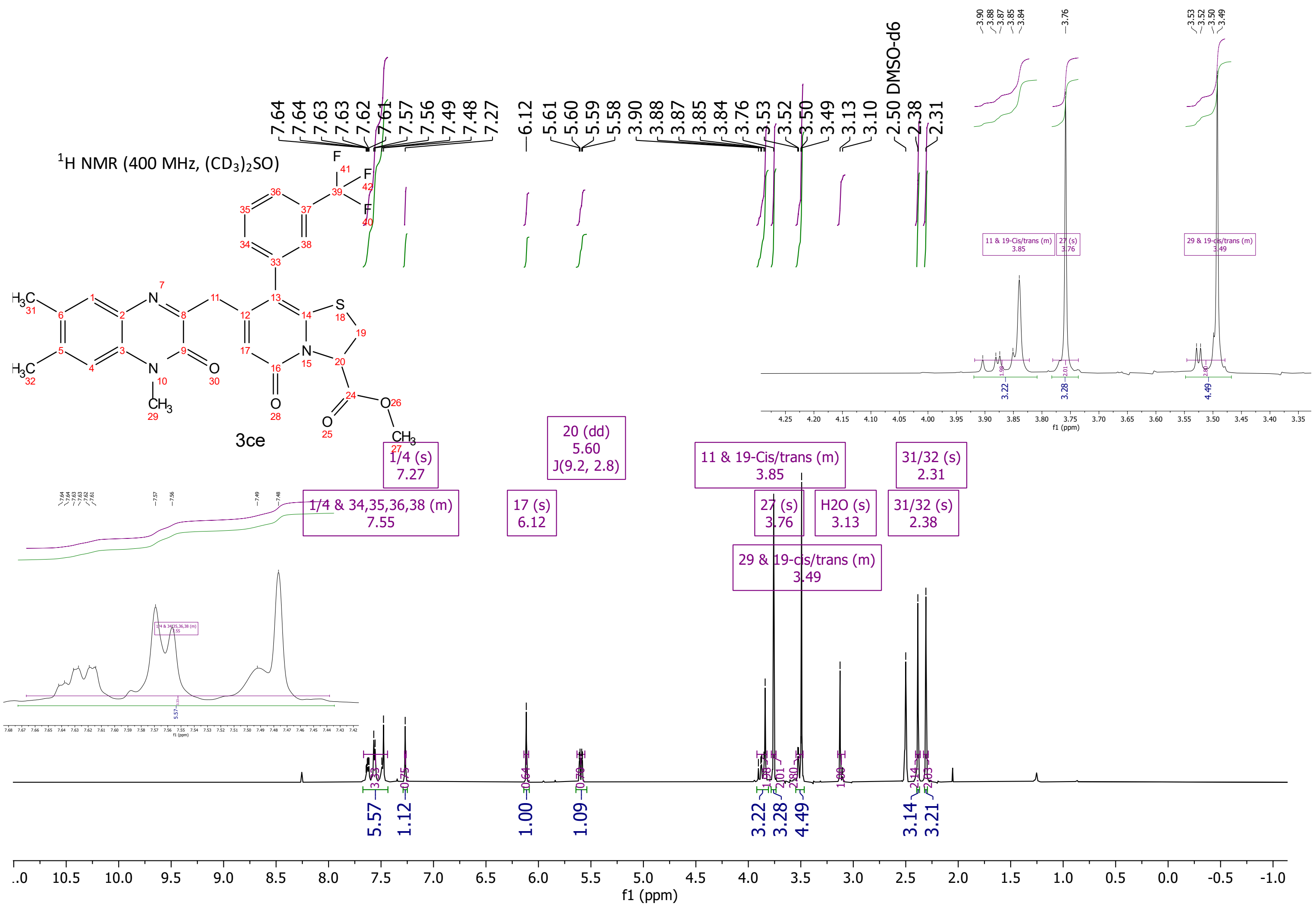

Figure S.123: ^{1}H NMR spectrum ($(CD_3)_2SO$, 343 K, 400 MHz) of methyl 5-oxo-8-(3-(trifluoromethyl)phenyl)-7-((4,6,7-trimethyl-3-oxo-3,4-dihydroquinoxalin-2-yl)methyl)-2,3-dihydro-5H-thiazolo[3,2-a]pyridine-3-carboxylate, **3ce**.

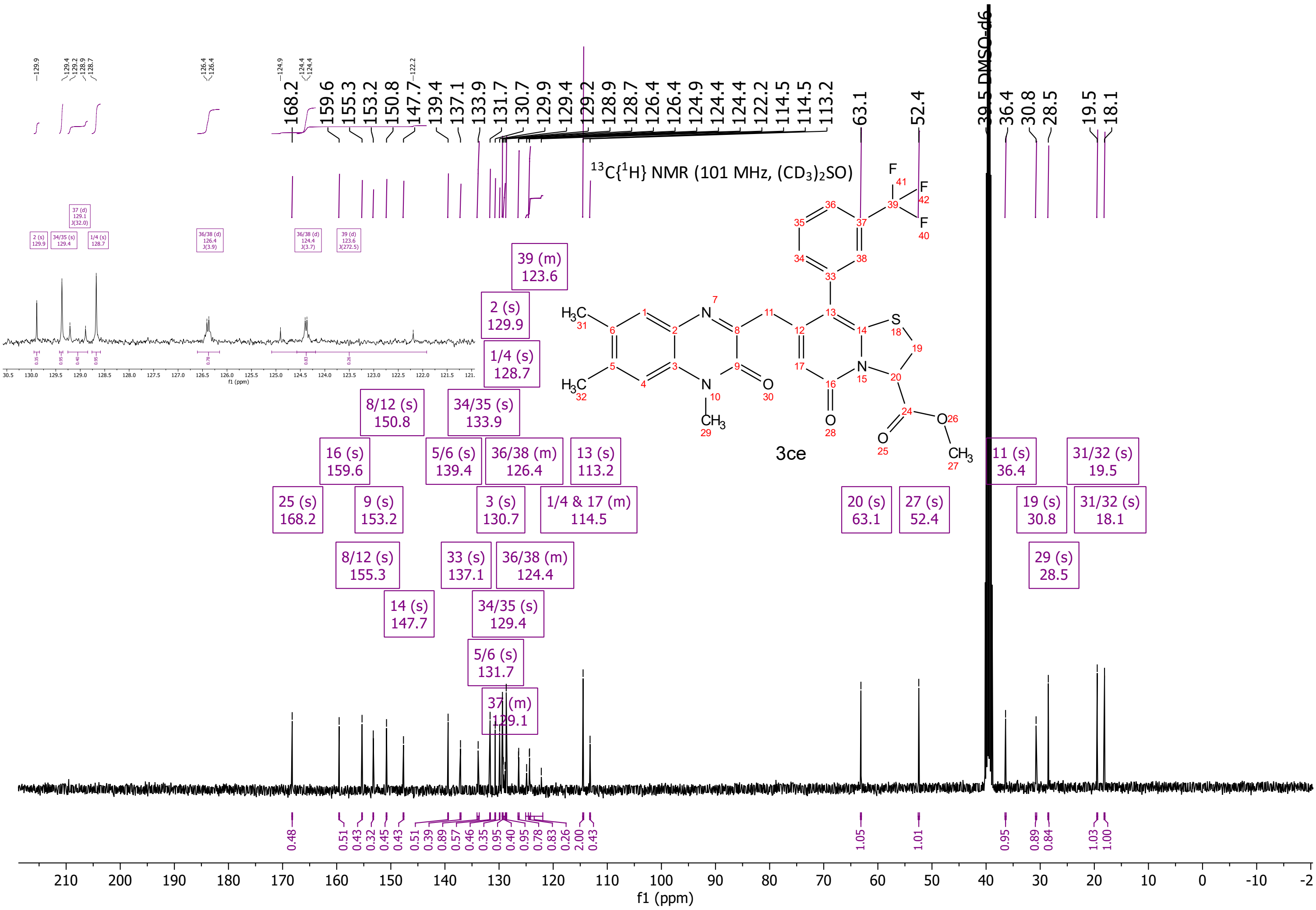

Figure S.124: $^{13}C\{^{1}H\}$ NMR spectrum ($(CD_3)_2SO$, 343 K, 101 MHz) of methyl 5-oxo-8-(3-(trifluoromethyl)phenyl)-7-((4,6,7-trimethyl-3-oxo-3,4-dihydroquinoxalin-2-yl)methyl)-2,3-dihydro-5H-thiazolo[3,2-a]pyridine-3-carboxylate, **3ce**.

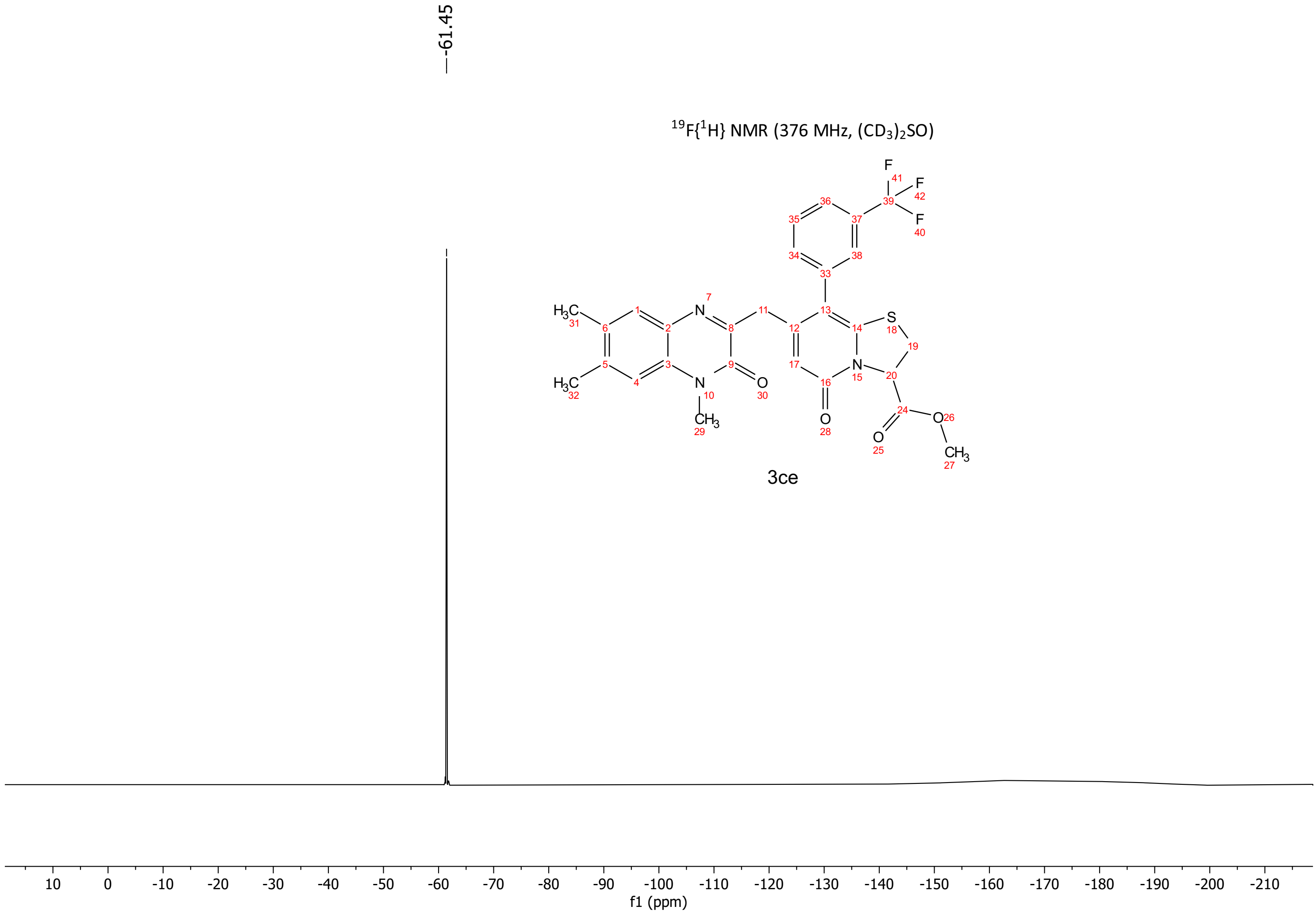

Figure S.125: $^{19}F\{^1H\}$ NMR spectrum ($(CD_3)_2SO$, 343 K, 376 MHz) of methyl 5-oxo-8-(3-(trifluoromethyl)phenyl)-7-((4,6,7-trimethyl-3-oxo-3,4-dihydroquinoxalin-2-yl)methyl)-2,3-dihydro-5H-thiazolo[3,2-a]pyridine-3-carboxylate, **3ce**.

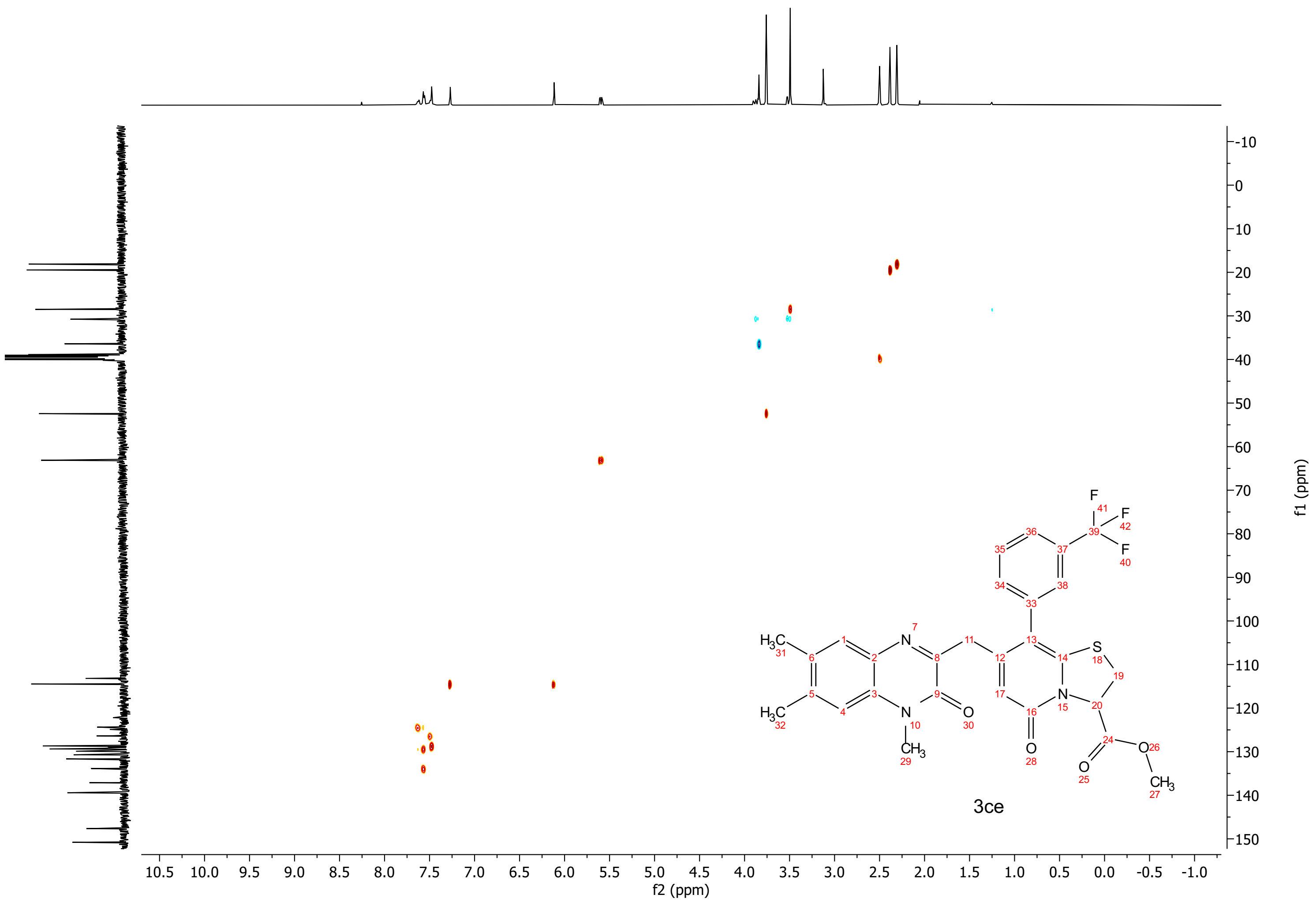

Figure S.126: gHSQC spectrum ($(CD_3)_2SO$, 343 K) of methyl 5-oxo-8-(3-(trifluoromethyl)phenyl)-7-((4,6,7-trimethyl-3-oxo-3,4-dihydroquinoxalin-2-yl)methyl)-2,3-dihydro-5H-thiazolo[3,2-a]pyridine-3-carboxylate, **3ce**.

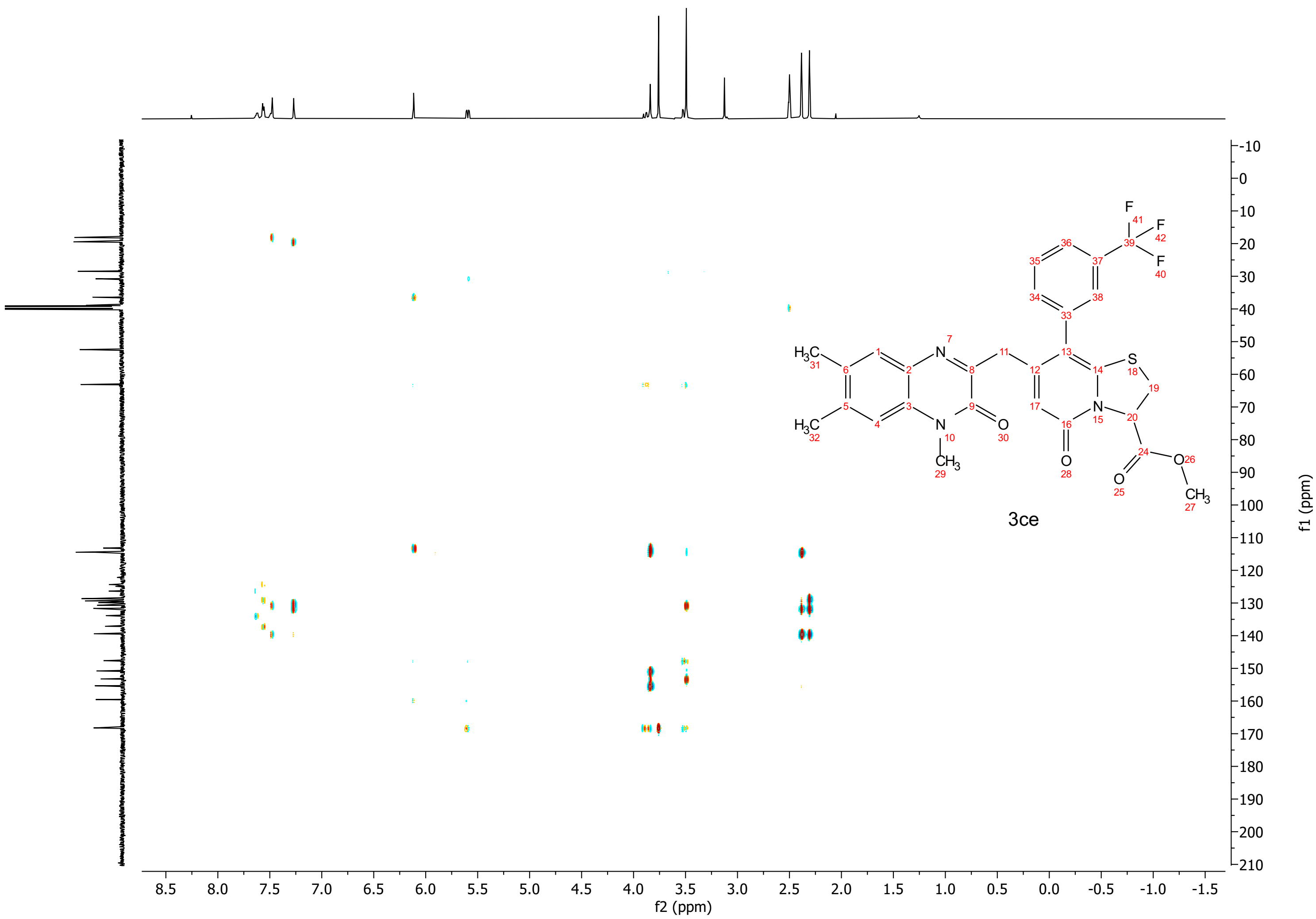

Figure S.127: gHMBC spectrum ($(CD_3)_2SO$, 343 K) of methyl 5-oxo-8-(3-(trifluoromethyl)phenyl)-7-((4,6,7-trimethyl-3-oxo-3,4-dihydroquinoxalin-2-yl)methyl)-2,3-dihydro-5H-thiazolo[3,2-a]pyridine-3-carboxylate, **3ce**.

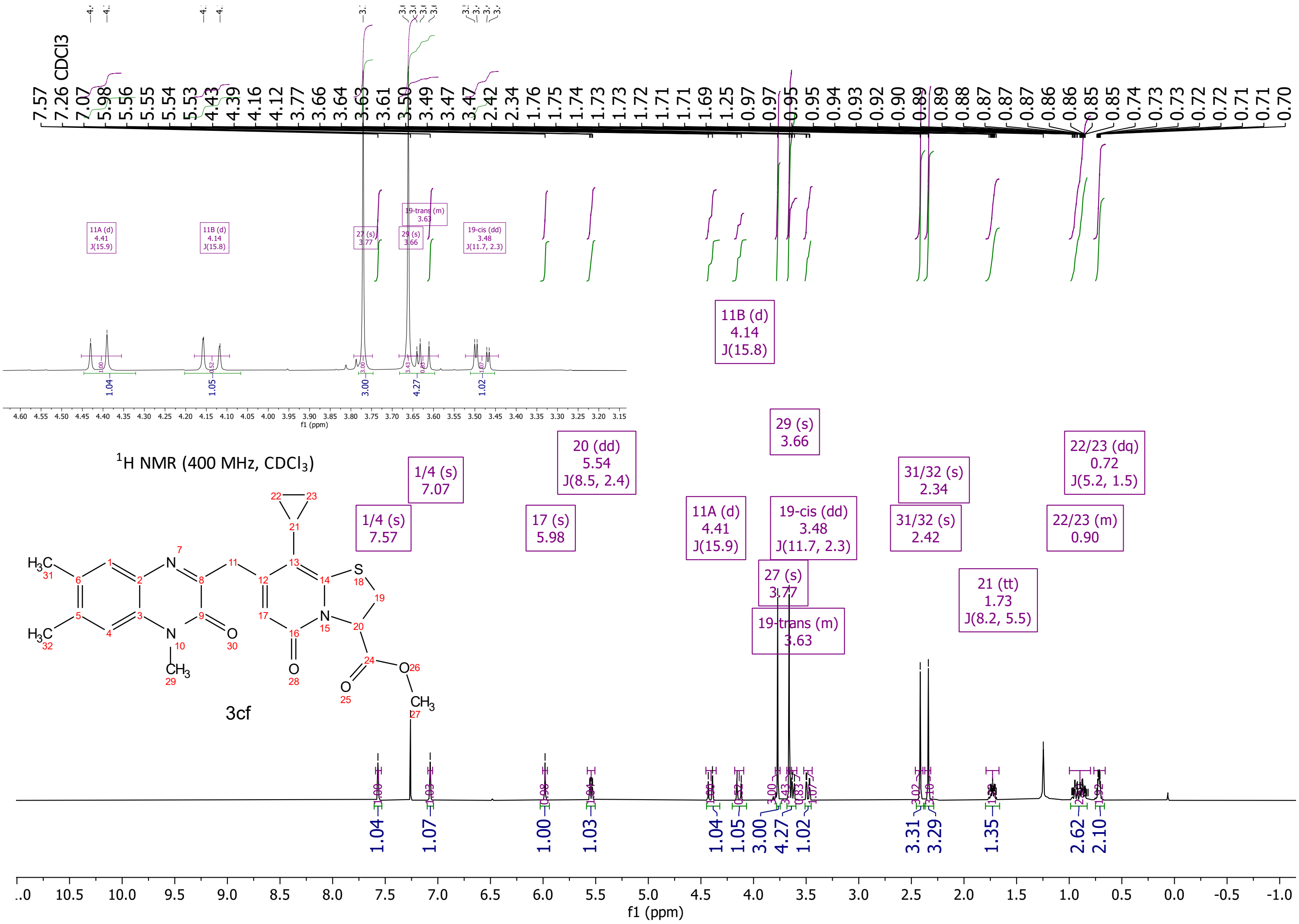

Figure S.128: ^{1}H NMR spectrum ($CDCl_3$, 400 MHz) of methyl 8-cyclopropyl-5-oxo-7-((4,6,7-trimethyl-3-oxo-3,4-dihydroquinoxalin-2-yl)methyl)-2,3-dihydro-5H-thiazolo[3,2-a]pyridine-3-carboxylate, **3cf**.

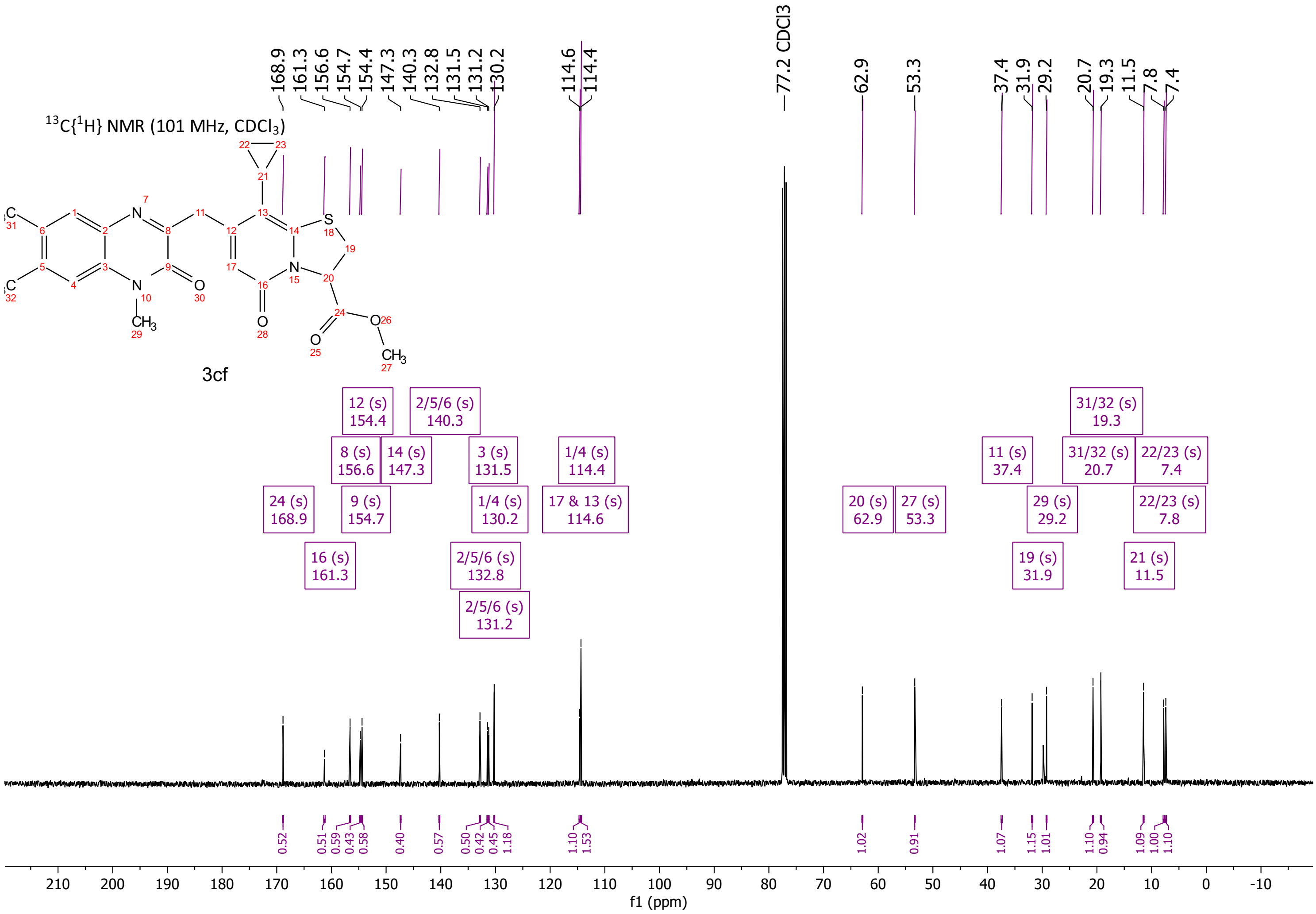

Figure S.129: $^{13}C\{^1H\}$ NMR spectrum ($CDCl_3$, 101 MHz) of methyl 8-cyclopropyl-5-oxo-7-((4,6,7-trimethyl-3-oxo-3,4-dihydroquinoxalin-2-yl)methyl)-2,3-dihydro-5H-thiazolo[3,2-a]pyridine-3-carboxylate, **3cf**.

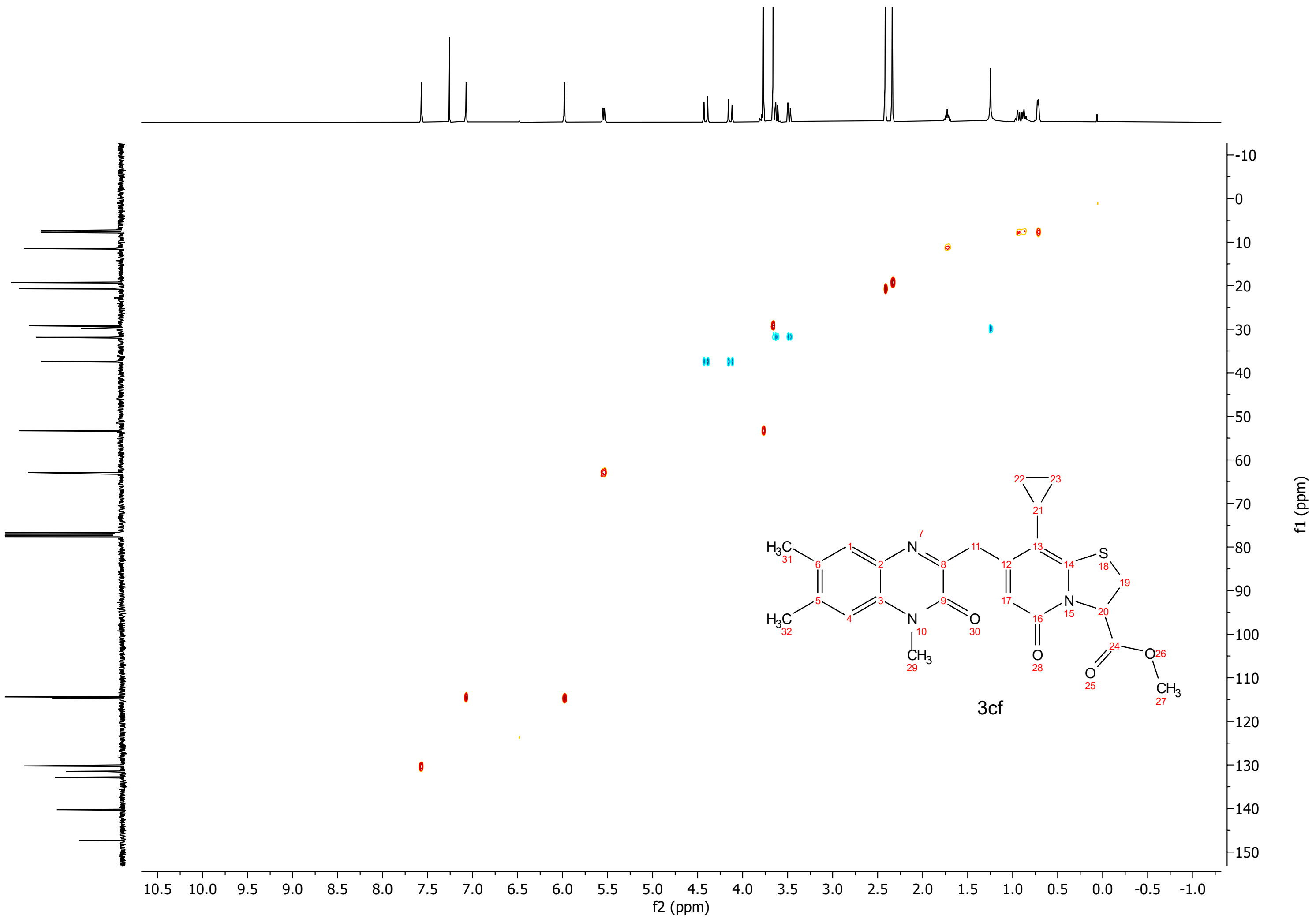

Figure S.130: gHSQC spectrum ($CDCl_3$) of methyl 8-cyclopropyl-5-oxo-7-((4,6,7-trimethyl-3-oxo-3,4-dihydroquinoxalin-2-yl)methyl)-2,3-dihydro-5H-thiazolo[3,2-a]pyridine-3-carboxylate, **3cf**.

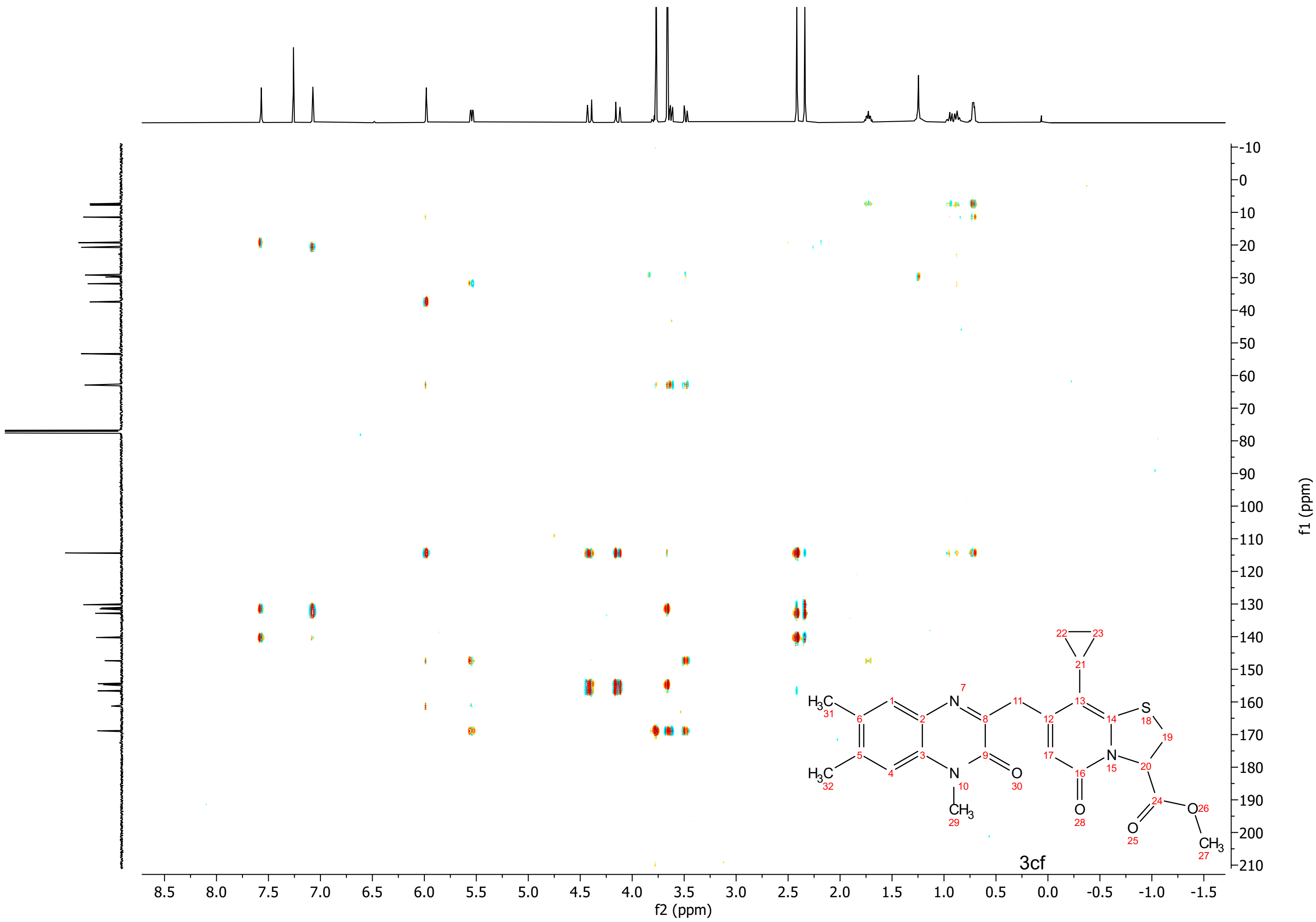

Figure S.131: gHMBC spectrum ($CDCl_3$) of methyl 8-cyclopropyl-5-oxo-7-((4,6,7-trimethyl-3-oxo-3,4-dihydroquinoxalin-2-yl)methyl)-2,3-dihydro-5H-thiazolo[3,2-a]pyridine-3-carboxylate, **3cf**.

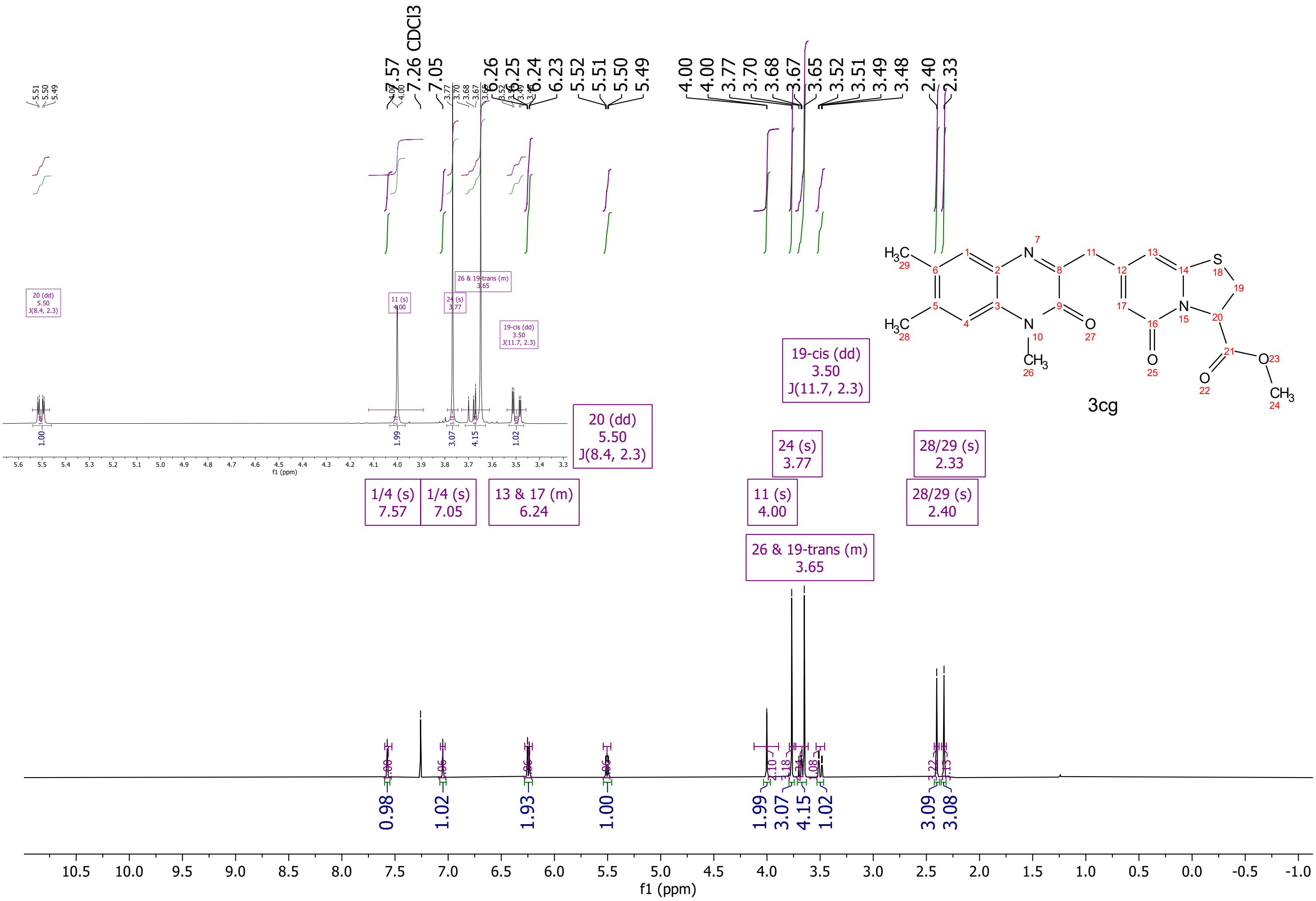

Figure S.132: ^{1}H NMR spectrum ($CDCl_3$, 400 MHz) of methyl 5-oxo-7-((4,6,7-trimethyl-3-oxo-3,4-dihydroquinoxalin-2-yl)methyl)-2,3-dihydro-5H-thiazolo[3,2-a]pyridine-3-carboxylate, **3cg**.

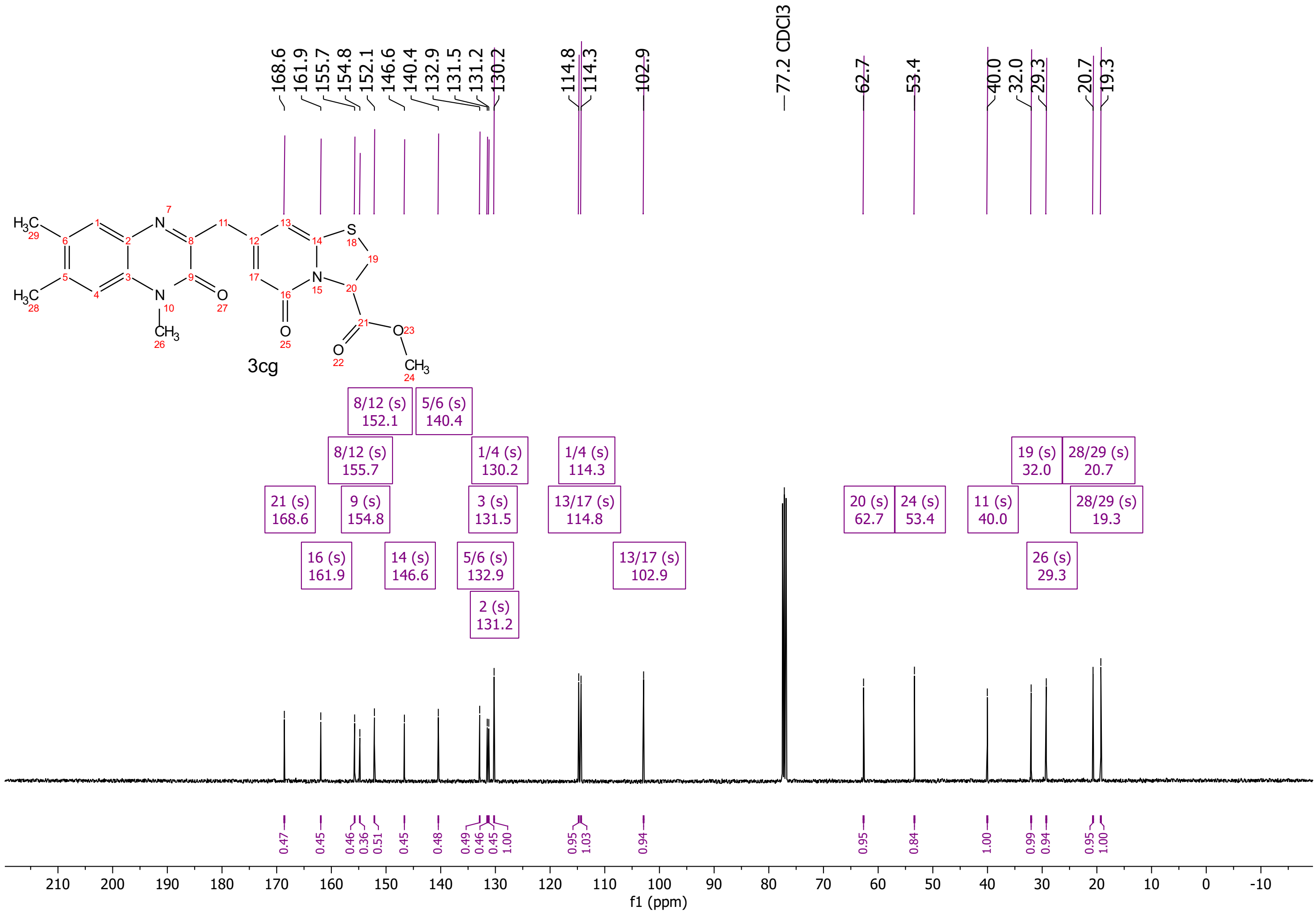

Figure S.133: $^{13}C\{^{1}H\}$ NMR spectrum ($CDCl_3$, 101 MHz) of methyl 5-oxo-7-((4,6,7-trimethyl-3-oxo-3,4-dihydroquinoxalin-2-yl)methyl)-2,3-dihydro-5H-thiazolo[3,2-a]pyridine-3-carboxylate, **3cg**.

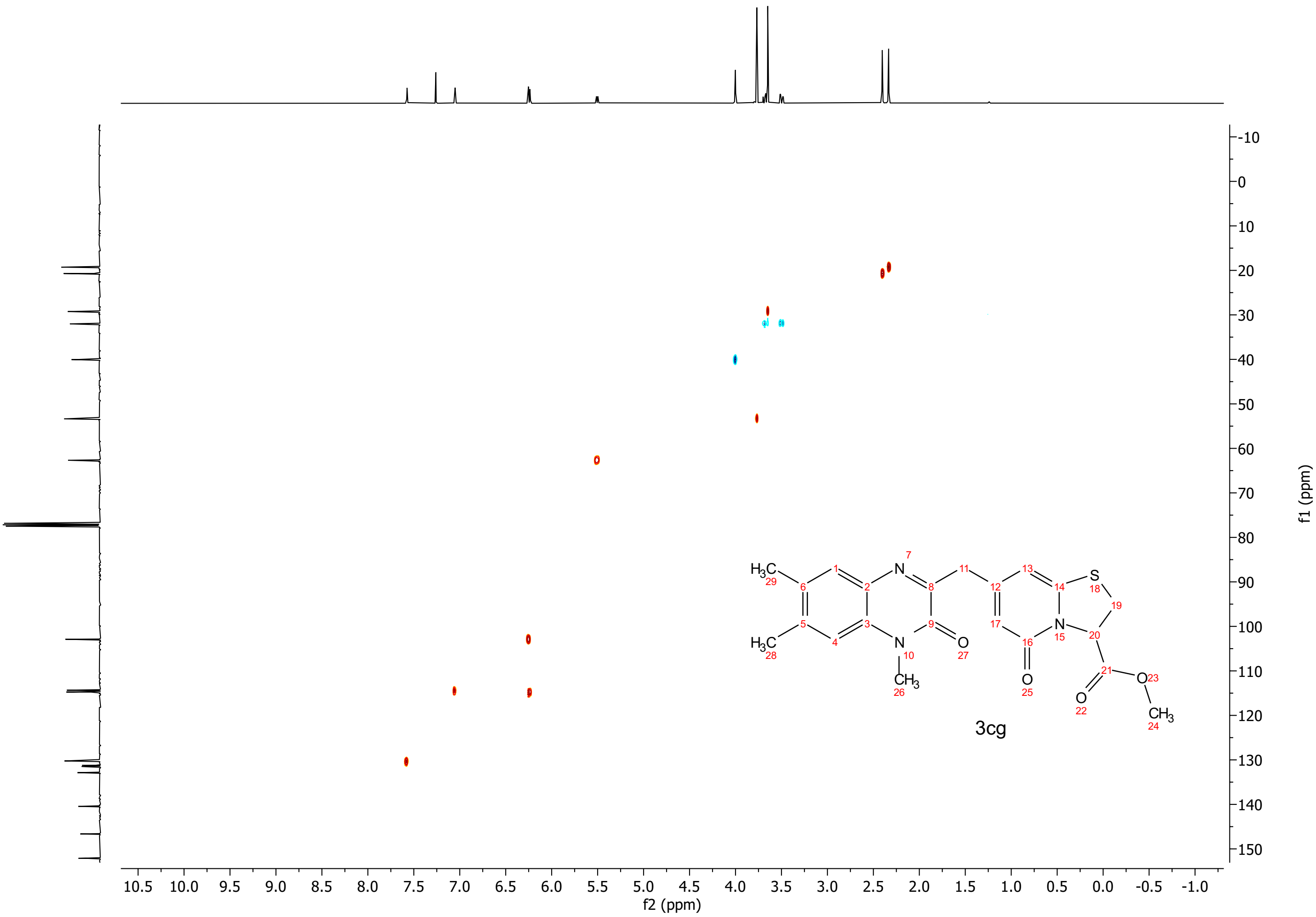

Figure S.134: gHSQC spectrum ($CDCl_3$) of methyl 5-oxo-7-((4,6,7-trimethyl-3-oxo-3,4-dihydroquinoxalin-2-yl)methyl)-2,3-dihydro-5H-thiazolo[3,2-a]pyridine-3-carboxylate, **3cg**.

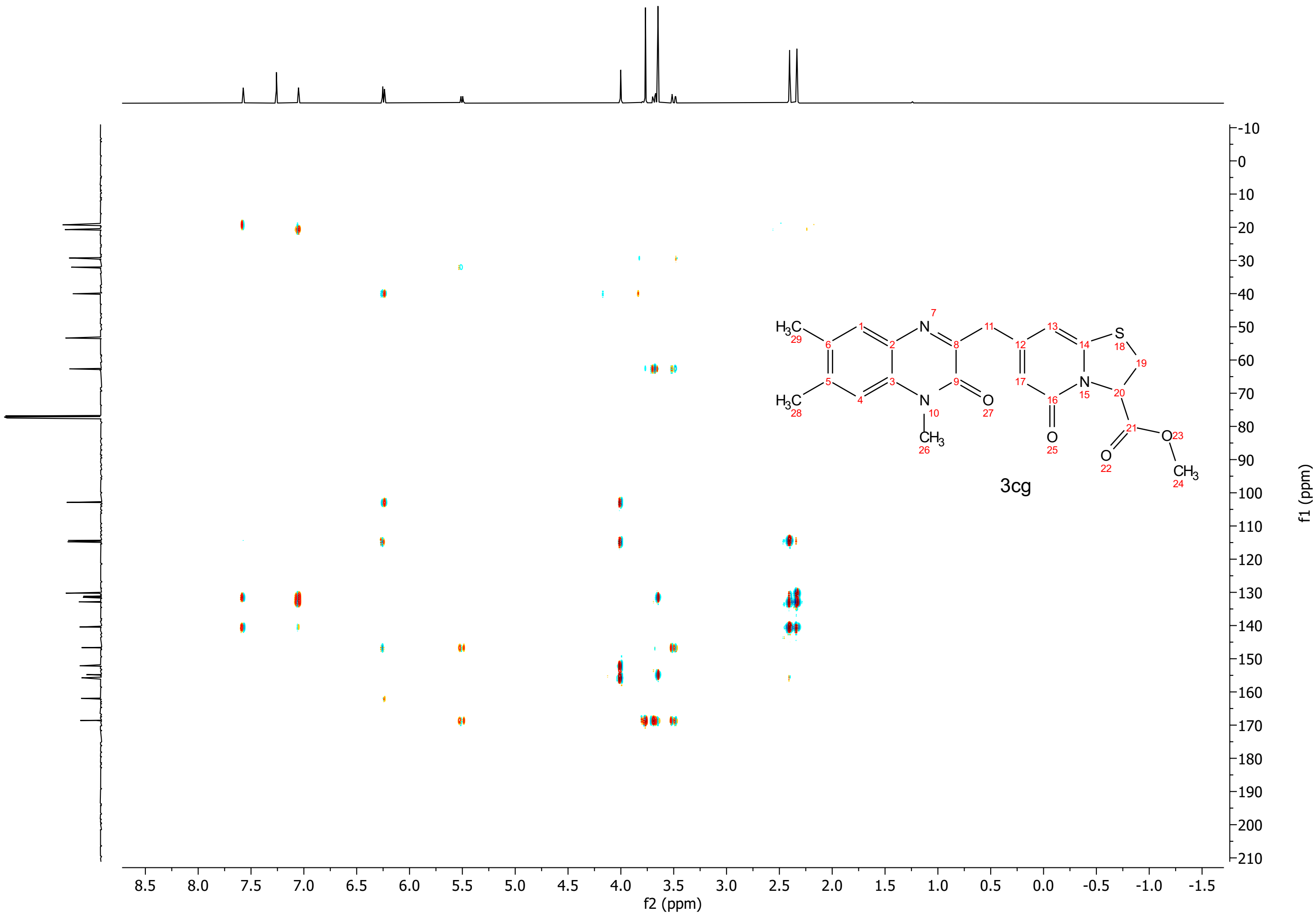

Figure S.135: gHMBC spectrum ($CDCl_3$) of methyl 5-oxo-7-((4,6,7-trimethyl-3-oxo-3,4-dihydroquinoxalin-2-yl)methyl)-2,3-dihydro-5H-thiazolo[3,2-a]pyridine-3-carboxylate, **3cg**.

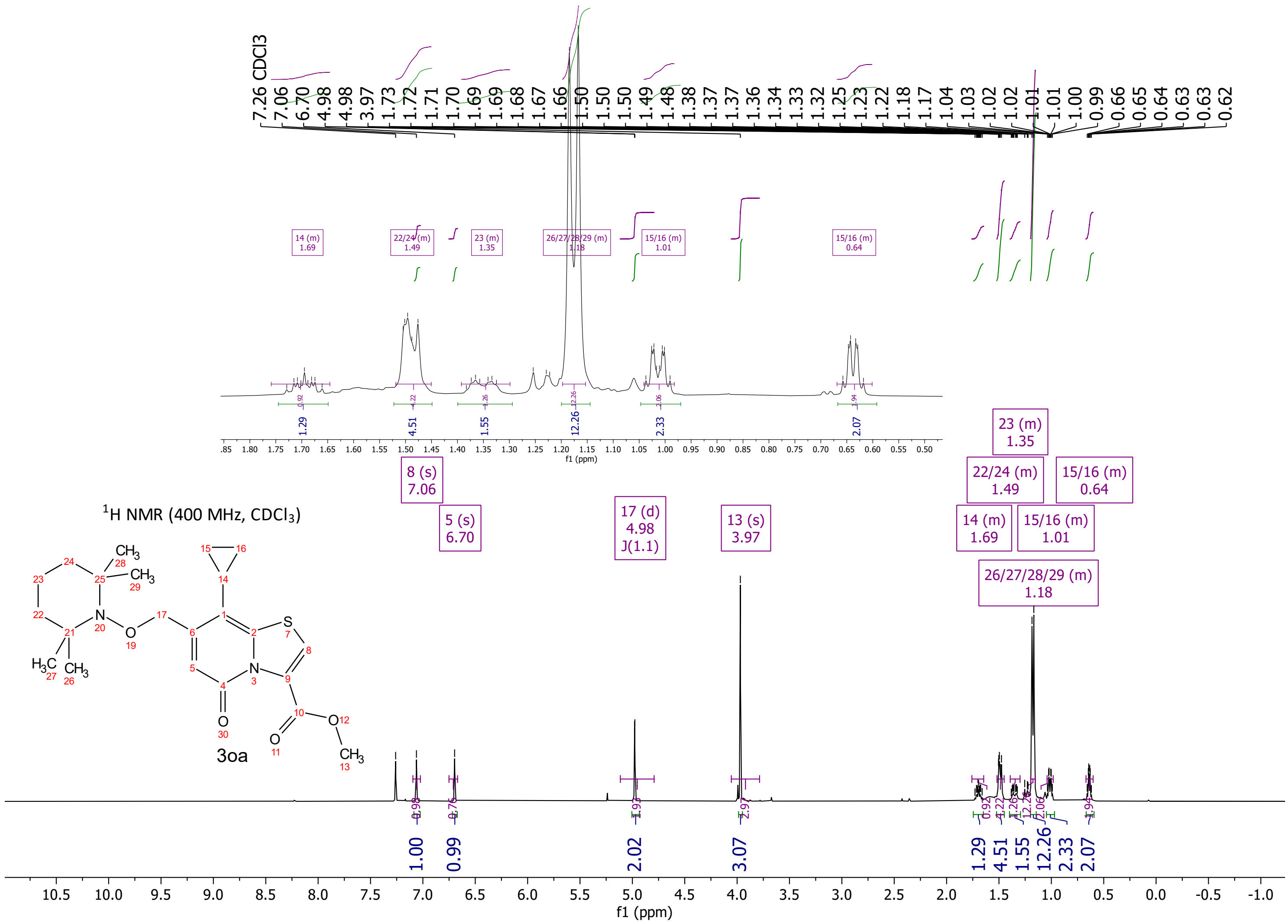

Figure S.136: ^{1}H NMR spectrum (CDCl$_3$, 400 MHz) of methyl 8-cyclopropyl-5-oxo-7-(((2,2,6,6-tetramethylpiperidin-1-yl)oxy)methyl)-5H-thiazolo[3,2-a]pyridine-3-carboxylate, **3oa**.

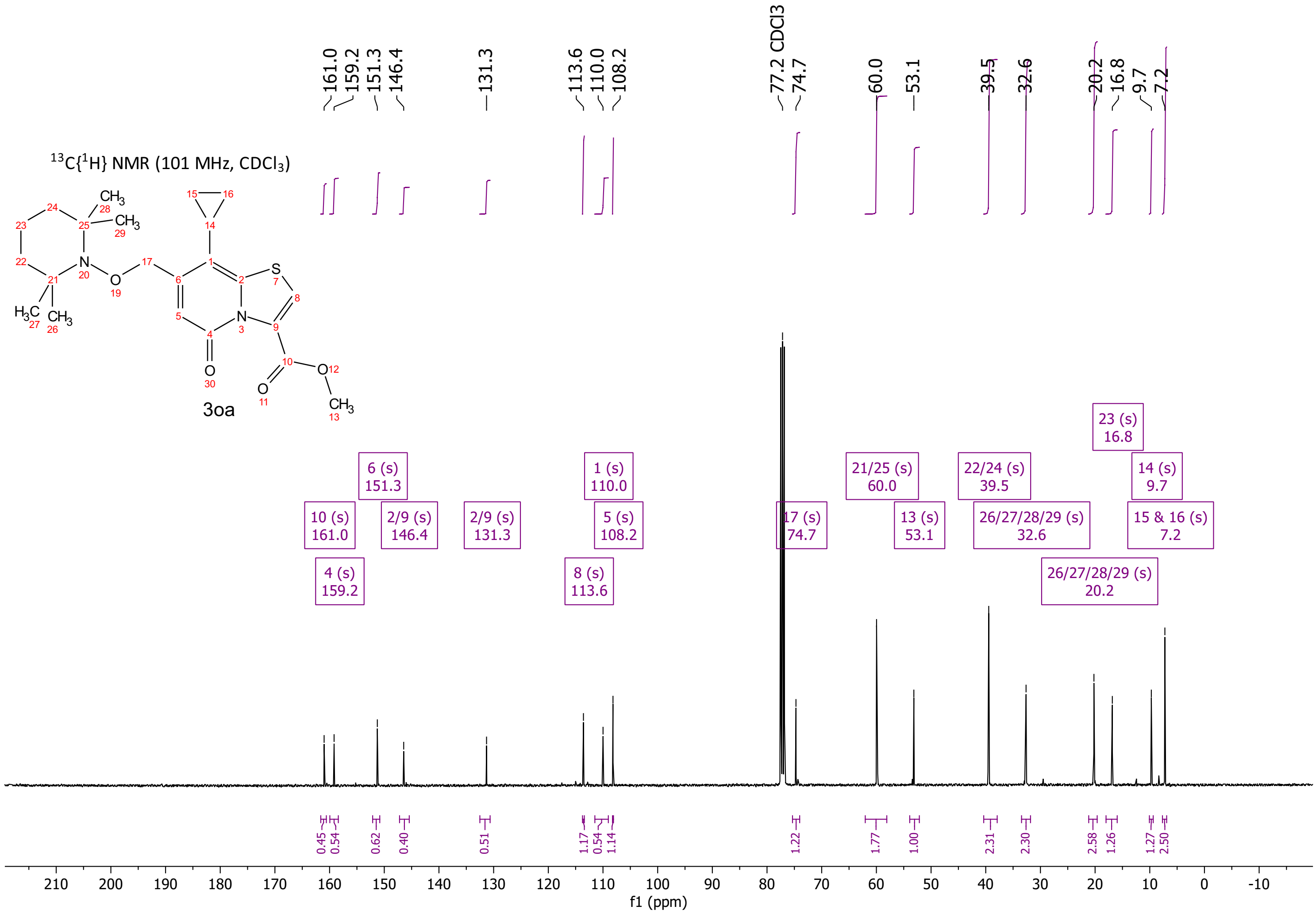

Figure S.137: $^{13}C\{^1H\}$ NMR spectrum ($CDCl_3$, 101 MHz) of methyl 8-cyclopropyl-5-oxo-7-(((2,2,6,6-tetramethylpiperidin-1-yl)oxy)methyl)-5H-thiazolo[3,2-a]pyridine-3-carboxylate, **3oa**.

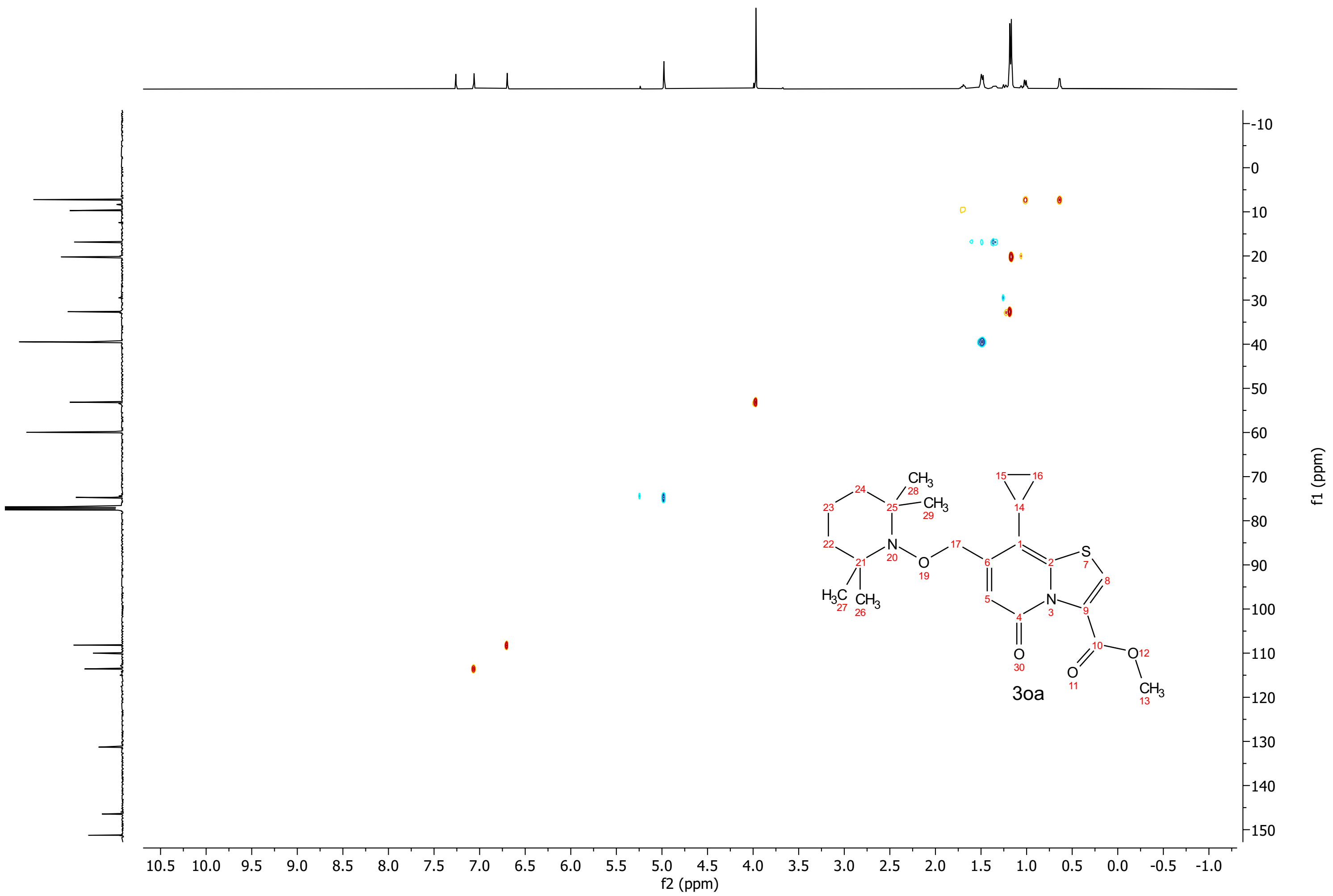

Figure S.138: gHSQC spectrum ($CDCl_3$) of methyl 8-cyclopropyl-5-oxo-7-(((2,2,6,6-tetramethylpiperidin-1-yl)oxy)methyl)-5H-thiazolo[3,2-a]pyridine-3-carboxylate, **3oa**.

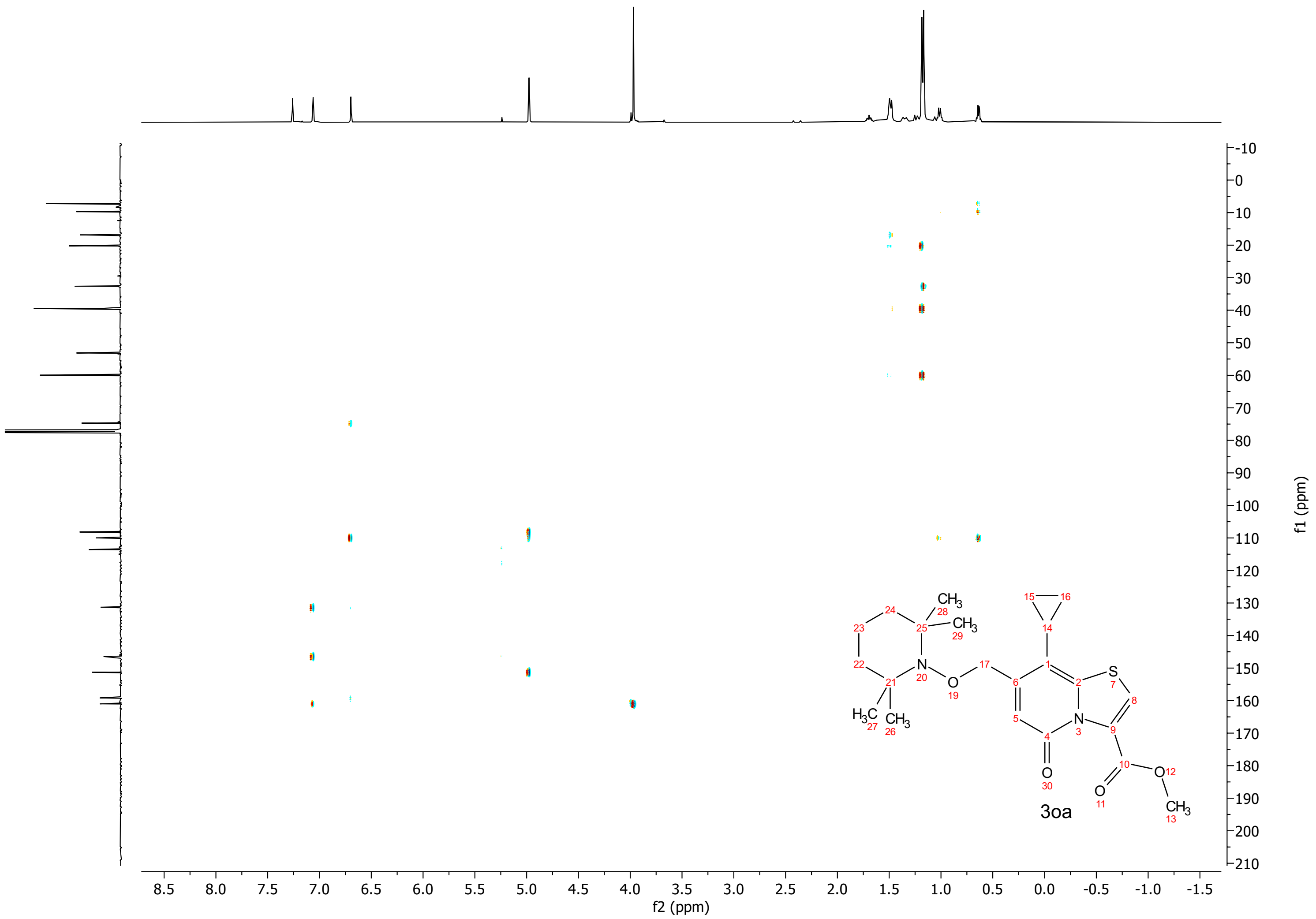

Figure S.139: gHMBC spectrum ($CDCl_3$) of methyl 8-cyclopropyl-5-oxo-7-(((2,2,6,6-tetramethylpiperidin-1-yl)oxy)methyl)-5H-thiazolo[3,2-a]pyridine-3-carboxylate, **3oa**.

H Copies of ATR-FTIR spectra

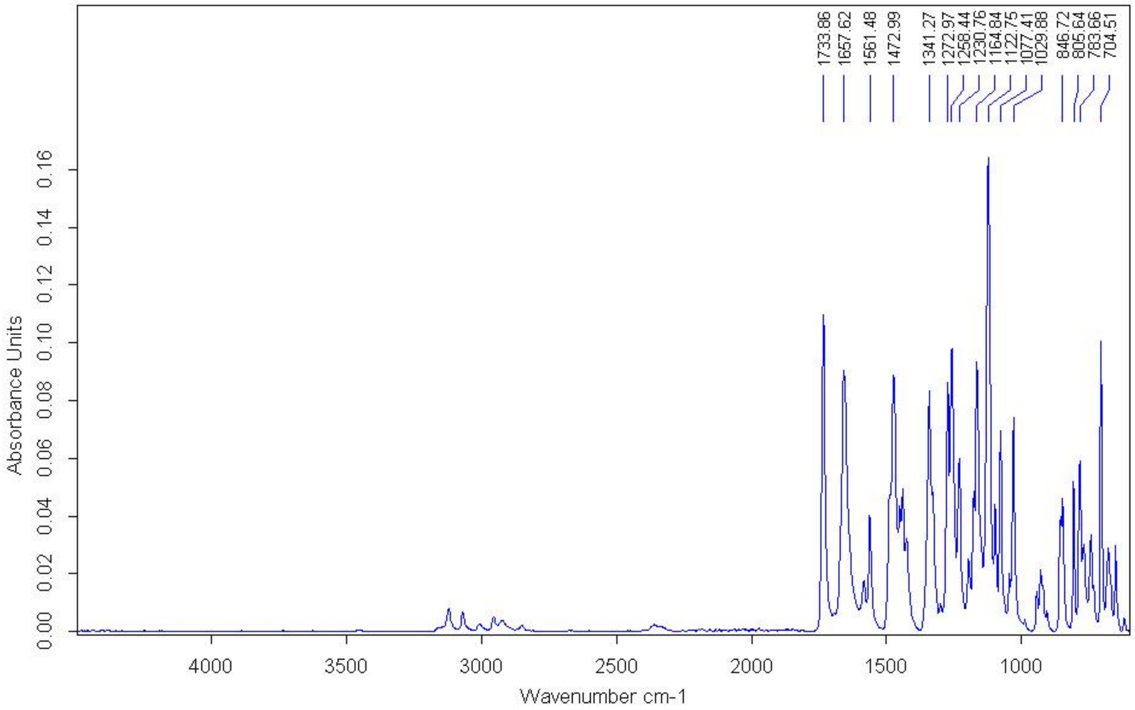

Figure S.140: ATR-FTIR spectrum (neat) of methyl 7-(chloromethyl)-5-oxo-8-(3-(trifluoromethyl)phenyl)-5H-thiazolo[3,2-a]pyridine-3-carboxylate, **2b**.

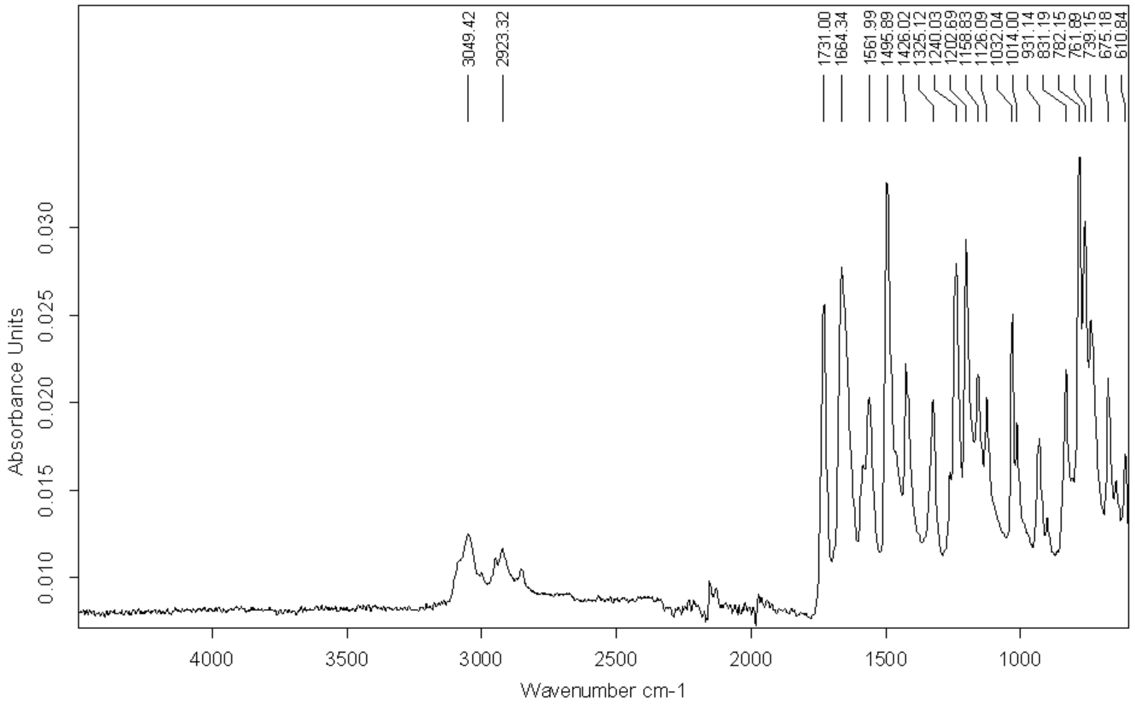

Figure S.141: ATR-FTIR spectrum (neat) of methyl 7-(chloromethyl)-5-oxo-5H-thiazolo[3,2-a]pyridine-3-carboxylate, **2c**.

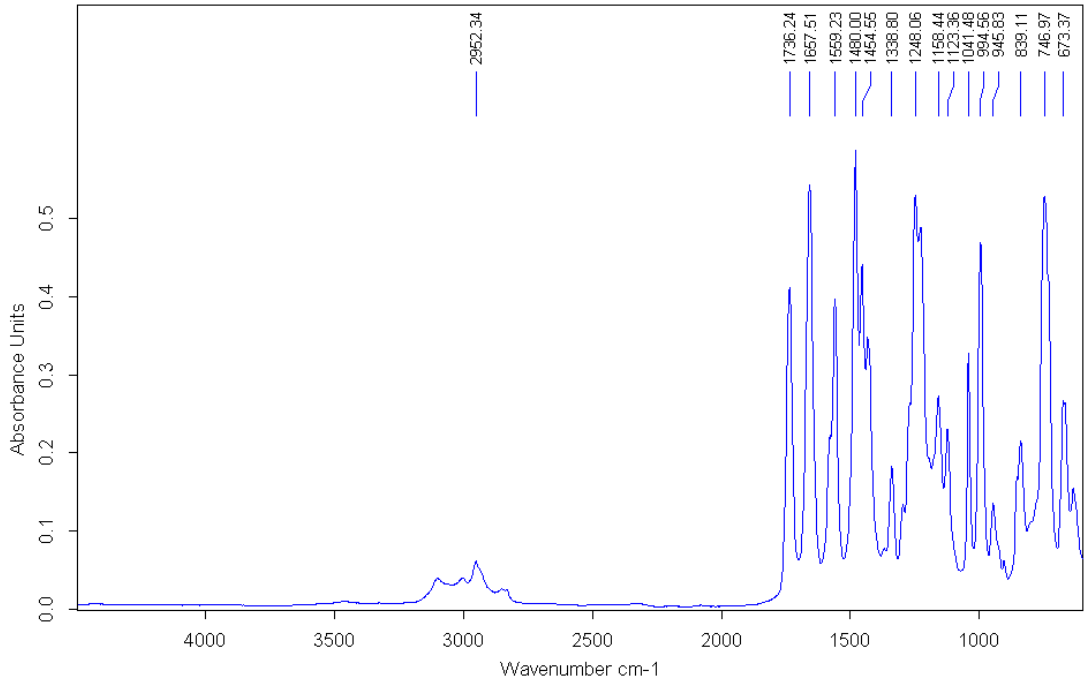

Figure S.142: ATR-FTIR spectrum (neat) of methyl 7-(chloromethyl)-8- methoxy-5-oxo-5H-thiazolo[3,2- a]pyridine-3-carboxylate, **2d**.

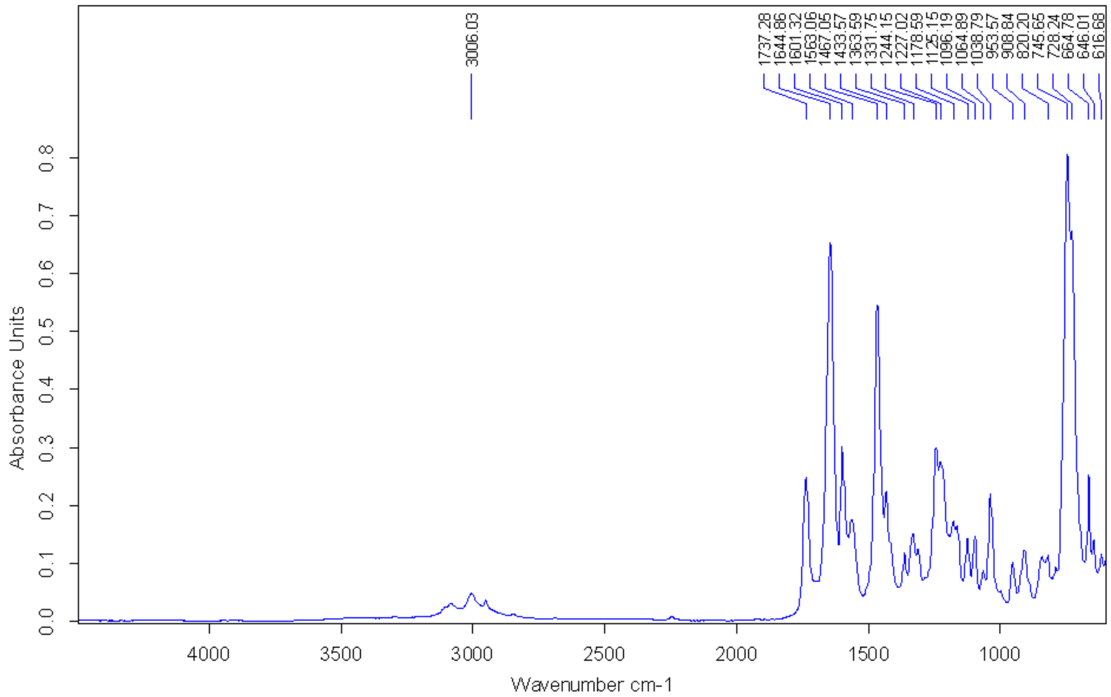

Figure S.143: ATR-FTIR spectrum (neat) of methyl 8-cyclopropyl-7-((4-methyl-3-oxo-3,4-dihydroquinoxalin-2-yl)methyl)-5-oxo-5H-thiazolo[3,2-a]pyridine-3-carboxylate, **3aa**.

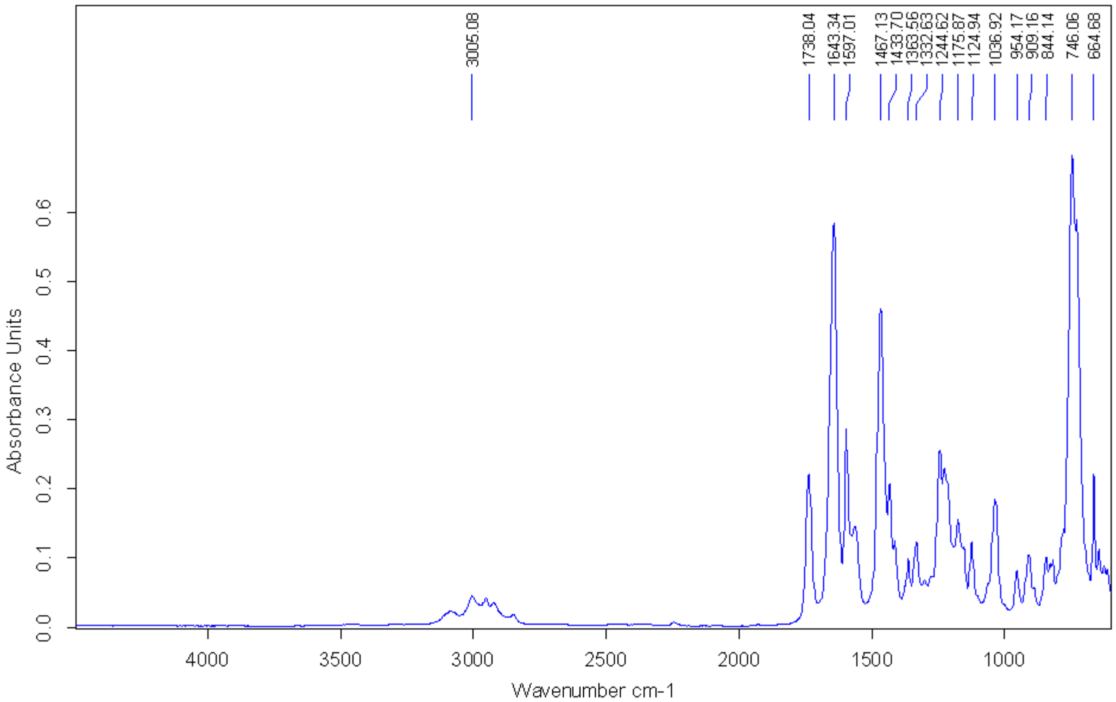

Figure S.144: ATR-FTIR spectrum (neat) of methyl 8-cyclopropyl-7-((4,8-dimethyl-3-oxo-3,4-dihydroquinoxalin-2-yl)methyl)-5-oxo-5H-thiazolo[3,2-a]pyridine-3-carboxylate, **3ba**.

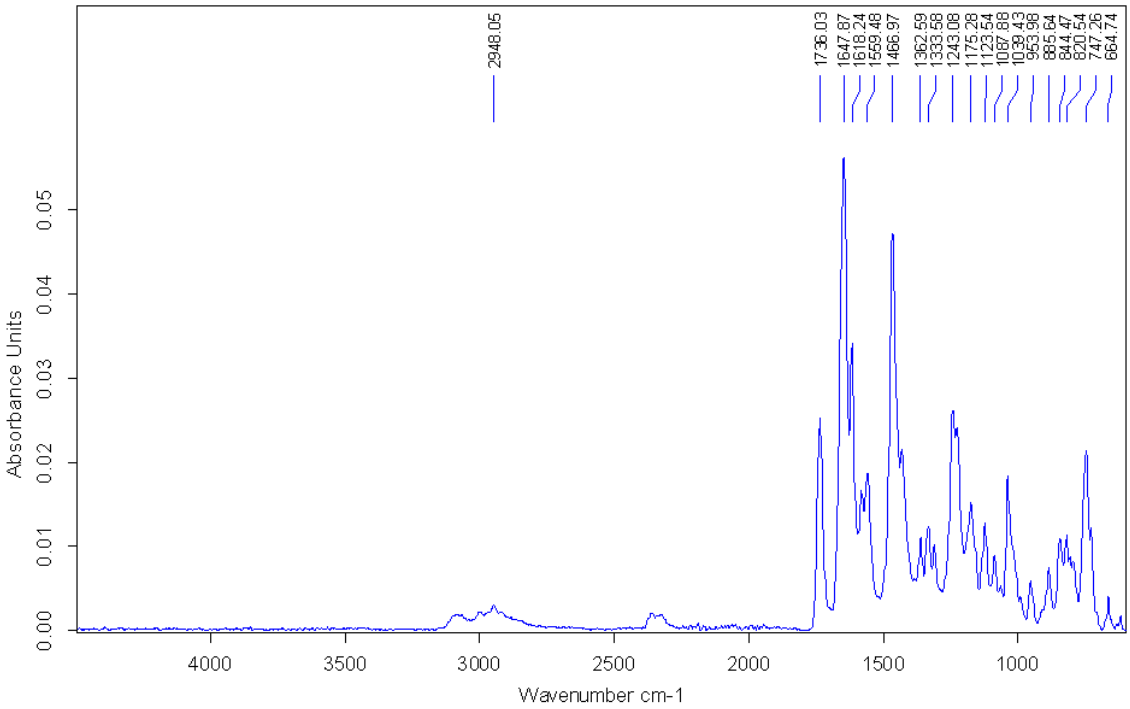

Figure S.145: ATR-FTIR spectrum (neat) of methyl 8-cyclopropyl-5-oxo-7-((4,6,7-trimethyl-3-oxo-3,4-dihydroquinoxalin-2-yl)methyl)-5H-thiazolo[3,2-a]pyridine-3-carboxylate, **3ca**.

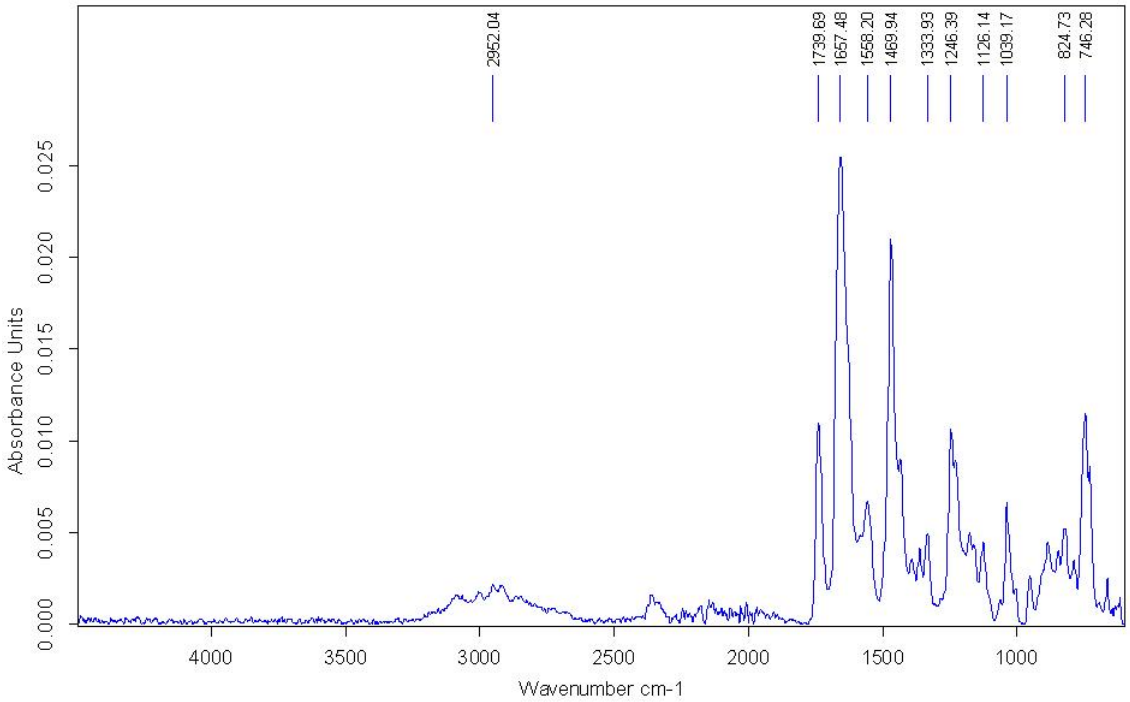

Figure S.146: ATR-FTIR spectrum (neat) of methyl 8-cyclopropyl-7-((6,7-dimethyl-3-oxo-3,4-dihydroquinoxalin-2-yl)methyl)-5-oxo-5H-thiazolo[3,2-a]pyridine-3-carboxylate, **3da**.

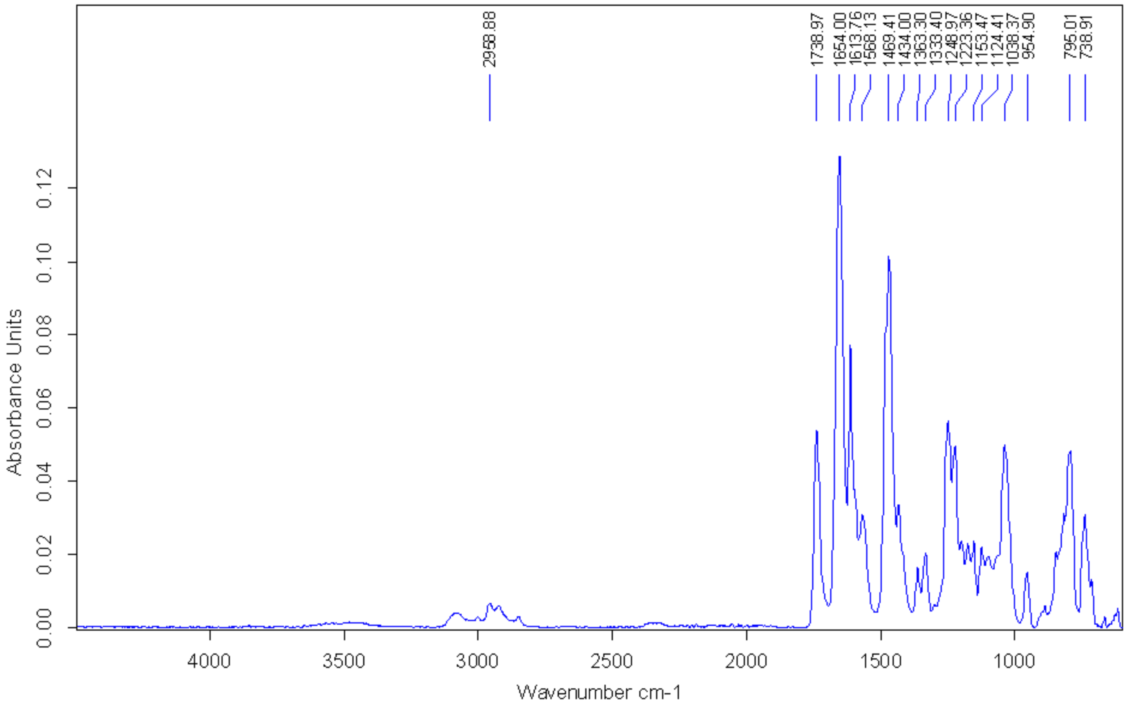

Figure S.147: ATR-FTIR spectrum (neat) of methyl 8-cyclopropyl-7-((8-fluoro-4-methyl-3-oxo-3,4-dihydroquinoxalin-2-yl)methyl)-5-oxo-5H-thiazolo[3,2-a]pyridine-3-carboxylate, **3ea**.

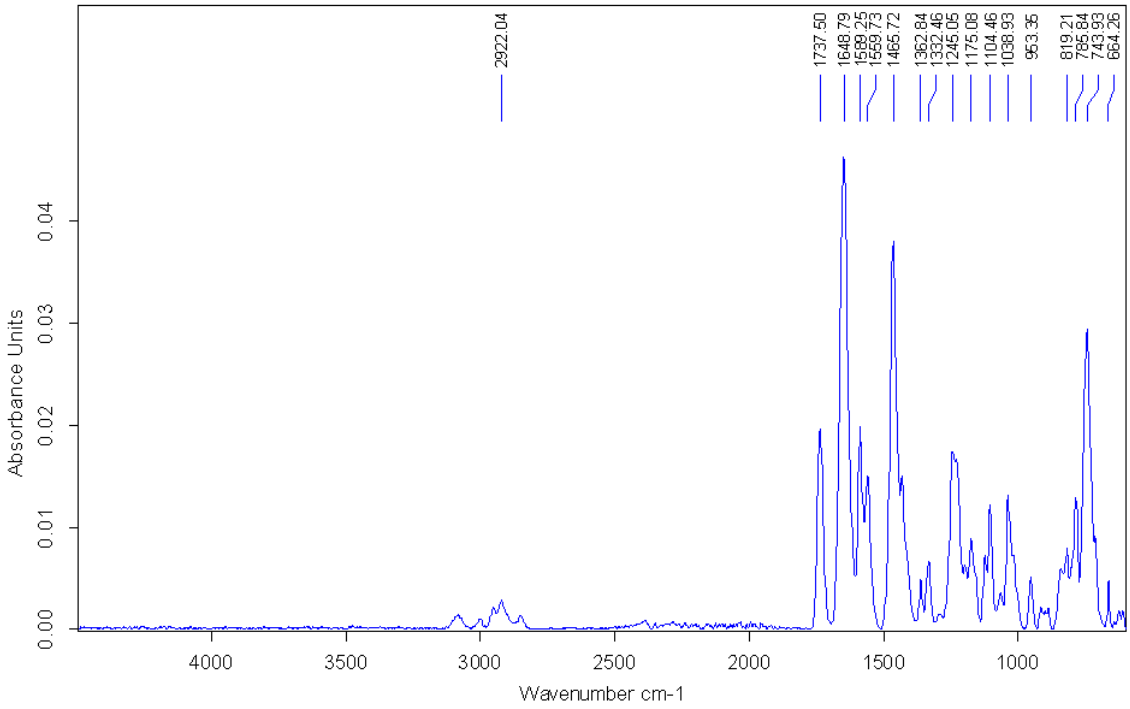

Figure S.148: ATR-FTIR spectrum (neat) of methyl 7-((8-bromo-4-methyl-3-oxo-3,4-dihydroquinoxalin-2-yl)methyl)-8-cyclopropyl-5-oxo-5H-thiazolo[3,2-a]pyridine-3-carboxylate, **3fa**.

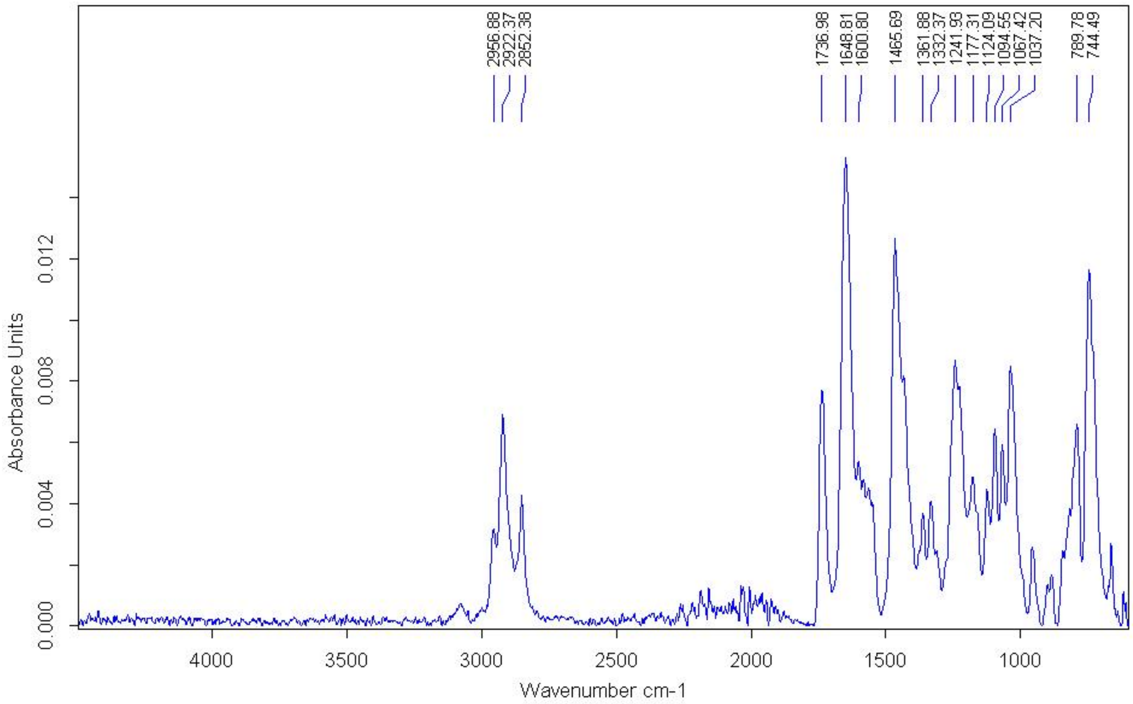

Figure S.149: ATR-FTIR spectrum (neat) of methyl 7-((5-bromo-4-methyl-3-oxo-3,4-dihydroquinoxalin-2-yl)methyl)-8-cyclopropyl-5-oxo-5H-thiazolo[3,2-a]pyridine-3-carboxylate, **3ga**.

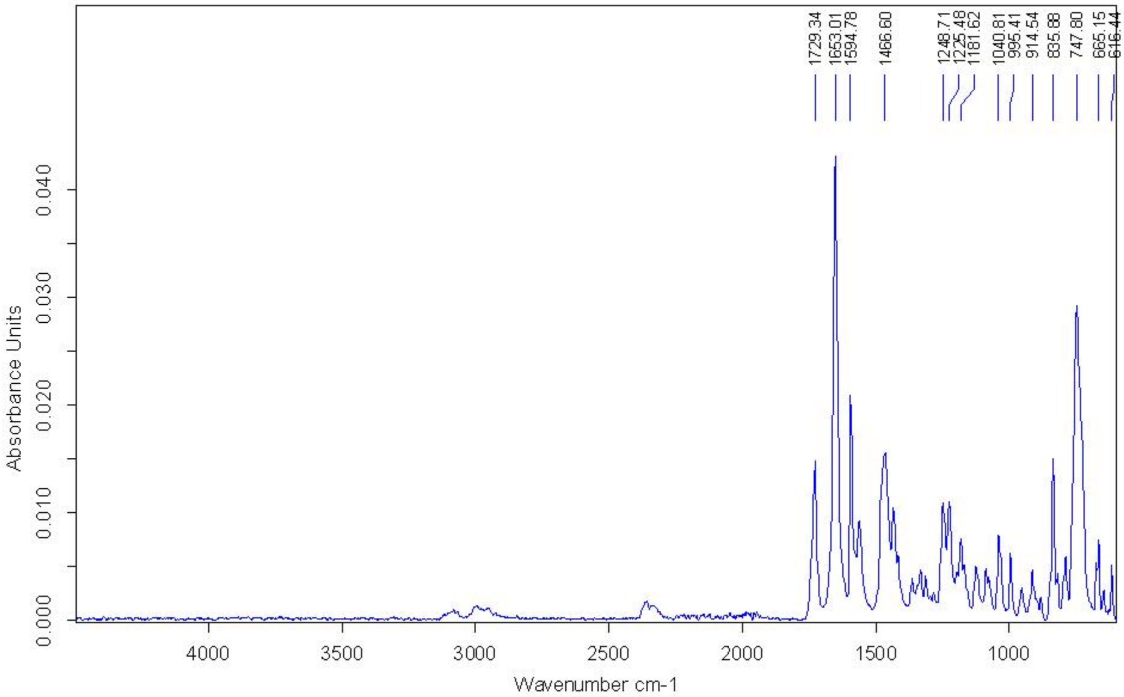

Figure S.150: ATR-FTIR spectrum (neat) of methyl 7-((6-bromo-4-methyl-3-oxo-3,4-dihydroquinoxalin-2-yl)methyl)-8-cyclopropyl-5-oxo-5H-thiazolo[3,2-a]pyridine-3-carboxylate, **3ha**.

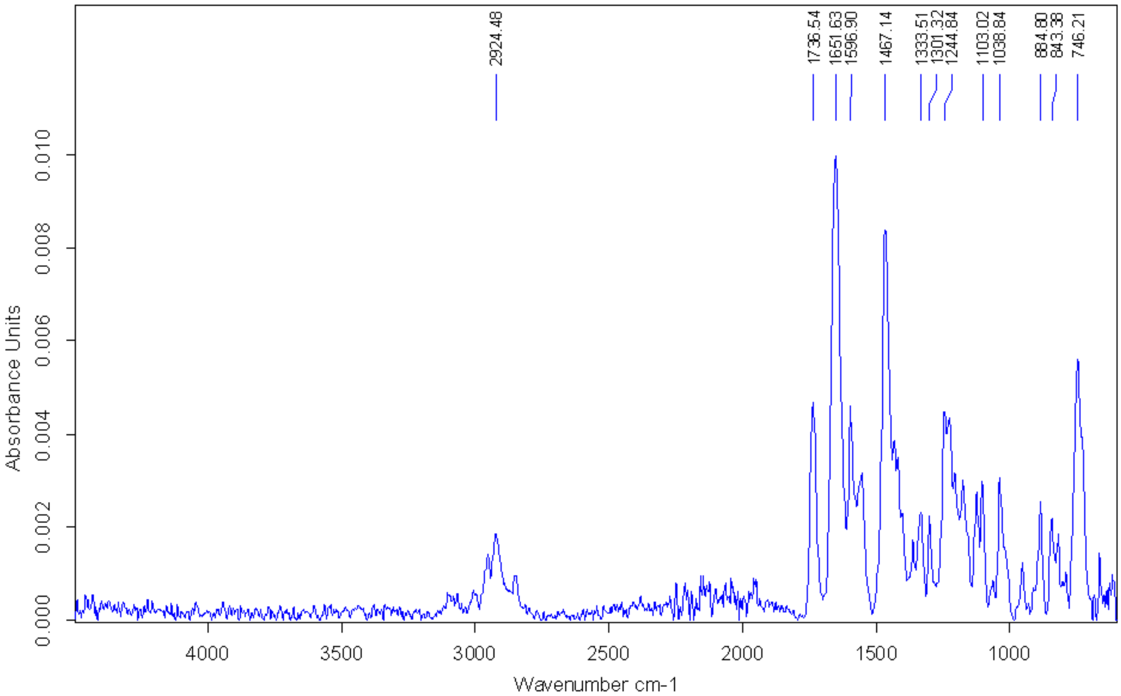

Figure S.151: ATR-FTIR spectrum (neat) of methyl 8-cyclopropyl-7-((6,7-dichloro-4-methyl-3-oxo-3,4-dihydroquinoxalin-2-yl)methyl)-5-oxo-5H-thiazolo[3,2-a]pyridine-3-carboxylate, **3ia**.

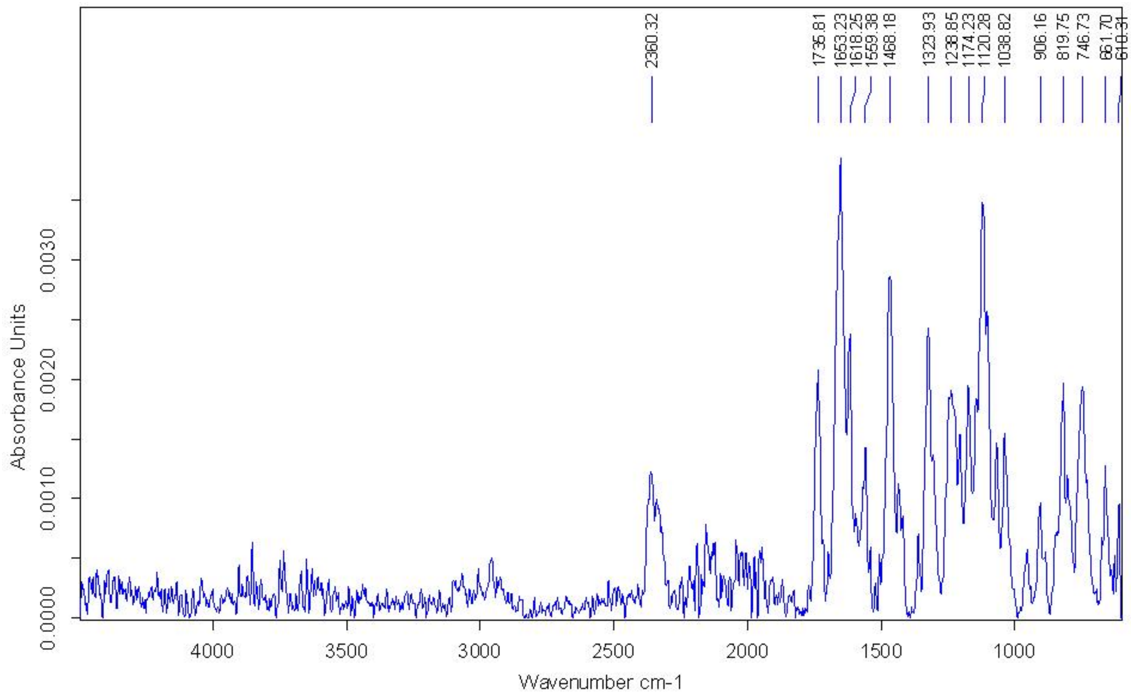

Figure S.152: ATR-FTIR spectrum (neat) of methyl 8-cyclopropyl-7-((4-methyl-3-oxo-7-(trifluoromethyl)-3,4-dihydroquinoxalin-2-yl)methyl)-5-oxo-5H-thiazolo[3,2-a]pyridine-3-carboxylate, **3ja**.

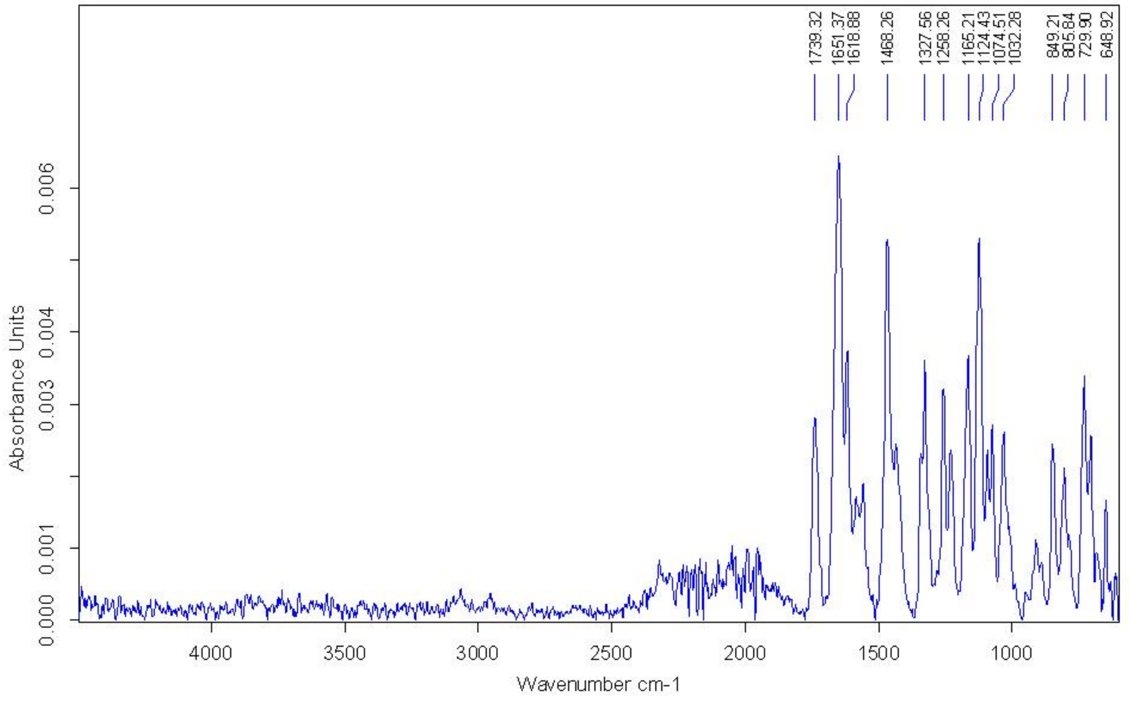

Figure S.153: ATR-FTIR spectrum (neat) of methyl 5-oxo-8-(3-(trifluoromethyl)phenyl)-7-((4,6,7-trimethyl-3-oxo-3,4-dihydroquinoxalin-2-yl)methyl)-5H-thiazolo[3,2-a]pyridine-3-carboxylate, **3cb**.

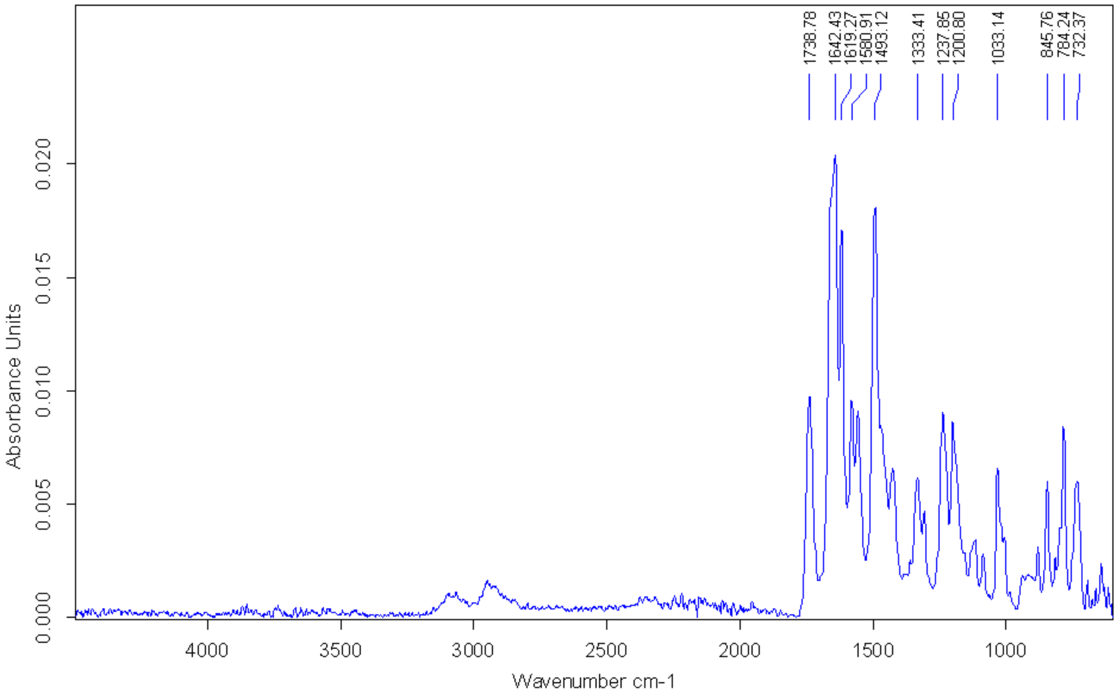

Figure S.154: ATR-FTIR spectrum (neat) of methyl 5-oxo-7-((4,6,7-trimethyl-3-oxo-3,4-dihydroquinoxalin-2-yl)methyl)-5H-thiazolo[3,2-a]pyridine-3-carboxylate, **3cc**.

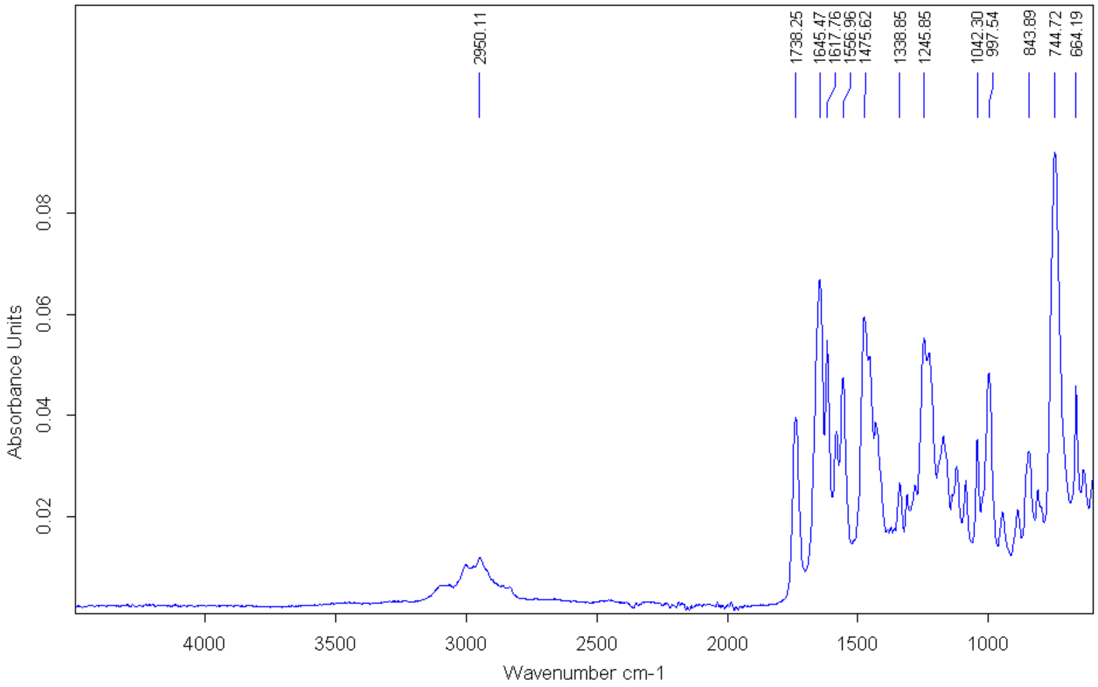

Figure S.155: ATR-FTIR spectrum (neat) of methyl 8-methoxy-5-oxo-7-((4,6,7-trimethyl-3-oxo-3,4-dihydroquinoxalin-2-yl)methyl)-5H-thiazolo[3,2-a]pyridine-3-carboxylate, **3cd**.

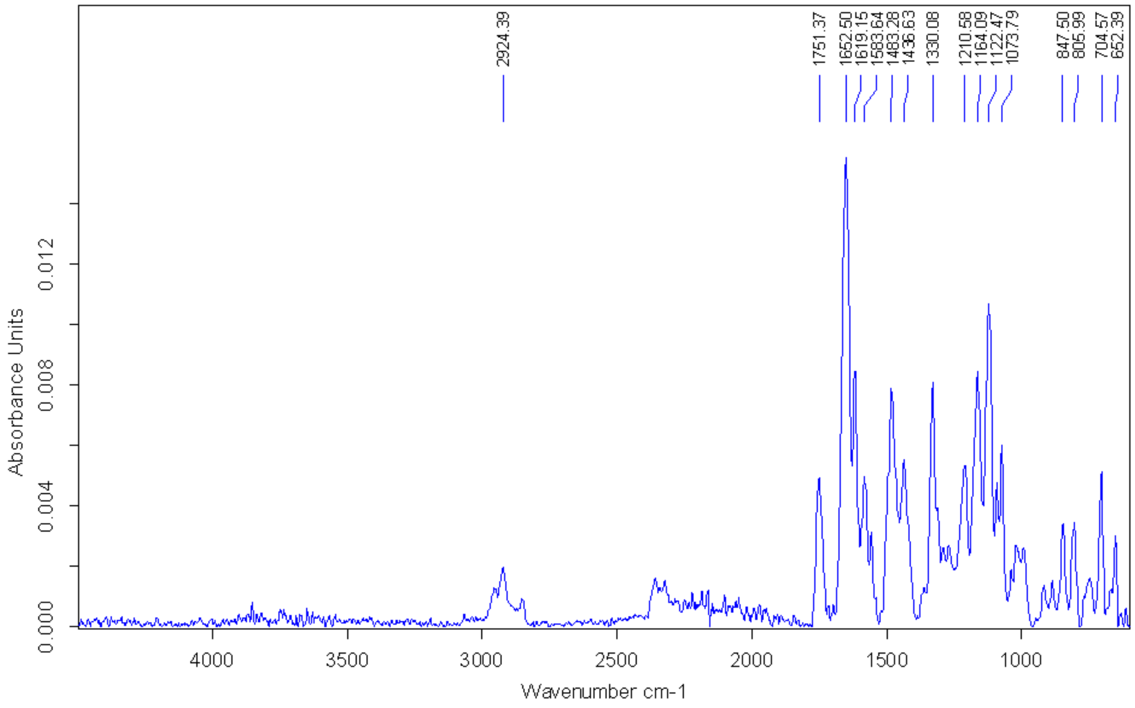

Figure S.156: ATR-FTIR spectrum (neat) of methyl 5-oxo-8-(3-(trifluoromethyl)phenyl)-7-((4,6,7-trimethyl-3-oxo-3,4-dihydroquinoxalin-2-yl)methyl)-2,3-dihydro-5H-thiazolo[3,2-a]pyridine-3-carboxylate, **3ce**.

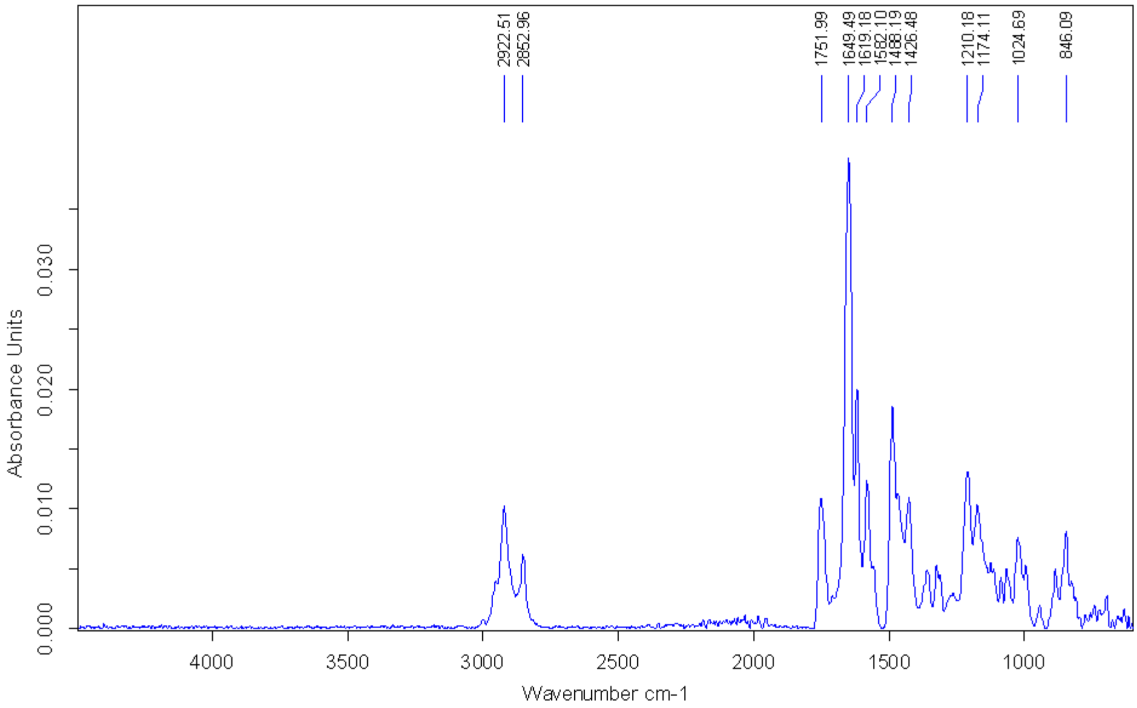

Figure S.157: ATR-FTIR spectrum (neat) of methyl 8-cyclopropyl-5-oxo-7-((4,6,7-trimethyl-3-oxo-3,4-dihydroquinoxalin-2-yl)methyl)-2,3-dihydro-5H-thiazolo[3,2-a]pyridine-3-carboxylate, **3cf**.

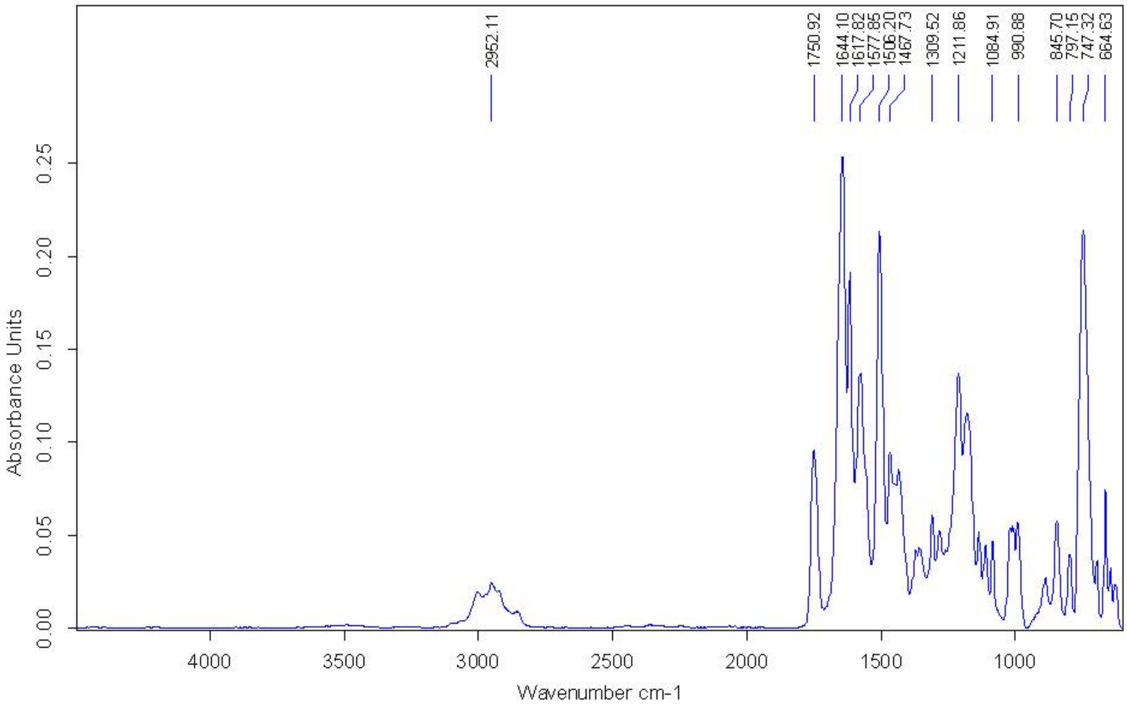

Figure S.158: ATR-FTIR spectrum (neat) of methyl 5-oxo-7-((4,6,7-trimethyl-3-oxo-3,4-dihydroquinoxalin-2-yl)methyl)-2,3-dihydro-5H-thiazolo[3,2-a]pyridine-3-carboxylate, **3cg**.

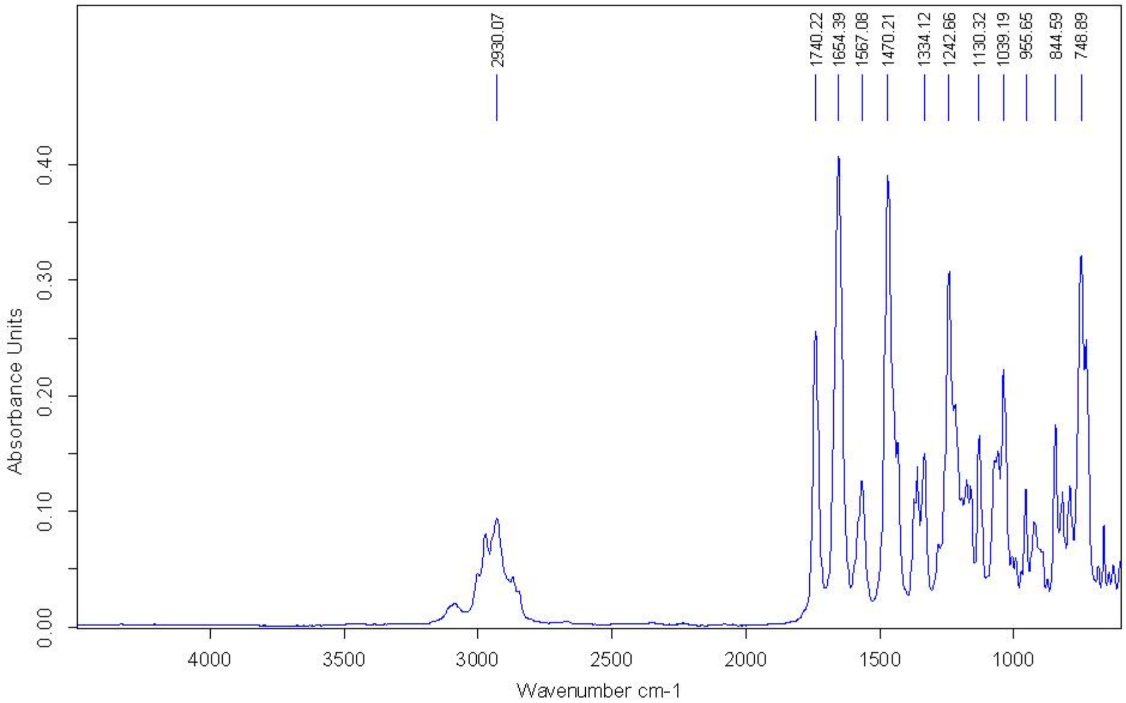

Figure S.159: ATR-FTIR spectrum (neat) of methyl 8-cyclopropyl-5-oxo-7-(((2,2,6,6-tetramethylpiperidin-1-yl)oxy)methyl)-5H-thiazolo[3,2-a]pyridine-3-carboxylate, **3oa**.

I Copies of Mass spectra

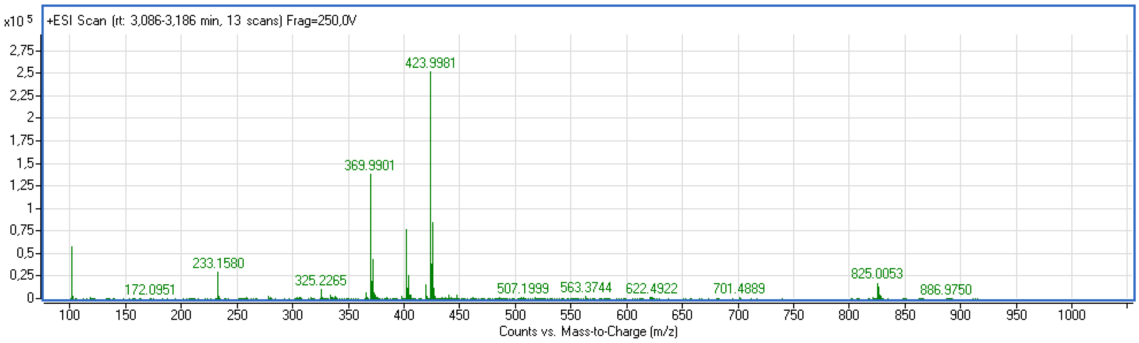

Figure S.160: MS of methyl 7-(chloromethyl)-5-oxo-8-(3-(trifluoromethyl)phenyl)-5H-thiazolo[3,2-a]pyridine-3-carboxylate, **2b**.

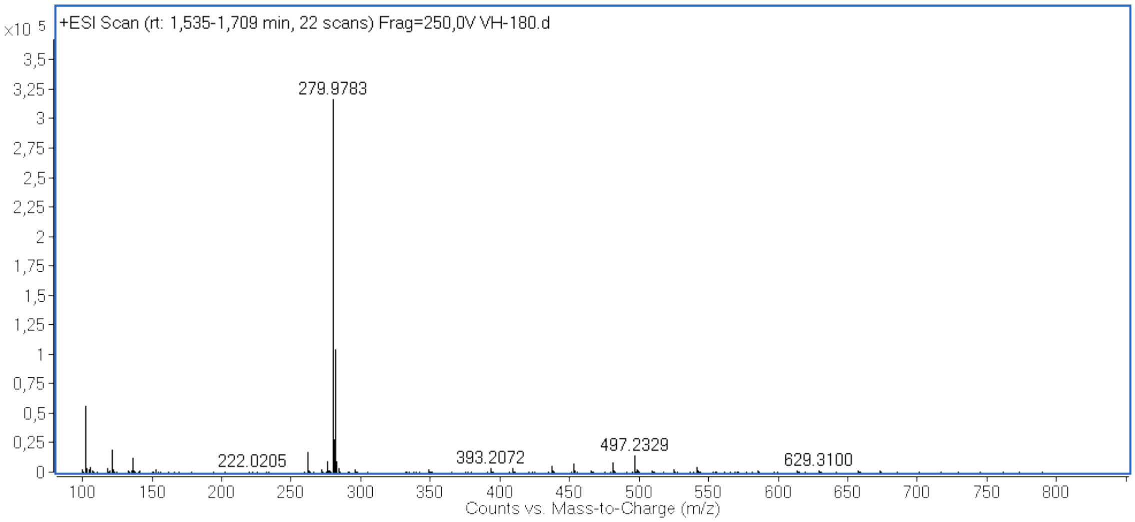

Figure S.161: MS of methyl 7-(chloromethyl)-5-oxo-5H-thiazolo[3,2-a]pyridine-3-carboxylate, **2c**.

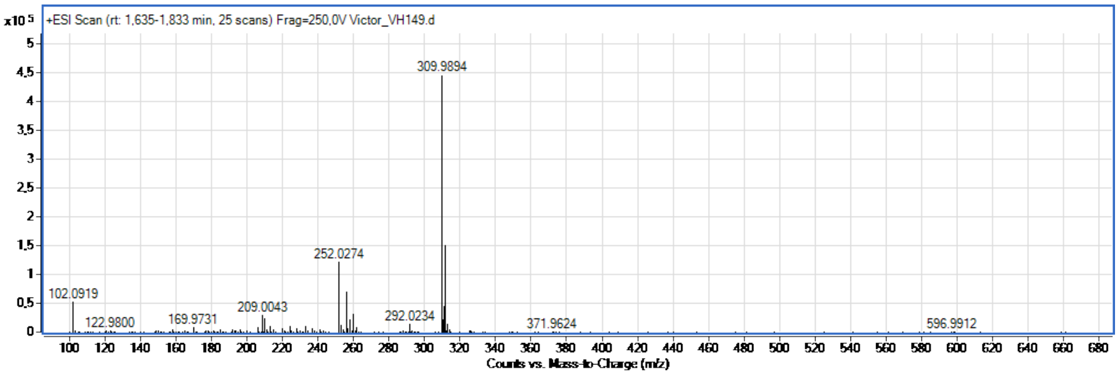

Figure S.162: MS of methyl 7-(chloromethyl)-8- methoxy-5-oxo-5H-thiazolo[3,2-a]pyridine-3-carboxylate, **2d**.

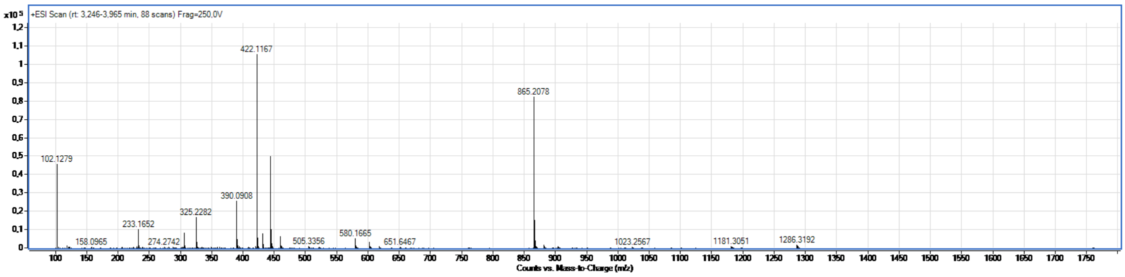

Figure S.163: MS of methyl 8-cyclopropyl-7-((4-methyl-3-oxo-3,4-dihydroquinoxalin-2-yl)methyl)-5-oxo-5H-thiazolo[3,2-a]pyridine-3-carboxylate, **3aa**.

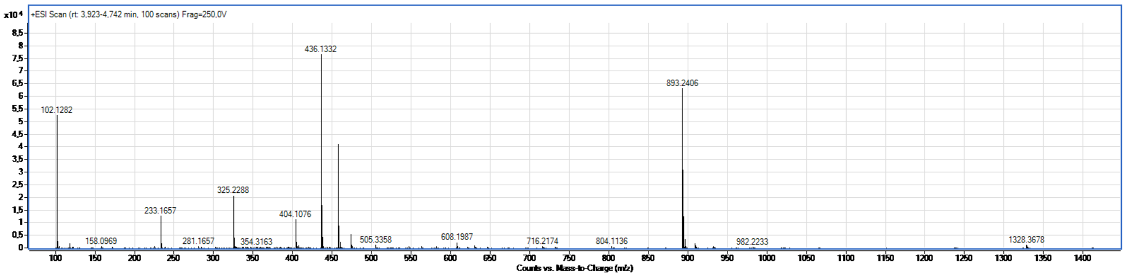

Figure S.164: MS of methyl 8-cyclopropyl-7-((4,8-dimethyl-3-oxo-3,4-dihydroquinoxalin-2-yl)methyl)-5-oxo-5H-thiazolo[3,2-a]pyridine-3-carboxylate, **3ba**.

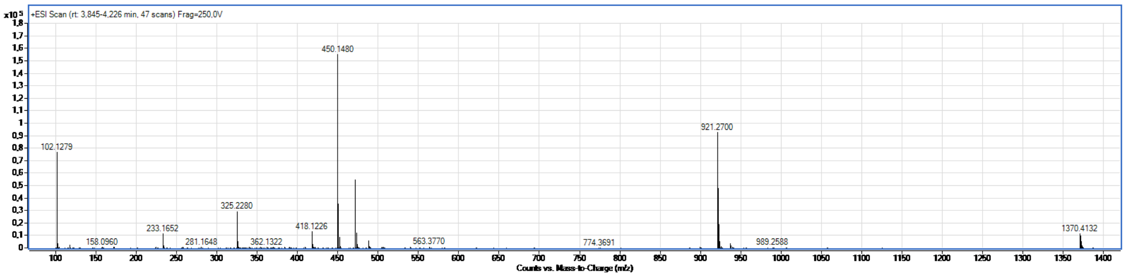

Figure S.165: MS of methyl 8-cyclopropyl-5-oxo-7-((4,6,7-trimethyl-3-oxo-3,4-dihydroquinoxalin-2-yl)methyl)-5H-thiazolo[3,2-a]pyridine-3-carboxylate, **3ca**.

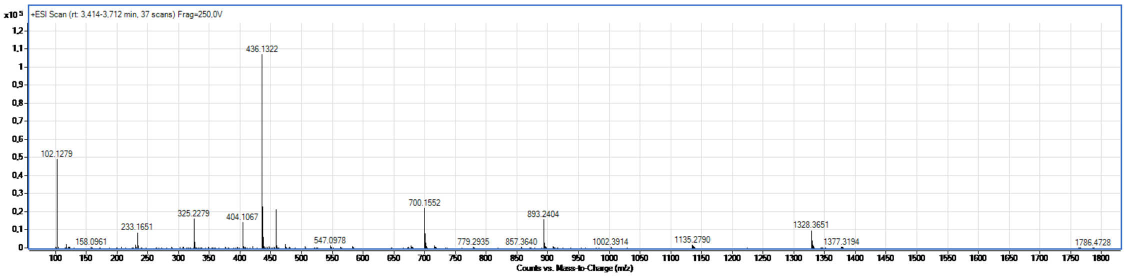

Figure S.166: MS of methyl 8-cyclopropyl-7-((6,7-dimethyl-3-oxo-3,4-dihydroquinoxalin-2-yl)methyl)-5-oxo-5H-thiazolo[3,2-a]pyridine-3-carboxylate, **3da**.

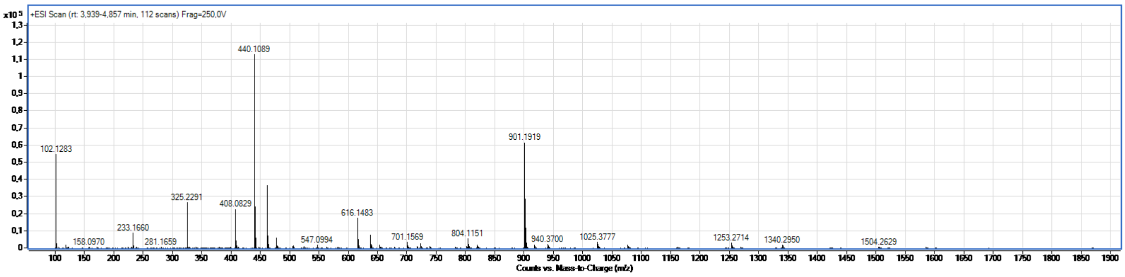

Figure S.167: MS (neat) of methyl 8-cyclopropyl-7-((8-fluoro-4-methyl-3-oxo-3,4-dihydroquinoxalin-2-yl)methyl)-5-oxo-5H-thiazolo[3,2-a]pyridine-3-carboxylate, **3ea**.

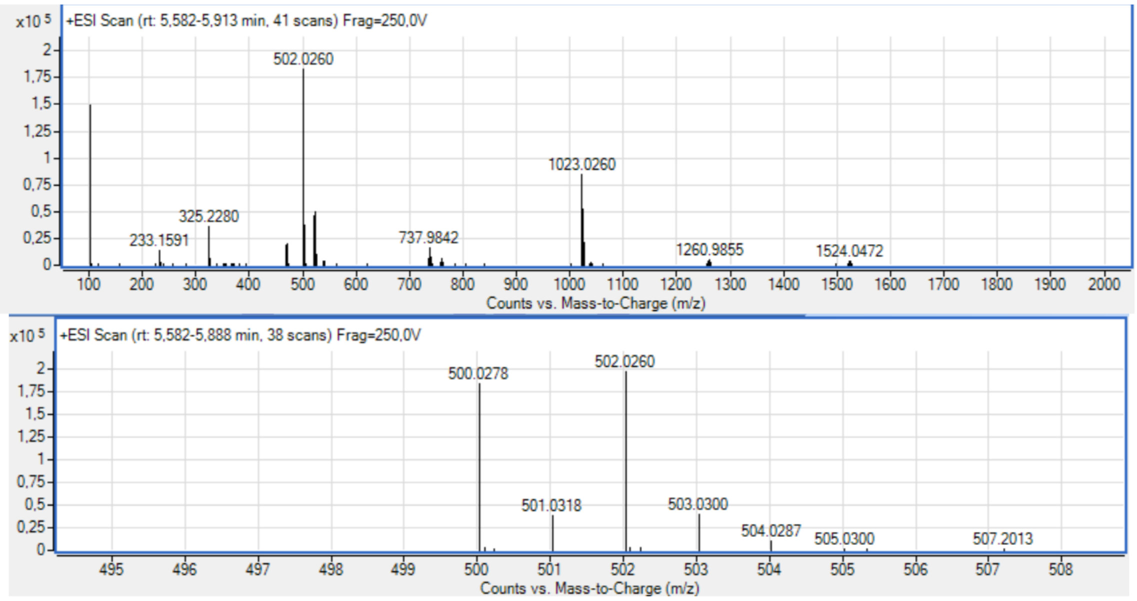

Figure S.168: MS of methyl 7-((8-bromo-4-methyl-3-oxo-3,4-dihydroquinoxalin-2-yl)methyl)-8-cyclopropyl-5-oxo-5H-thiazolo[3,2-a]pyridine-3-carboxylate, **3fa** with expansion.

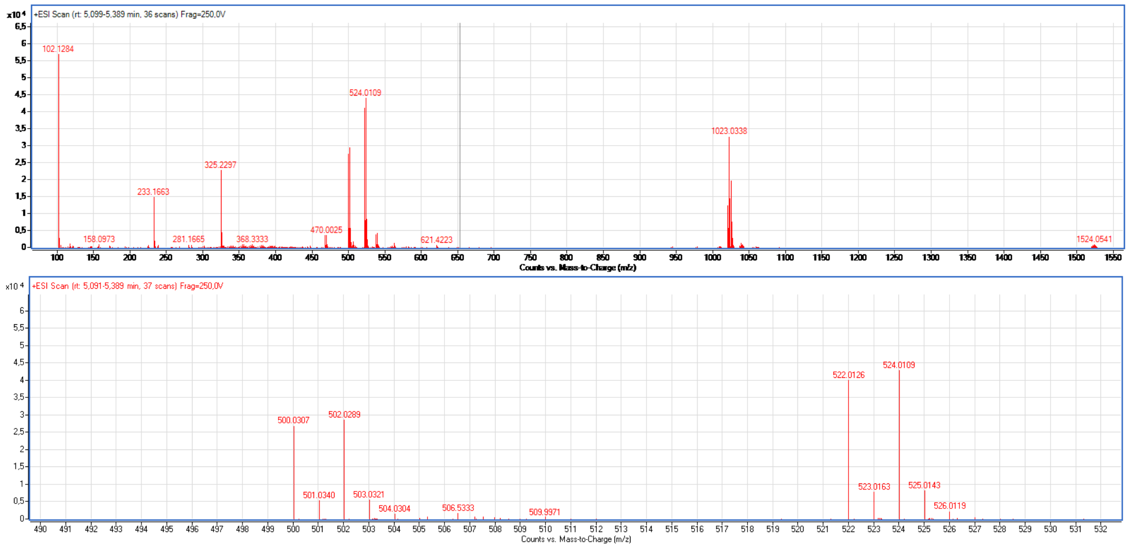

Figure S.169: MS of methyl 7-((5-bromo-4-methyl-3-oxo-3,4-dihydroquinoxalin-2-yl)methyl)-8-cyclopropyl-5-oxo-5H-thiazolo[3,2-a]pyridine-3-carboxylate, **3ga** with expansion.

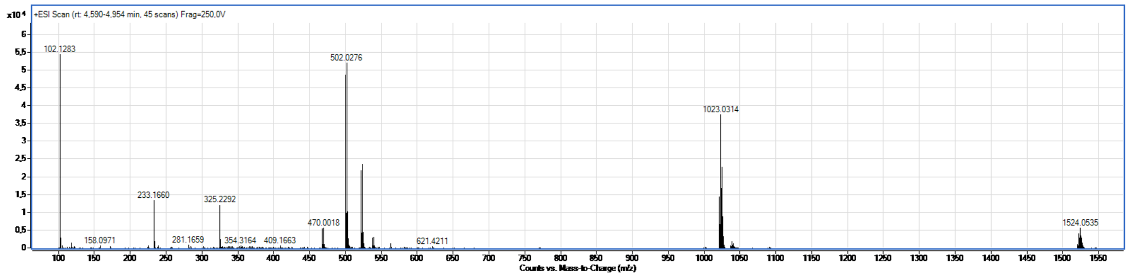

Figure S.170: MS of methyl 7-((6-bromo-4-methyl-3-oxo-3,4-dihydroquinoxalin-2-yl)methyl)-8-cyclopropyl-5-oxo-5H-thiazolo[3,2-a]pyridine-3-carboxylate, **3ha**.

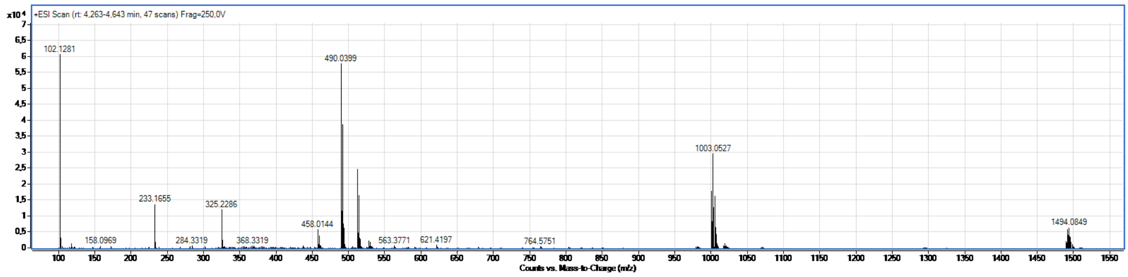

Figure S.171: MS of methyl 8-cyclopropyl-7-((6,7-dichloro-4-methyl-3-oxo-3,4-dihydroquinoxalin-2-yl)methyl)-5-oxo-5H-thiazolo[3,2-a]pyridine-3-carboxylate, **3ia**.

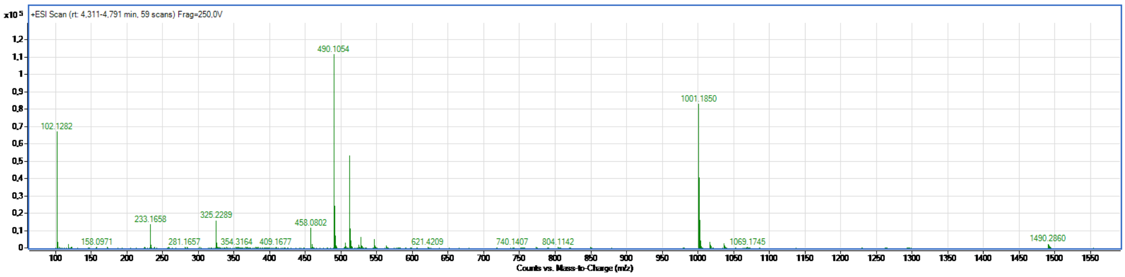

Figure S.172: MS of methyl 8-cyclopropyl-7-((4-methyl-3-oxo-7-(trifluoromethyl)-3,4-dihydroquinoxalin-2-yl)methyl)-5-oxo-5H-thiazolo[3,2-a]pyridine-3-carboxylate, **3ja**.

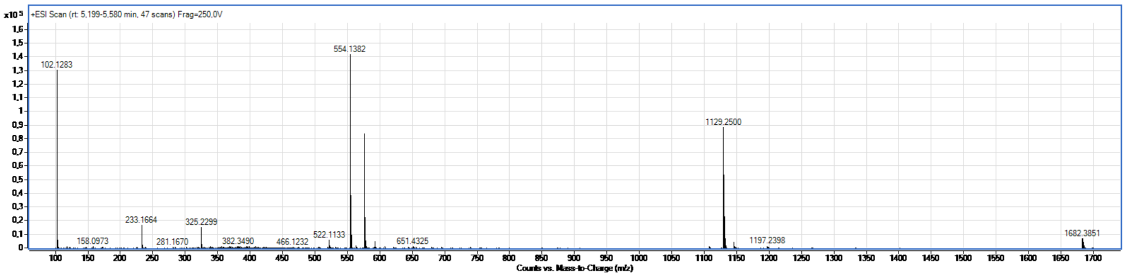

Figure S.173: MS of methyl 5-oxo-8-(3-(trifluoromethyl)phenyl)-7-((4,6,7-trimethyl-3-oxo-3,4-dihydroquinoxalin-2-yl)methyl)-5H-thiazolo[3,2-a]pyridine-3-carboxylate, **3cb**.

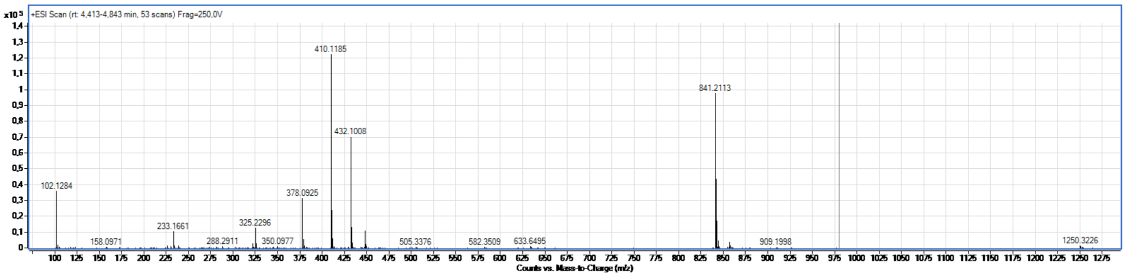

Figure S.174: MS of methyl 5-oxo-7-((4,6,7-trimethyl-3-oxo-3,4-dihydroquinoxalin-2-yl)methyl)-5H-thiazolo[3,2-a]pyridine-3-carboxylate, **3cc**.

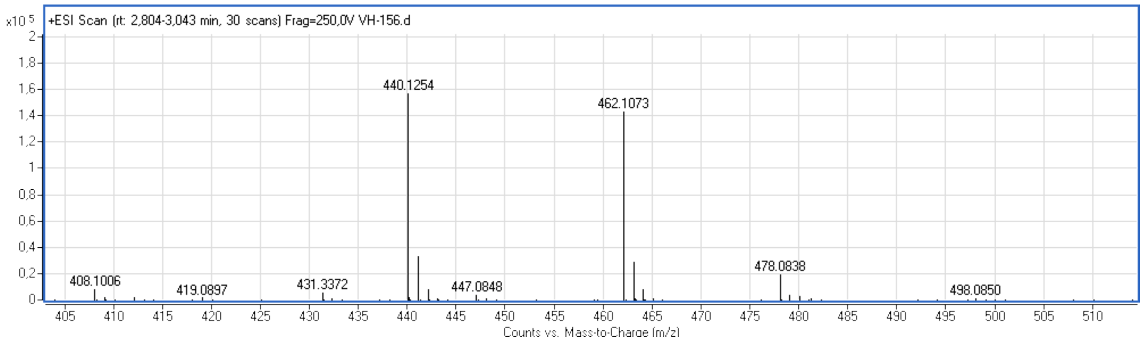

Figure S.175: MS of methyl 8-methoxy-5-oxo-7-((4,6,7-trimethyl-3-oxo-3,4-dihydroquinoxalin-2-yl)methyl)-5H-thiazolo[3,2-a]pyridine-3-carboxylate, **3cd**.

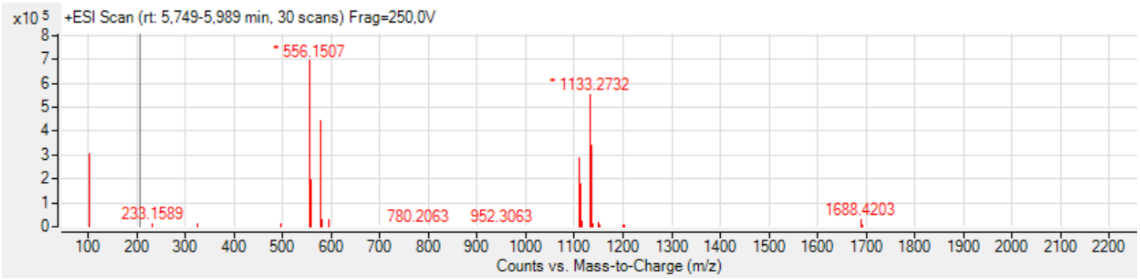

Figure S.176: MS of methyl 5-oxo-8-(3-(trifluoromethyl)phenyl)-7-((4,6,7-trimethyl-3-oxo-3,4-dihydroquinoxalin-2-yl)methyl)-2,3-dihydro-5H-thiazolo[3,2-a]pyridine-3-carboxylate, **3ce**.

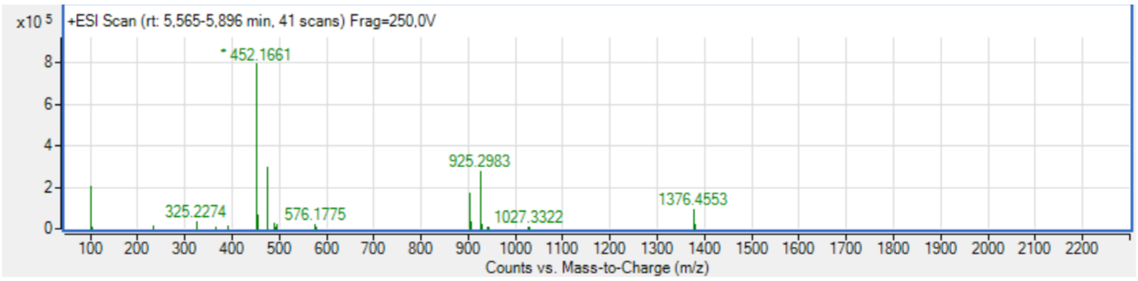

Figure S.177: MS of methyl 8-cyclopropyl-5-oxo-7-((4,6,7-trimethyl-3-oxo-3,4-dihydroquinoxalin-2-yl)methyl)-2,3-dihydro-5H-thiazolo[3,2-a]pyridine-3-carboxylate, **3cf**.

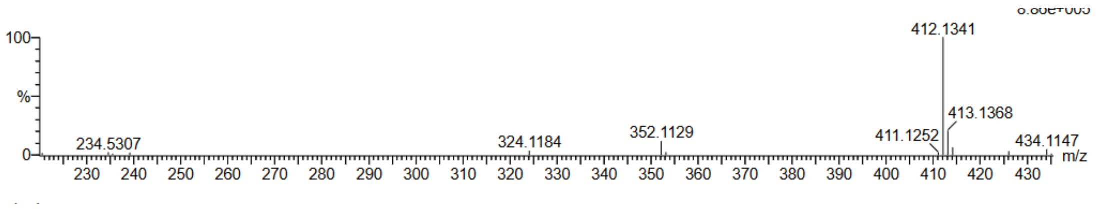

Figure S.178: MS of methyl 5-oxo-7-((4,6,7-trimethyl-3-oxo-3,4-dihydroquinoxalin-2-yl)methyl)-2,3-dihydro-5H-thiazolo[3,2-a]pyridine-3-carboxylate, **3cg**.

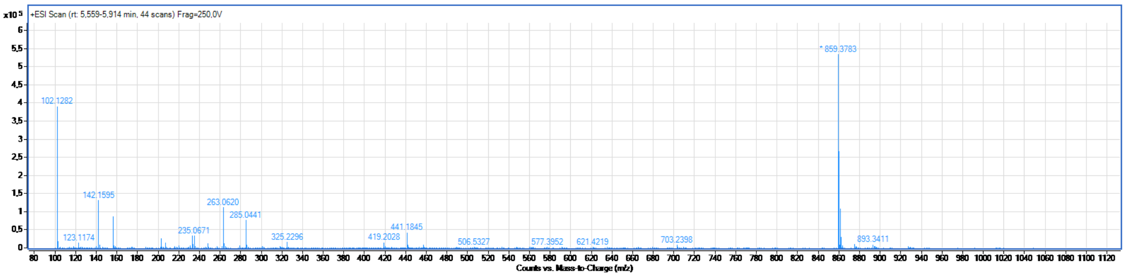

Figure S.179: MS of methyl 8-cyclopropyl-5-oxo-7-(((2,2,6,6-tetramethylpiperidin-1-yl)oxy)methyl)-5H-thiazolo[3,2-a]pyridine-3-carboxylate, **3oa**.

References

[1] F. Monti, A. Baschieri, L. Sambri and N. Armaroli, *Acc. Chem. Res.*, 2021, **54**, 1492–1505.
